# The indole motif is essential for the antitrypanosomal activity of N^5^-substituted paullones

**DOI:** 10.1371/journal.pone.0292946

**Published:** 2023-11-30

**Authors:** Irina Ihnatenko, Marco J. Müller, Oliver C. F. Orban, Jens C. Lindhof, Diego Benítez, Cecilia Ortíz, Estefanía Dibello, Leonardo L. Seidl, Marcelo A. Comini, Conrad Kunick

**Affiliations:** 1 Institute of Medicinal and Pharmaceutical Chemistry, TU Braunschweig, Braunschweig, Germany; 2 PVZ–Center of Pharmaceutical Engineering, TU Braunschweig, Braunschweig, Germany; 3 Laboratory Redox Biology of Trypanosomes, Institut Pasteur de Montevideo, Montevideo, Uruguay; 4 Laboratorio de Síntesis Orgánica, Departamento de Química Orgánica, Facultad de Química, Universidad de la República, Montevideo, Uruguay; kafrelsheikh University, EGYPT

## Abstract

Severe infections with potentially fatal outcomes are caused by parasites from the genera *Trypanosoma* and *Leishmania* (class Kinetoplastea). The diseases affect people of remote areas in the tropics and subtropics with limited access to adequate health care. Besides insufficient diagnostics, treatment options are limited, with tenuous developments in recent years. Therefore, new antitrypanosomal antiinfectives are required to fight these maladies. In the presented approach, new compounds were developed and tested on the target trypanothione synthetase (TryS). This enzyme is crucial to the kinetoplastids’ unique trypanothione-based thiol redox metabolism and thus for pathogen survival. Preceding studies have shown that N^5^-substituted paullones display antitrypanosomal activity as well as TryS inhibition. Herein, this compound class was further examined regarding the structure-activity relationships (SAR). Diverse benzazepinone derivatives were designed and tested in cell-based assays on bloodstream *Trypanosoma brucei brucei* (*T*. *b*. *brucei*) and intracellular amastigotes of *Leishmania infantum* (*L*. *infantum*) as well as in enzyme-based assays on *L*. *infantum* TryS (*Li*TryS) and *T*. *b*. *brucei* TryS (*Tb*TryS). While an exchange of just the substituent in the 9-position of paullones led to potent inhibitors on *Li*TryS and *T*. *b*. *brucei* parasites, new compounds lacking the indole moiety showed a total loss of activity in both assays. Conclusively, the indole as part of the paullone structure is pivotal for keeping the TryS inhibitory and antitrypanosomal activity of this substance class.

## Introduction

### Trypanosomes cause neglected tropical diseases (NTDs)

**Fig 1:**
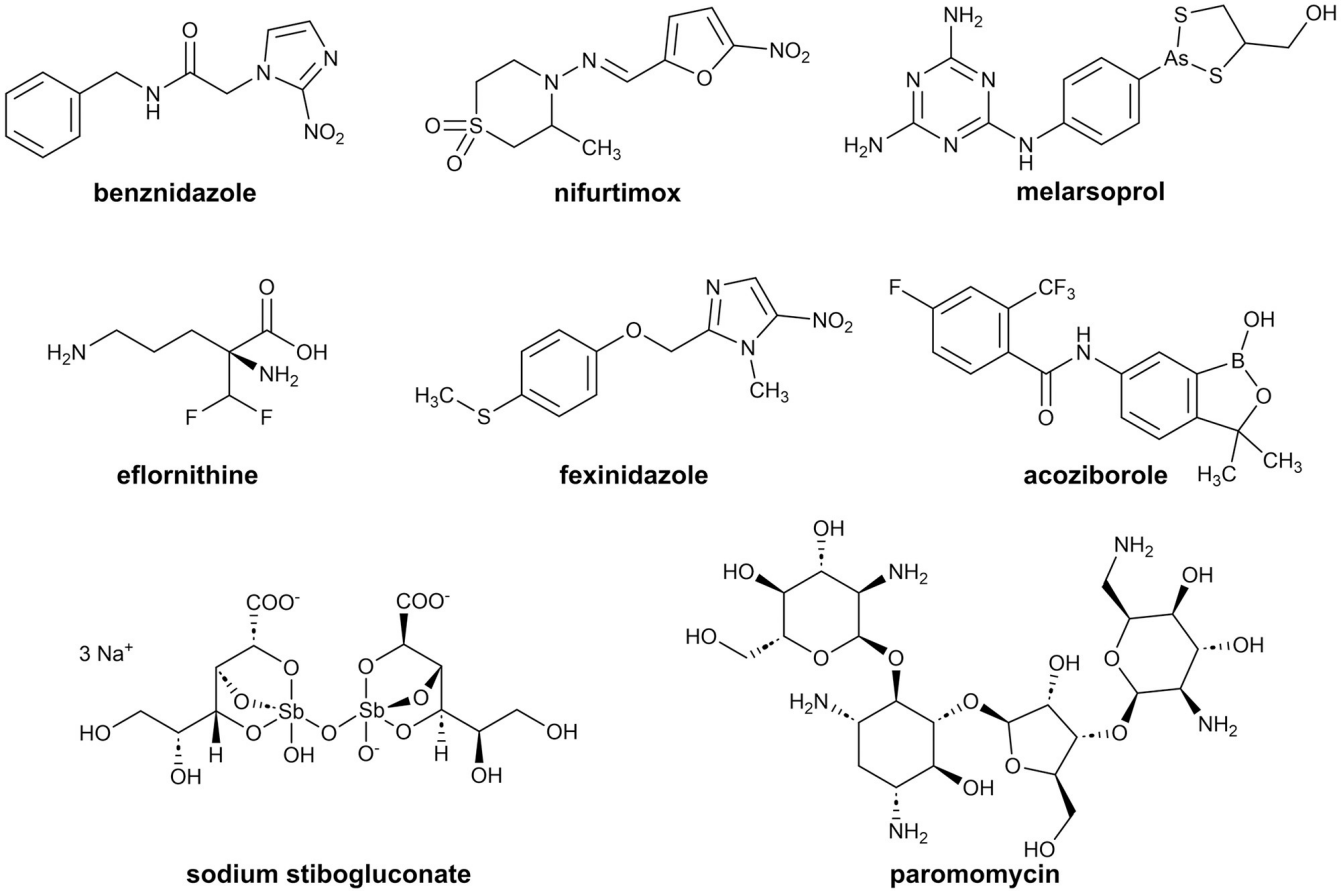
Structures of selected current antileishmanial and antitrypanosomal drugs. Acoziborole is in clinical testing.

Unicellular eukaryotes grouped as kinetoplastids include genera that are pathogenic to humans [1, 2]. Particularly, *Trypanosoma* and *Leishmania* parasites are responsible for the disease burden. Conditions include human African trypanosomiasis (HAT, sleeping sickness) caused by subspecies of *Trypanosoma brucei*, American trypanosomiasis (Chagas disease) caused by *Trypanosoma cruzi* and diverse forms of leishmaniasis caused by *Leishmania* species [3, 4]. These infections are transmitted *via* insect vectors and are classified as neglected tropical diseases (NTDs) by the World Health Organisation (WHO) [4]. NTDs affect more than 1 billion people worldwide, most significantly communities in the tropics and subtropics with insufficient access to health care services [5–7]. Another issue is the low availability of treatments, with the options mainly being old chemotherapeutics whose efficacies depend on the disease stage. Benznidazole and nifurtimox are the current sole options for Chagas disease treatment. African trypanosomiasis treatment includes a broader range of therapeutics like the organic arsenical melarsoprol, a nifurtimox-eflornithine combination and the recently introduced orally applicable fexinidazole. Acoziborole, a promising candidate for oral one-dose treatment of HAT caused by *T*. *b*. *gambiense* is in the drug development pipeline. The diverse forms of leishmaniasis are treated with compounds ranging from antimony-based therapeutics such as sodium stibogluconate, with the last established therapeutic option being the aminoglycoside paromomycin (Fig 1) [8–10].

Antiinfectives constitute a central pillar to combat these diseases besides preventive measures like vector management. One of the goals of organisations like WHO, public-private partnerships and academic institutions alike is to develop new effective medicines to help containing NTDs [5, 11, 12].

### Trypanothione synthetase (TryS) is a validated target

**Fig 2:**
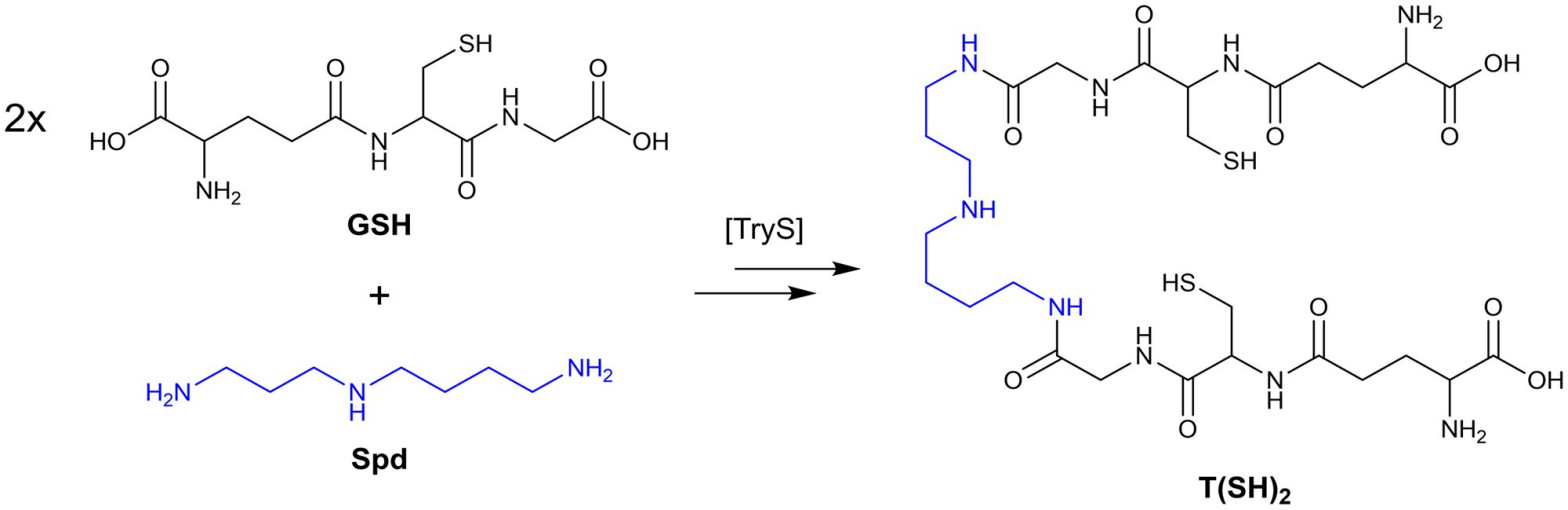
Trypanothione biosynthesis. In kinetoplastids, the *N*^1^,*N*^8^-bis(glutathionyl)spermidine trypanothione (T(SH)_2_) is synthesised from 1 eq spermidine (Spd) and 2 eq glutathione (GSH) through catalysis by TryS.

A well-known biological target or metabolic process is a good starting point for developing new drugs. The enzyme TryS plays a vital role in the parasites’ one-of-a-kind thiol redox metabolism by being the only enzyme that synthesises an essential low molecular weight thiol, trypanothione (T(SH)_2_) (Fig 2) [13]. This dithiol is the functional homologue of glutathione (GSH) and the basis for this metabolism. Like GSH, T(SH)_2_ provides reducing power to the antioxidant defence and other important cellular processes. Indeed, trypanothione possesses advantages over glutathione, including a higher reactivity as demonstrated in *in vitro* redox assays [14, 15].

TryS has been validated as a drug target [16–19]. Recent studies on genetically modified trypanosomatids overexpressing TryS showed survival advantages of the modified organisms in an environment with increased oxidative stress [20]. Due to the importance of TryS for parasite survival and the absence of homologues in mammals, the enzyme represents a valuable target for developing new antitrypanosomal compounds [13, 15].

### The compound class of N^5^-substituted paullones inhibits TryS

**Fig 3:**
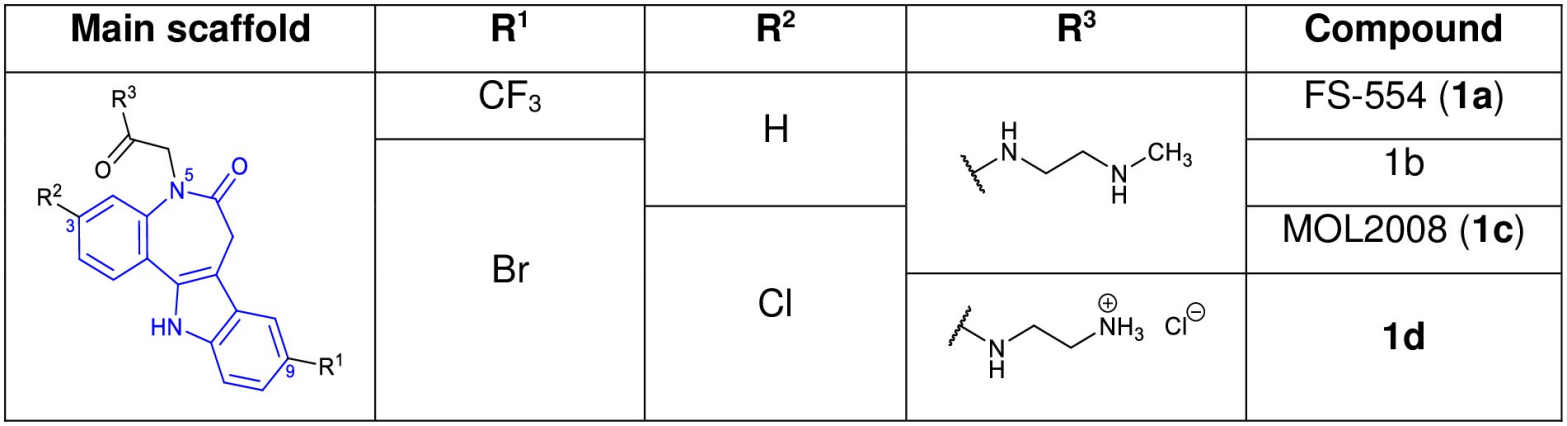
Antitrypanosomal paullones with TryS activity. FS-554 (**1a**) inhibits *Li*TryS (IC_50_ = 0.35 μM). **1b** is a *Crithidia fasciculata* TryS inhibitor (*Cf*TryS, 92% inhibition at 10 μM compound concentration). MOL2008 (**1c**) is a potent *Li*TryS inhibitor (IC_50_ = 0.15 μM). **1d** inhibits *Li*TryS with an IC_50_ = 0.3 μM.

The paullone family of compounds gained attention due to their activity on several protein kinases like cyclin-dependent kinases or glycogen synthase kinase-3 [21–23]. A number of these indole-fused benzazepinones display antitrypanosomal activity on a cellular level. Selected paullone derivatives were tested in enzyme assays on TryS and demonstrated activity (Fig 3) [24–26]. However, other paullones have an antitrypanosomal effect without inhibiting TryS, indicating another, as yet unknown target [27–30].

The main feature of TryS-inhibiting paullones is an N^5^-substitution with a side chain comprising an amide link and/or a basic amino group. In contrast, N^5^-unsubstituted paullones do not show any TryS inhibition [26]. Compared to TryS from *L*. *infantum*, the orthologues from *T*. *cruzi* and, most notably, *T*. *b*. *brucei* were less prone to inhibition by N^5^-substituted paullones. The diverse sensitivity of TryS of different species is explained by the structural features of the respective enzymes [26]. Some challenges, including limited improvements in antiparasitic activity, insufficient solubility in aqueous media, and low selectivity toward mammalian cells, have hampered the development of paullones into antiparasitic drugs. A difficulty in rational structure-based development also arises from the as yet unidentified binding site and orientation of paullones in TryS and incomplete SAR data of the compound class.

**Fig 4:**
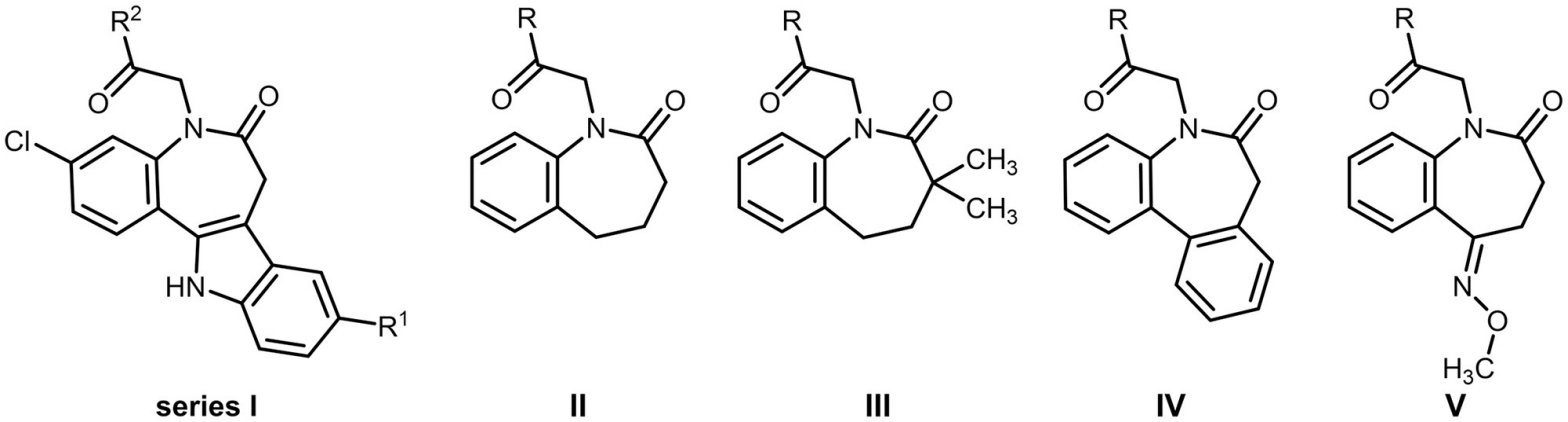
Paullone-derived novel series of benzazepinones I–V. For R, R^1^ and R^2^ please refer to figures in the section **Biological results**.

In consequence, the objectives of this study were to gain further insight into the SAR, to improve antitrypanosomal activity and to optimize the physicochemical properties of bioactive paullone derivatives. Using MOL2008 as a template structure, we started with subtle modifications of the 9-substituent and/or the N^5^-side chain (series I). Then, intending to improve drug-likeness by lowering molecular weight and lipophilicity, we downsized the paullone structure by removing or replacing the annulated indole ring, resulting in series II–V (Fig 4) [31–34]. Series I comprises molecules with combined structural features of MOL2008 and FS-554 and corresponding precursor compounds from the synthetic procedure. Series II consists of unsubstituted benzazepinones, and series III includes dimethylated analogues of series II. In series IV, the indole scaffold is replaced by a benzene ring, resulting in dibenzazepinone. Finally, in series V, the indole is replaced by a ketoxime O-methyl ether moiety. The series were systematically selected to keep production costs low for potential drugs against neglected infectious diseases by considering ease of accessibility to starting materials and developing straightforward synthetic procedures with a minimal number of steps [35].

## Results and discussion

### Molecular docking studies

#### The rationale for docking in TryS

**Fig 5:**
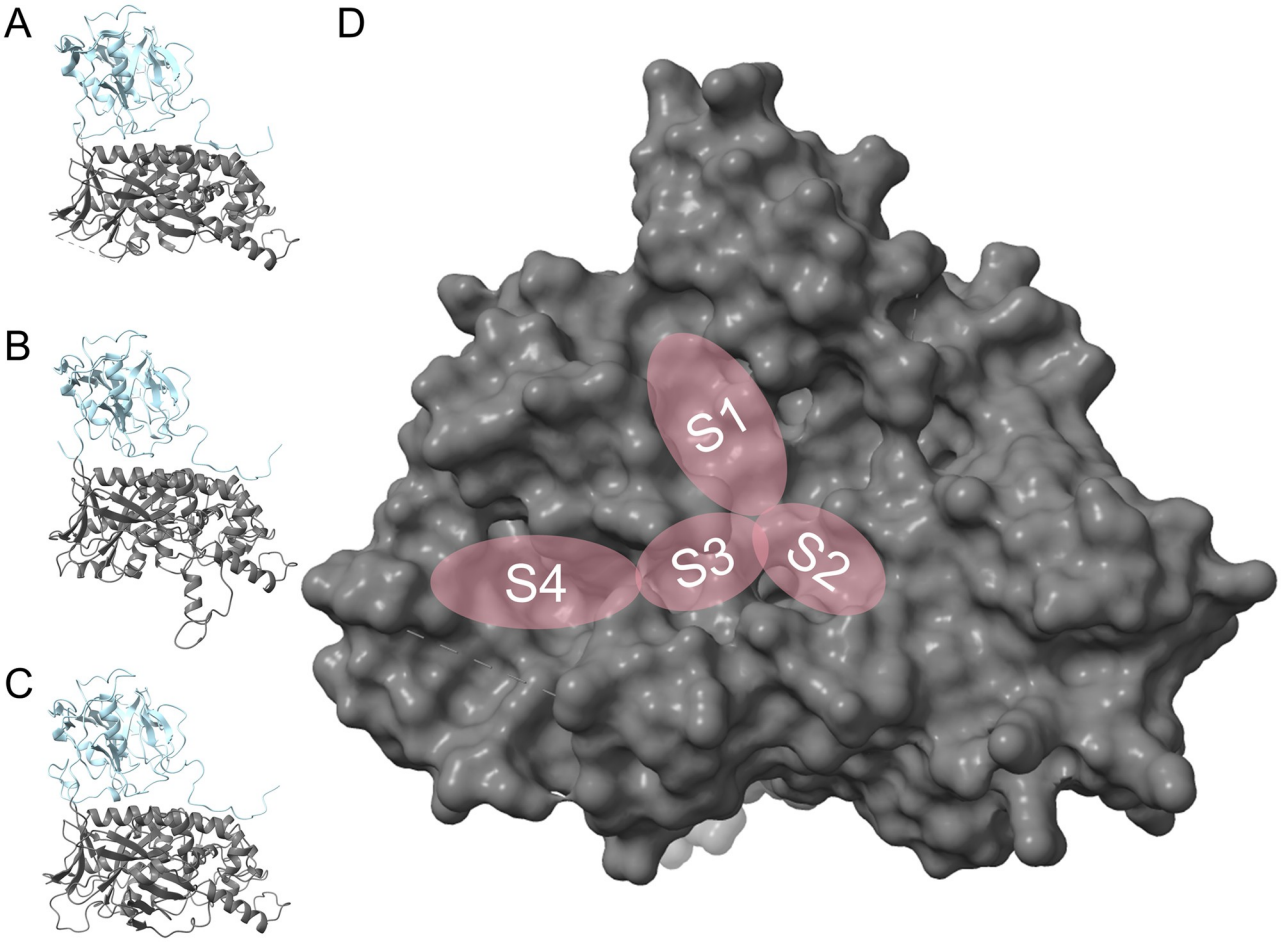
TryS structures and details of the synthetase domain. (A) The *Leishmania major* trypanothione synthetase (*Lm*TryS) crystal structure 2VPS. (B) The *Li*TryS homology model generated by superposition. (C) The *Li*TryS model generated by AlphaFold. (D) View onto the N-terminal synthetase domain of 2VPS with the binding sites for ATP (S1), GSH (S2), Spd (S3) and Gsp (S3/S4). The amidase and the synthetase domains are coloured in light blue and in grey, respectively.

As TryS is the target of the presented investigations, computer-aided drug design was performed on enzyme models. The first elucidation of a TryS enzyme was presented in 2008 by Fyfe *et al*. by X-ray diffraction and resulted in three crystal structures of *Leishmania major* TryS (*Lm*TryS, PDB: 2VPS, 2VPM, 2VOB) [36]. However, no co-crystal structure of TryS and a substrate is available to date. The analysis mentioned above by Fyfe *et al*. revealed two domains of interest: firstly, the N-terminal amidase domain and, secondly, the C-terminal synthetase; both sites are influencing each other’s activity through conformational changes. The latter domain contains an adenosine triphosphate (ATP) grasp fold found in synthetases and kinases [36, 37]. This ATP binding site (S1) is arranged together with a GSH binding site (S2) and an Spd/Gsp site (S3/S4) to form a trifoliate shape. Here, the synthesis of trypanothione happens in two steps: Firstly, GSH is ligated to Spd, and secondly, glutathionylspermidine (Gsp) is bound to another GSH [38–40]. These two steps are both mediated by ATP through activation of the glycine carboxy group of GSH for the subsequent amide coupling to form the product. Further studies identified another binding site (S4) to accommodate the GSH part of a Gsp molecule in the inverted orientation for the second ligation (Fig 5) [41]. Although the mechanism between TryS of different species matches, the binding site geometries vary. The variation leads to species-dependent activities of inhibitors, e.g. the N-substituted paullones showing the highest activity on *Leishmania* TryS [26, 27].

Two main theories of binding paullones to TryS are the basis for this work and are discussed below. The first proposes that paullones bind to the ATP site (S1), and the second suggests that they bind to the Spd/Gsp site (S3/S4). The first hypothesis was rationalised on the basis that paullones were primarily discovered as kinase inhibitors with targets including some cyclin-dependent kinases and glycogen synthase kinase-3 (GSK-3) [21, 22]. Here, they act ATP-competitively, as shown by enzyme kinetics [37] and co-crystallisation studies in the ATP binding site [42]. The binding of alsterpaullone to GSK-3β leaves no room for the N^5^-side chain substituents of the paullone. This can be explained by a possible clash of the N^5^-side chain with the hinge segment Tyr134–Val135 when keeping the binding mode of alsterpaullone [27, 42].

In contrast, the modelled binding mode of paullones in the ATP site of TryS leaves room for diverse N^5^-substituents, as this part is oriented towards solvent-accessible space [27]. Particularly a substitution with side chains bearing basic functionalities led to structures with activity against *Li*TryS and selectivity towards the enzymes of other species (*Tc*TryS and *Tb*TryS). The binding mode was generated by the alignment to the similarly structured ATP site of GSK-3 occupied by alsterpaullone, showing a comparable hydrogen bonding pattern [26]. This modelling serves as the primary basis for the development of the presented compounds by molecular docking and SAR.

Kinetic characterisation of the inhibition mechanism exerted by MOL2008 (**1c**) on *Li*TryS showed that it is competitive to Spd and Gsp, and uncompetitive to ATP and GSH [25, 26]. This mode of inhibition was further supported by isothermal titration calorimetry assays that showed an increase in compound affinity when GSH and/or adenosine diphosphate (ADP) were bound to the enzyme [26]. This experimental evidence led to the hypothesis that the basic side chain of paullones is possibly responsible for the competition with the likewise basic native polyamine substrates. Thus, molecular docking experiments of possible binding modes of paullones and related benzazepinones to the Spd/Gsp binding site are presented below.

#### The basis for the docking models used

**Fig 6:**
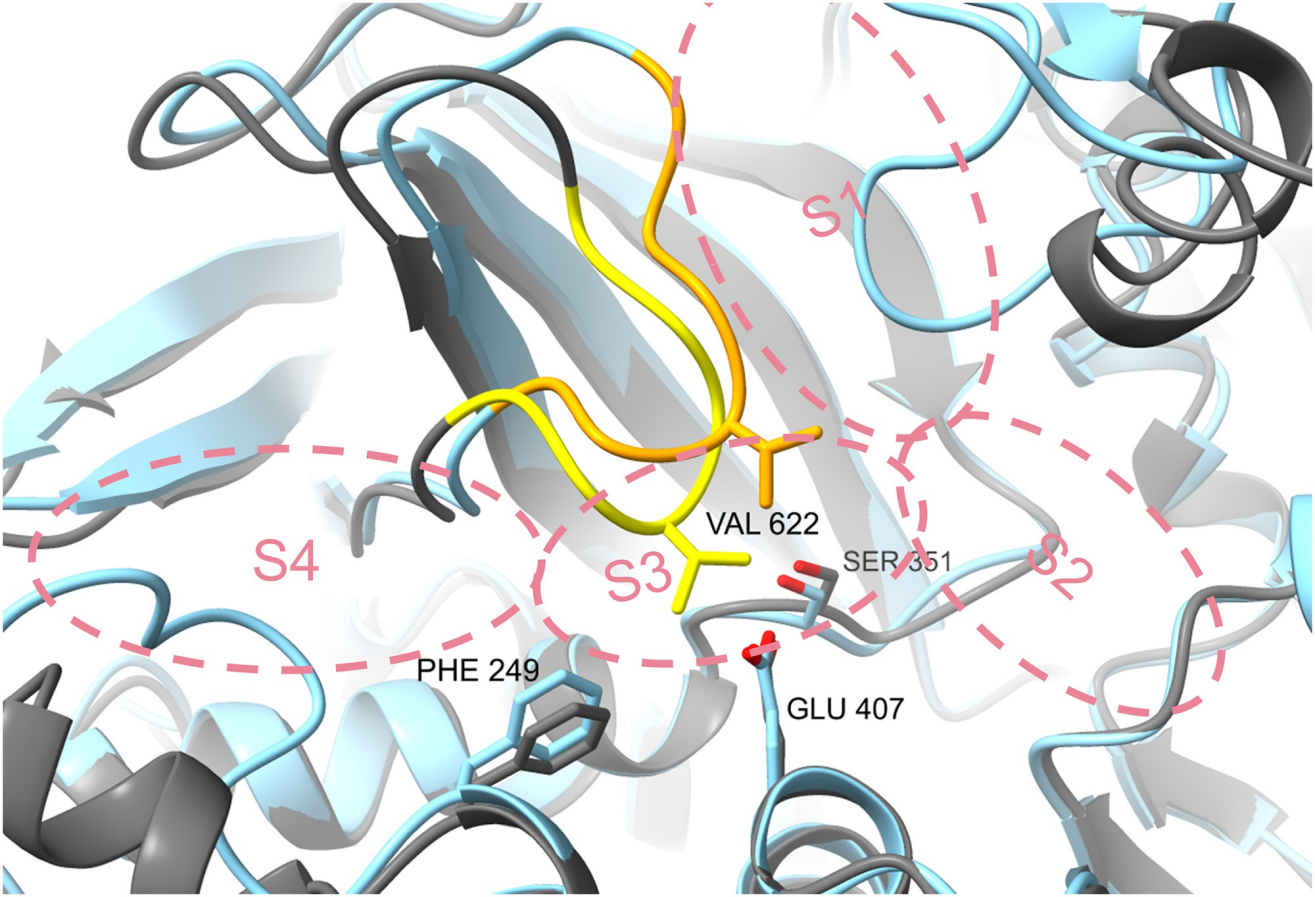
Comparison of the TryS homology model and the AlphaFold model synthetase active site. The *Li*TryS homology model (grey) differs from the AlphaFold generated model (cyan) in the location of the segment Ile619 to Ser624 (yellow / orange). S1–4 as explained in Fig 5. The proximity of Val622 in the homology model leaves no room for substrate binding in the Spd site.

The revealed structures of TryS from Fyfe *et al*. have the drawback of containing an unresolved flexible loop adjacent to the S4 binding site and no native substrates in the possible binding region. Thus, this model is not adequately suited for docking studies. Therefore, we based our docking studies on homology models [41] or AlphaFold-predicted three-dimensional structures [43, 44]. The homology models are based on molecular dynamics investigations of TryS and glutathionylspermidine synthetase (GspS) [41, 45]. As paullones show a higher activity on *Leishmania* TryS, the *Li*TryS homology model was used in the presented studies. AlphaFold-generated structures were used for docking to the Spd/Gsp site, as the homology model presented above has a different orientation in a segment (Ile619 to Ser624) that shapes the polyamine binding site, compared to the protein crystal structure. In the homology model, the Val622 side chain is oriented to separate the Spd/Gsp site from the central active site (Fig 6). The mentioned region is structurally more similar between the crystal structure (PDB: 2VPS) and the AlphaFold structure (A4I2Z3_LEIIN). Since no water molecules are part of the models used, neither a thermodynamic nor a kinetic binding profile can be predicted [46].

#### Docking into the ATP site

**Fig 7:**
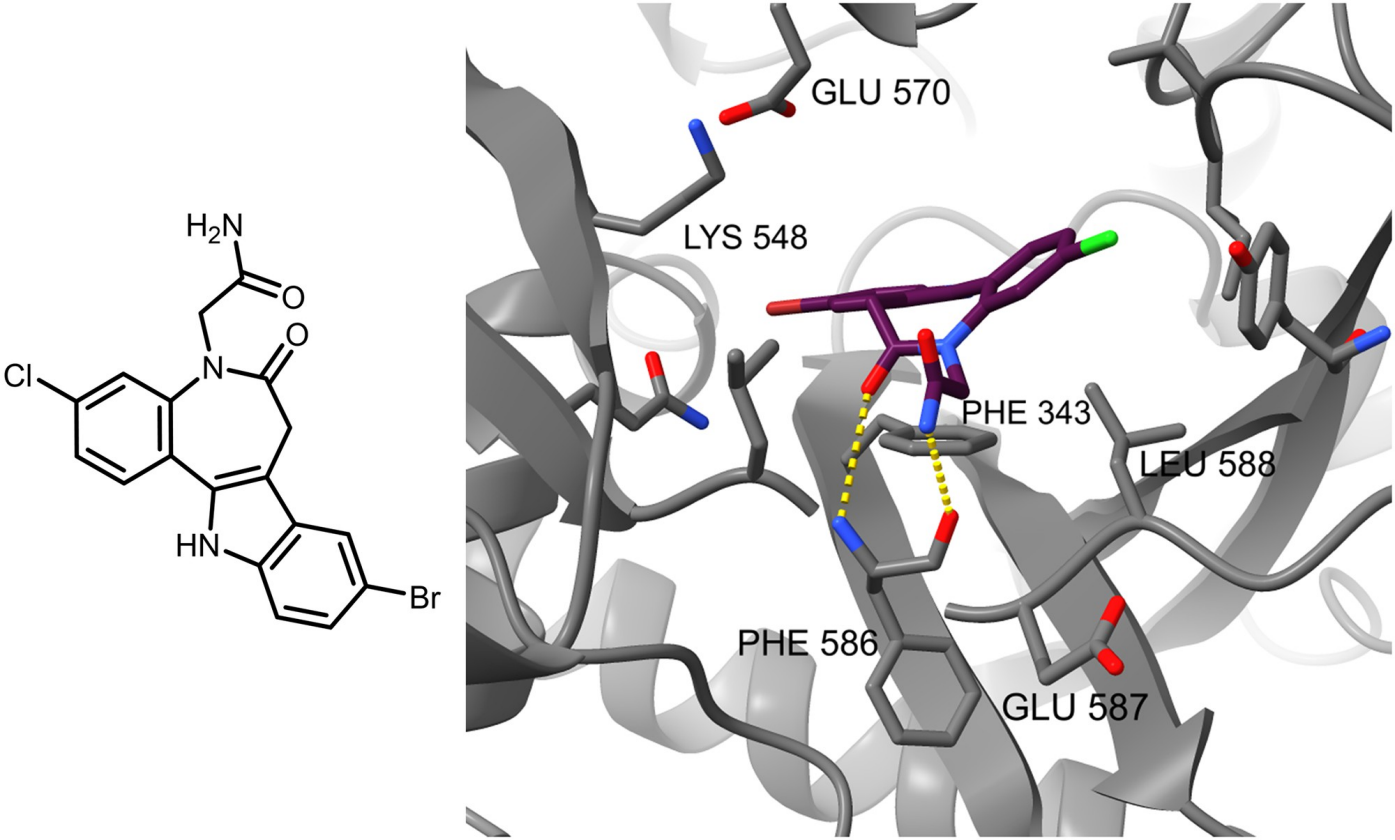
Molecular docking binding mode of 1e in the homology model, a basis for further docking investigations. Hydrogen bonds towards the Phe586 backbone are displayed in cyan and serve as a template for setting constraints.

The presented docking studies in the ATP binding site are based on a docking model of ATP bound into its respective site of *Lm*TryS [27, 41]. In the displayed model (Fig 7), the paullone-*N*^5^-acetamide (**1e**) shows no interaction with Lys548, a residue engaged in interactions with the purine *N*^1^ from ATP. Here, the 9-bromo substituent of the paullone occupies a pocket formed as the lysine side chain is repositioned to interact ionically with the terminal carboxyl group of Glu570. However, similarly to ATP, the indole partial structure of the paullone displays π-stacking with Phe343 and hydrogen bonding of the benzazepinone carbonyl group with the Phe586 NH. One further hydrogen bond is found between the paullone side chain amide NH to the carboxyl group of the Phe586 side chain. The benzazepinone moiety is located in an open hydrophobic region where the Leu588 side chain interacts with the benzene ring.

The above displayed pose of **1e** is difficult to reproduce without constraints for the kenpaullone derivative itself, and also for the designed compounds of series I–V (scaffolds presented in Fig 4). The molecular docking into the ATP site without constraints leads to conformationally and locationally diverse results (not shown). Thus, constraints were used as follows: hydrogen bonds from the backbone NH and CO of Phe586 of the protein to an arbitrary part of the docked ligand.

**Fig 8:**
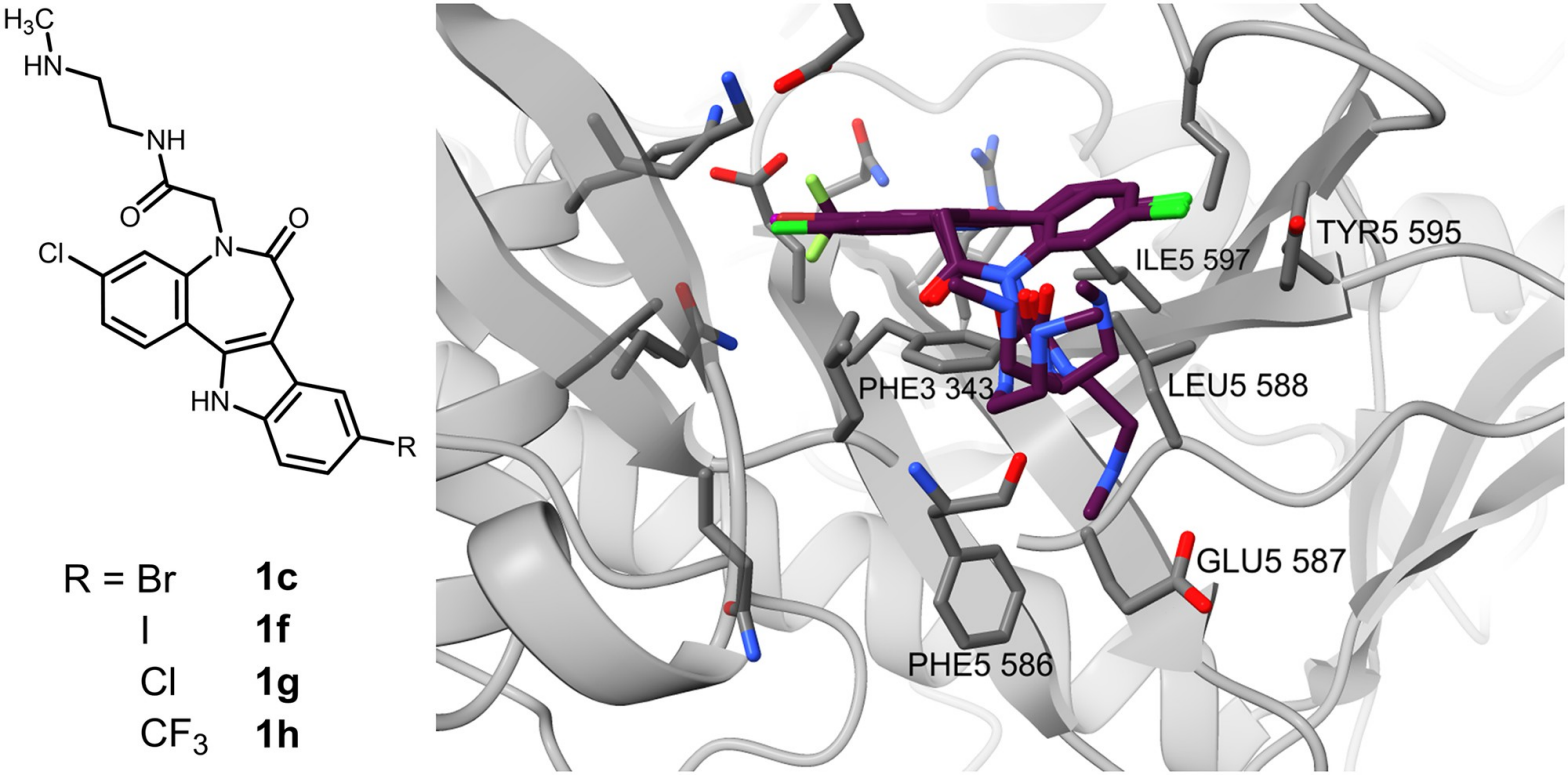
Molecular docking binding mode of MOL2008 (1c) and series I paullone-acetamides (1f–h) in the ATP site (S1) of the homology model.

The paullone derivatives (series I) showed a similar mode across the differently 9-substituted derivatives (Fig 8), with some of the poses deviating, most pronounced for the 9-trifluoromethyl derivative **1h** displaying various rotations of the central hydrophobic scaffold. The paullones’ side chain amide NH almost always acted as an H-bond donor of the Phe586 carbonyl group. Despite the other constraint set to Phe586 NH, the distance between the hetero atoms of the donor and the acceptor from the ligand was over 4 Å. The azepinone carbonyl acts hereby as the closest possible acceptor. Another important recovery of an interaction is the π-stacking between Phe343 and the indole present across all the docked paullones. A possible secondary interaction would be a halogen bond between the 3-chloro substituent of the paullones and the Tyr595 aromatic ring. However, the geometry is not optimal for such an interaction in the displayed poses meaning the prerequisites for halogen bond formation are not met [47]. The 9-halogen substituent of the paullones occupies a hydrophobic pocket as described above for **1e**. The flexible side chain with the basic methylammonium partial structure is charged under physiological conditions and, for some poses, shows ionic interactions with the terminal carboxylate of Glu587. However, the side chain is as likely to be oriented into the solvent-accessible space.

**Fig 9:**
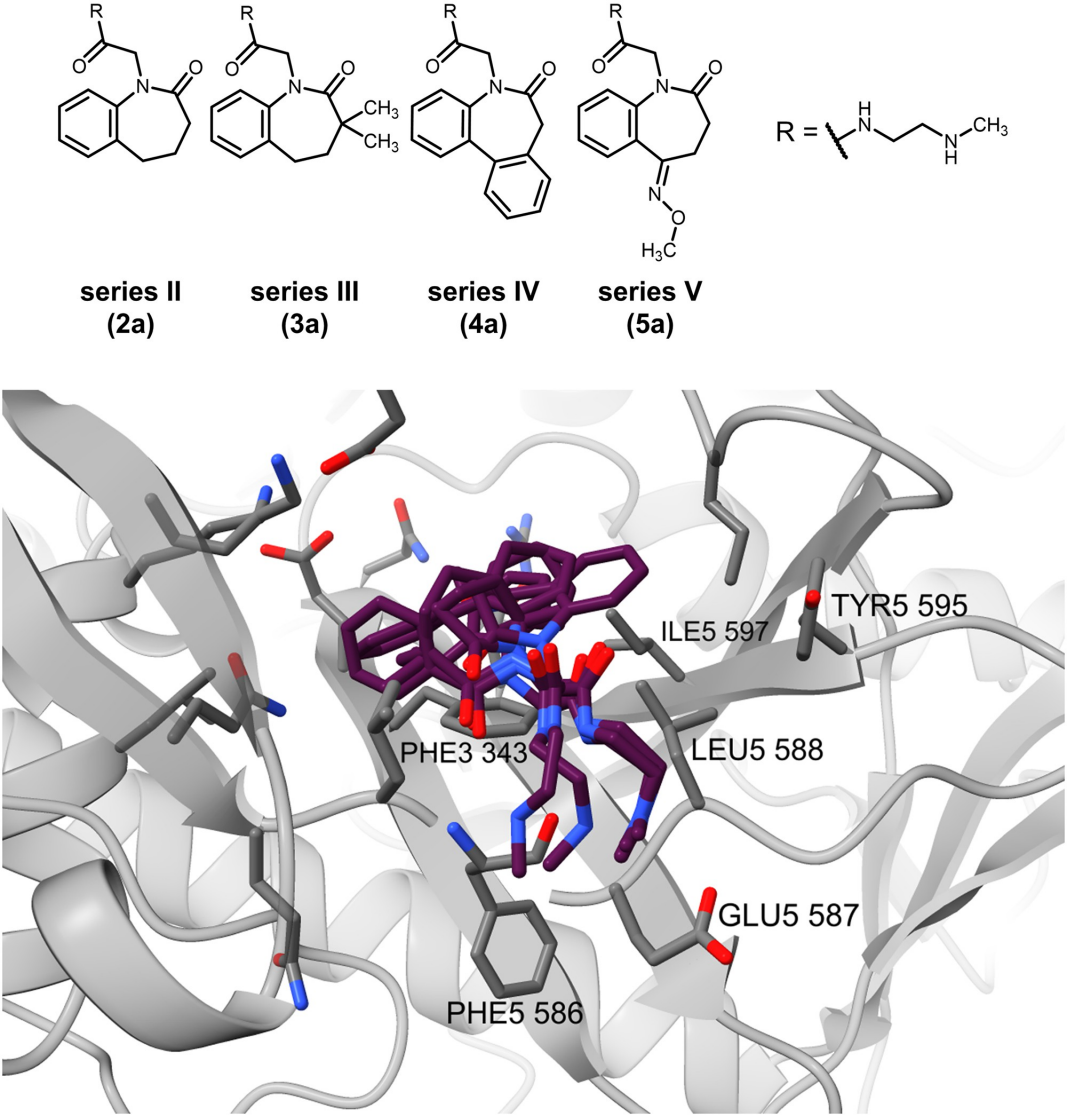
Molecular docking binding mode of benzazepinones of series II–V (2a, 3a, 4a, 5a) in the ATP site (S1) of the homology model.

The benzazepinones of series II–V show an even more diverse set of conformations and positions in the binding site (Fig 9). The two constraints are fulfilled well by either a binding mode similar to the paullones’ or closer proximity to the Phe586 NH for possible hydrogen bond formation. Alternatively, the two set hydrogen bonds are formed to the ligands’ nitrogens of the side chain, relocating the main scaffold deeper into the binding pocket (not shown). The central scaffold in the shown orientation is very diversely located, e.g. the benzene ring being close to the Leu585 side chain or flipped towards Phe343, Leu588 or Ile597 for possible hydrophobic interactions. The basic side chain is oriented towards the solvent-accessible space or the Glu587 side chain, similar to the paullones’ orientation. Overall, there is little coherence for the binding patterns across the benzazepinone derivatives, especially for the main scaffold.

As observed in the diverse poses of the paullones and designed benzazepinones, an essential partial structure for interactions would primarily be the lactam, the side chain amide and a basic alkylamine. Secondarily, larger hydrophobic structures, such as the indole, could interact with Phe343. One possibility to improve the physicochemical properties of the compounds, while preserving their affinity for the molecular target, would be to cut short on the hydrophobic interactions and instead establish a salt bridge between the basic paullone side chain and the terminal carboxyl group of Glu587. As the side chain is exposed on the protein surface, larger basic substituents like alkyl piperazines could be tolerated.

#### Docking into the Spd/Gsp site

Kinetic, thermodynamic and computational approaches suggested that paullones occupy the Spd/Gsp binding site of TryS [26, 48]. Thus, the paullone derivatives were docked into the Spd/Gsp site to analyse an alternative plausible binding mode.

To define a binding site for docking in the Spd/Gsp site, a central atom was used for a 10 Å radius to specify the cavity. Either the Glu407 carboxyl O1 was used, as it is located in the Spd binding site close to the catalytic centre and is also considered important for an interaction with the polyamine substrates [36, 41], or the Phe626 backbone NH as it is located in the centre of the Spd/Gsp site. As highlighted above, the homology model of *Li*TryS was not suitable for docking in the polyamine binding site because the orientation of the Val622 side chain prevents the bound molecules to accommodate alongside this binding region and interact with residues from the catalytic site. This was confirmed with docked paullones, which adopted low scored and upright poses in this site.

**Fig 10:**
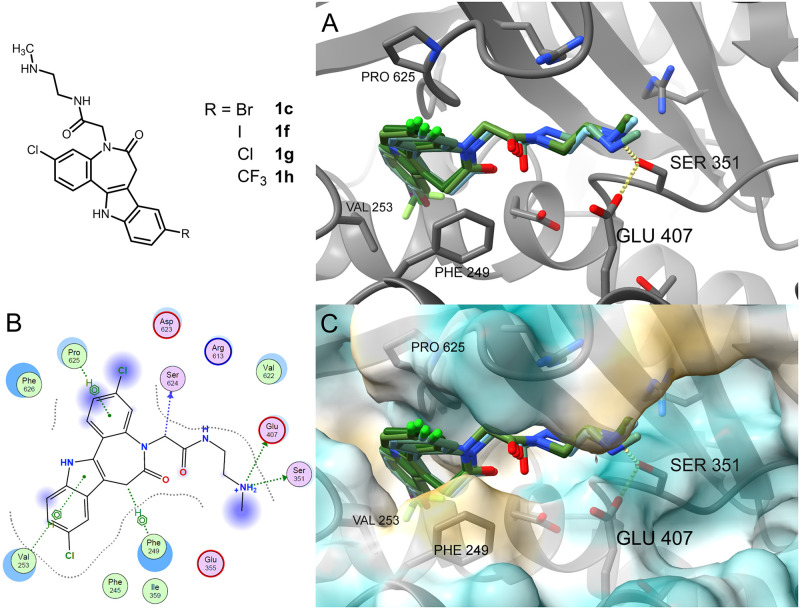
Molecular docking of paullones into the Spd/Gsp site. (A) Binding mode of MOL2008 (**1c**) and series I paullone-acetamides (**1f**–**h**) in the Spd/Gsp site (S3/S4) of the AlphaFold generated model (A4I2Z3_LEIIN). (B) Ligand interactions calculated for compound **1g** by MOE. (C) Molecular lipophilicity surface map of the AlphaFold generated model calculated by ChimeraX with colouring ranging from golden (most lipophilic) to cyan (most hydrophilic) with the docked compounds (**1c**,**f**–**h**; see panel A).

The AlphaFold model of *Li*TryS (overall RMSD 1.15 Å to 2VPS), where the Spd site is connected to the catalytic centre, was used instead. Here, the highly scored binding mode across the paullones is dominated by hydrogen bonds or ionic interactions to Ser351 or Glu407 (Fig 10). The 3-substituent of the scaffold is more exposed, while the 9-substituted indole part is located deeper in the cavity. The paullone scaffold forms hydrophobic interactions with Phe249, Val253 and Pro625. Particularly, the hydrogen bonds are important, as the structural elements of Ser351 and Glu407 are presumably necessary to align the terminal amine of the native substrates Spd or Gsp for the ligation to GSH to form the products Gsp or T(SH)_2_ [36, 41]. Hence, by binding in the depicted way, the paullone derivatives can compete with the native polyamine substrates, prevent formation of T(SH)_2_ and inhibit TryS.

**Fig 11:**
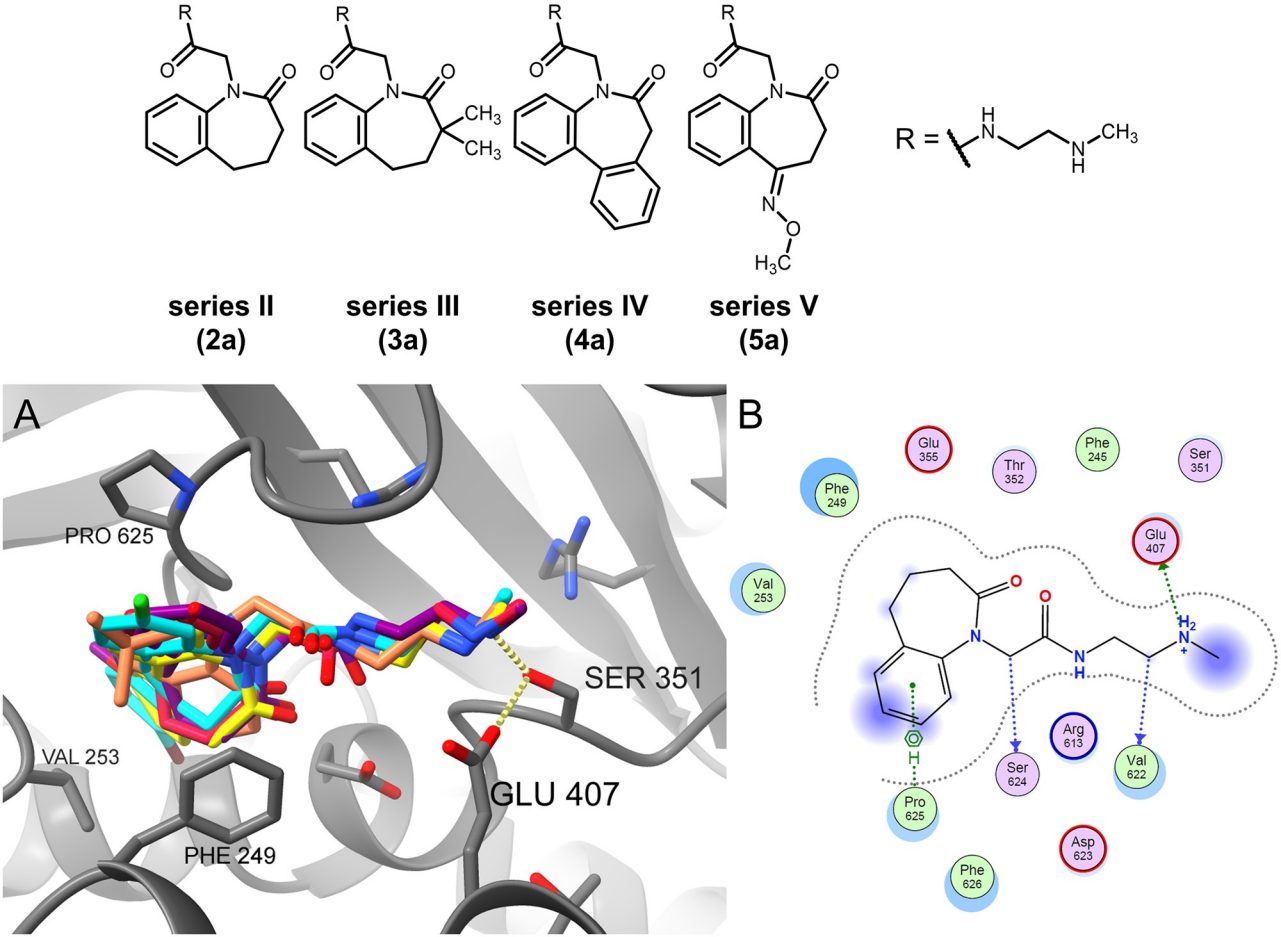
Molecular docking of benzazepinones into the Spd/Gsp site. Series II–V compounds (**2a**: crimson, **3a**: coral, **4a**: yellow; **5a**: purple) show a similar binding mode to series I examples and MOL2008 (cyan) with less lipophilic interactions as seen in the binding mode visualised in ChimeraX (A) and ligand interactions map for compound **2a** calculated by MOE (B).

The benzazepinones of series II–V show a similar orientation in the Spd/Gsp site to series I compounds, with the amine side chain of the ligands mostly pointing towards the central catalytic site (Fig 11). Likewise, the interactions of the basic side chain of the docked compounds to the Ser351 side chain hydroxyl-group and/or Glu407 side chain carboxylate are retained. A greater deviation is visible in the exact position of the benzazepinone core. Furthermore, the smaller benzazepinone core leads to fewer lipophilic interactions than the paullones. The benzazepinone scaffolds interact with Pro625, and additionally for the series IV example, with Phe249 and Val253.

#### Conclusion of the docking experiments

The true binding mode of the docked substrates remains unknown as no co-crystal structure is available. The most reasonable results with the highest scoring by GOLD (docking software) are poses occupying the Spd/Gsp site with the aminoalkyl side chain oriented towards the catalytic site of the synthetase domain and interacting with the side chains of Glu407 and Ser351, which are essential for the native substrate orientation towards the active site. The following series of concerted recognition events taking place during paullone binding to leishmanial TryS could be reasonable: 1) primary binding of the N^5^-substituent at the polyamine binding site of the enzyme that, along with GSH (S2) and ATP (S1) binding, triggers, 2) a conformational change at the adjacent loop to the S4 site (residues 248–263 of *Li*TryS) that 3) favours the access of the paullone scaffold to the hydrophobic and, otherwise, solvent hidden S4 site of the enzyme and, 4) anchors the indole and benzazepinone rings of the paullone *via* hydrophobic interactions with several vicinal residues (Phe245, Phe249, Val253, Ile359, Pro625, Phe626).

A more accurate docking prediction could be performed by docking the paullones into a model of the enzyme with either ATP/Mg^2+^ and GSH or phosphorylated GSH bound. Ensemble docking with the suggested substrates bound might lead to results closer to the paullones’ true binding mode [49], supported by molecular dynamics simulations and by enzyme kinetic and thermodynamic studies [26, 41]. Here, the uncompetitive behaviour of MOL2008 (**1c**) towards ATP/GSH is described with a higher binding affinity of the paullone with these substrates present. As substrate binding leads to a conformational change, the Spd/Gsp site geometry may be altered by the other substrates bound in a more favourable way to incorporate the paullones. Other TryS inhibitors from the literature showed Spd competitive behaviour, such as the aminoalkyl substituted phenothiazine prochlorperazine (DDD66604 in Torrie *et al*. [18]) which possesses a basic side chain and a flat, hydrophobic main scaffold.

### Syntheses

**Fig 12:**
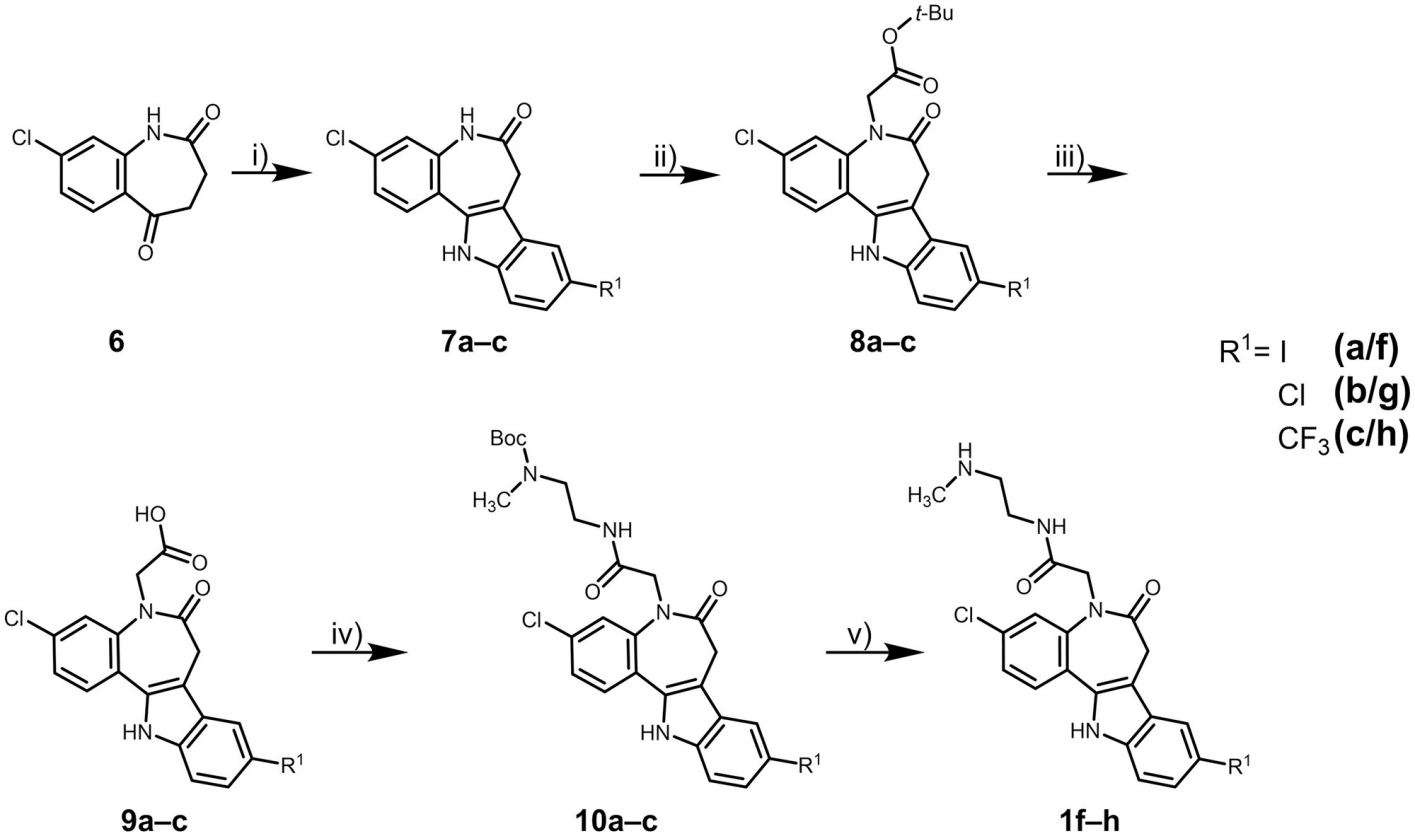
Syntheses of paullone-acetamides (3,9-disubstituted derivatives of 7,12-dihydrobenzo[2,3]azepino[4,5-*b*]indol-6(5*H*)-ones, series I). Reagents, conditions and yields: i) 1. 4-iodophenylhydrazine (for **7a**) / 4-chlorophenylhydrazine hydrochloride + NaOAc (for **7b**) / 4-(trifluoromethyl)phenylhydrazine (for **7c**), AcOH, 70°C, 75–90 min; 2. AcOH, H_2_SO_4_, 70°C, 1.25–2.5 h, 57–75%; ii) 1. THF, *tert*-BuOK, 0°C (RT for **8a**), N_2_, 60–75 min; 2. *tert*-butyl bromoacetate, 0°C → RT, 22–23 h, 40–62%; iii) CH_2_Cl_2_, TFA, N_2_, RT, 5–18 h, 18–58%; iv) DMF, DIPEA, HOBt, EDCl, *N*-*tert*-butoxycarbonyl-*N*-methylethane-1,2-diamine, RT, argon, 17–21.5 h, 51–89%; v) CH_2_Cl_2_, TFA, RT, argon, 23 h, 5–61%.

The compounds of series I were synthesised by a Fischer indole reaction of the cyclic ketone **6** [27, 50] with suitable 4-substituted phenylhydrazines to obtain the new paullones **7a**–**c**, which were subsequently N^5^-alkylated with *tert*-butyl bromoacetate. N^12^-alkylated side products were removed by column chromatography. Cleavage of the *tert*-butyl esters **8a**–**c** by means of trifluoroacetic acid (TFA) led to the corresponding paullone acetic acids **9a**–**c**, which were then coupled to *N*-*tert*-butoxycarbonyl-*N*-methylethane-1,2-diamine using 1-ethyl-3-(3-dimethylaminopropyl)carbodiimide hydrochloride (EDCl), yielding the amides **10a**–**c**. Deprotection of the respective carbamates yielded the corresponding amines **1f**–**h** (Fig 12).

**Fig 13:**
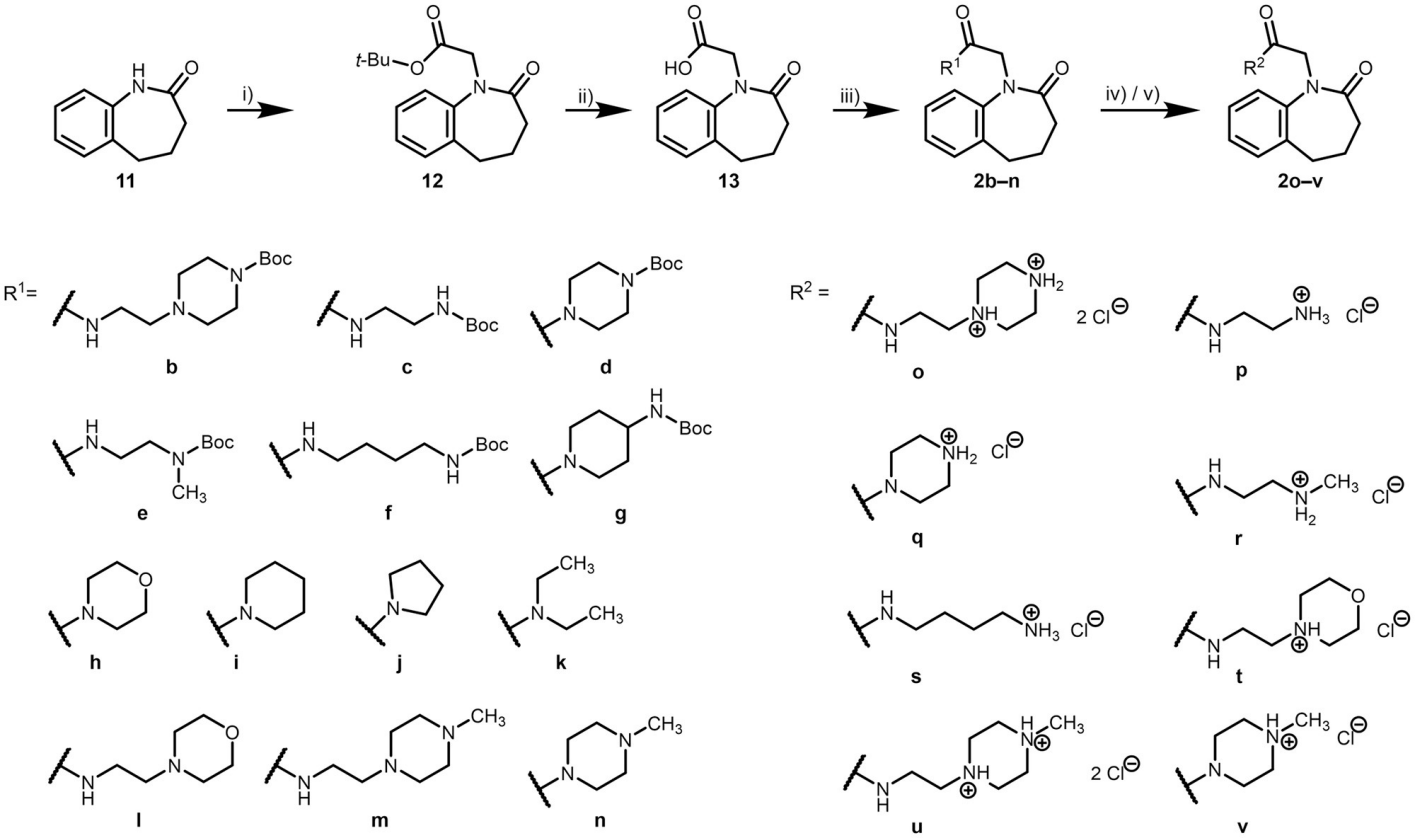
Syntheses of 2-(2-oxo-2,3,4,5-tetrahydro-1*H*-benzo[*b*]azepin-1-yl)acetamides (series II). Reagents, conditions and yields: i) 1. THF, *tert*-BuOK, RT, N_2_, 1 h; 2. *tert*-butyl bromoacetate, RT, N_2_, 2.5–21 h, 49–83%; ii) H_3_PO_4_ (85% w/w), toluene, RT, 4–20 h, 6–46%; iii) DMF, DIPEA, HOBt, EDCl, amine, RT, argon, 22–40 h, 9–83%; iv) 1. CH_2_Cl_2_, TFA, RT, argon, 20–24 h; 2. propan-2-ol, HCl, Et_2_O or *n*-hexane, reflux, 1 h, 12–66% (**2o**–**s**); v) propan-2-ol, HCl, Et_2_O or *n*-hexane, RT, 1 h, 23–65% (**2t**–**v**).

The synthesis of N-substituted benzazepinones belonging to series II is displayed in Fig 13. Alkylation of the starting material **11** [51] with *tert*-butyl bromoacetate led to ester **12**, which subsequently was deprotected using phosphoric acid in toluene according to a procedure reported by Li *et al*. [52]. The resulting acetic acid derivative **13** was coupled to a diverse set of amines resulting in Boc-protected (**2b**–**g**), neutral (**2h**–**k**) and basic (**2l**–**n**) derivatives. The carbamates **2b**–**f** were deprotected using TFA to obtain free amines, which were converted to the hydrochlorides **2o**–**s** by treatment with hydrochloric acid. The amines which resulted directly from the amide coupling (**2l**–**n**) were likewise converted to hydrochlorides (**2t**–**v**).

**Fig 14:**
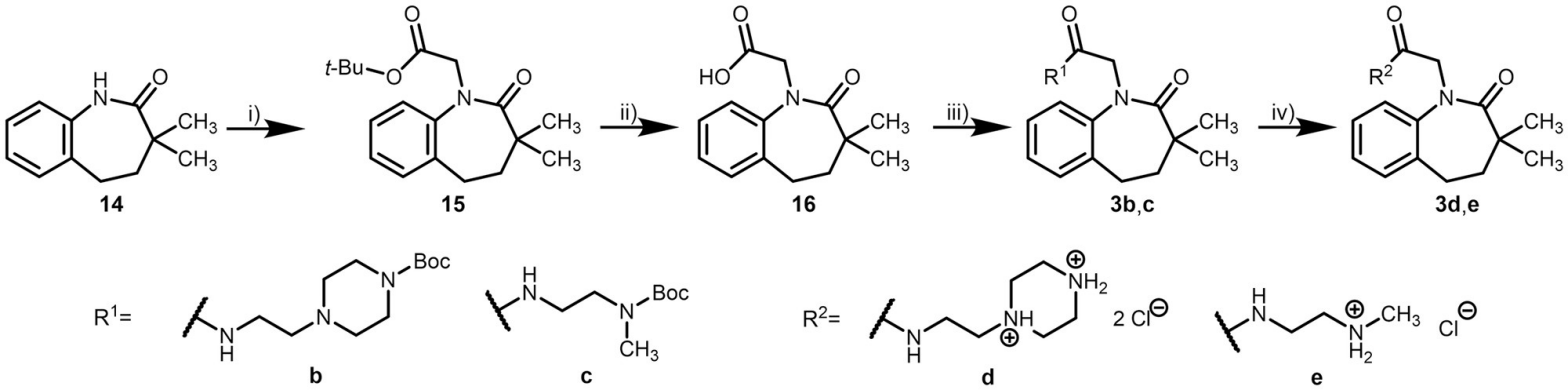
Syntheses of 3,3-dimethyl-2-(2-oxo-2,3,4,5-tetrahydro-1*H*-benzo[*b*]azepin-1-yl)acetamides (series III). Reagents, conditions and yields: i) 1. THF, *tert*-BuOK, RT, N_2_, 1 h; 2. *tert*-butyl bromoacetate, RT, N_2_, 9 h, 50%; ii) CH_2_Cl_2_, TFA, RT, argon, 22 h, 76%; iii) DMF, DIPEA, HOBt, EDCl, amine, RT, argon, 24 h, 30–68%; iv) 1. CH_2_Cl_2_ (**3d**), TFA, RT, argon, 18–43 h; 2. propan-2-ol, HCl, *n*-hexane, RT, 18 h, 36–46%.

For compounds of series III, the dimethylated benzazepinone **14** [53] was alkylated by the same procedure described for series II. Deprotection of the *tert*-butyl ester **15** was carried out with trifluoroacetic acid in dichloromethane to yield **16**. The above-described procedure for amide coupling suggested by Dou *et al*. [54] led to amides with a carbamate side chain that was further deprotected to gain the corresponding amines (Fig 14).

**Fig 15:**
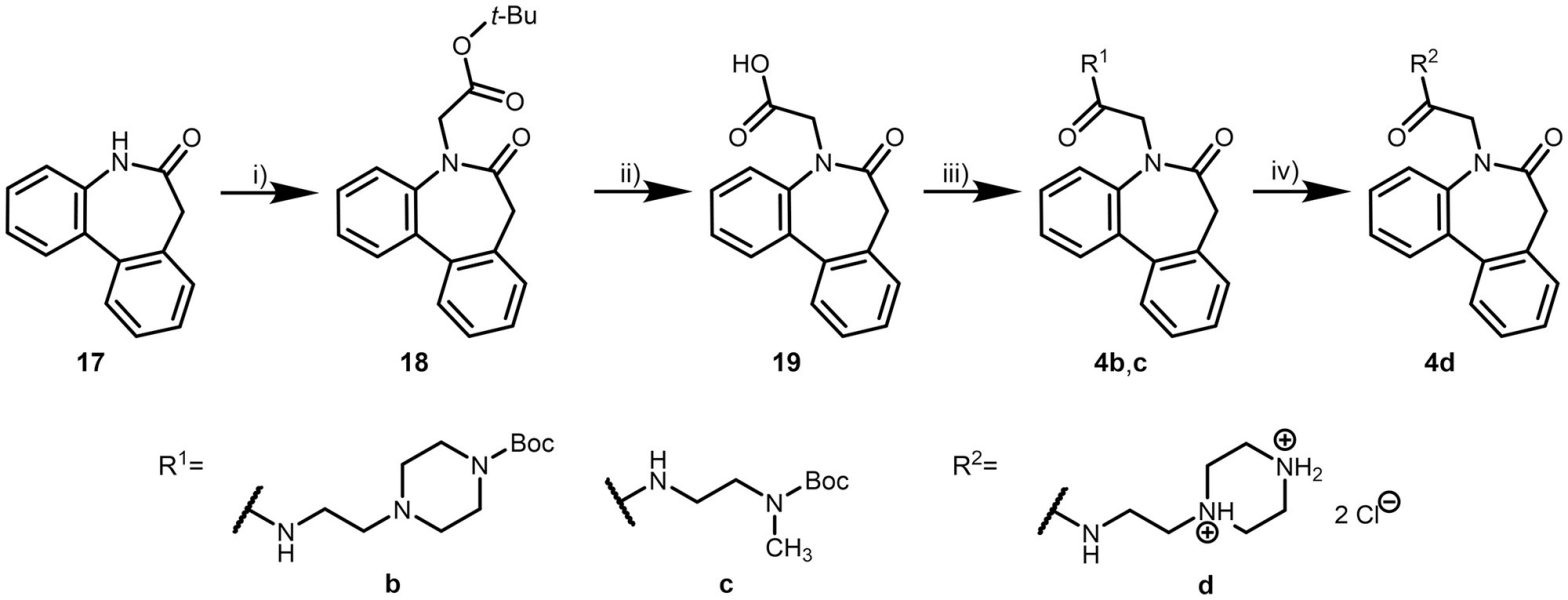
Syntheses of dibenzo[*b*,*d*]azepinylacetamides (series IV). Reagents, conditions and yields: i) 1. DMF, *tert*-butyl bromoacetate, *tert*-BuOK, 0°C → RT → 40°C → 30°C, N_2_, 21 h, 17%; ii) CH_2_Cl_2_, TFA, RT, argon, 23 h, 87%; iii) HOBt (**4b**), DMF, DIPEA, EDCl, amine, RT, argon, 21–95 h, 26–78%; iv) 1. CH_2_Cl_2_, TFA, RT, argon, 18 h; 2. propan-2-ol, HCl, *n*-hexane, RT, 18 h, 31%.

The synthetic procedures to obtain dibenzazepinones of series IV are outlined in Fig 15. Starting with lactam **17** [55, 56], the N-alkylation was carried out following a report by Nelissen *et al*. [57]. Deprotection, amide coupling, carbamate cleavage and preparation of hydrochlorides were performed as described previously for series III.

**Fig 16:**
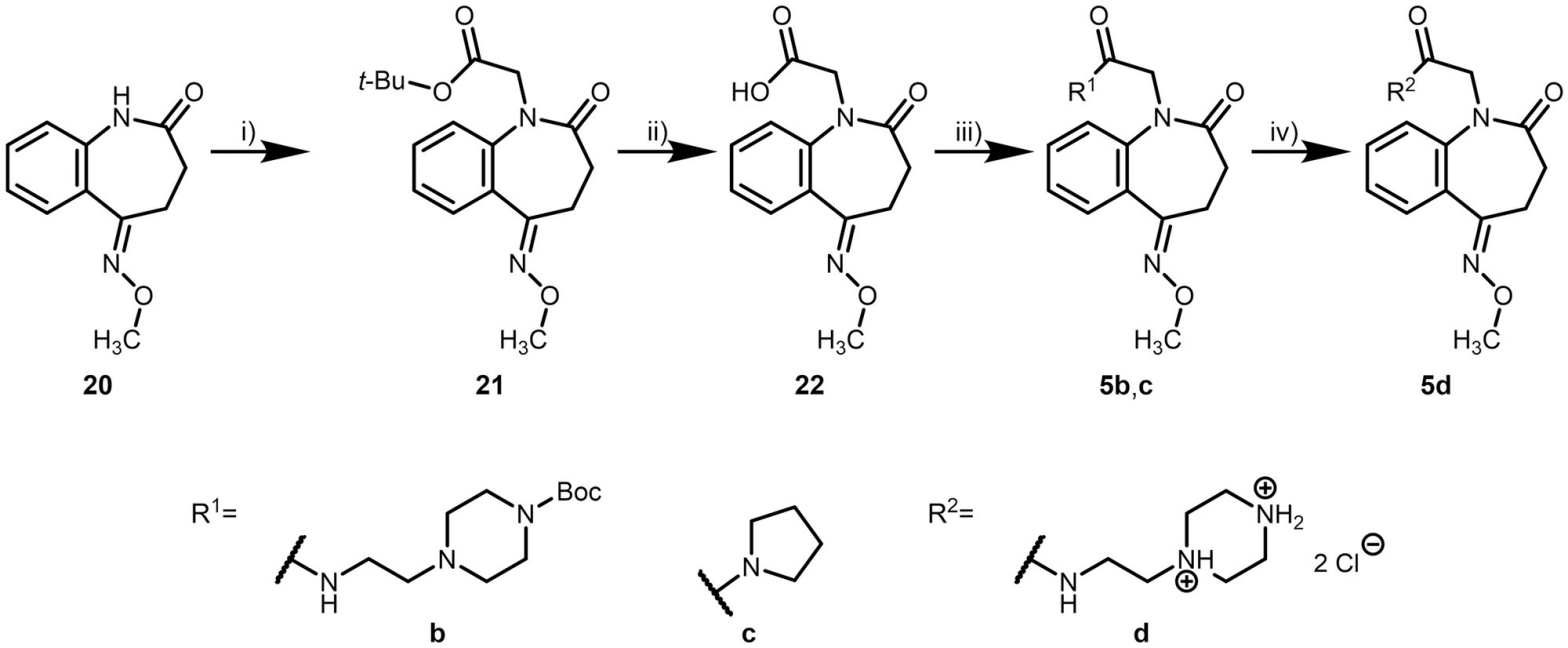
Syntheses of methoxyiminobenzo[*b*]azepinylacetamides (series V). Reagents, conditions and yields: i) 1. THF, *tert*-BuOK, RT, N_2_, 60–75 min; 2. *tert*-butyl bromoacetate, 7–95 h, 52–73%; vi) CH_2_Cl_2_, TFA, RT, N_2_, 8.5 h, 91%; vii) DMF, DIPEA, EDCl, amine, RT, argon, 21–87.5 h, 29–31%; viii) 1. CH_2_Cl_2_, TFA, RT, argon, 22 h; 2. propan-2-ol, HCl, *n*-hexane, RT, 18 h, 20%.

The synthesis of the oxime ether derivatives constituting series V started with oxime ether **20** [58], which was N-alkylated to yield the *tert*-butyl ester **21**. Deprotection of **21** furnished the carboxylic acid **22**, which was further converted to amides **5b**–**d** (Fig 16).

### Biological results

All novel compounds were tested in three primary biological test systems in one-dose experiments. Enzyme assays were performed on the trypanothione synthetase of *L*. *infantum* (*Li*TryS) and *T*. *b*. *brucei* (*Tb*TryS). The antiparasitic activity was assessed on a bloodstream (BS) form of *T*. *b*. *brucei* and for selected compounds also on the intracellular stage (called amastigote) of *L*. *infantum*. To evaluate toxicity to mammalian cells, mouse macrophages (J774.A1 cell line) were exposed to the compounds. IC_50_ or EC_50_ values were determined when respective one-dose assays revealed ≥50% inhibition of TryS activity or ≤50% *T*. *b*. *brucei* proliferation.

**Fig 17:**
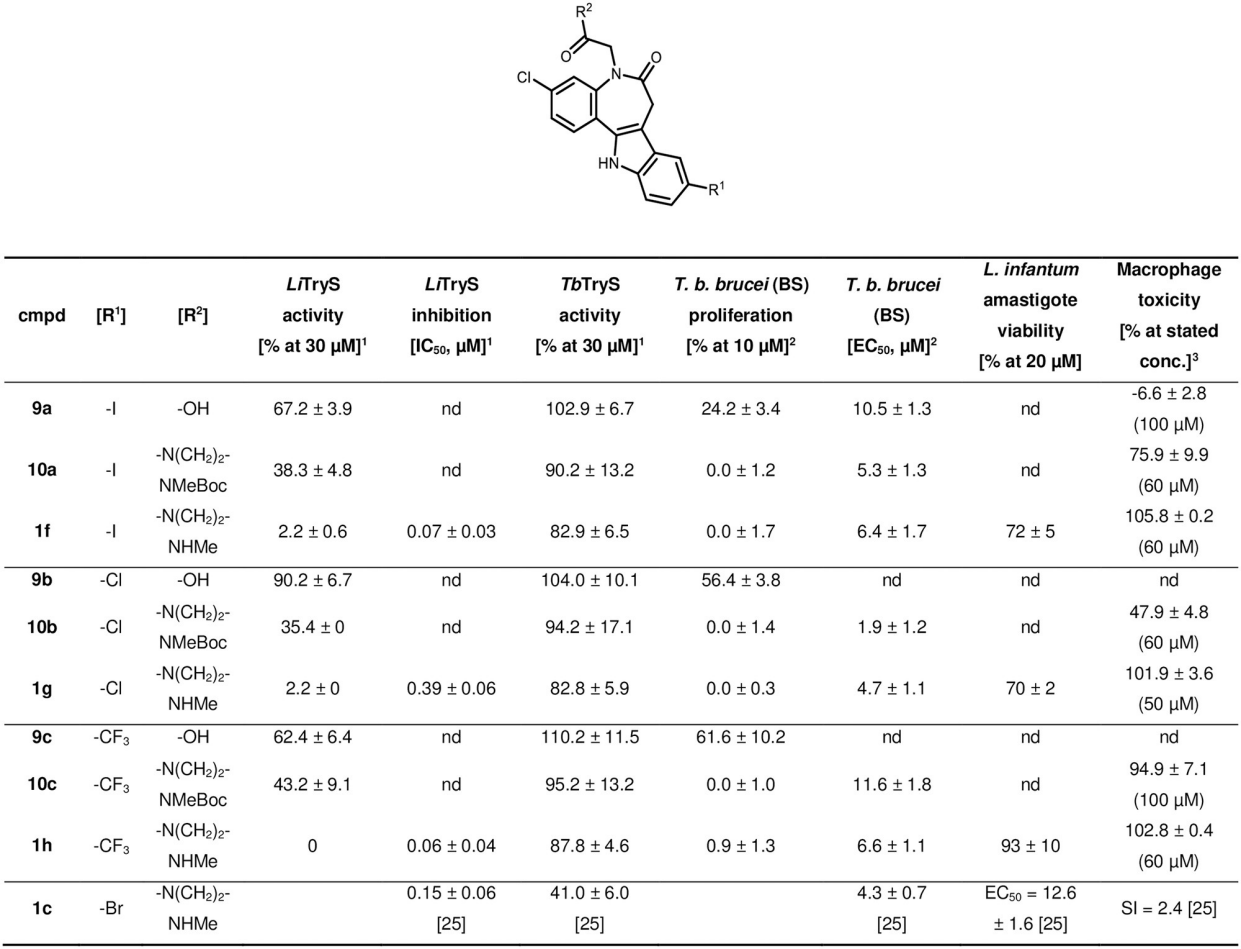
Biological activities of series I compounds on various biological systems. ^1^Enzyme assay on recombinant TryS (% activity at 30 μM compound concentration, or IC_50_ in μM); ^2^Whole parasite assay on BS *T*. *b*. *brucei* (% proliferation at 10 μM compound concentration, or EC_50_ in μM); ^3^Cytotoxicity assay on murine macrophages J774.A1 (% cell death at stated compound concentration). Not determined values = nd. Enzymatic activity errors are displayed as 2 standard deviations (SD), cytotoxicity as 1 SD. MOL2008 (1c) results obtained from Benítez *et al*. [25] and displayed for comparison.

Some of the paullone derivatives of series I exhibited noteworthy activity in the biological test systems (Fig 17). Enzyme assays on *Li*TryS proved moderate inhibitory activity of the carboxylic acids **9a** and **9c** and the carbamates **10a**–**c** at 30 μM concentration. The amines **1f**–**h** exhibited submicromolar activity comparable to the reference MOL2008 (**1c**). Among the amine derivatives, including MOL2008, one can observe an increasing inhibitory activity in the order **1g** < MOL2008 < **1f** ≤ **1h**, consistent with an enhanced potency in the order Cl < Br < I ≤ CF_3_ for the 9-substituent. However, the compounds displayed no activity on *Tb*TryS.

*T*. *b*. *brucei* proliferation is impacted by both the carbamates **10a**–**c** and amines **1f**–**h**, while the carboxylic acids **9a**–**c** only have moderate inhibitory activity at 10 μM. Likely, the negative charge of the acidic compounds **9a**–**c** contributes to their low bioactivity by precluding them from freely crossing the cell membranes. The carbamates and amines both have an activity in the single-digit micromolar range except for **10c**, with an activity of approx. 12 μM. While the inhibitory activities of the carbamates rank **10c** < **10a** < **10b** (substituents: CF_3_ < I < Cl), the corresponding amines perform in the order **1h** = **1f** ≤ MOL2008 = **1g** (substituents: CF_3_ = I ≤ Br = Cl).

The compounds **10a**–**c** and **1f**–**h** display no *Tb*TryS inhibition, but impair *T*. *b*. *brucei* proliferation, which points to a mechanism involving a different target. Possible biotransformation of the Boc-protected compounds could liberate the amines, like an enzymatic cleavage of the carbamates [59]. Contrarily, the amines **1f**–**h** with submicromolar activity on *Li*TryS show limited impairment of *L*. *infantum* amastigotes viability at 20 μM compound concentration. *Leishmania* amastigotes reside and multiply inside macrophages. Thus, the divergent activity of the amines **1f**–**h** on *Li*TryS versus the amastigotes might be explained by the difficulty of the compounds to cross, at least, three cell membranes: the macrophage outer membrane, the parasitophorous vacuole membrane (PVM) and the parasitic membrane. Apart from substance inactivity due to insufficient membrane penetration, there are diverse other reasons as active transport out of the cells or degradation and metabolism within the host cells or amastigotes.

To be considered as a ‘hit’, bioactive compounds should ideally have a 10-fold selectivity in their cytotoxicity towards the pathogen *vs*. host cells [60]. With this concept in mind, active compounds were tested against macrophages at concentrations ≥10-fold than their EC_50_ for *T*. *b*. *brucei*. Under these conditions, compounds that show biological activity on the parasites also tend to be toxic against murine macrophages. While the carboxylic acid derivative **9a** does not impair macrophage viability at 100 μM, the respective amines significantly reduced the viability of the host cells at concentrations ranging from 50 to 100 μM, indicating a low selectivity of these compounds between host and parasite cells.

**Fig 18:**
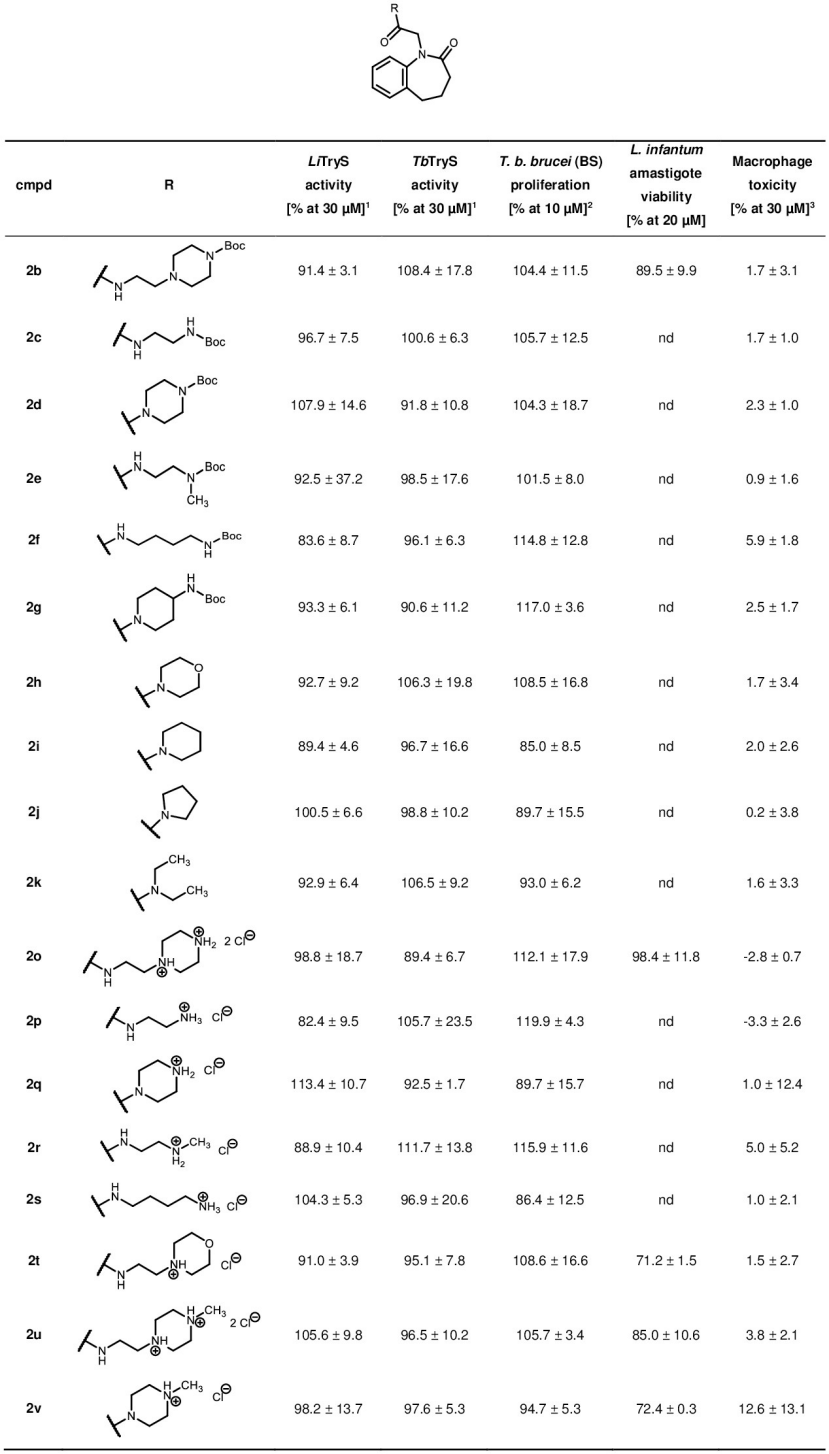
Biological activities of series II compounds on various biological systems. ^1^Enzyme assay on recombinant TryS (% activity at 30 μM compound concentration); ^2^Whole parasite assay on BS *T*. *b*. *brucei* (% proliferation at 10 μM compound concentration); ^3^Cytotoxicity assay on murine macrophages J774.A1 (% cell death at 30 μM compound concentration). Not determined values = nd. Enzymatic activity errors are displayed as 2 standard deviations (SD), cytotoxicity as 1 SD.

While rich in diversity regarding the alkyl side chain, molecules belonging to series II only have an undecorated benzazepinone scaffold. These compounds showed no inhibition on *Li*TryS or *Tb*TryS and failed to inhibit the growth of the *T*. *b*. *brucei* parasites and *L*. *infantum* amastigotes. Yet, the compounds also proved to be non-cytotoxic for the tested macrophages (Fig 18).

**Fig 19:**
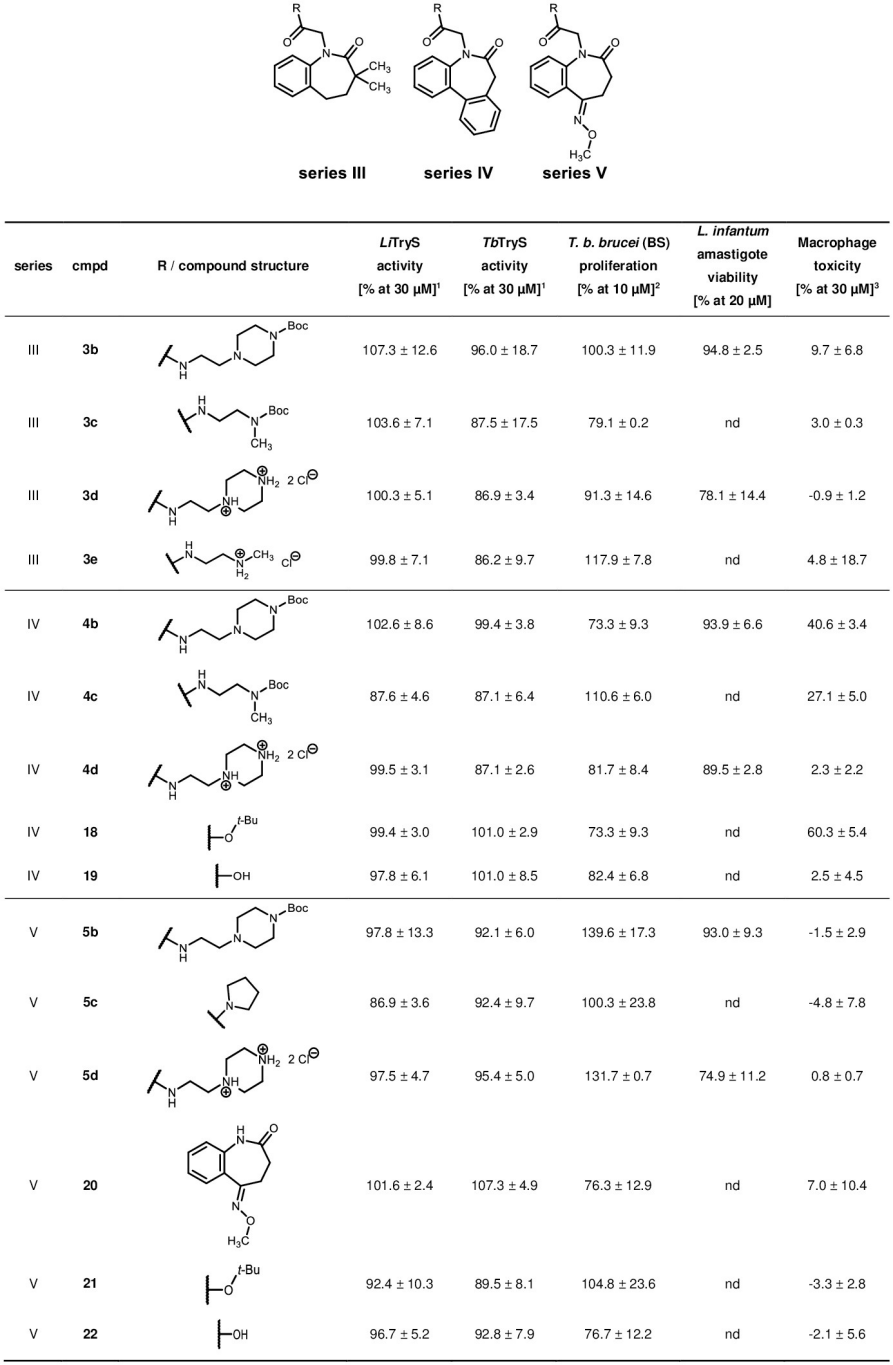
Biological activities of series III–V compounds on various biological systems. ^1^Enzyme assay on recombinant TryS (% activity at 30 μM compound concentration); ^2^Whole parasite assay on BS *T*. *b*. *brucei* (% proliferation at 10 μM compound concentration); ^3^Cytotoxicity assay on murine macrophages J774.A1 (% cell death at 30 μM compound concentration). Not determined values = nd. Enzymatic activity errors are displayed as 2 standard deviations (SD), cytotoxicity as 1 SD.

Compounds of series III impair neither *Li*TryS nor *Tb*TryS activity nor proliferation of *T*. *b*. *brucei* parasites or viability of *L*. *infantum* amastigotes. Alike, series IV contains no active compounds against *Li*TryS or *Tb*TryS. *T*. *b*. *brucei* and *L*. *infantum* parasite growth is barely impaired. Except for compounds **4b** and **18** (40% and 60% cytotoxicity), all compounds from series III–V displayed low cytotoxicity (0–27%) against macrophages when tested at 30 μM. The oxime ether derivatives of series V do not significantly affect the tested biological systems, including the mammalian cells (Fig 19).

### Solubility and physicochemical properties

The solubility of some benzyl-substituted paullones was previously characterised, including investigations of solubility improvement by drug formulation [27, 61]. The paullones’ thermodynamic solubility was found to be submicromolar, making them less druglike (logS = -5.30). The desired value for the solubility is dependent on other factors, such as the drug’s potency or permeability, ultimately determining whether the drug is fully absorbed. For oral absorption of a compound with medium intestinal permeability, a minimum thermodynamic aqueous solubility of roughly 0.05 mg/mL (0.1 mmol/L for a 500 g/mol compound, logS = -4) is needed, about 0.2 mg/mL for poorly permeable compounds, with up to 1 mg/mL (logS = -2.70) for a safety margin [62, 63]. Other sources set the target values for good solubility in a similar range with >200 μM / logS = -3.70 (Hill and Young [64]) or >60 μg/mL / 120 μM / logS = -3.92 (Kerns and Di [65]). However, the bar is lower for NTD lead candidates with ‘acceptable physicochemical properties’ and a solubility >10 μM / logS > -5 in phosphate-buffered saline [35].

**Fig 20:**
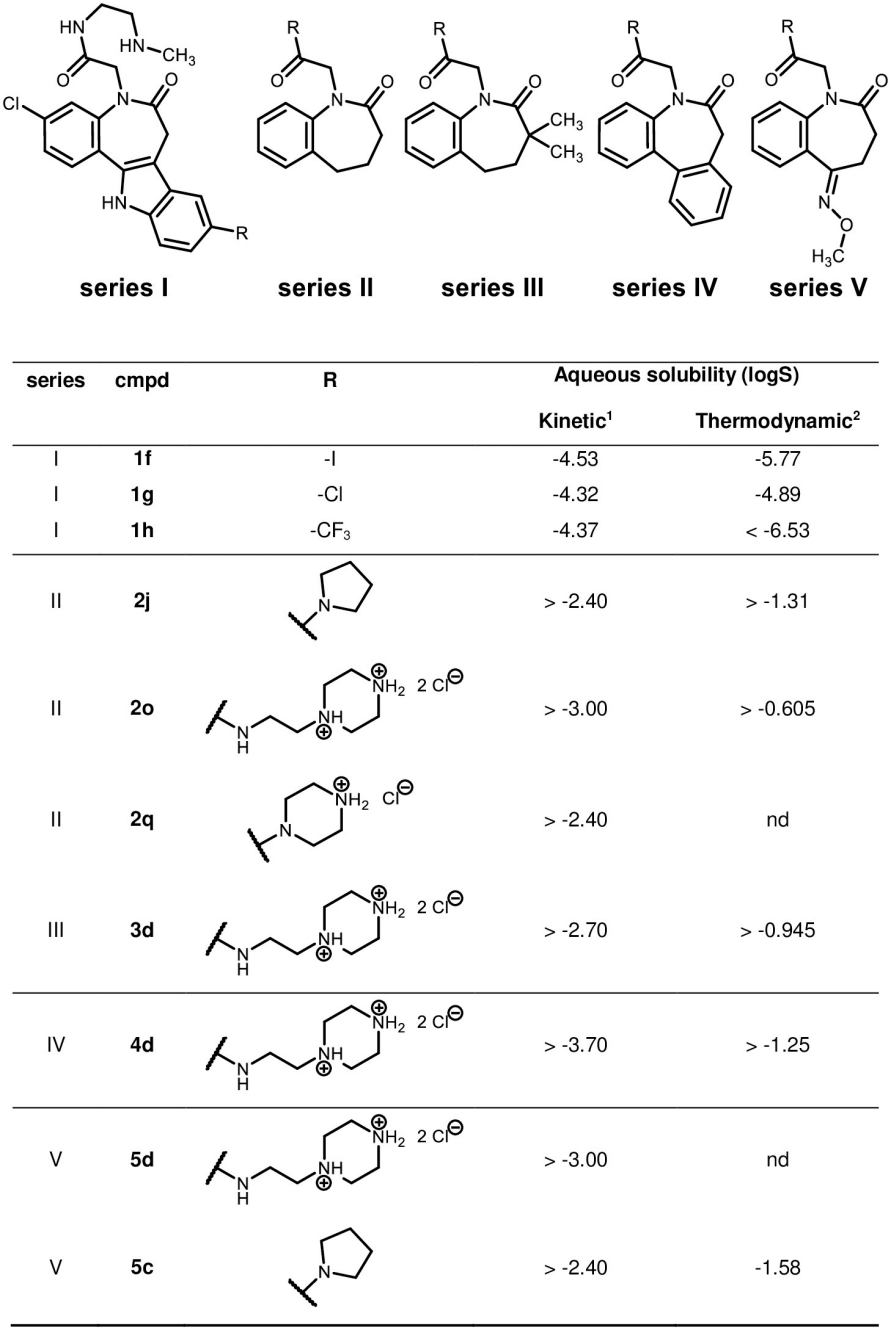
Aqueous solubility of selected compounds. ^1^Kinetic solubility measured by nephelometry; ^2^Thermodynamic solubility determined by a shake-flask method and HPLC-UV measurement; Not determined values = nd.

The solubility presented by aminoalkyl-substituted paullones is double-digit micromolar when kinetically determined, whereas the thermodynamically generated results range down to submicromolar concentrations (**1h**). Thus, the kinetically determined solubilities of series I compounds are ‘moderate’, but their thermodynamically determined solubilities are ‘low’ in some cases (10–60 μg/mL / 20–120 μM / logS -4.70 to -3,92 (Kerns and Di [65]). The paullones (series I) are more lipophilic than series II–V compounds, which is also reflected in their lower aqueous solubility. The tested paullones meet the requirements of compounds in NTD drug discovery in the kinetic solubility assay (>10 μM, [35]). However, the thermodynamic measurement results only meet these criteria for **1g**. Compounds **1f** and **1h** demonstrate a lower solubility, which could cause problems in assays with longer incubation times through slow precipitation in the medium during the conversion to the thermodynamically more stable, yet less soluble form. In contrast, the measured series II–V compounds display single-digit millimolar or higher solubilities and meet the requirements for good aqueous solubility of a drug candidate (Fig 20).

## Conclusion

Aiming to explore structure-activity relationships of antitrypanosomal and antileishmanial benzazepinones and to generate novel TryS inhibitors, we designed, synthesised and tested various paullone and benzazepinone derivatives.

The biological testing results vary vastly between the presented series. Whereas the closely related analogues of MOL2008 (series I) showed both *Li*TryS inhibition and antiparasitic activity on *T*. *b*. *brucei* bloodstream forms, the simplified derivatives of series II–V displayed negligible biological activity in all test systems. The significant structural difference between active and inactive series is the presence or absence of the indole building block. Deleting or replacing this structural element leads to complete activity loss in the shown examples. On the other hand, the halogen variation of the paullone 9-substituent (**1f**–**h**) leads to retained biological activities compared to the parent compound MOL2008 (**1c**) and less than one order of magnitude activity variation among the three derivatives. Modifications of the methylamino ethyl side chain disturb *Li*TryS inhibition; however, the *N*-Boc substituted derivatives (**10a**–**c**) retain their antitrypanosomal activity. The problematic aspect of the compounds **10a**–**c** and **1f**–**h** is the lack of selectivity towards the tested mammalian cell line.

Similarly to MOL2008, the presented docking studies point towards an Spd/Gsp competitive mechanism for TryS inhibition which was shown in enzyme assays for MOL2008 [26]. The presented docking poses can further underpin this mechanism by showing that the paullones of series I interact with crucial structural components, which are essential for substrate orientation towards the active site. Despite a similar binding mode of series II–V compounds to the respective paullone derivatives, these substances do not impair enzyme activity. The paullone indole moiety seems to provide the necessary voluminous hydrophobic core that occupies the second GSH site (S4) and by doing so, competes very efficiently with Gsp binding. However, as pointed earlier [26], the paullone scaffold without substituents in N^5^ position is unable to affect *Li*TryS activity, even at concentrations 500-fold higher than the IC_50_ of the most potent derivatives reported here (**1f** and **1h**). Further investigations like ensemble docking of TryS-inhibiting paullones to ATP-/GSH-enzyme complexes would be of interest to elucidate a more realistic binding mode.

Regarding solubility, series I compounds perform moderately, fulfilling the NTDs drug discovery criteria when solubility was determined kinetically. The series II–V compounds’ solubilities are about two orders of magnitude higher than series I compounds, making them ‘good’ water-soluble compounds according to the above-presented definitions for drug candidates. Yet, by indole replacement, these compounds display no biological activity to speak of. They are physicochemically drug-like but not like a drug in terms of activity.

New drugs are still needed for diseases classified as ‘neglected’, like American trypanosomiasis, African trypanosomiasis and leishmaniasis. Antitrypanosomal paullone derivatives such as MOL2008 or amines **1f**–**h** mark a starting point for lead candidate development. However, presented series II–V lacking the indole moiety appear as a dead end. A successful strategy of different paullone modifications with structures retaining the indole was presented in a parallel project [66].

## Materials and methods

### Chemistry

#### Materials

Commercially available reagents were used without further purification unless otherwise stated. Starting materials and reagents were purchased from abcr (Karlsruhe, Germany), Acros Organics (Geel, Belgium), Alfa Aesar (Kandel, Germany), Enamine (Kyiv, Ukraine), fluorochem/Chempur (Derbyshire, UK), Sigma-Aldrich/Merck (Steinheim, Germany) or TCI (Tokyo, Japan). *N*,*N*-dimethylformamide was purchased in Uvasol^®^ quality from Sigma-Aldrich (Steinheim, Germany). Dichloromethane, tetrahydrofuran and toluene were dried according to published procedures [67]. Water was purified by demineralisation in-house. HPLC solvents and reagents were of analytical grade and purchased from Sigma-Aldrich (Steinheim, Germany) or Acros Organics (Geel, Belgium); doubly distilled water was used. Deuterated solvents for NMR spectroscopy were purchased from Deutero GmbH (Kastellaun, Germany). Silica gel 60 Å was used for purification by column chromatography.

#### Instrumentation

Reaction monitoring by thin layer chromatography (TLC) on TLC plates (Macherey-Nagel, Düren, Germany), Polygram SIL G/UV254 on polyester film (40x80 mm) with the indicated elution system (mixtures of dichloromethane, acetic acid, ethanol, ethyl acetate, petroleum ether, toluene or triethylamine of various ratios); The bands were analysed under UV light (254 and 366 nm) and, if applicable, *via* a TLC-MS coupled system. Melting points (m.p.) were measured in open-glass capillaries on an electric variable heater (Electrothermal IA 9200, Cole-Parmer, Staffordshire, UK; ramp 1°C/min). Decomposition is pointed out (decomp.). Infrared spectra (IR) were recorded from a KBr pellet on an FTIR spectrometer (Nicolet FT-IR 200, Thermo Fisher Scientific, Waltham, MA, USA; 1/*λ* in cm^−1^; range 4000–400 cm^−1^). ^1^H-NMR and ^13^C-NMR spectra were recorded at the NMR laboratories of the Chemical Institutes at TU Braunschweig, Germany. The spectrometers used were either Bruker Avance IIIHD 300N (BBO probe), Bruker Avance III 400 (BBO probe), Bruker Avance III HD 500 (BBO cryo-probe) or Bruker Avance II 600 (BBI cryo-probe or BBO probe) (Bruker Biospin, Rheinstetten, Germany). Samples were measured at room temperature as a solution in DMSO-*d*_*6*_ or CDCl_3_ (concentration range approx. 2–20 mg/mL, samples were dissolved in the specified solvent, if necessary, in the ultrasonic bath, then filtered). No signal suppression was applied. Chemical shifts are expressed relative to the internal standard TMS (δ 0 ppm), *J* in Hz. Signals, splitting patterns and integrals were assigned with the software MestReNova (Mestrelab Research, Santiago de Compostela, Spain). In the software, auto peak picking, auto multiplet analysis and auto integration were applied. Evaluation of the automatic assignment was done by comparison with predicted spectra under the same conditions as the acquired spectrum (spectrometer frequency, solvent, number of points, line width, spectral width). Multiplets were evaluated by overlaying the pattern of simulated multipletts with the same coupling constants as for the measured signals. AX and AB systems were identified manually and evaluated as follows: coupling constants were calculated with the software auto multiplet analysis and chemical shifts were calculated by the formula *v*_*a or b*_ = *v*_*center*_ + 0,5 * Δ*v*_*a or b*_ Allocation of observed signals to the respective groups in the molecule were assigned manually based on literature. If an integral is not reported, the assignment was difficult due to overlapping signals. Broad (br) and weak (w) signals are outlined. ^13^C-NMR spectra were recorded by application of proton decoupling by ‘composite pulse decoupling’ (cpd). ^13^C signals were assigned based on results of ^13^C-DEPT135 experiments. Other experiments as indicated in the compounds’ details. HPLC was performed on following devices with settings as indicated: Merck Hitachi LaChrom Elite (Hitachi High Technologies Corporation, Tokyo, Japan) with hardware consisting of the autosampler L-2200, the diode array detector L-2450, the pump L-2130, the column oven L-2300 (40°C); the column holder LiChroCART 125–4; column LiChrospher 100 RP-18 (5 μm) (Merck, Darmstadt, Deutschland); software: EZChrom Elite 3.3.2 SP2 (Agilent Technologies, Santa Clara, CA, USA); the threshold of the integration method was set to 1000. VWR Hitachi Chromaster system (Hitachi High Technologies Corporation, Tokyo, Japan) with hardware consisting of the diode array detector 5430, the column oven 5310, the pump 5110, the autosampler 5260, the column Merck LiChroCART 125–4, LiChrospher 100 RP-18 (5 μm) (Merck, Darmstadt, Germany); the threshold of the integration method was set to 50. Isocratic eluent: acetonitrile/water mixtures or acetonitrile/aqueous buffer mixtures; The aqueous triethylamine/triethylammonium sulfate buffer (pH 2.7) was prepared by dissolving triethylamine (20 mL) and sodium hydroxide (242 mg) in bidestilled water (to 1 L). pH was adjusted to 2.7 with conc. H_2_SO_4_. Gradient elution: acetonitrile/water mixtures with the following acetonitrile concentration at retention times 0–2 min: 10%; 2–12 min: 10% → 90% (linear); 12–20 min: 90%; elution rate: 1.000 mL/min; detection wavelength: 254 nm and 280 nm (isocratic and gradient); overall run time: 15 min (isocratic), 20 min (gradient); t_M_ = dead time related to DMSO; t_M+S_ = total retention time. AUC was assessed, 100% method; Absorption maxima (λ_max_) were extracted from the UV spectra recorded by the DAD detector. For mass spectrometry, an expression^L^ CMS spectrometer was used with an ESI or APCI source coupled to an ASAP (atmospheric solids analysis probe), TLC-MS Interface Plate express or direct injection of a methanolic solution (approx. 50 μg/1.5 mL, injection volume: approx. 100–150 μL) (DI) for sample inlet (Advion, Ltd., Harlow, UK). Measurements were performed simultaneously in the negative (−) and positive (+) ionisation modes. On-site generated nitrogen gas was used for nebulisation. Characteristic ions, adducts and fragments are subject to interpretation. Electron spray ionisation (ESI): source gas temperature: 200°C (pos. mode), 250°C (neg. mode), capillary temperature: 250°C, capillary voltage: 180 V, spray voltage: 3500 V. Atmospheric pressure chemical ionisation (APCI): Source gas temperature: 250°C (pos. mode), 350°C (neg. mode), APCI corona discharge: 5.0 μA, capillary temperature: 250°C, capillary voltage: 180 V. APCI soft: Source gas temperature: 250°C (pos. mode), 350°C (neg. mode), APCI corona discharge: 5.0 μA, capillary temperature: 250°C, capillary voltage: 150 V. Software: Advion Mass Express and Advion Data Express (version 6.2.21). Elemental analysis was performed on a CE Instruments Flash EA 1112 Elemental Analyzer (Thermo Quest, San Jose, CA, USA). Theoretical values were calculated using *ChemDraw* 22.0.0 software (PerkinElmer Informatics, Waltham, MA, USA). If the elemental analyses were inconclusive, HRMS was performed. Electron spray ionisation (ESI) HRMS spectra were recorded on LTQ-Orbitrap Velos (ThermoFisher Scientific, Bremen, Germany) at the MS laboratories of the Chemical Institutes at TU Braunschweig, Germany. Tetradecyltrimethylammonium bromide (0.1 mg/mL) was used as an internal standard. Compounds were dissolved in methanol to obtain a concentration of approximately 50 μg/mL. Flow rate: 1 μL/min, spray voltage: pos. mode: 2.3–2.8 kV, neg. mode: 1.7–2.5 kV. Software: Xcalibur 2.2 (Thermo Fisher Scientific, Waltham, MA, USA).

### Biological and biochemical investigations

#### Expression and purification of recombinant TryS

TryS from different trypanosomatids was produced in recombinant form with an N-terminal His-tag. The constructs pET-15b *Tb*TryS and pET-28c(+)*Li*TryS were used to express TryS of *T*. *b*. *brucei* 427 (MITat1.4, GenBank accession protein id CAC87573.1), and *L*. *infantum* JPCM5 (GenBank accession protein id CAM69145.1). *E*. *coli* strain BL21 (DE3) served as the expression host. For *Tb*TryS, the cells were cultured in a medium based in Terrific Broth and induction with isopropyl 1-thio-β-D-galactopyranoside (IPTG), while for *Li*TryS, the cells were cultured in ZYM-5052 auto-induction medium. For a detailed description of the expression, see Benítez *et al*. [25]. The purification was performed by immobilised metal affinity chromatography in a HisTrap Fast Flow column (GE Healthcare) followed by size exclusion chromatography. Elution fractions from the metal-affinity chromatography (~60 mg for *Tb*TryS and *Li*TryS) were loaded onto a HiLoad^®^ 16/60 Superdex^®^200 pg column (GE Healthcare, Piscataway, NJ) pre-equilibrated with 100 mM HEPES pH 7.4, 10 mM MgSO_4_, 0.5 mM EDTA, 1 mM DTT and 150 mM NaCl, at room temperature (20–25°C) using an Akta-FPLC device (GE Healthcare, Piscataway, NJ) and a flow rate of 0.75 mL/min [26]. The fractions containing recombinant TryS with ≥95% purity were pooled and concentrated to >10 mg protein/mL using a 10 kDa cut-off Amicon filter (Millipore, Billerica, CA). Glycerol was added at a final concentration of 40% (v/v). The samples were stored at -80°C. Enzyme purity and activity were assessed by SDS-PAGE (12%) under reducing conditions and by the end-point TryS assay (see TryS activity screening assay), respectively. Protein concentration was determined using the bicinchoninic acid assay with bovine serum albumin as standard. The protocols yielded 25–30 mg *Tb*TryS or *Li*TryS per litre of culture medium and homogeneous specific activity.

#### TryS activity screening assay

For all TryS, ATP was used at 150 μM because of a high background signal at higher concentrations and Spd fixed at 2 mM, in agreement with the intracellular levels calculated from data reported in the literature for trypanosomatids. GSH was adjusted to 0.05 and 0.25 mM for *Tb*TryS and *Li*TryS, respectively, to avoid substrate inhibition or to approach physiological concentrations [25]. A master mix (MM) solution, containing all the substrates at 1.25-fold their end concentration in the assay, was prepared in screening reaction buffer (5 mM DTT, 10 mM MgSO_4_, 0.5 mM EDTA, 100 mM HEPES pH 7.4, 9 mM NaCl, 10% v/v DMSO) and kept on ice until use. Microtiter plate wells were loaded with 5 μL of the test compound, DMSO (reaction control) or a TryS specific inhibitor (inhibition control) and 40 μL of MM solution. The reactions were then started by adding 5 μL of TryS (2 and 3_*_10^−5^ μmol_*_min^-1^_*_mL^-1^ for *Li*TryS and *Tb*TryS, respectively) and stopped after 15 min with 200 μL BIOMOL^®^ GREEN reagent (Enzo Life Sciences). The compounds used as inhibition control were added at concentrations that inhibited 50% TryS activity: 30 μM and 150 nM MOL2008 for *Tb*TryS and *Li*TryS, respectively. Blanks were prepared for each condition by adding 5 μL of screening reaction buffer instead of the enzyme. The TryS activity is calculated as follows:

$$TryS\;activity\;\left(\%\right)=\frac{A_{650nm\;Ci}-A_{650nm\;CiB}}{A_{650nm\;E}-A_{650nm\;EB}}*100\%$$

A_650 nm Ci_ is the mean absorbance at 650 nm for the reaction test with compound I (Ci), A_650 nm CiB_ is the mean absorbance at 650 nm for the blank control with compound I (CiB), A_650 nm E_ is the mean absorbance at 650 nm for the reaction control with DMSO (E) and A_650 nm EB_ is the mean absorbance at 650 nm for the blank control with DMSO (EB). The errors were expressed as 2 standard deviations (SD), SD estimated as σ^n−1^.

#### Cell culture and cytotoxicity assays

*Viability assay for T*. *b*. *brucei*. The bloodstream form of the monomorphic *T*. *b*. *brucei* strain 427, cell line 5141313, constitutively and stably expressing an ectopic copy of the *Photinus pyralis* red-shifted luciferase [68], was cultured *in vitro* with HMI-9 medium [69], supplemented with 10% fetal bovine serum, 10 U/mL penicillin (Gibco), 10 μg/mL streptomycin (Gibco), 0.2 μg/mL bleomycin (Jena Bioscience), 4 μg/mL G418 disulfate salt (Sigma-Aldrich) and 0.2 μg/mL puromycin (Invitrogen), inside a humidified incubator (Thermo Fisher Scientific) with controlled temperature (37°C) and CO_2_ (5%) in a 25 or 75 cm^2^ vented-cap culture-flask. The anti-trypanosomal activity of compounds was evaluated in a 96-well culture plate containing 2.2 μL/well DMSO (negative control) or compounds dissolved in DMSO (1% final concentration), and 220 μL/well of a suspension of 1 × 10^5^ parasites/mL. The plates were incubated at 37°C and 5% CO_2_ for 24 h. Next, each well was transferred to a 96-well black plate, and 20 μL of a solution containing d-luciferin (1.5 mg/mL in PBS glucose 1% *w*/*v*) and Triton X-100 (0.05% v/v) was added. Bioluminescence signal was measured in a LUMIstar OPTIMA Microplate luminometer using the following settings: 10 s shaking, 5 s/well acquisition, 0.2 s measurement delay, maximum gain, and 37°C. For the bioluminescence assay, parasite viability was calculated according to the following formula:

$$parasite\;viability\;\left(\%\right)=\frac{{BL}_{cpd}-{BL}_{blank}}{{BL}_{neg}-{BL}_{blank}}*100\%$$

BL refers to the mean of bioluminescence signal corresponding to the tested compound (cpd), the blank (*blank*, complete media containing 1% *v*/*v* DMSO), or the negative control (*neg*, parasites treated with 1% *v*/*v* DMSO). EC_50_ values were determined from concentration-response curves fitted to a four-parameter sigmoid equation using the GraphPad Prism software (version 6.0). All errors are expressed as one SD, estimated as σ^n-1^. For a detailed explanation of the protocol, see Dibello *et al*. [70].

*Cytotoxicity assays on murine macrophages*. Murine macrophages from the cell line J774.A1 (ATCC^®^ TIB-67TM) were cultivated under a humidified 5% CO_2_/95% air atmosphere at 37°C in Dulbecco’s modified Eagle’s medium (DMEM) supplemented with 10% (*v*/*v*) FBS, 10 U/mL penicillin and 10 μg/mL streptomycin. The experimental protocol for the determination of EC_50_ values was identical as described by Demoro *et al*. 2012 [71], with the following change: 200 μL/well of a cell suspension at 6 × 10^4^ cells per mL was added in a 96-well culture plate and washed with 150 μL of DMEM. The cytotoxicity of the compounds was evaluated in triplicate using the WST-1 reagent (Roche). The control treatment included cells cultured in the presence of DMSO 1% (*v*/*v*). Absorbance at 450 nm (A_i 450 nm_), corresponding to the formazan dye produced by metabolically active cells, was measured with an EL 800-microplate reader. The corrected absorbance values at 450 nm (A_i c 450 nm_) were obtained by subtracting the corresponding absorbance value at 630 nm (A_i 630 nm_) and the blank average (A_blank 450 nm_):

$$A_{i\;c\;450nm}=A_{i\;450nm}-A_{i\;630nm}-A_{blank450\;nm}$$

Cell viability (%), with the number of viable cells proportional to the absorbance, was calculated as follows:

$$cell\;viability\;\left(\%\right)=\frac{A_{i\;c\;450nm}}{A_{negative\;control\;c\;450nm}}*100\%$$

A_i c 450 nm_ refers to an absorbance obtained from cells treated with compound I at a specific concentration (*i*) and A_negative control c 450 nm_ refers to an absorbance obtained from cells in the DMSO-treated control (*negative control*). The errors were calculated using error propagations and are expressed as one SD, estimated as σ^n−1^.

Viability assay for *L*. *infantum* amastigotes. *L*. *infantum* promastigotes from the reference strain MHOM/MA/67/ITMAP263, stably transfected with a vector encoding a red-shifted luciferase gene (RE9HLUC; Branchini *et al*. [72]; Benítez *et al*. [73]) were grown in fresh complete RPMI medium (RPMI basic medium containing 25 mM HEPES, 10% v/v iFBS, 100 U/mL penicillin, 100 μg/mL streptomycin and 15 μg/mL hygromycin B) at 28°C in sealed culture flasks. Metacyclic promastigotes were obtained by a negative selection procedure using peanut agglutinin and used to infect murine macrophages (cell line J774.A1) at a 1:10 ratio for macrophages (5×10^6^ cells): parasites (5×10^7^ cells). 200 μL of this suspension was added per well to a 96-well culture microplate, which was then incubated at 37°C for 24 h in a 5% CO_2_/95% air atmosphere incubator. The culture supernatant was removed and the cell monolayer was carefully washed with pre-warmed PBS (37°C) prior to adding the compounds (20 μM or lower if cytotoxic) and the reference drug amphotericin B at 0.1 M (approx. EC_90_), or the vehicle (0.5% DMSO) all prepared in fresh complete DMEM medium (DMEM basic medium, containing 10% v/v iFBS, 100 U/mL penicillin, 100 μg/mL streptomycin). The culture microplate was incubated in the CO_2_ incubator for additional 24 h. Next, the supernatant was removed and the microplate wells were washed with pre-warmed PBS (200 μL). Luciferase activity was revealed using the Luciferase Assay System E4530 (Promega) and measured with a LUMIstar OPTIMA microplate luminometer. All conditions were tested in triplicates and the percentage amastigote viability was calculated as described for infective *T*. *b*. *brucei*, with errors expressed as one SD, estimated as σ^n-1^.

A macrophage cell density correction factor based on the incorporation of propidium iodide was calculated and applied to normalise amastigote viability related to host cell density in the well. For a detailed explanation of the protocol, see Benítez *et al*. [73].

### Aqueous solubility determination

Phosphate-buffered saline pH 7.4 was prepared according to the European Pharmacopeia 10.4 by dissolving 2.38 g sodium monohydrogen phosphate dodecahydrate, 0.19 g potassium dihydrogen phosphate and 8.0 g sodium chloride in demineralised water (to a total volume of 1000 mL). The pH was adjusted to a value of 7.4 with hydrochloric acid. The buffer was filtered through a 0.45 μm membrane filter (Chromafil^®^ Xtra PES-45/25) before use.

#### Kinetic aqueous solubility

The kinetic aqueous solubility was determined by nephelometry. The measurements were performed on the *NEPHELOstar Plus*, with accompanying software Omega 5.11 and MARS 3.20 (*BMG LABTECH*, Ortenberg, Germany). Generally, 2 dilution series of 1–2 weighed portions of each substance were measured. First, stock solutions of the compounds were prepared in DMSO. A series of dilutions in DMSO were prepared from one stock solution to obtain at least 3 concentrations below and 3 concentrations above the precipitation point in the measured series. The specific concentrations were usually measured as duplicates.

The 96-well plate (Corning UV transparent flat-bottom 96-well plate, Corning Incorporated, Kennenbunk, ME, USA) was inspected for contamination and scratches and measured without samples prior to the solubility measurement of the compounds. 198 μL of phosphate-buffered saline pH 7.4 was placed in a 96 well plate for each well to be measured. 2 μL of a DMSO solution was added and immediately mixed by moving the pipette plunger. This solution either contained no substance—and served as a blank for the measurement (3 wells)—or the respective DMSO dilution of the substance (2–4 wells). The concentration of the solvent DMSO in each measured solution was 1%. The concentration of the compound corresponds to a 1:100 dilution compared to the respective DMSO dilution. The well plate bottom was cleaned with a lint-free cloth and compressed air and inspected for impurities before measurement. After shaking at 500 rpm for 10 s (double orbital), the measurement was carried out in the nephelometer with the laser intensity set to 80% and beam focus of 2.5 mm at a temperature of 25°C.

The obtained measuring points, relative nephelometric units (RNU) per compound concentration, were used to model two straight lines: one straight line with constant low readings and slope → 0 characterises the concentration range where no precipitation has taken place yet; The other straight line has a steep slope and increase of the RNU per concentration and characterises the concentrations at which precipitation takes place in the respective sample vessels.

The evaluation was carried out by calculating the two straight lines and their point of intersection. Outliers and values in a saturation range for the kick-off curve (usually >800 kRNU) were not used to create the straight lines and are listed separately in the evaluation file. If precipitation was not yet obtained at the highest measured concentration of the compound, a solubility of ‘greater than highest concentration’ was reported. The x-value of the intersection of the two straight lines represents the kinetic solubility. The results of two dilution series are additionally indicated as range (in brackets). For measurements of 3 or more series, a mean value with standard deviation is provided. The logS value describes the decadic logarithm of the molar solubility: logS (molar solubility [M]).

#### Thermodynamic aqueous solubility

The thermodynamic aqueous solubility was determined by a miniaturised shake-flask method. The compounds (with a final concentration more than twice (**2j** and **2o**) or more than five times the calculated highest solubility) were incubated in a Whatman Mini-Uniprep vial (GE Healthcare, Freiburg, Germany) with phosphate-buffered saline pH 7.4 (200–400 μL) in an incubation shaker (IKA KS 3000 ic control, Staufen, Germany) at 25°C, 400 rpm. A negative control with the buffer (400 μL) was incubated for every time point and measured likewise. After incubation for 24 h, 48 h and 72 h, the vials were checked for undissolved compound; then the filter plunger was punched into the vial for filtration. The concentration in the supernatant was determined by HPLC, if necessary, after a dilution in aqueous buffer pH 7.4 (Merck Hitachi LaChrom Elite system, three injections per time point, external calibration, AUC method, isocratic elution of ACN/aqueous buffer pH 2.7 mixtures, detection wavelength of 254 nm). The calibration curve was obtained from at least three dilutions and five measuring points from a DMSO stock solution of the respective compound. Calculated values were averaged from the last measured time point (72 h) and preceding time points when they did not deviate by more than 15% (as indicated in the OECD guidelines for the testing of chemicals, test No. 105 [74]).

### Molecular docking simulations

The following three-dimensional models were used for the target protein TryS: either homology models of *Li*TryS, based on a crystal structure of *Lm*TryS (*Lm*TryS pdb 2VPS) [27, 41] or the three-dimensional structure of *Li*TryS (A4I2Z3_LEIIN) generated by AlphaFold [43, 44]. The protein used was prepared in MOE 2022.02 [75] with the functions QuickPrep, Protonate 3D and Structure Preparation and saved as a mol2 file. The functions were used to add missing hydrogens to the structure, to protonate it according to a pH of 7 and to minimise the energy of the structure (force field: AMBER10:EHT). The ligands used were first drawn two-dimensionally with ChemDraw 22.0.0, loaded into MOE, energy-minimised with the QuickPrep function (force field: AMBER10:EHT), ionisable groups were protonated with Protonate 3D at a pH of 7 if necessary and saved as a mol2 file. The molecular docking was carried out in GOLD 5.2.2 [76] (GUI: Hermes 1.6.2). The wizard of the programme was used to load the protein, add protons and extract water and ligands if necessary. The binding pocket or interaction space at the target structure was defined by selecting a 10 Å radius around one of the following positions:

- When docking in the ATP-binding pocket of the homology models, the original ligand was extracted (chloropaullone-*N*^5^-acetamide), and the binding region was defined 10 Å around this area; if necessary, constraints were set for the investigations as protein hydrogen bond constraints. Specific protein atoms were defined to which the ligand had to form hydrogen bonds (Phe586 backbone CO and NH), but it was not specified which ligand atoms were to bind.
- When docking in the spermidine binding pocket, either a 10 Å radius around OE1 of the carboxyl group of Glu407 was chosen to define a region of the Spd/Gsp binding pocket close to the catalytic centre, or 10 Å radius around Phe626 backbone NH to define a region centred in the Spd/Gsp binding pocket. No constraints were set.

Chemscore_kinase was used as the template, and ChemPLP [77] (parameter file: DEFAULT) was selected as the scoring function. With a search efficiency of 200%, no early termination and the setting to generate diverse solutions, 10 genetic algorithm (GA) runs were performed. The exact parameters can be found in the ‘gold.conf’ text files of the corresponding docking runs. The evaluation of the results was carried out by comparing the resulting scores of the obtained docking poses, with the help of the ligand interactions function of MOE and by visualisation in UCSF ChimeraX [78, 79].

## Experimental

### Chemistry

#### General procedures

The synthesis of the following compounds was prepared according to published procedures: 8-chloro-3,4-dihydro-1*H*-benzo[*b*]azepin-2,5-dione (**6**) [50, 80], 4-iodophenylhydrazine [81], 1,3,4,5-tetrahydro-2*H*-benzo[*b*]azepin-2-one (**11**) [51], 3,3-dimethyl-1,3,4,5-tetrahydro-2*H*-benzo[*b*]azepin-2-one (**14**) [53, 82], 5,7-dihydro-6*H*-dibenzo[*b*,*d*]azepin-6-one (**17**) [55, 56] and (*E*)-5-methoxyimino-1,3,4,5-tetrahydro-2*H*-benzo[*b*]azepin-2-one (**20**) [58].

*General procedure 1*: *Fischer indole synthesis of paullones as described by Falke et al*. [83]. 8-Chloro-3,4-dihydro-1*H*-benzo[*b*]azepine-2,5-dione (1 eq) and the phenylhydrazine or phenylhydrazine hydrochloride (1.2 eq) are suspended in glacial acetic acid. If the phenylhydrazine hydrochloride is used, anhydrous sodium acetate (1.2 eq) is also added to the mixture. Then, the mixture is heated at 70°C for 75–90 min. Next, concentrated sulfuric acid is added to the mixture and stirred for further 1.25–2.5 h at 70°C. After cooling to room temperature, the mixture is poured onto a sodium acetate solution (5%), then stored overnight in the refrigerator (4°C). The resulting precipitate is vacuum filtered the next day and purified according to the indicated procedure.

*General procedure 2*: *selective benzazepine N-alkylation* [27]. The benzazepine derivative (1 eq) and potassium *tert*-butoxide (1.2–2.4 eq) are dissolved in tetrahydrofuran under a nitrogen atmosphere and stirred for 60–75 min at room temperature. Optionally, the mixture is cooled to about 0°C in an ice bath. The bromoalkane (1.1–2.2 eq) is added, optionally as a solution in THF. The mixture is stirred for another 2.5–31 h at room temperature. The solvent is removed under reduced pressure. After addition of water, the mixture is extracted with dichloromethane. The combined organic phases are distilled under reduced pressure until a solid remains. The residue is purified according to the indicated procedure.

*General procedure 3*: *tert-butyl deprotection* [27]. The *tert*-butyl protected compound (1 eq) is mixed with trifluoroacetic acid (40–43 eq) in dry dichloromethane and stirred at room temperature for the indicated reaction time under a protective gas atmosphere. The solvent is removed under vacuum, then the residue is purified according to the indicated procedure.

*General procedure 4*: *amide coupling as described by Dou et al*. [54]. The acetic acid derivative (1 eq), 1-hydroxybenzotriazole (HOBt, 0 or 1.2 eq) and 1-ethyl-3-(3-dimethylaminopropyl)carbodiimide hydrochloride (EDCl, 1.2–2.6 eq) are dissolved in *N*,*N*-dimethylformamide (DMF) in an argon-flushed reaction vessel. The solution is stirred for 30 min at room temperature and then diisopropylethylamine (DIPEA, 1.2–2.4 eq) and the amine to be coupled (1.1–3.6 eq) are added. After the required reaction time at room temperature, the mixture is purified according to the indicated method.

*General procedure 5*: *polar solvent removal from reaction mixtures as described by Hultin* [84]. The extraction series is carried out as follows: After addition of water, the reaction mixture is transferred to a separating funnel. Extraction is carried out with dichloromethane or indicated solvent, and the organic phase is transferred to a second separating funnel. A fresh portion of dichloromethane is added to the first separating funnel, a fresh portion of water is added to the second, and the mixtures are agitated. The process is repeated with the first organic phase being extracted multiple times with clean portions of water, then followed by as many organic phases as indicated in the procedure to be extracted in the same order with the same portions of water in a series of separating funnels. After passing through the last aqueous phase, the organic phases are combined and dried over sodium sulfate.

*General procedure 6*: *Boc deprotection* [27]. Trifluoroacetic acid (100–328 eq) is added to the Boc-protected compound (1 eq) in dry dichloromethane and stirred at room temperature for the indicated reaction time in an argon-flushed reaction vessel. After the removal of the solvent, an alkaline liquid-liquid extraction is carried out (target pH for the aqueous phase of 10–11), if indicated in the procedure. If applicable, the resulting solid is dissolved in 2-propanol and hydrogen chloride in 2-propanol (5–6 M) was added. Petroleum ether or *n*-hexane is slowly added to the solution while stirring at room temperature until a solid is formed that is further purified as indicated.

#### Compounds

*2-(3-Chloro-9-iodo-6-oxo-7*,*12-dihydrobenzo[2*,*3]azepino[4*,*5-b]indol-5(6H)-yl)-N-[2-(methylamino)ethyl]acetamide (1f)*. According to general procedure 6 from *tert*-butyl {2-[2-(3-chloro-9-iodo-6-oxo-7,12-dihydrobenzo[2,3]azepino[4,5-*b*]indol-5(6*H*)-yl)acetamido]ethyl}(methyl)carbamate (**10a**, 218 mg, 0.350 mmol) in trifluoroacetic acid (2.7 mL, 35 mmol) and dichloromethane (8 mL) (23 h). After removal of the solvent by distillation under reduced pressure, the residue was mixed with water (10 mL) and alkalised to pH 10–11 with sodium hydroxide solution (8% m/m) and diluted to an approximate volume of 100 mL, then extracted with ethyl acetate (100 mL). The aqueous phase was then extracted with dichloromethane (2x 100 mL) and next with toluene (100 mL). An insoluble residue was filtrated from the extractions, recrystallised from ethanol (37 mL) and ethyl acetate (14 mL) and dried (4 h, 60°C) to yield a colourless solid (29 mg, 55 μmol, 16%). Decomp.: starting at 245°C; IR (KBr): ṽ [cm^-1^] = [3427, 3317] (N-H), 1641 (C = O); ^1^H-NMR (DMSO-*d*_6_, 600 MHz): δ (ppm) = 2.28 (s, 3H, *N*-CH_3_), 2.54 (t, *J* = 6.3 Hz, 2H, *N*-CH_2_), 3.06 (br s, 1H, H_3_C-*N*H / azepine-CH_2_), 3.18 (q, *J* = 6.1 Hz, 2H, *N*-CH_2_), 3.94 (br s, 1H, *N*-CH_2_ / azepine-CH_2_), 4.14 (br s, 1H, *N*-CH_2_ / azepine-CH_2_), 4.35 (br s, 1H, *N*-CH_2_), 7.31 (dd, *J* = 8.5 / 0.6 Hz, 1H, ArH), 7.45 (dd, *J* = 8.5 / 1.7 Hz, 1H, ArH), 7.47 (dd, *J* = 8.4 / 2.1 Hz, 1H, ArH), 7.68 (d, *J* = 2.1 Hz, 1H, ArH), 7.71 (d, *J* = 8.4 Hz, 1H, ArH), 8.04 (t, *J* = 5.6 Hz, 1H, amide-NH), 8.11 (dd, *J* = 1.7 / 0.6 Hz, 1H, ArH), 11.96 (s, 1H, indole-NH); The signals between 3.06 ppm and 3.18 ppm overlap with each other and the HDO signal; The signals between 3.94 ppm and 4.35 ppm overlap; The amine NH signal is not assignable in the ^1^H experiment; ^13^C-NMR (DMSO-*d*_6_, 151 MHz): δ (ppm) = 35.5 (CH_3_), 31.1, 38.2, 50.4, 53.4 (w in DEPT135) (CH_2_), 114.0, 124.0, 125.0, 126.7, 128.48, 130.2 (CH), 82.8, 108.9, 123.8, 128.50, 132.3, 132.4, 136.3, 141.0, 168.1, 170.0 (C); The signal at 31.1 ppm is not assignable in the DEPT135 experiment (cf. **2q** for further experiments); C_21_H_20_ClIN_4_O_2_ (522.77); HRMS (ESI, +): m/z [M+H]^+^ calc. 523.03922, found 523.03937; ESI-MS (DI, -): m/z (%): 521 [M-H]^-^ (100); HPLC (isocr.): 94.1% at 254 nm, 94.1% at 280 nm, t_M+S_ = 4.1 min, t_M_ (DMSO) = 1.1 min (ACN/buffer pH 2.7 35:65), λ_max_ [nm] = 233, 321.

*2-(3*,*9-Dichloro-6-oxo-7*,*12-dihydrobenzo[2*,*3]azepino[4*,*5-b]indol-5(6H)-yl)-N-[2-(methylamino)ethyl]acetamide (1g)*. According to general procedure 6 from *tert*-butyl {2-[2-(3,9-dichloro-6-oxo-7,12-dihydrobenzo[2,3]azepino[4,5-*b*]indol-5(6*H*)-yl)acetamido]ethyl}(methyl)carbamate (**10b**, 159 mg, 0.300 mmol) in trifluoroacetic acid (2.3 mL, 30 mmol) and dichloromethane (7 mL) (23 h). After removal of the solvent by distillation under reduced pressure, the residue was mixed with water (10 mL) and alkalised to pH 11 with sodium hydroxide solution (8% m/m) and diluted to an approximate volume of 50 mL, then extracted with dichloromethane (6x 50 mL), then toluene (50 mL). An insoluble residue was filtrated off and the combined organic phases were subjected to reduced pressure until a solid remained. Recrystallisation of the obtained solids from ethanol and drying (4 h, 60°C) led to la light yellow solid (75 mg). Recrystallisation from ethyl acetate (12.5 mL) and drying (4 h, 60°C) led to an off-white solid (7 mg, 16 μmol, 5%). Decomp.: starting at 240°C; IR (KBr): ṽ [cm^-1^] = 3423 (N-H), 1641 (C = O); ^1^H-NMR (DMSO-*d*_6_, 500 MHz): δ (ppm) = 2.27 (s, 3H, *N*-CH_3_), 2.53 (t, *J* = 6.3 Hz, 2H, *N*-CH_2_), 3.17 (q, *J* = 6.1 Hz, 2H, *N*-CH_2_), 3.95 (br s, 1H, azepine-CH_2_), 4.16 (br s, 1H, *N*-CH_2_), 4.35 (br s, 1H, *N*-CH_2_), 7.19 (dd, *J* = 8.6 / 2.1 Hz, 1H, ArH), 7.43–7.51 (m, 2H, ArH, d and dd superimposed), 7.68 (d, *J* = 2.1 Hz, 1H, ArH), 7.72 (d, *J* = 8.4 Hz, 1H, ArH), 7.81 (d, *J* = 2.0 Hz, 1H, ArH), 8.04 (t, *J* = 5.6 Hz, 1H, amide-NH), 11.97 (br s, 1H, indole-NH); The signals between 3.95 ppm and 4.35 ppm overlap; The signals for the amine proton and the azepine-CH_2_ group are not assignable in the ^1^H experiment; ^13^C-NMR (DMSO-*d*_6_, 126 MHz): δ (ppm) = 35.6 (CH_3_), 31.1 (w in DEPT135), 38.3, 50.4, 53.5 (w in DEPT135) (CH_2_), 113.1, 117.6, 122.3, 124.0, 125.0, 128.5 (CH), 109.4, 123.90, 123.93, 127.0, 132.3, 133.1, 135.7, 141.0, 168.1, 170.1 (C); The structure was confirmed by 2D-NMR spectroscopy (H,H-COSY, H,C-HSQC); C_21_H_20_Cl_2_N_4_O_2_ (431.32); HRMS (ESI, +): m/z [M+H]^+^ calc. 431.10361, found 431.10405; ESI-MS (DI, -): m/z (%): 429 [M-H]^-^ (100); HPLC (isocr.): 93.5% at 254 nm, 96.4% at 280 nm, t_M+S_ = 3.1 min, t_M_ (DMSO) = 1.1 min (ACN/buffer pH 2.7 35:65), λ_max_ [nm] = 232, 319.

*2-(3-Chloro-6-oxo-9-trifluoromethyl-7*,*12-dihydrobenzo[2*,*3]azepino[4*,*5-b]indol-5(6H)-yl)-N-[2-(methylamino)ethyl]acetamide (1h)*. According to general procedure 6 from *tert*-butyl {2-[2-(3-chloro-6-oxo-9-trifluoromethyl-7,12-dihydrobenzo[2,3]azepino[4,5-*b*]indol-5(6*H*)-yl)acetamido]ethyl}(methyl)carbamate (**10c**, 198 mg, 0.350 mmol) in trifluoroacetic acid (2.7 mL, 35 mmol) and dichloromethane (10 mL) (22 h). After removal of the solvent by distillation under reduced pressure, the oily residue was mixed with water (10 mL) and alkalised to pH 11 with sodium hydroxide solution (8% m/m) to obtain a flaky precipitate that was filtrated off and dried (5 h, 60°C) (154 mg). Recrystallisation from THF (7.5 mL) and water (10 mL) and drying (3 h at 60°C, 1 h at 100°C and 2 h at 60°C) yielded a colourless solid (100 mg, 0.215 mmol, 61%). Decomp.: starting at 264°C; IR (KBr): ṽ [cm^-1^] = 3321 (N-H), 1642 (C = O), 1305, 1101; ^1^H-NMR (DMSO-*d*_6_, 500 MHz): δ (ppm) = 2.29 (s, 3H, *N*-CH_3_), 2.55 (t, *J* = 6.3 Hz, 2H, *N*-CH_2_, overlaps with CHD_2_-signal at 2.51 ppm of DMSO-*d*_6_ after H/D-exchange), 3.18 (q, *J* = 6.1 Hz, 2H, *N*-CH_2_), 4.17 (br s, 2H, *N*-CH_2_ / azepine-CH_2_), 4.35 (br s, 1H, *N*-CH_2_), 7.46–7.53 (m, 2H, ArH, 2x dd superimposed), 7.64 (dt, *J* = 8.6 / 0.8 Hz, 1H, ArH), 7.70 (d, *J* = 2.1 Hz, 1H, ArH), 7.75 (d, *J* = 8.4 Hz, 1H, ArH), 8.07 (t, *J* = 5.6 Hz, 1H, amide-NH, near complete H/D exchange), 8.18 (dt, *J* = 1.7 / 0.8 Hz, 1H, ArH), 12.25 (br s, 1H, indole-NH, complete H/D exchange); The signals between 4.17 ppm and 4.35 ppm overlap; The signals for the amine proton and the azepine-CH_2_ group are not assignable in the ^1^H experiment; ^13^C-NMR (DMSO-*d*_6_, 126 MHz): δ (ppm) = 35.5 (CH_3_), 31.0 (w in DEPT135), 38.1, 50.3, 53.4 (CH_2_), 112.3, 116.22 (q, *J* = 4.2 Hz), 118.57 (q, *J* = 3.2 Hz), 124.1, 125.1, 128.5 (CH), 110.6, 120.17 (q, *J* = 31.2 Hz), 123.8, 125.3, 125.78 (q, *J* = 271.4 Hz), 132.6, 133.6, 138.7, 141.1, 168.1, 170.1 (C); C_22_H_20_ClF_3_N_4_O_2_ (464.87); HRMS (ESI, +): m/z [M+H]^+^ calc. 465.12996, found 465.13084; APCI-MS (ASAP, -): m/z (%): 463 [M-H]^-^ (100), 219 [M-245]^-^ (28); ESI-MS (DI, -): m/z (%): 463 [M-H]^-^ (100); HPLC (isocr.): 95.3% at 254 nm, 97.7% at 280 nm, t_M+S_ = 4.3 min, t_M_ (DMSO) = 1.1 min (ACN/buffer pH 2.7 35:65), λ_max_ [nm] = 233, 315.

*tert-Butyl 4-{2-[2-(2-oxo-2*,*3*,*4*,*5-tetrahydro-1H-benzo[b]azepin-1-yl)acetamido]ethyl}piperazine-1-carboxylate (2b)*. According to general procedure 4 from 2-(2-oxo-2,3,4,5-tetrahydro-1*H*-benzo[*b*]azepin-1-yl)acetic acid (**13**, 220 mg, 1.00 mmol), HOBt (162 mg, 1.20 mmol), EDCl (249 mg, 1.30 mmol), DIPEA (205 μL, 1.20 mmol) and *tert*-butyl 4-(2-aminoethyl)piperazine-1-carboxylate (252 mg, 1.10 mmol) in DMF (6 mL) (17 h). Water (100 mL) was added. After stirring for 30 min, the mixture was extracted with ethyl acetate (3x 100 mL). The combined and dried organic phases were subjected to reduced pressure until a brown oil remained which was further purified by column chromatography (silica gel, eluent ethyl acetate/ethanol/triethylamine 18:1:1). After drying under reduced pressure (60°C) a glassy, yellow solid was obtained (200 mg). Recrystallisation of 80 mg of the compound from ethyl acetate (0.5 mL) and drying (60°C) yielded a colourless solid (40 mg, 0.093 mmol, 9%). M.p.: 118–120°C; IR (KBr): ṽ [cm^-1^] = 3321 (N-H), 1666 (C = O), 1459, 1421, 1245, 1170; ^1^H-NMR (DMSO-*d*_6_, 600 MHz): δ (ppm) = 1.39 (s, 9H, 3xCH_3_), 2.05 (br s, 2H, azepine-CH_2_), 2.15 (t, *J* = 7.0 Hz, 2H, azepine-CH_2_), 2.24–2.38 (m = 2x t superimposed, 6H, *N*-CH_2_), 2.78 (br s, 2H, azepine-CH_2_), 3.16 (dt, *J* = 6.4 Hz, 2H, amide-*N*-CH_2_), 3.27 (t, *J* = 5.0 Hz, 4H, 2x *N*-CH_2_), 4.30 (s, 2H, azepine-*N*-CH_2_), 7.13–7.18 (m, 1H, ArH), 7.23–7.29 (m, 3H, ArH), 7.92 (t, *J* = 5.7 Hz, 1H, NH); ^13^C-NMR (DMSO-*d*_6_, 151 MHz): δ (ppm) = 28.0 (3C) (CH_3_), 28.2, 29.1, 32.5, 36.0, 42.6 (br, w), 43.7 (br, w), 50.6, 52.3, 56.7 (CH_2_), 122.3, 125.8, 127.2, 129.1 (CH), 78.6, 135.3, 142.8, 153.7, 167.9, 171.8 (C); The structure was confirmed by 2D-NMR spectroscopy (H,H-COSY, H,C-HSQC, H,C-HMBC, H,N-HMBC); C_23_H_34_N_4_O_4_ (430.55); CHN: calc. C 64.16, H 7.96, N 13.01, found C 64.37, H 8.00, N 12.76; APCI-MS (ASAP, +): m/z (%): 431 [M+H]^+^ (100), 375 [M-56]^+^ (14), 331 [M-100]^+^ (12); ESI-MS (DI, +): m/z (%): 453 [M+Na]^+^ (100), 431 [M+H]^+^ (20); HPLC (isocr.): 99.0% at 254 nm, t_M+S_ = 3.4 min, t_M_ (DMSO) = 1.2 min (ACN/buffer pH 2.7 = 30:70); λ_max_ [nm] = 238.

*tert-Butyl {2-[2-(2-oxo-2*,*3*,*4*,*5-tetrahydro-1H-benzo[b]azepin-1-yl)acetamido]ethyl}carbamate (2c)*. According to general procedure 4 from 2-(2-oxo-2,3,4,5-tetrahydro-1*H*-benzo[*b*]azepin-1-yl)acetic acid (**13**, 110 mg, 0.502 mmol), HOBt (81 mg, 0.60 mmol), EDCl (125 mg, 0.650 mmol), DIPEA (103 μL, 0.603 mmol) and *tert*-butyl (2-aminoethyl)carbamate (285 μL, 1.80 mmol) in DMF (4 mL) (96 h). Aqueous sodium chloride solution (20%, 50 mL) was added and the mixture was extracted with ethyl acetate (3x 50 mL). The combined dried organic phases were subjected to reduced pressure until a brown oil remained that was purified by column chromatography (silica gel, eluent ethyl acetate/ethanol/triethylamine 18:1:1). After drying under reduced pressure (60°C), a light-yellow solid was obtained (150 mg, 0.450 mmol, 83%). M.p.: 146–147°C; IR (KBr): ṽ [cm^-1^] = [3356, 3300] (NH), [1683, 1657] (C = O), 1520, 1368, 1172; ^1^H-NMR (DMSO-*d*_6_, 600 MHz): δ (ppm) = 1.37 (s, 9H, 3xCH_3_), 2.05 (br s, 2H, azepine-CH_2_), 2.15 (t, *J* = 7.0 Hz, 2H, azepine-CH_2_), 2.86 (br s, 2H, azepine-CH_2_), 2.95 (dt, *J* = 6.4 Hz, 2H, *N*-CH_2_), 3.06 (dt, *J* = 6.4 Hz, 2H, *N*-CH_2_), 4.28 (s, 2H, azepine-*N*-CH_2_), 6.78 (t, *J* = 5.8 Hz, 1H, NH), 7.16 (ddd, *J* = 7.3 / 1.4 Hz, 1H, ArH), 7.23–7.30 (m, 3H, ArH), 8.02 (t, *J* = 5.7 Hz, 1H, NH); ^13^C-NMR (DMSO-*d*_6_, 151 MHz): δ (ppm) = 28.1 (3C) (CH_3_), 28.2, 29.1, 32.5, 38.7, 39.4, 50.7 (CH_2_), 122.4, 125.8, 127.2, 129.1 (CH), 77.6, 135.3, 142.8, 155.5, 168.1, 171.8 (C); C_19_H_27_N_3_O_4_ (361.44); CHN: calc. C 63.14, H 7.53, N 11.63, found C 63.46, H 7.53, N 11.52; APCI-MS (ASAP, +): m/z (%): 624 [M+263]^+^ (7), 262 [M-99]^+^ (70), 202 [M-159]^+^ (44), 174 [M-159]^+^ (100), 146 [M-159]^+^ (52); ESI-MS (DI, +): m/z (%): 384 [M+Na]^+^ (100); HPLC (grad.): 98.0% at 254 nm, t_M+S_ = 9.4 min, t_M_ (DMSO) = 1.2 min, λ_max_ [nm] = 239; HPLC (isocr.): 98.7% at 254 nm, t_M+S_ = 3.9 min, t_M_ (DMSO) = 1.2 min (ACN/buffer pH 2.7 40:60), λ_max_ [nm] = 238.

*tert-Butyl 4-[2-(2-oxo-2*,*3*,*4*,*5-tetrahydro-1H-benzo[b]azepin-1-yl)acetyl]piperazine-1-carboxylate (2d)*. According to general procedure 4 from 2-(2-oxo-2,3,4,5-tetrahydro-1*H*-benzo[*b*]azepin-1-yl)acetic acid (**13**, 220 mg, 1.00 mmol), HOBt (162 mg, 1.20 mmol), EDCl (250 mg, 1.30 mmol), DIPEA (208 μL, 1.20 mmol) and *tert*-butyl piperazine-1-carboxylate (223 mg, 1.20 mmol) in DMF (5 mL) (40 h). Water (20 mL) was added and the mixture was extracted according to general procedure 5 (4x 20 mL water and 4x 20 mL dichloromethane). After removing the solvent from the combined organic phases under vacuum, the semi-solid, brownish residue was recrystallised from ethyl acetate (2.0 mL) and ethanol (0.1 mL). A light yellow solid was obtained after drying (2 h, 60°C) (241 mg, 0.620 mmol, 62%). M.p.: 166–167°C; IR (KBr): ṽ [cm^-1^] = [1691, 1653] (C = O), 1461, 1422, 1172; ^1^H-NMR (DMSO-*d*_6_, 400 MHz): δ (ppm) = 1.41 (s, 9H, 3x CH_3_), 2.06 (br s, 2H, azepine-CH_2_), 2.16 (t, *J* = 7.0 Hz, 2H, azepine-CH_2_), 2.92 (br s, 2H, azepine-CH_2_), 3.24–3.31 (m, 2H, piperazine-CH_2_), 3.33–3.38 (m, 2H, piperazine-CH_2_), 3.38–3.43 (m, 2H, piperazine-CH_2_), 3.43–3.51 (m, 2H, piperazine-CH_2_), 4.64 (s, 2H, azepine-*N*-CH_2_), 7.11–7.18 (m, 1H, ArH), 7.20–7.31 (m, 3H, ArH); The signals at 3.24 ppm and 3.51 ppm overlap with each other and the HDO signal; ^13^C-NMR (DMSO-*d*_6_, 101 MHz): δ (ppm) = 28.0 (3C) (CH_3_), 28.3, 29.1, 32.5, 41.1, 43.9, 49.1 (CH_2_), 122.3, 125.8, 127.2, 129.2 (CH), 79.1, 135.6, 142.7, 153.8, 166.4, 171.8 (C); C_21_H_29_N_3_O_4_ (387.48); CHN: calc. C 65.10, H 7.54, N 10.84, found C 65.24, H 7.55, N 10.82; APCI-MS (ASAP, +): m/z (%): 676 [M+289]^+^ (4), 388 [M+H]^+^ (6), 332 [M-55]^+^ (17), 288 [M-99]^+^ (38), 202 [M-185]^+^ (70), 174 [M-213]^+^ (100), 146 [M-241]^+^ (34); ESI-MS (DI, +): m/z (%): 797 [2M+Na]^+^ (5), 410 [M+Na]^+^ (100); HPLC (grad.): 98.4% at 254 nm, t_M+S_ = 10.2 min, t_M_ (DMSO) = 1.2 min, λ_max_ [nm] = 239; HPLC (isocr.): 98.5% at 254 nm, t_M+S_ = 10.2 min, t_M_ (DMSO) = 1.2 min (ACN/water 40:60), λ_max_ [nm] = 238.

*tert-Butyl methyl-{2-[2-(2-oxo-2*,*3*,*4*,*5-tetrahydro-1H-benzo[b]azepin-1-yl)acetamido]ethyl}carbamate (2e)*. According to general procedure 4 from 2-(2-oxo-2,3,4,5-tetrahydro-1*H*-benzo[*b*]azepin-1-yl)acetic acid (**13**, 219 mg, 1.00 mmol), HOBt (162 mg, 1.20 mmol), EDCl (250 mg, 1.30 mmol), DIPEA (208 μL, 1.20 mmol) and *N*-(*tert*-butoxycarbonyl)-*N*-methylethane-1,2-diamine (209 mg, 1.20 mmol) in DMF (3 mL) (29 h). Water (20 mL) was added and the mixture was extracted according to general procedure 5 (4x 20 mL water and 4x 20 mL dichloromethane). After removing the solvent from the combined organic phases under vacuum, the brown oil was layered with petroleum ether (10 mL) and stirred overnight. The resulting beige solid was dried for 2 h at 60°C and recrystallised from petroleum ether (1 mL) and ethyl acetate (1.8 mL) to yield a colourless solid after drying (195 mg, 0.519 mmol, 52%). M.p.: 132–133°C; IR (KBr): ṽ [cm^-1^] = 3350 (N-H), [1688, 1638] (C = O), 1394, 1388, 1155; ^1^H-NMR (DMSO-*d*_6_, 400 MHz): δ (ppm) = 1.37 (s, 9H, 3x CH_3_), 2.05 (br s, 2H, azepine-CH_2_), 2.15 (t, *J* = 7.0 Hz, 2H, azepine-CH_2_), 2.68–2.96 (m, 5H, *N*-CH_3_ and azepine-CH_2_ superimposed), 3.11–3.22 (m, 4H, 2x *N*-CH_2_), 4.21–4.34 (m, 2H, azepine-*N*-CH_2_, peak split due to *N*-Boc rotamers), 7.11–7.19 (m, 1H, ArH), 7.21–7.32 (m, 3H, ArH), 8.00–8.20 (m, 1H, NH, peak split due to *N*-Boc rotamers); ^13^C-NMR (DMSO-*d*_6_, 101 MHz): δ (ppm) = 28.0 (3C), [34.3 (br, w), 34.5 (br, w) split due to *N*-Boc rotamers] (CH_3_), 28.2, 29.1, 32.6, [36.8 (br, w), 37.1 (br, w) split due to *N*-Boc rotamers], [47.3 (br, w), 47.8 (br, w) split due to *N*-Boc rotamers], 50.7 (CH_2_), 122.4, 125.9, 127.3, 129.2 (CH), 78.4, 135.4, 142.9, [154.7 (br, w), 154.9 (br, w) split due to *N*-Boc rotamers], 168.2, 171.8 (C); The structure was confirmed by 2D-NMR spectroscopy (H,C-HSQC, H,C-HMBC); C_20_H_29_N_3_O_4_ (375.47); CHN: calc. C 63.98, H 7.79, N 11.19, found C 64.31, H 7.79, N 10.93; APCI-MS (ASAP, +): m/z (%): 330 [M-45]^+^ (1), 276 [M-99]^+^ (100), 245 [M-130]^+^ (10), 202 [M-173]^+^ (23), 174 [M-201]^+^ (38), 146 [M-221]^+^ (24); ESI-MS (DI, +): m/z (%): 414 [M+K]^+^ (5), 398 [M+Na]^+^ (100); HPLC (grad.): 98.0% at 254 nm, t_M+S_ = 9.9 min, t_M_ (DMSO) = 1.2 min, λ_max_ [nm] = 241; HPLC (isocr.): 98.3% at 254 nm, t_M+S_ = 5.1 min, t_M_ (DMSO) = 1.1 min (ACN/water 40:60), λ_max_ [nm] = 238.

*tert-Butyl {4-[2-(2-oxo-2*,*3*,*4*,*5-tetrahydro-1H-benzo[b]azepin-1-yl)acetamido]butyl}carbamate (2f)*. According to general procedure 4 from 2-(2-oxo-2,3,4,5-tetrahydro-1*H*-benzo[*b*]azepin-1-yl)acetic acid (**13**, 438 mg, 2.00 mmol), HOBt (324 mg, 2.40 mmol), EDCl (498 mg, 2.60 mmol), DIPEA (0.42 mL, 2.4 mmol) and *tert*-butyl (4-aminobutyl)carbamate (0.46 mL, 2.4 mmol) in DMF (6 mL) (24 h). Water (20 mL) was added and the mixture was extracted according to general procedure 5 (3x 20 mL water and 5x 20 mL dichloromethane). After removing the solvent from the combined organic phases under vacuum, the brown oil was layered with petroleum ether (15 mL) and ethyl acetate (total volume 0.8 mL) was added dropwise until a brown solid formed that was recrystallised from ethyl acetate (2 mL) and dried (60°C, 2 h) to yield a light beige solid (340 mg, 0.873 mmol, 44%). M.p.: 99–101°C; IR (KBr): ṽ [cm^-1^] = [3414, 3369, 3305] (N-H), [1711, 1685, 1658] (C = O), 1513, 1252, 1170; ^1^H-NMR (DMSO-*d*_6_, 500 MHz): δ (ppm) = 1.27–1.35 (m, 4H, 2x CH_2_), 1.37 (s, 9H, 3x CH_3_), 2.02 (br s, 2H, azepine-CH_2_), 2.15 (t, *J* = 7.0 Hz, 2H, azepine-CH_2_), 2.66–2.97 (m, 4H, azepine-CH_2_ and *N*-CH_2_), 3.01 (q, *J* = 6.3 Hz, 2H, *N*-CH_2_), 4.29 (s, 2H, azepine-*N*-CH_2_), 6.77 (t, *J* = 5.8 Hz, 1H, NH), 7.15 (td, *J* = 7.3 / 1.5 Hz, 1H, ArH), 7.21–7.32 (m, 3H, ArH), 7.97 (t, *J* = 5.6 Hz, 1H, NH); ^13^C-NMR (DMSO-*d*_6_, 126 MHz): δ (ppm) = 28.2 (3C + 2C) (CH_3_), 26.4, 26.8, 29.1, 32.5, 38.2, 39.4 (superimposed onto the DMSO-*d*_6_ signal), 50.5 (CH_2_), 122.3, 125.8, 127.2, 129.1 (CH), 77.2, 135.3, 142.8, 155.5, 167.7, 171.8 (C); Two signal groups at 28.2 ppm are isochronous by coincidence (CH_3_ and CH_2_); The structure was confirmed by 2D-NMR spectroscopy (H,C-HSQC); C_21_H_31_N_3_O_4_ (389.50); CHN: calc. C 64.76, H 8.02, N 10.79, found C 64.60, H 7.93, N 10.68; APCI-MS (ASAP, +): m/z (%): 390 [M+H]^+^ (1), 334 [M-55]^+^ (4), 290 [M-99]^+^ (100), 202 [M-187]^+^ (18), 174 [M-215]^+^ (22); ESI-MS (DI, +): m/z (%): 412 [M+Na]^+^ (100); HPLC (grad.): 98.8% at 254 nm, t_M+S_ = 9.7 min, t_M_ (DMSO) = 1.2 min, λ_max_ [nm] = 241; HPLC (isocr.): 99.8% at 254 nm, t_M+S_ = 4.7 min, t_M_ (DMSO) = 1.1 min (ACN/water 40:60), λ_max_ [nm] = 239.

*tert-Butyl {1-[2-(2-oxo-2*,*3*,*4*,*5-tetrahydro-1H-benzo[b]azepin-1-yl)acetyl]piperidine-4-yl}carbamate (2g)*. According to general procedure 4 from 2-(2-oxo-2,3,4,5-tetrahydro-1*H*-benzo[*b*]azepin-1-yl)acetic acid (**13**, 434 mg, 1.98 mmol), HOBt (324 mg, 2.40 mmol), EDCl (500 mg, 2.60 mmol), DIPEA (415 μL, 2.40 mmol) and *tert*-butyl piperidine-4-yl-carbamate (483 mg, 2.41 mmol) in DMF (4 mL) (23 h). Cold aqueous sodium chloride solution (1 M, 20 mL) was added. A precipitate was filtered off and extracted according to general procedure 5 (2x 20 mL 1 M cold aqueous sodium chloride solution and 3x 40 mL ethyl acetate). The combined organic phases were subjected to reduced pressure to yield a brown solid (0.49 g). The filtrate was extracted according to general procedure 5 (2x 20 mL water and 3x 40 mL ethyl acetate) and yielded a brownish oil that solidified upon addition of petroleum ether (10 mL). The combined solids were recrystallised from ethyl acetate (8.9 mL) and petroleum ether (10 mL). After drying at 60°C for 2 h, a light beige solid was obtained (347 mg, 0.864 mmol, 44%). M.p.: 168°C; IR (KBr): ṽ [cm^-1^] = 3311 (N-H), [1695, 1668, 1646] (C = O), 1531, 1446, 1316, 1232, 1161; ^1^H-NMR (DMSO-*d*_6_, 500 MHz): δ (ppm) = 1.12–1.24 (m, 1H, CH_2_), 1.26–1.35 (m, 1H, CH_2_), 1.38 (s, 9H, 3x CH_3_), 1.69 (br d, *J* = 11.7 Hz, 1H, CH_2_), 1.76 (br d, *J* = 11.5 Hz, 1H, CH_2_), 2.06 (br s, 2H, azepine-CH_2_), 2.15 (t, *J* = 6.9 Hz, 2H, azepine-CH_2_), 2.69 (apparent t, *J* = 12.2 Hz, 1H, *N*-CH_2_), 2.92 (br s, 2H, azepine-CH_2_), 3.08 (apparent t, *J* = 12.2 Hz, 1H, *N*-CH_2_), 3.44–3.50 (m, 1H, *N*-CH), 3.81 (br d, *J* = 13.6 Hz, 1H, *N*-CH_2_), 4.13 (br d, *J* = 13.5 Hz, 1H, *N*-CH_2_), 4.58 (d, *J* = 16.8 Hz, 1H, azepine-*N*-CH_2_, part B of an AB system), 4.65 (d, *J* = 16.8 Hz, 1H, azepine-*N*-CH_2_, part A of an AB system), 6.89 (br d, *J* = 7.9 Hz, 1H, NH), 7.14 (td, *J* = 7.3 / 1.6 Hz, 1H, ArH), 7.20–7.30 (m, 3H, ArH); ^13^C-NMR (d_6_-DMSO, 126 MHz): δ (ppm) = 28.2 (3C) (CH_3_), 28.3, 29.1, 31.3, 32.1, 32.5, 40.3, 42.9, 49.0 (CH_2_), 47.0, 122.2, 125.7, 127.2, 129.2 (CH), 77.5, 135.6, 142.7, 154.7, 165.8, 171.7 (C); The structure was confirmed by 2D-NMR spectroscopy (H,H-COSY, H,C-HSQC); C_22_H_31_N_3_O_4_ (401.51); CHN: calc. C 65.81, H 7.78, N 10.47, found C 66.00, H 7.87, N 10.36; APCI-MS (ASAP, +, soft): m/z (%): 402 [M+H]^+^ (8), 346 [M-55]^+^ (50), 302 [M-99]^+^ (18), 202 [M-199]^+^ (100), 174 [M-227]^+^ (54); ESI-MS (DI, +): m/z (%): 440 [M+K]^+^ (4), 424 [M+Na]^+^ (100); HPLC (grad.): 99.7% at 254 nm, t_M+S_ = 9.9 min, t_M_ (DMSO) = 1.3 min, λ_max_ [nm] = 218, 240; HPLC (isocr.): 99.5% at 254 nm, t_M+S_ = 5.7 min, t_M_ (DMSO) = 1.2 min (ACN/water 40:60), λ_max_ [nm] = 217, 239.

*1-(2-Morpholino-2-oxoethyl)-1*,*3*,*4*,*5-tetrahydro-2H-benzo[b]azepin-2-one (2h)*. According to general procedure 4 from 2-(2-oxo-2,3,4,5-tetrahydro-1*H*-benzo[*b*]azepin-1-yl)acetic acid (**13**, 110 mg, 0.502 mmol), HOBt (81 mg, 0.60 mmol), EDCl (125 mg, 0.650 mmol), DIPEA (104 μL, 0.601 mmol) and morpholine (52 μL, 0.60 mmol) in DMF (4 mL) (44 h). Water (20 mL) was added and the mixture was extracted according to general procedure 5 (4x 20 mL water and 4x 20 mL dichloromethane). After removing the solvent from the combined organic phases under vacuum, the yellow solid was recrystallised from ethyl acetate (1 mL) to yield a colourless solid after drying (40 mg, 0.14 mmol, 28%). M.p.: 160°C; IR (KBr): ṽ [cm^-1^] = 1650 (C = O), 1458, 1236; ^1^H-NMR (DMSO-*d*_6_, 500 MHz): δ (ppm) = 2.06 (br s, 2H, azepine-CH_2_), 2.16 (t, *J* = 6.9 Hz, 2H, azepine-CH_2_), 2.92 (br s, 2H, azepine-CH_2_), 3.40 (t, *J* = 4.9 Hz, 2H, morpholine-CH_2_), 3.48 (t, *J* = 4.8 Hz, 2H, morpholine-CH_2_), 3.54 (t, *J* = 4.9 Hz, 2H, morpholine-CH_2_), 3.59 (t, *J* = 4.7 Hz, 2H, morpholine-CH_2_), 4.64 (s, 2H, azepine-*N*-CH_2_), 7.15 (ddd, *J* = 7.2 / 1.3 Hz, 1H, ArH), 7.21–7.31 (m, 3H, ArH); ^13^C-NMR (DMSO-*d*_6_, 126 MHz): δ (ppm) = 28.3, 29.0, 32.5, 41.6, 44.6, 49.0, 65.87, 65.94 (CH_2_), 122.3, 125.7, 127.2, 129.1 (CH), 135.6, 142.6, 166.3, 171.8 (C); C_16_H_20_N_2_O_3_ (288.35); CHN: calc. C 66.65, H 6.99, N 9.72, found C 66.72, H 7.26, N 9.73; APCI-MS (ASAP, +): m/z (%): 289 [M+H]^+^ (1), 202 [M-86]^+^ (28), 174 [M-114]^+^ (97), 146 [M-142]^+^ (100); ESI-MS (DI, +): m/z (%): 599 [2M+Na]^+^ (5), 311 [M+Na]^+^ (100); HPLC (grad.): 99.5% at 254 nm, t_M+S_ = 8.0 min, t_M_ (DMSO) = 1.2 min, λ_max_ [nm] = 239; HPLC (isocr.): 99.6% at 254 nm, t_M+S_ = 3.8 min, t_M_ (DMSO) = 1.1 min (ACN/buffer pH 2.7 30:70), λ_max_ [nm] = 237.

*1-[2-Oxo-2-(piperidin-1-yl)ethyl]-1*,*3*,*4*,*5-tetrahydro-2H-benzo[b]azepin-2-one (2i)*. According to general procedure 4 from 2-(2-oxo-2,3,4,5-tetrahydro-1*H*-benzo[*b*]azepin-1-yl)acetic acid (**13**, 110 mg, 0.502 mmol), HOBt (81 mg, 0.60 mmol), EDCl (125 mg, 0.650 mmol), DIPEA (105 μL, 0.607 mmol) and piperidine (59 μL, 0.60 mmol) in DMF (4 mL) (44 h). Water (20 mL) was added and the mixture was extracted according to general procedure 5 (4x 20 mL water and 4x 20 mL dichloromethane). After removing the solvent from the combined organic phases under vacuum, the yellowish solid was recrystallised from ethyl acetate (1.2 mL) to yield a light beige solid (103 mg, 0.360 mmol, 72%). M.p.: 151–152°C; IR (KBr): ṽ [cm^-1^] = 1643 (C = O), 1599, 1457, 1441, 1388, 1249, 1225; ^1^H-NMR (DMSO-*d*_6_, 500 MHz): δ (ppm) = 1.36–1.44 (m, 2H, piperidine-CH_2_), 1.46–1.54 (m, 2H, piperidine-CH_2_), 1.54–1.62 (m, 2H, piperidine-CH_2_), 2.06 (br s, 2H, azepine-CH_2_), 2.15 (t, *J* = 6.6 Hz, 2H, azepine-CH_2_), 2.92 (br s, 2H, azepine-CH_2_), 3.35–3.43 (m, 4H, piperidine-CH_2_), 4.60 (s, 2H, azepine-*N*-CH_2_), 7.14 (ddd, *J* = 1.5 Hz, 1H, ArH), 7.21–7.30 (m, 3H, ArH); ^13^C-NMR (DMSO-*d*_6_, 126 MHz): δ (ppm) = 23.8, 25.1, 25.9, 28.3, 29.1, 32.5, 42.3, 45.0, 49.1 (CH_2_), 122.2, 125.6, 127.2, 129.1 (CH), 135.6, 142.7, 165.7, 171.7 (C); C_17_H_22_N_2_O_2_ (286.38); CHN: calc. C 71.30, H 7.74, N 9.78, found C 71.03, H 8.07, N 9.77; APCI-MS (ASAP, +): m/z (%): 287 [M+H]^+^ (9), 202 [M-84]^+^ (40), 174 [M-112]^+^ (100), 146 [M-140]^+^ (81); ESI-MS (DI, +): m/z (%): 595 [2M+Na]^+^ (11), 309 [M+Na]^+^ (100); HPLC (grad.): 98.3% at 254 nm, t_M+S_ = 9.6 min, t_M_ (DMSO) = 1.2 min, λ_max_ [nm] = 239; HPLC (isocr.): 97.9% at 254 nm, t_M+S_ = 4.8 min, t_M_ (DMSO) = 1.1 min (ACN/buffer pH 2.7 40:60), λ_max_ [nm] = 238.

*1-[2-Oxo-2-(pyrrolidin-1-yl)ethyl]-1*,*3*,*4*,*5- tetrahydro-2H-benzo[b]-azepin-2-one (2j)*. According to general procedure 4 from 2-(2-oxo-2,3,4,5-tetrahydro-1*H*-benzo[*b*]azepin-1-yl)acetic acid (**13**, 436 mg, 1.99 mmol), HOBt (325 mg, 2.40 mmol), EDCl (502 mg, 2.62 mmol), DIPEA (415 μL, 2.40 mmol) and pyrrolidine (200 μL, 2.40 mmol) in DMF (4 mL) (22.5 h). Cold aqueous sodium chloride solution (1 M, 20 mL) was added and the mixture was extracted according to general procedure 5 (2x 30 mL 1 M cold aqueous sodium chloride solution and 3x 40 mL ethyl acetate). The brownish crystals obtained after removal of the solvent by distillation under reduced pressure from the combined organic phases were recrystallised from ethyl acetate (3.7 mL) and petroleum ether (10 mL) and the hot solution was decanted. Light brown crystals were obtained after drying (60°C, 2 h) (225 mg, 0.826 mmol, 42%). M.p.: 114–115°C; IR (KBr): ṽ [cm^-1^] = 1656 (C = O), 1598 (C = C Ar), 1438, 1375; ^1^H-NMR (DMSO-*d*_6_, 400 MHz): δ (ppm) = 1.69–1.81 (m, 2H, CH_2_), 1.83–1.95 (m, 2H, CH_2_), 2.06 (br s, 2H, azepine-CH_2_), 2.15 (t, *J* = 7.1 Hz, 2H, azepine-CH_2_), 2.92 (br s, 2H, azepine-CH_2_), 3.27 (t, *J* = 6.9 Hz, 2H, CH_2_), 3.45 (t, *J* = 6.8 Hz, 2H, CH_2_), 4.51 (s, 2H, azepine-*N*-CH_2_), 7.09–7.20 (m, 1H, ArH), 7.21–7.31 (m, 3H, ArH); ^13^C-NMR (DMSO, 101 MHz): δ (ppm) = 23.6. 25.6, 28.3, 29.1, 32.5, 45.0, 45.6, 50.0 (CH_2_), 122.4, 125.7, 127.2, 129.1 (CH), 135.6, 142.8, 165.8, 171.7 (C); C_16_H_20_N_2_O_2_ (272.35); CHN: calc. C 70.56, H 7.40, N 10.29, found C 70.58, H 7.38, N 10.24; APCI-MS (ASAP, +, soft): m/z (%): 273 [M+H]^+^ (50), 202 [M-70]^+^ (100), 174 [M-98]^+^ (88), 146 [M-126]^+^ (9); ESI-MS (DI, +): m/z (%): 567 [2M+Na]^+^ (10), 295 [M+Na]^+^ (100); HPLC (grad.): 98.0% at 254 nm, t_M+S_ = 8.9 min, t_M_ (DMSO) = 1.2 min, λ_max_ [nm] = 241; HPLC (isocr.): 98.5% at 254 nm, t_M+S_ = 3.2 min, t_M_ (DMSO) = 1.20 min (ACN/water 40:60), λ_max_ [nm] = 240.

*N*,*N-Diethyl-2-(2-oxo-2*,*3*,*4*,*5-tetrahydro-1H-benzo[b]azepin-1-yl)acetamide (2k)*. According to general procedure 4 from 2-(2-oxo-2,3,4,5-tetrahydro-1*H*-benzo[*b*]azepin-1-yl)acetic acid (**13**, 439 mg, 2.00 mmol), HOBt (329 mg, 2.43 mmol), EDCl (502 mg, 2.62 mmol), DIPEA (830 μL, 4.80 mmol) and diethylamine hydrochloride (263 mg, 2.40 mmol) in DMF (4 mL) (19 h). Cold aqueous sodium chloride solution (1 M, 20 mL) was added and the mixture was extracted according to general procedure 5 (2x 30 mL 1 M cold aqueous sodium chloride solution and 3x 40 mL ethyl acetate). After removal of the solvent by distillation under reduced pressure from the combined organic phases, the obtained brown oil was purified by column chromatography (silica gel, eluent ethyl acetate/petroleum ether 4:1). The combined fractions yielded a yellow oil that solidified to a light yellow solid (384 mg) in the desiccator. The substance was recrystallised from acetone (0.6 mL) and dried at 60°C for 2 h to yield colourless crystals (90 mg, 0.33 mmol, 16%). M.p.: 92–94°C; IR (KBr): ṽ [cm^-1^] = 2934 (-C-H), 1650 (C = O), 1461, 1383; ^1^H-NMR (400 MHz, DMSO-*d*_6_) δ (ppm) = 0.99 (t, *J* = 7.0 Hz, 3H, CH_3_), 1.15 (t, *J* = 7.1 Hz, 3H, CH_3_), 2.06 (br s, 2H, azepine-CH_2_), 2.15 (t, *J* = 6.4 Hz, 2H, azepine-CH_2_), 2.91 (br s, 2H, azepine-CH_2_), 3.24 (q, *J* = 7.1 Hz, 2H, CH_2_), 3.33 (q, *J* = 7.0 Hz, 2H, CH_2_, superimposed onto the HDO signal), 4.57 (s, 2H, azepine-*N*-CH_2_), 7.10–7.20 (m, 1H, ArH), 7.21–7.32 (m, 3H, ArH); ^13^C-NMR (DMSO-*d*_6_, 101 MHz): δ (ppm) = 12.9, 14.0 (CH_3_), 28.3, 29.1, 32.6, 39.7 (superimposed onto the DMSO-*d*_6_ signal), 40.5, 49.1 (CH_2_), 122.2, 125.7, 127.2, 129.2 (CH), 135.6, 142.9, 166.5, 171.8 (C); C_16_H_22_N_2_O_2_ (274.36); CHN: calc. C 70.04, H 8.08, N 10.21, found C 70.13, H 8.20, N 10.12; APCI-MS (ASAP, +, soft): m/z (%): 275 [M+H]^+^ (48), 202 [M-72]^+^ (98), 174 [M-100]^+^ (100); ESI-MS (DI, +): m/z (%): 571 [2M+Na]^+^ (9), 297 [M+Na]^+^ (100); HPLC (grad.): 99.7% at 254 nm, t_M+S_ = 9.3 min, t_M_ (DMSO) = 1.2 min, λ_max_ [nm] = 241; HPLC (isocr.): 99.9% at 254 nm, t_M+S_ = 3.9 min, t_M_ (DMSO) = 1.1 min (ACN/water 40:60), λ_max_ [nm] = 240.

*2-(2-Oxo-2*,*3*,*4*,*5-tetrahydro-1H-benzo[b]azepin-1-yl)-N-[2-(piperazin-1-yl)ethyl]acetamide dihydrochloride (2o)*. According to general procedure 6 from *tert*-butyl 4-{2-[2-(2-oxo-2,3,4,5-tetrahydro-1*H*-benzo[*b*]azepin-1-yl)acetamido]ethyl}piperazine-1-carboxylate (**2b**, 129 mg, 0.300 mmol) in trifluoroacetic acid (7.5 mL, 98 mmol) in dichloromethane (50 mL) (20 h). Precipitation as hydrochloride in 2-propanol (2 mL) with hydrogen chloride in 2-propanol (60 μL) and diethyl ether (10 mL) yielded a colourless solid (78 mg) that was recrystallised from ethanol (11.5 mL) and water (0.2 mL). After drying (60°C, 2 h), colourless crystals were obtained (14 mg, 0.035 mmol, 12%). Decomp.: starting at 153°C; IR (KBr): ṽ [cm^-1^] = [3346, 3210] (N-H), [2646, 2545, 2459] (NR_3_H^+^ / NR_2_H_2_^+^), [1673, 1643] (C = O), 1394, 1377; ^1^H-NMR (DMSO-*d*_6_, 500 MHz): δ (ppm) = 2.06 (br s, 2H, azepine-CH_2_), 2.16 (t, *J* = 7.0 Hz, 2H, azepine-CH_2_), 2.84 (br s, 2H, azepine-CH_2_), 3.19 (br s, CH_2_), 3.29 (br s, CH_2_), 3.46 (br s, CH_2_), 3.48 (br s, CH_2_), 3.68 (br s, CH_2_), 4.36 (s, 2H, azepine-*N*-CH_2_), 7.13–7.20 (m, 1H, ArH), 7.22–7.33 (m, 3H, ArH), 8.41 (br s, 1H, amide-NH), 9.74 (br s, 2H, NH_2_^+^), 11.77 (br s, 1H, NH^+^); The signals between 3.19 ppm and 3.68 ppm overlap with each other and the HDO signal and were assigned with help of H,C-HSQC; ^13^C-NMR (DMSO-*d*_6_, 126 MHz): δ (ppm) = 28.2, 29.1, 32.5, 33.3 (w), 47.9, 51.1, 54.7 (CH_2_), 122.5, 125.9, 127.3, 129.1 (CH), 135.2, 142.8, 168.7, 171.9 (C); The structure was confirmed by 2D-NMR spectroscopy (H,H-COSY, H,C-HSQC, H,C-HMBC, H,N-HMBC); A CH_2_ signal is not assignable in the ^13^C-1D spectra as it overlaps with the DMSO-*d*_6_ signal (H,C-HSQC cross peak 3.5; 39.7); C_18_H_28_Cl_2_N_4_O_2_ (403.35); HRMS (ESI, +): m/z [M+H]^+^ calc. 331.21285, found 331.21303; APCI-MS (ASAP, -): m/z (%): 329 [M-H]^-^ (100); ESI-MS (DI, +): m/z (%): 353 [M+Na]^+^ (14), 331 [M+H]^+^ (100); HPLC (isocr.): 100.0% at 254 nm, t_M+S_ = 5.7 min, t_M_ (DMSO) = 1.3 min (ACN/buffer pH 2.7 10:90), λ_max_ [nm] = 237.

*N-(2-Aminoethyl)-2-(2-oxo-2*,*3*,*4*,*5-tetrahydro-1H-benzo[b]azepin-1-yl)acetamide hydrochloride (2p)*. According to general procedure 6 from *tert*-butyl {2-[2-(2-oxo-2,3,4,5-tetrahydro-1*H*-benzo[*b*]azepin-1-yl)acetamido]ethyl}carbamate (**2c**, 108 mg, 0.299 mmol) in trifluoroacetic acid (7.5 mL, 98 mmol) in dichloromethane (50 mL) (20 h). Precipitation as hydrochloride in 2-propanol (2 mL) with hydrogen chloride in 2-propanol (60 μL) and diethyl ether (10 mL) yielded a light beige solid (47 mg, 0.16 mmol, 53%). M.p.: 218–220°C; IR (KBr): ṽ [cm^-1^] = 3359 (N-H), 2950 (NRH_3_^+^ / -C-H), [1673, 1642] (C = O), 1489, 1458; ^1^H-NMR (DMSO-*d*_6_, 500 MHz): δ (ppm) = 2.06 (br s, 2H, azepine-CH_2_), 2.15 (t, *J* = 6.9 Hz, 2H, azepine-CH_2_), 2.66–3.03 (m, 4H, 2x CH_2_), 3.31 (td, *J* = 6.3 / 5.9 Hz, 2H, amide-*N*-CH_2_), 4.35 (s, 2H, azepine-*N*-CH_2_), 7.13–7.19 (m, 1H, ArH), 7.23–7.32 (m, 3H, ArH), 8.06 (br s, 3H, NH_3_^+^), 8.36 (t, *J* = 5.8 Hz, 1H, NH); ^13^C-NMR (DMSO-*d*_6_, 126 MHz): δ (ppm) = 28.2, 29.0, 32.5, 36.3, 38.3, 50.8 (CH_2_), 122.5, 125.9, 127.3, 129.1 (CH), 135.3, 142.8, 168.8, 171.9 (C); C_14_H_20_ClN_3_O_2_ (297.78); CHN: calc. C 56.47, H 6.77, N 14.11, found C 56.41, H 6.49, N 13.81; APCI-MS (ASAP,+): m/z (%): 262 [M+H]^+^ (100), 244 [M-17]^+^ (25), 202 [M-59]^+^ (28), 174 [M-87]^+^ (80), 146 [M-115]^+^ (62); ESI-MS (DI, +): m/z (%): 284 [M+Na]^+^ (82), 262 [M+H]^+^ (93); HPLC (isocr.): 100.0% at 254 nm, t_M+S_ = 7.2 min, t_M_ (DMSO) = 1.3 min (ACN/buffer pH 2.7 10:90), λ_max_ [nm] = 237.

*1-[2-Oxo-2-(piperazin-1-yl)ethyl]-1*,*3*,*4*,*5-tetrahydro-2H-benzo[b]azepin-2-one hydrochloride (2q)*. According to general procedure 6 from *tert*-butyl 4-[2-(2-oxo-2,3,4,5-tetrahydro-1*H*-benzo[*b*]azepin-1-yl)acetyl]piperazine-1-carboxylate (**2d**, 310 mg, 0.800 mmol) in trifluoroacetic acid (11 mL, 0.14 mol) and dichloromethane (30 mL) (24 h). Precipitation as hydrochloride in 2-propanol (2 mL) with hydrogen chloride in 2-propanol (160 μL) and *n*-hexane (10 mL). The gum was crystallised upon addition of *n*-hexane (5 mL) and ethanol (0.5 mL). After drying (60°C, 2 h), a colourless solid was obtained (149 mg, 0.460 mmol, 58%). Decomp.: 250–251°C; IR (KBr): ṽ [cm^-1^] = 2936 (-C-H), [2721, 2619, 2480] (NR_2_H_2_^+^), 1660 (C = O), 1457, 1388, 1246; ^1^H-NMR (DMSO-*d*_6_, 600 MHz): δ (ppm) = 2.06 (br s, 2H, azepine-CH_2_), 2.16 (t, *J* = 6.9 Hz, 2H, azepine-CH_2_), 2.91 (br s, 2H, azepine-CH_2_), 3.04 (t, *J* = 5.2 Hz, 2H, *N*-CH_2_), 3.13 (t, *J* = 5.3 Hz, 2H, *N*-CH_2_), 3.62–3.68 (m, 2H, *N*-CH_2_), 3.73 (t, *J* = 5.4 Hz, 2H, *N*-CH_2_), 4.69 (s, 2H, azepine-*N*-CH_2_), 7.16 (td, *J* = 7.3 / 1.4 Hz, 1H, ArH), 7.22–7.31 (m, 3H, ArH), 9.34 (br s, 2H, NH_2_^+^, complete H/D exchange); ^13^C-NMR (DMSO-d_6_, 151 MHz): δ (ppm) = 28.3, 32.5, 38.1, 41.1, 42.3, 42.5, 49.0 (CH_2_), 122.4, 125.8, 127.2, 129.1 (CH), 135.5, 142.6, 166.5, 171.8 (C), 29.0 (no signal in the DEPT135 experiment; t, ^1^*J* = 131 Hz in the gated-decoupled experiment); C_16_H_22_ClN_3_O_2_ (323,82); CHN: calc. C 59.35, H 6.85, N 12.98, found C 59.05, H 7.10, N 12.99; APCI-MS (ASAP, +): m/z (%): 288 [M+H]^+^ (10), 202 [M-85]^+^ (30), 174 [M-113]^+^ (100), 146 [M-141]^+^ (58); ESI-MS (DI, +): m/z (%): 310 [M+Na]^+^ (50), 288 [M+H]^+^ (100); HPLC (isocr.): 99.9% at 254 nm, t_M+S_ = 7.9 min, t_M_ (DMSO) = 1.2 min (ACN/buffer pH 2.7 10:90), λ_max_ [nm] = 237.

*N-[2-(Methylamino)ethyl]-2-(2-oxo-2*,*3*,*4*,*5-tetrahydro-1H-benzo[b]azepin-1-yl)acetamide hydrochloride (2r)*. According to general procedure 6 from *tert*-butyl methyl-{2-[2-(2-oxo-2,3,4,5-tetrahydro-1*H*-benzo[*b*]azepin-1-yl)acetamido]ethyl}carbamate (**2e**, 150 mg, 0.400 mmol) in trifluoroacetic acid (10.0 mL, 131 mmol) and dichloromethane (38 mL) (22 h). Precipitation as hydrochloride in 2-propanol (1 mL) with hydrogen chloride in 2-propanol (80 μL) and petroleum ether (20 mL). After recrystallisation from ethanol (0.5 mL) and drying (60°C, 2 h), a colourless solid was obtained (30 mg, 0.096 mmol, 24%). Decomp.: 153–154°C; IR (KBr): ṽ [cm^-1^] = 3226 (NH), [2739, 2726] (NR_2_H_2_^+^), [1667, 1659] (C = O), 1493, 1457, 1376, 1203, 1167, 1146; ^1^H-NMR (DMSO-*d*_6_, 500 MHz): δ (ppm) = 2.07 (br s, 2H, azepine-CH_2_), 2.15 (t, *J* = 7.0 Hz, 2H, azepine-CH_2_), 2.55 (s, 3H, *N*-CH_3_), 2.83 (br s, 2H, azepine-CH_2_), 2.94 (t, *J* = 6.2 Hz, 2H, *N*-CH_2_), 3.34 (t, *J* = 6.1 Hz, 2H, *N*-CH_2_), 4.35 (s, 2H, azepine-*N*-CH_2_), 7.17 (ddd, *J* = 7.9 / 6.1 / 2.4 Hz, 1H, ArH), 7.23–7.32 (m, 3H, ArH), 8.32 (t, *J* = 5.9 Hz, 1H, NH, partial H/D exchange), 8.62 (br s, 2H, NH_2_^+^, complete H/D exchange); ^13^C-NMR (DMSO-*d*_6_, 126 MHz): δ (ppm) = 32.4 (CH_3_), 28.2, 29.0, 32.5, 35.0, 47.6, 50.8 (CH_2_), 122.5, 125.9, 127.3, 129.1 (CH), 135.3, 142.8, 168.9, 171.9 (C); C_15_H_22_ClN_3_O_2_ (311.81); HRMS (ESI, +): m/z [M+H]^+^ calc. 276.17065, found 276.17087; APCI-MS (ASAP, +): m/z (%): 276 [M+H]^+^ (100), 245 [M-30]^+^ (29), 202 [M-73]^+^ (28), 174 [M-101]^+^ (99), 146 [M-129]^+^ (79); ESI-MS (DI, +): m/z (%): 298 [M+Na]^+^ (33), 276 [M+H]^+^ (100); HPLC (isocr.): 98.9% at 254 nm, t_M+S_ = 6.2 min, t_M_ (DMSO) = 1.3 min (ACN/buffer pH 2.7 30:70), λ_max_ [nm] = 237.

*N-(4-Aminobutyl)-2-(2-oxo-2*,*3*,*4*,*5-tetrahydro-1H-benzo[b]azepin-1-yl)acetamide hydrochloride (2s)*. According to general procedure 6 from *tert*-butyl {4-[2-(2-oxo-2,3,4,5-tetrahydro-1*H*-benzo[*b*]azepin-1-yl)acetamido]butyl}carbamate (**2f**, 273 mg, 0.700 mmol) in trifluoroacetic acid (17.5 mL, 229 mmol) and dichloromethane (28 mL) (22 h). Precipitation as hydrochloride in 2-propanol (3 mL) with hydrogen chloride in 2-propanol (140 μL) and petroleum ether (5 mL), treatment with *n*-hexane (10 mL) and drying (60°C, 2 h) yielded a light beige solid (68 mg, 0.21 mmol, 30%). Decomp.: starting at 141°C; IR (KBr): ṽ [cm^-1^] = [3447,3329, 3271] (N-H), 2944 (-C-H), [1657, 1630] (C = O), 1597, 1381, 766; ^1^H-NMR (DMSO-*d*_6_, 500 MHz): δ (ppm) = 1.36–1.47 (m, 2H, *C*-CH_2_), 1.47–1.59 (m, 2H, *C*-CH_2_), 2.02 (br s, 2H, azepine-CH_2_), 2.15 (t, *J* = 7.0 Hz, 2H, azepine-CH_2_), 2.75 (t, *J* = 7.4 Hz, 2H, *N*-CH_2_), 2.84 (br s, 2H, azepine-CH_2_), 3.05 (q, *J* = 6.5 Hz, 2H, *N*-CH_2_), 4.31 (s, 2H, azepine-*N*-CH_2_), 7.16 (td, *J* = 7.2 / 1.4 Hz, 1H, ArH), 7.22–7.33 (m, 3H, ArH), 7.90 (br s, 3H, NH_3_^+^, complete H/D exchange), 8.12 (t, *J* = 5.8 Hz, 1H, NH); ^13^C-NMR (DMSO-*d*_6_, 126 MHz): δ (ppm) = 24.3, 25.9, 28.2, 29.0, 32.5, 37.8, 38.3, 50.6 (CH_2_), 122.3, 125.8, 127.3, 129.1 (CH), 135.3, 142.8, 167.9, 171.8 (C); C_16_H_24_ClN_3_O_2_ (325.84); HRMS (ESI, +): m/z [M+H]^+^ calc. 290.18630, found 290.18665; APCI-MS (ASAP, +): m/z (%): 290 [M+H]^+^ (100), 202 [M-87]^+^ (47), 174 [M-115]^+^ (75), 146 [M-143]^+^ (29); ESI-MS (DI, +): m/z (%): 312 [M+Na]^+^ (38), 290 [M+H]^+^ (100); HPLC (isocr.): 100.0% at 254 nm, t_M+S_ = 8.0 min, t_M_ (DMSO) = 1.1 min (ACN/buffer pH 2.7 10:90), λ_max_ [nm] = 237.

*N-(2-Morpholinoethyl)-2-(2-oxo-2*,*3*,*4*,*5-tetrahydro-1H-benzo[b]azepin-1-yl)acetamide hydrochloride (2t)*. According to general procedure 4 from 2-(2-oxo-2,3,4,5-tetrahydro-1*H*-benzo[*b*]azepin-1-yl)acetic acid (**13**, 110 mg, 0.500 mmol), HOBt (81 mg, 0.60 mmol), EDCl (125 mg, 0.650 mmol), DIPEA (104 μL, 0.600 mmol) and 4-(2-aminoethyl)morpholine (79 μL, 0.60 mmol) in DMF (4 mL) (22 h). Water (20 mL) was added and the mixture was extracted according to general procedure 5 (4x 20 mL water and 4x 20 mL dichloromethane). After removal of the solvent by distillation under reduced pressure from the combined organic phases the obtained brown oil was purified by column chromatography (silica gel, eluent ethyl acetate/ethanol/trimethylamine 10:2:1). The obtained orange oil was precipitated as hydrochloride in 2-propanol (1 mL) with hydrogen chloride in 2-propanol (100 μL) and petroleum ether (2 mL). A light yellow solid was obtained after crystallisation by addition of petroleum ether (5 mL) and ethanol (0.4 mL) and drying (60°C, 2 h) (30 mg, 0.082 mmol, 16%). Decomp.: 191–192°C; IR (KBr): ṽ [cm^-1^] = 3246 (N-H), 2586 (NR_3_H^+^), [1689, 1658] (C = O), 1547, 1460, 1451, 1398, 1243, 1101; ^1^H-NMR (DMSO-*d*_6_, 500 MHz): δ (ppm) = 2.06 (br s, 2H, azepine-CH_2_), 2.16 (t, *J* = 7.0 Hz, 2H, azepine-CH_2_), 2.84 (br s, 2H, azepine-CH_2_), 3.01–3.11 (m, 2H, CH_2_), 3.15 (q, *J* = 6.0 Hz, 2H, CH_2_), 3.42 (d, *J* = 12.3 Hz, 2H, CH_2_), 3.49 (q, *J* = 6.2 Hz, 2H, CH_2_), 3.80 (t, *J* = 12.3 Hz, 2H, CH_2_), 3.93 (dd, *J* = 12.6 / 2.8 Hz, 2H, CH_2_),, 4.35 (s, 2H, azepine-*N*-CH_2_), 7.14–7.19 (m, 1H, ArH), 7.23–7.32 (m, 3H, ArH), 8.45 (t, *J* = 5.8 Hz, 1H, amide-NH, partial H/D exchange), 10.92 (br s, 1H, NH^+^, complete H/D exchange); ^13^C-NMR (DMSO-*d*_6_, 126 MHz): δ (ppm) = 28.2, 29.0, 32.5, 33.1, 50.9, 51.0, 54.9, 63.0 (CH_2_), 122.5, 125.9, 127.3, 129.1 (CH), 135.3, 142.8, 168.7, 171.9 (C); C_18_H_26_ClN_3_O_3_ (367.87); HRMS (ESI, +): m/z [M+H]^+^ calc. 332.19687, found 332.19719; APCI-MS (ASAP, +): m/z (%): 376 [M+2Na-H]^+^ (2), 332 [M+H]^+^ (100); ESI-MS (DI, +): m/z (%): 354 [M+Na]^+^ (66), 332 [M+H]^+^ (100); HPLC (isocr.): 98.5% at 254 nm, t_M+S_ = 3.8 min, t_M_ (DMSO) = 1.2 min (ACN/buffer pH 2.7 15:85), λ_max_ [nm] = 237.

*N-[2-(4-Methylpiperazin-1-yl)ethyl]-2-(2-oxo-2*,*3*,*4*,*5-tetrahydro-1H-benzo[b]azepin-1-yl)acetamide dihydrochloride (2u)*. According to general procedure 4 from 2-(2-oxo-2,3,4,5-tetrahydro-1*H*-benzo[*b*]azepin-1-yl)acetic acid (**13**, 110 mg, 0.500 mmol), HOBt (81 mg, 0.60 mmol), EDCl (125 mg, 0.650 mmol), DIPEA (104 μL, 0.600 mmol) and 1-(2-aminoethyl)-4-methylpiperazine (86 mg, 0.60 mmol) in DMF (3 mL) (26 h). Water (20 mL) was added and the mixture was extracted according to general procedure 5 (4x 20 mL water and 4x 20 mL ethyl acetate). Sodium hydroxide solution was added dropwise to the combined aqueous phases until pH 10 was obtained. Extraction with ethyl acetate (3x 20 mL) and removal of the solvent by distillation under reduced pressure from the combined organic phases yielded a yellow oil. Precipitation as hydrochloride in 2-propanol (1 mL) with hydrogen chloride in 2-propanol (200 μL) and petroleum ether (5 mL) yielded a yellow solid. Recrystallisation from ethyl acetate (0.6 mL), ethanol (3.6 mL) and water (11 drops) and further recrystallisation from ethanol (2.0 mL) and water (6 drops) and drying (60°C, 4 h) lead to a colourless solid (46 mg, 0.11 mmol, 22%). Decomp.: starting at 240°C; IR (KBr): ṽ [cm^-1^] = 3315 (N-H), 2351 (NR_3_H^+^), [1670, 1636] (C = O), 1538, 1459; ^1^H-NMR (DMSO-*d*_6_, 600 MHz): δ (ppm) = 2.06 (br s, 2H, azepine-CH_2_), 2.16 (t, *J* = 7.0 Hz, 2H, azepine-CH_2_), 2.81 (br s, 3H, CH_3_), 2.84 (br s, CH_2_), 3.14 (br s, CH_2_), 3.37 (br s, CH_2_), 3.46 (br s, CH_2_), 3.63 (br s, CH_2_), 4.35 (s, 2H, azepine-*N*-CH_2_), 7.15–7.19 (m, 1H, ArH), 7.24–7.32 (m, 3H, ArH), 8.37 (br s, 1H, amide-NH, partial H/D exchange), 11.65 (br s, 2H, 2x NH^+^, complete H/D exchange); The signals between 2.81 ppm and 3.63 ppm overlap with each other and the HDO signal and were assigned with help of H,C-HSQC; ^13^C-NMR (DMSO-*d*_6_, 151 MHz): δ (ppm) = 42.0 (br, w) (CH_3_), 28.2, 29.1, 32.5, 33.6 (br, w), 48.4 (br, w), 49.7 (br, w), 51.1, 54.9 (br, w) (CH_2_), 122.5, 125.9, 127.3, 129.1 (CH), 135.2, 142.8, 168.7, 171.9 (C); The structure was confirmed by 2D-NMR spectroscopy (H,C-HSQC); C_19_H_30_Cl_2_N_4_O_2_ (417.37); CHN: calc. C 54.68, H 7.25, N 13.42, found C 54.50, H 7.19, N 13.02; APCI-MS (ASAP, +): m/z (%): 345 [M+H]^+^ (100); ESI-MS (DI, +): m/z (%): 367 [M+Na]^+^ (8), 345 [M+H]^+^ (100); HPLC (isocr.): 97.2% at 254 nm, t_M+S_ = 5.7 min, t_M_ (DMSO) = 1.3 min (ACN/buffer pH 2.7 10:90), λ_max_ [nm] = 237.

*1-[2-(4-Methylpiperazin-1-yl)-2-oxoethyl]-1*,*3*,*4*,*5-tetrahydro-2H-benzo[b]azepin-2-one hydrochloride (2v)*. According to general procedure 4 from 2-(2-oxo-2,3,4,5-tetrahydro-1*H*-benzo[*b*]azepin-1-yl)acetic acid (**13**, 164 mg, 0.750 mmol), HOBt (122 mg, 0.900 mmol), EDCl (187 mg, 0.975 mmol), DIPEA (156 μL, 0.900 mmol) and 1-methylpiperazine (100 μL, 0.900 mmol) in DMF (3 mL) (21 h). Cold aqueous sodium chloride solution (1 M, 20 mL) was added and the mixture was extracted according to general procedure 5 (2x 20 mL 1 M cold aqueous sodium chloride solution and 3x 30 mL ethyl acetate). After solvent evaporation, the brown residue was precipitated as hydrochloride in 2-propanol (1 mL) with hydrogen chloride in 2-propanol (200 μL) and *n*-hexane (10 mL). Upon further addition of 2-propanol (2 mL), a light beige solid was obtained and dried (60°C, 2 h) (165 mg, 0.488 mmol, 65%). Decomp.: 227–228°C; IR (KBr): ṽ [cm^-1^] = 3454 (N-H), 2388 (NR_3_H^+^), [1659, 1628] (C = O), 1455, 1391, 1251, 769; ^1^H-NMR (DMSO-*d*_6_, 600 MHz): δ (ppm) = 2.07 (br s, 2H, azepine-CH_2_), 2.16 (t, *J* = 7.0 Hz, 2H, azepine-CH_2_), 2.76 (s, 3H, *N*-CH_3_), 2.92 (br s, *N*-CH_2_), 3.02–3.08 (m, 2H, *N*-CH_2_), 3.38–3.43 (m, *N*-CH_2_), 3.52 (t, *J* = 13.0 Hz, 1H, *N*-CH_2_), 4.09 (d, *J* = 14.5 Hz, 1H, *N*-CH_2_), 4.35 (d, *J* = 14.2 Hz, 1H, *N*-CH_2_), 4.66 (d, *J* = 16.6 Hz, 1H, azepine-*N*-CH_2_, part B of an AB system), 4.76 (d, *J* = 16.8 Hz, 1H, azepine-*N*-CH_2_, part A of an AB system), 7.16 (td, *J* = 7.3 / 1.3 Hz, 1H, ArH), 7.21–7.31 (m, 3H, ArH), 11.12 (br s, 1H, NH^+^, near complete H/D exchange); If an integral is not reported, the assignment was difficult due to overlapping with the HDO signal or with a very broad signal; ^13^C-NMR (DMSO-d_6_, 151 MHz): δ (ppm) = 41.8 (CH_3_), 28.3, 32.5, 38.2, 41.0, 49.0, 51.8, 52.0 (CH_2_), 122.3, 125.8, 127.2, 129.2 (CH), 135.6, 142.5, 166.5, 171.8 (C), 29.0 (no signal in the DEPT135 experiment, although a CH_2_ group is expected, cf. **2q** for further experiments); The structure was confirmed by 2D-NMR spectroscopy (H,H-COSY, H,C-HSQC); A signal for an azepine CH_2_ expected at approx. 2.8 ppm is not assignable; C_17_H_24_ClN_3_O_2_ (337.85); HRMS (ESI, +): m/z [M+H]^+^ calc. 302.18630, found 302.18658; APCI-MS (ASAP, +): m/z (%): 302 [M+H]^+^ (89), 202 [M-99]^+^ (68), 174 [M-127]^+^ (100), 146 [M-155]^+^ (26); ESI-MS (DI, +): m/z (%): 324 [M+Na]^+^ (100); HPLC (isocr.): 100.0% at 254 nm, t_M+S_ = 7.3 min, t_M_ (DMSO) = 1.2 min (ACN/buffer pH 2.7 10:90), λ_max_ [nm] = 237.

*tert-Butyl 4-{2-[2-(3*,*3-dimethyl-2-oxo-2*,*3*,*4*,*5-tetrahydro-1H-benzo[b]azepin-1-yl)acetamido]ethyl}piperazine-1-carboxylate (3b)*. According to general procedure 4 from 2-(3,3-dimethyl-2-oxo-2,3,4,5-tetrahydro-1*H*-benzo[*b*]azepin-1-yl)acetic acid (**16**, 336 mg, 1.36 mmol), HOBt (220 mg, 1.63 mmol), EDCl (360 mg, 1.88 mmol), DIPEA (282 μL, 1.63 mmol) and *tert*-butyl 4-(2-aminoethyl)piperazine-1-carboxylate (343 mg, 1.50 mmol) in DMF (4 mL) (24 h). Ethyl acetate (30 mL) was added to the mixture and washed with an aqueous sodium hydroxide solution (1 mM, 6x 15 mL). The brown residue after evaporation of the organic phase was purified by column chromatography (silica gel, eluent ethyl acetate/triethylamine 10:1). Drying (60°C, 4 h; 40°C, 6 h) led to a pale yellow solid (184 mg, 0.427 mmol, 30%). Decomp.: starting at 62°C; IR (KBr): ṽ [cm^-1^] = 3328 (N-H), 1696 (C = O), 1459, 1421, 1364, 1244, 1171; ^1^H-NMR (DMSO-*d*_6_, 500 MHz): δ (ppm) = 0.83 (s, 6H, 2x CH_3_), 1.39 (s, 9H, 3x CH_3_), 1.94 (t, *J* = 7.1 Hz, 2H, azepine-CH_2_), 2.24–2.40 (m = 2x t superimposed, 6H, *N*-CH_2_), 2.84 (br s, 2H, azepine-CH_2_), 3.18 (q, *J* = 6.6 Hz, 2H, amide-*N*-CH_2_), 3.27 (t, *J* = 5.0 Hz, 4H, 2x carbamate-*N*-CH_2_), 4.32 (s, 2H, azepine-*N*-CH_2_), 7.12 (td, *J* = 7.4 / 1.3 Hz, 1H, ArH), 7.17 (dd, *J* = 7.9 / 1.2 Hz, 1H, ArH), 7.23 (dd, *J* = 7.5 / 1.6 Hz, 1H, ArH), 7.24–7.28 (m, 1H, ArH), 7.84 (t, *J* = 5.6 Hz, 1H, NH); ^13^C-NMR (DMSO-*d*_6_, 126 MHz): δ (ppm) = 28.0 (3C), 28.1 (2C, br) (CH_3_), 29.6, 36.0, 42.5 (br, w), 43.6 (br, w), 45.0, 52.3, 52.7, 56.8 (CH_2_), 122.0, 125.4, 127.3, 128.4 (CH), 41.3, 78.6, 135.6, 143.4, 153.7, 168.1, 175.7 (C); C_25_H_38_N_4_O_4_ (458.60); CHN: calc. C 65.48, H 8.35, N 12.22, found C 65.63, H 8.69, N 12.27; APCI-MS (ASAP, +, soft): m/z (%): 459 [M+H]^+^ (100); ESI-MS (DI, +): m/z (%): 497 [M+K]^+^ (6), 481 [M+Na]^+^ (100); HPLC (isocr.): 98.5% at 254 nm, t_M+S_ = 5.0 min, t_M_ (DMSO) = 1.1 min (ACN/buffer pH 2.7 35:65), λ_max_ [nm] = 242.

*tert-Butyl {2-[2-(3*,*3-dimethyl-2-oxo-2*,*3*,*4*,*5-tetrahydro-1H-benzo[b]azepin-1-yl)acetamido]ethyl}(methyl)carbamate (3c)*. According to general procedure 4 from 2-(3,3-dimethyl-2-oxo-2,3,4,5-tetrahydro-1*H*-benzo[*b*]azepin-1-yl)acetic acid (**16**, 111 mg, 0.450 mmol), HOBt (73 mg, 0.54 mmol), EDCl (112 mg, 0.585 mmol), DIPEA (92 μL, 0.54 mmol) and *N*-(*tert*-butoxycarbonyl)-*N*-methylethane-1,2-diamine (94 mg, 0.54 mmol) in DMF (3 mL) (24 h). Cold aqueous sodium chloride solution (1 M, 20 mL) was added and the mixture was extracted with ethyl acetate (3x 30 mL). The remaining residue crystallised in the desiccator, was washed with *n*-hexane (5 mL) and ethyl acetate (0.3 mL) and dried (60°C, 2 h) to yield a white solid (124 mg, 0.307 mmol, 68%). M.p.: 110–111°C; IR (KBr): ṽ [cm^-1^] = 3256 (N-H), [1689, 1643] (C = O), 1389, 1360, 1168; ^1^H-NMR (DMSO-*d*_6_, 600 MHz): δ (ppm) = 0.83 (br s, 6H, 2x CH_3_), 1.36 and 1.37 (br, 9H, 3x CH_3_, peak split due to *N*-Boc rotamers), 1.94 (t, *J* = 7.1 Hz, 2H, azepine-CH_2_), 2.75 and 2.77 (br, 3H, *N*-CH_3_, peak split due to *N*-Boc rotamers), 2.84 (br s, 2H, azepine-CH_2_), 3.12–3.23 (m, 4H, 2x *N*-CH_2_), 4.29 and 4.31 (br, 2H, azepine-*N*-CH_2_, peak split due to *N*-Boc rotamers), 7.12 (td, *J* = 7.4 / 1.2 Hz, 1H, ArH), 7.17 (br d, *J* = 8.0 Hz, 1H, ArH), 7.22 (dd, *J* = 7.5 / 1.6 Hz, 1H, ArH), 7.25 (ddd, *J* = 8.0 / 7.2 / 1.6 Hz, 1H, ArH), 8.02 and 8.08 (br, 1H, NH, peak split due to *N*-Boc rotamers); ^13^C-NMR (DMSO-d_6_, 151 MHz): δ (ppm) = 28.0 (3C), 28.1 (br, 2C), [34.2 (w), 34.5 (w) split due to *N*-Boc rotamers] (CH_3_), 29.6, [36.6 (w), 37.0 (w) split due to *N*-Boc rotamers], 45.0, [47.2 (w), 47.7 (w) split due to *N*-Boc rotamers], 52.7 (CH_2_), 122.0, 125.4, 127.3, 128.4 (CH), 41.3, 78.3, [135.6 (w), 135.7 (w) split due to *N*-Boc rotamers], 143.5 (w), [154.6 (w), 154.9 (w) split due to *N*-Boc rotamers], 168.3, 175.6 (C); The structure was confirmed by 2D-NMR spectroscopy (H,H-COSY, H,C-HSQC); C_22_H_33_N_3_O_4_ (403.52); CHN: calc. C 65.48, H 8.24, N 10.41, found C 65.44, H 8.30, N 10.49; APCI-MS (ASAP, +): m/z (%): 330 [M-73]^+^ (8), 304 [M-99]^+^ (100), 230 [M-173]^+^ (63), 202 [M-201]^+^ (14), 174 [M-229]^+^ (12); ESI-MS (DI, +): m/z (%): 426 [M+Na]^+^ (100); HPLC (grad.): 99.1% at 254 nm, t_M+S_ = 11.1 min, t_M_ (DMSO) = 1.3 min, λ_max_ [nm] = 242; HPLC (isocr.): 99.7% at 254 nm, t_M+S_ = 3.1 min, t_M_ (DMSO) = 1.0 min (ACN/water 60:40), λ_max_ [nm] = 242.

*2-(3*,*3-Dimethyl-2-oxo-2*,*3*,*4*,*5-tetrahydro-1H-benzo[b]azepin-1-yl)-N-[2-(piperazin-1-yl)ethyl]acetamide dihydrochloride (3d)*. According to general procedure 6 from *tert*-butyl 4-{2-[2-(3,3-dimethyl-2-oxo-2,3,4,5-tetrahydro-1*H*-benzo[*b*]azepin-1-yl)acetamido]ethyl}piperazine-1-carboxylate (**3b**, 229 mg, 0.500 mmol) in trifluoroacetic acid (6.7 mL, 88 mmol) and dichloromethane (25 mL) (18 h). After removal of the solvent by distillation under reduced pressure, the yellowish residue was mixed with sodium hydroxide solution (40 mL, 1 mM), alkalised to pH 10 with sodium hydroxide solution (8% m/m) and extracted with ethyl acetate (2x 10 mL). A yellow solid (0.13 g) was obtained after solvent distillation. Precipitation as hydrochloride in 2-propanol (2 mL) with hydrogen chloride in 2-propanol (10 drops) and *n*-hexane (10 mL) overnight at room temperature. The solid was filtrated and the mixture dried at 60°C under reduced pressure for 4 h. An off-white solid was obtained (99 mg, 0.23 mmol, 46%). Decomp.: starting at 260°C; IR (KBr): ṽ [cm^-1^] = 3235 (N-H), 2929 (-C-H), [2702, 2624] (NR_3_H^+^ / NR_2_H_2_^+^), [1682, 1637] (C = O), 1392, 1375, 1365; ^1^H-NMR (DMSO-*d*_6_, 500 MHz): δ (ppm) = 0.83 (s, 6H, 2x CH_3_), 1.94 (t, *J* = 7.1 Hz, 2H, azepine-CH_2_), 2.84 (br s, 2H, azepine-CH_2_), 3.21 (br s, *N*-CH_2_), 3.29 (br s, *N*-CH_2_), 3.46 (br s, *N*-CH_2_), 3.50 (br s, *N*-CH_2_), 3.71 (br s, *N*-CH_2_), 4.38 (s, 2H, azepine-*N*-CH_2_), 7.13 (td, *J* = 7.2 / 1.3 Hz, 1H, ArH), 7.18–7.31 (m, 3H, ArH), 8.39 (t, *J* = 6.1 Hz, 1H, amide-NH, partial H/D exchange), 9.84 (br s, 2H, NH_2_^+^, near complete H/D exchange), 11.83 (br s, 1H, NH^+^, complete H/D exchange); The signals between 3.21 ppm and 3.71 ppm overlap with each other and the HDO signal and were assigned with help of H,C-HSQC; ^13^C-NMR (DMSO-*d*_6_, 126 MHz): δ (ppm) = 28.1 (br) (2C) (CH_3_), 29.6, 33.2, 39.6 (superimposed onto the DMSO-*d*_6_ signal), 45.0, 47.8, 53.2, 54.7 (CH_2_), 122.2, 125.4, 127.4, 128.3 (CH), 41.3, 135.5, 143.5, 168.9, 175.7 (C); The structure was confirmed by 2D-NMR spectroscopy (H,H-COSY, H,C-HSQC); C_20_H_32_Cl_2_N_4_O_2_ (431.40); CHN: calc. C 55.68, H 7.48, N 12.99, found C 55.27, H 7.41, N 12.69; APCI-MS (ASAP, +): m/z (%): 359 [M+H]^+^ (100); ESI-MS (DI, +): m/z (%): 381 [M+Na]^+^ (7), 359 [M+H]^+^ (100); HPLC (isocr.): 98.8% at 254 nm, t_M+S_ = 4.5 min, t_M_ (DMSO) = 1.1 min (ACN/buffer pH 2.7 20:80), λ_max_ [nm] = 241.

*2-(3*,*3-Dimethyl-2-oxo-2*,*3*,*4*,*5-tetrahydro-1H-benzo[b]azepin-1-yl)-N-[2-(methylamino)ethyl]acetamide hydrochloride (3e)*. According to general procedure 6 from *tert*-butyl {2-[2-(3,3-dimethyl-2-oxo-2,3,4,5-tetrahydro-1*H*-benzo[*b*]azepin-1-yl)acetamido]ethyl}(methyl)carbamate (**3c**, 95 mg, 0.24 mmol) in trifluoroacetic acid (6.0 mL, 78 mmol) (43 h). Solvent evaporation, precipitation as hydrochloride in 2-propanol (2 mL) with hydrogen chloride in 2-propanol (50 μL) and *n*-hexane (10 mL) overnight at room temperature. Solvent removal by distillation. The residue was washed with acetone (5 mL) and dried (60°C, 2 h) to yield a white flaky precipitate (29 mg, 0.085 mmol, 36%). Decomp.: starting at 96°C; IR (KBr): ṽ [cm^-1^] = 3453, 3257 (N-H), 2962 (-C-H), 2775 (NR_2_H_2_^+^), 1649 (C = O), 1595, 1459; ^1^H-NMR (DMSO-*d*_6_, 500 MHz): δ (ppm) = 0.83 (br s, 6H, 2x CH_3_), 1.94 (t, *J* = 7.1 Hz, 2H, azepine-CH_2_), 2.54 (s, 3H, *N*-CH_3_), 2.85 (br s, 2H, azepine-CH_2_), 2.95 (t, *J* = 6.2 Hz, 2H, *N*-CH_2_), 3.36 (dt, *J* = 6.1 Hz, 2H, *N*-CH_2_, superimposed onto the HDO signal), 4.36 (s, 2H, azepine-*N*-CH_2_), 7.13 (td, *J* = 7.4 / 1.4 Hz, 1H, ArH), 7.18–7.31 (m, 3H, ArH), 8.31 (t, *J* = 5.6 Hz, 1H, NH, near complete H/D exchange), 8.71 (br s, 2H, NH_2_^+^, complete H/D exchange); ^13^C-NMR (DMSO-d_6_, 126 MHz): δ (ppm) = 28.1 (2C, br), 32.3 (CH_3_), 29.5, 34.9, 45.0, 47.6, 53.0 (CH_2_), 122.2, 125.4, 127.3, 128.4 (CH), 41.3, 135.6, 143.5, 169.1, 175.8 (C); C_17_H_26_ClN_3_O_2_ (339.86); HRMS (ESI, +): m/z [M+H]^+^ calc. 304.20195, found 304.20220; APCI-MS (ASAP, +, soft): m/z (%): 304 [M+H]^+^ (100), 248 [M-55]^+^ (10); 230 [M-73]^+^ (14); ESI-MS (DI, +): m/z (%): 326 [M+Na]^+^ (21), 304 [M+H]^+^ (77), 230 [M-73]^+^ (100); HPLC (isocr.): 99.9% at 254 nm, t_M+S_ = 3.3 min, t_M_ (DMSO) = 1.1 min (ACN/buffer pH 2.7 25:75), λ_max_ [nm] = 241.

*tert-Butyl 4-{2-[2-(6-oxo-6*,*7-dihydro-5H-dibenzo[b*,*d]azepin-5-yl)acetamido]ethyl}piperazine-1-carboxylate (4b)*. According to general procedure 4 from 2-(6-oxo-6,7-dihydro-5*H*-dibenzo[*b*,*d*]azepin-5-yl)acetic acid (**19**, 348 mg, 1.30 mmol), HOBt (211 mg, 1.56 mmol), EDCl (326 mg, 1.70 mmol), DIPEA (270 μL, 1.56 mmol) and *tert*-butyl 4-(2-aminoethyl)piperazine-1-carboxylate (328 mg, 1.43 mmol) in DMF (4 mL) (95 h). Ethyl acetate (60 mL) was added to the mixture and washed with an aqueous sodium hydroxide solution (1 mM, 5x 15 mL). The obtained residue was purified by column chromatography (silica gel, eluent ethyl acetate/triethylamine 10:1). After drying (4 h, 60°C), an off-white solid was obtained (484 mg, 1.12 mmol, 78%). M.p.: 148–150°C; IR (KBr): ṽ [cm^-1^] = 3300 (N-H), [1684, 1643] (C = O), 1423; ^1^H-NMR (DMSO-*d*_6_, 500 MHz): δ (ppm) = 1.40 (s, 9H, 3x CH_3_), 2.34 (t, *J* = 5.1 Hz, 4H, *N*-CH_2_), 2.37 (t, *J* = 6.8 Hz, 2H, *N*-CH_2_), 3.22 (qd, *J* = 6.7 / 2.8 Hz, 2H, amide-*N*-CH_2_), 3.30 (t, *J* = 5.1 Hz, 4H, carbamate-*N*-CH_2_), 3.39 (d, *J* = 12.5 Hz, 1H, azepine-CH_2_, part B of an AB system), 3.53 (d, *J* = 12.5 Hz, 1H, azepine-CH_2_, part A of an AB system), 3.94 (d, *J* = 16.5 Hz, 1H, azepine-*N*-CH_2_), 4.37 (d, *J* = 16.6 Hz, 1H, azepine-*N*-CH_2_), 7.35 (ddd, *J* = 7.8 / 6.2 / 2.4 Hz, 1H, ArH), 7.38–7.51 (m, 5H, ArH), 7.58–7.62 (m, 1H, Ar H), 7.62–7.66 (m, 1H, ArH), 7.94 (t, *J* = 5.7 Hz, 1H, NH); ^13^C-NMR (DMSO-*d*_6_, 126 MHz): δ (ppm) = 28.0 (3C) (CH_3_), 36.1, 41.3, 42.7 (br), 43.7 (br), 52.0, 52.4, 56.7 (CH_2_), 122.8, 125.3, 127.6 (2C, isochronous by coincidence), 128.1, 128.5, 128.5, 129.7 (CH), 78.6, 132.9, 135.0, 136.1, 141.0, 153.7, 167.8, 169.9 (C); The structure was confirmed by 2D-NMR spectroscopy (H,H-COSY, H,C-HSQC); C_27_H_34_N_4_O_4_ (478.59); CHN: calc. C 67.76, H 7.16, N 11.71, found C 67.77, H 7.26, N 11.33; APCI-MS (ASAP, +, soft): m/z (%): 479 [M+H]^+^ (100); ESI-MS (DI, +): m/z (%): 517 [M+K]^+^ (6), 501 [M+Na]^+^ (100); HPLC (isocr.): 99.3% at 254 nm, t_M+S_ = 7.8 min, t_M_ (DMSO) = 1.1 min (ACN/buffer pH 2.7 30:70), λ_max_ [nm] = 231.

*tert-Butyl N-methyl-{2-[2-(6-oxo-6*,*7-dihydro-5H-dibenzo[b*,*d]azepin-5-yl)acetamido]ethyl}carbamate (4c)*. According to general procedure 4 from 2-(6-oxo-6,7-dihydro-5*H*-dibenzo[*b*,*d*]azepin-5-yl)acetic acid (**19**, 214 mg, 0.800 mmol), EDCl (200 mg, 1.04 mmol), DIPEA (166 μL, 0.960 mmol) and *N*-(*tert*-butoxycarbonyl)-*N*-methylethane-1,2-diamine (280 mg, 1.61 mmol) in DMF (4 mL) (21 h). Ethyl acetate (30 mL) was added and the mixture was washed with aqueous sodium hydroxide solution (1 mM, 4x 15 mL). The residing light brown oil was purified by column chromatography (silica gel, eluent ethyl acetate/petroleum ether 4:1). After drying (4 h, 60°C) a brown solid was obtained (88 mg, 0.21 mmol, 26%). M.p.: 80–82°C; IR (KBr): ṽ [cm^-1^] = 3328 (N-H), 1673 (C = O), 1156, 765; ^1^H-NMR (DMSO-*d*_6_, 500 MHz): δ (ppm) = 1.39 (s, 9H, 3x CH_3_), 2.80 (br, apparent ‘d’, 3H, *N*-CH_3_, peak split due to *N*-Boc rotamers), 3.13–3.22 (m, 2H, *N*-CH_2_), 3.25 (s, 2H, *N*-CH_2_), 3.38 (d, *J* = 12.4 Hz, 1H, azepine-CH_2_, part B of an AB system), 3.53 (d, *J* = 12.5 Hz, 1H, azepine-CH_2_, part A of an AB system), 3.90 (apparent ‘dd’, *J* = 16.9 Hz, 1H, azepine-*N*-CH_2_, part X of an AX system, peak split due to rotamers), 4.36 (d, *J* = 16.5 Hz, 1H, azepine-*N*-CH_2_, part A of an AX system), 7.33–7.38 (m, 1H, ArH), 7.39–7.51 (m, 5H, ArH), 7.59–7.62 (m, 1H, ArH), 7.63–7.66 (m, 1H, ArH), 8.12 (br s, 1H, NH); Signals between 3.13 ppm and 3.25 ppm overlap with each other and the HDO signal; ^13^C-NMR (DMSO-*d*_6_, 126 MHz): δ (ppm) = 28.0 (3C), [34.3 (w), 34.4 (w) split due to rotamers] (CH_3_), [36.8 (w), 37.1 (w) split due to rotamers], 41.3, [47.1 (w), 47.8 (w) split due to rotamers], 52.0 (CH_2_), 122.8, 125.3, 127.6 (2C, isochronous by coincidence), 128.1, 128.5, 128.6, 129.7 (CH), 78.4, 132.9 (w), 135.0, 136.1, 141.0, [154.6 (w), 154.9 (w) split due to rotamers], 168.0, 169.9; The structure was confirmed by 2D-NMR spectroscopy (H,H-COSY, H,C-HSQC); C_24_H_29_N_3_O_4_ (423.51); HRMS (ESI, +): m/z [M+Na]^+^ calc. 446.20503, found 446.20529; APCI-MS (ASAP, -): m/z (%): 422 [M-H]^-^ (100); ESI-MS (DI, +): m/z (%): 462 [M+K]^+^ (5), 446 [M+Na]^+^ (100); HPLC (grad.): 99.5% at 254 nm, t_M+S_ = 10.7 min, t_M_ (DMSO) = 1.2 min, λ_max_ [nm] = 235; HPLC (isocr.): 99.8% at 254 nm, t_M+S_ = 4.4 min, t_M_ (DMSO) = 1.0 min (ACN/water 50:50), λ_max_ [nm] = 232.

*2-(6-Oxo-6*,*7-dihydro-5H-dibenzo[b*,*d]azepin-5-yl)-N-[2-(piperazin-1-yl)ethyl]acetamide dihydrochloride (4d)*. According to general procedure 6 from *tert*-butyl 4-{2-[2-(6-oxo-6,7-dihydro-5*H*-dibenzo[*b*,*d*]azepin-5-yl)acetamido]ethyl}piperazine-1-carboxylate (**4b**, 239 mg, 0.500 mmol) in trifluoroacetic acid (6.7 mL, 88 mmol) and dichloromethane (25 mL) (18 h). After removal of the solvent by distillation under reduced pressure, the yellowish residue was mixed with sodium hydroxide solution (40 mL, 1 mM), alkalised to pH 10 with sodium hydroxide solution (8% m/m) and extracted with ethyl acetate (2x 50 mL). After the solvent was distilled off, the yellow residue (0.13 g) was precipitated as hydrochloride in 2-propanol (2 mL) with hydrogen chloride in 2-propanol (10 drops) and *n*-hexane (10 mL) overnight at room temperature. After solvent removal, the residue was dried (60°C, 8 h) to obtain an off-white solid (70 mg, 0.16 mmol, 31%). Decomp.: starting at 120°C; IR (KBr): ṽ [cm^-1^] = 3427 (N-H), 2925 (-C-H), [2652, 2454] (NR_3_H^+^ / NR_2_H_2_^+^), 1654 (C = O), 1441, 1383; ^1^H-NMR (DMSO-*d*_*6*_, 500 MHz): δ (ppm) = 3.18 (br s, CH_2_), 3.40 (d, J = 12.5 Hz, 1H, azepine-CH_2_, part B of an AB system), 3.43 (br s, CH_2_), 3.44 (br s, CH_2_), 3.52 (d, J = 12.5 Hz, 1H, azepine-CH_2_, part A of an AB system), 4.15 (br d, J = 16.6 Hz, 1H, azepine-N-CH_2_), 4.35 (d, J = 16.6 Hz, 1H, azepine-N-CH_2_), 7.36 (td, J = 7.5 / 1.3 Hz, 1H, ArH), 7.39–7.51 (m, 4H, ArH), 7.54 (dd, J = 8.3 / 1.3 Hz, 1H, ArH), 7.60 (dd, J = 7.8 / 1.6 Hz, 1H, ArH), 7.62–7.67 (m, 1H, ArH), 8.45 (s, 1H, amide-NH), 9.76 (br s, 2H, NH_2_^+^), 11.85 (br s, 1H, NH^+^); The signals between 3.18 ppm and 3.52 ppm overlap with each other and the HDO signal and were assigned with help of H,C-HSQC; ^13^C-NMR (DMSO-*d*_*6*_, 126 MHz): δ (ppm) = 33.5 (w), 39.7 (w, superimposed onto the DMSO-*d*_6_ signal), 41.3, 47.9, 52.3, 54.8 (w) (CH_2_), 123.2, 125.4, 127.6, 127.6, 128.1, 128.5, 128.6, 129.7 (CH), 132.9, 134.9, 136.1, 140.9, 168.7, 170.1 (C); The structure was confirmed by 2D-NMR spectroscopy (H,H-COSY, H,C-HSQC); C_22_H_28_Cl_2_N_4_O_2_ (451.39); HRMS (ESI, +): m/z [M+H]^+^ calc. 379.21285, found 379.21328; APCI-MS (ASAP, +): m/z (%): 379 [M+H]^+^ (100); ESI-MS (DI, +): m/z (%): 401 [M+Na]^+^ (12), 379 [M+H]^+^ (100); HPLC (isocr.): 97.2% at 254 nm, t_M+S_ = 4.3 min, t_M_ (DMSO) = 1.2 min (ACN/buffer pH 2.7 20:80), λ_max_ [nm] = 231.

*tert-Butyl (E/Z)-4-(2-{2-[5-(methoxyimino)-2-oxo-2*,*3*,*4*,*5-tetrahydro-1H-benzo[b]azepin-1-yl]acetamido}ethyl)piperazine-1-carboxylate (5b)*. According to general procedure 4 from (*E/Z*)-2-(5-(methoxyimino)-2-oxo-2,3,4,5-tetrahydro-1*H*-benzo[*b*]azepin-1-yl)acetic acid (**22**, 367 mg, 1.40 mmol), EDCl (314 mg, 1.64 mmol), DIPEA (291 μL, 1.68 mmol) and *tert*-butyl 4-(2-aminoethyl)piperazine-1-carboxylate (420 mg, 1.83 mmol) in DMF (4 mL) (21 h). Ethyl acetate (60 mL) was added to the mixture after solvent evaporation and washed with an aqueous sodium hydroxide solution (1 mM, 4x 30 mL). The resulting brown oil solidified in the desiccator and was recrystallised from ethyl acetate (1.6 mL) and petroleum ether (10 mL). After drying for 2 h at reduced pressure at 60°C, a colourless solid was obtained (193 mg, 0.408 mmol, 29%). M.p.: 130–131°C; IR (KBr): ṽ [cm^-1^] = 3307 (N-H), [1690, 1675, 1651] (C = O / C = N), 1456, 1423, 1380, 1247; ^1^H-NMR (DMSO-*d*_6_, 500 MHz): δ (ppm) = 1.39 (s, 9H, 3x CH_3_), 2.33 (t, *J* = 5.1 Hz, 4H, *N*-CH_2_), 2.37 (t, *J* = 6.7 Hz, 2H, *N*-CH_2_), 2.48 (br s, 2H, azepine-CH_2_), 2.95 (br s, 2H, azepine-CH_2_), 3.21 (q, *J* = 6.5 Hz, 2H, amide-*N*-CH_2_), 3.29 (t, *J* = 4.9 Hz, 4H, 2x carbamate-*N*-CH_2_), 3.92 (s, 3H, *O*-CH_3_), 4.20 (br s, 2H, azepine-*N*-CH_2_), 7.26 (td, *J* = 7.5 / 1.2 Hz, 1H, ArH), 7.33 (dd, *J* = 8.2 / 1.2 Hz, 1H, ArH), 7.38 (dd, *J* = 7.6 / 1.6 Hz, 1H, ArH), 7.47 (ddd, *J* = 8.1 / 7.3 / 1.8 Hz, 1H, ArH), 7.94 (t, *J* = 5.6 Hz, 1H, NH, partial H/D-exchange); ^13^C-NMR (DMSO-*d*_6_, 126 MHz): δ (ppm) = 28.0 (3C), 61.8 (CH_3_), 29.3, 30.8, 36.1, 51.6, 52.4, 56.7 (CH_2_), 123.1, 125.7, 129.4, 130.4 (CH), 78.6, 129.2, 142.0, 153.7, 157.2, 167.7, 171.4 (C); The structure was confirmed by 2D-NMR spectroscopy (H,H-COSY, H,C-HSQC: The signals at 42.6 ppm and 43.7 ppm corresponding to two CH_2_ groups are only assignable in the H,C-HSQC experiment); C_24_H_35_N_5_O_5_ (473.57); CHN: calc. C 60.87, H 7.45, N 14.79, found C 61.04, H 7.34, N 14.75; APCI-MS (ASAP, +): m/z (%): 474 [M+H]^+^ (100), 418 [M-55]^+^ (33); ESI-MS (DI, +): m/z (%): 512 [M+K]^+^ (5), 496 [M+Na]^+^ (100), 396 [M-77]^+^ (49); HPLC (isocr.): 94.8% at 254 nm, t_M+S_ = 3.4 min, t_M_ (DMSO) = 1.1 min (ACN/buffer pH 2.7 30:70), λ_max_ [nm] = 223.

*(E/Z)-5-(Methoxyimino)-1-[2-oxo-2-(pyrrolidin-1-yl)ethyl]-1*,*3*,*4*,*5-tetrahydro-2H-benzo[b]azepin-2-one (5c)*. According to general procedure 4 from (*E/Z*)-2-(5-(methoxyimino)-2-oxo-2,3,4,5-tetrahydro-1*H*-benzo[*b*]azepin-1-yl)acetic acid (**22**, 262 mg, 1.00 mmol), EDCl (499 mg, 2.60 mmol), DIPEA (416 μL, 2.40 mmol) and pyrrolidine (200 μL, 2.40 mmol) in DMF (4 mL) (87.5 h). The concentrated mixture was extracted according to general procedure 5 from ethyl acetate (3x 20 mL) and brine (20 mL), then water (20 mL). After storing the brown oil in the refrigerator overnight, the crystallised solid (271 mg) was recrystallised from ethyl acetate (1.2 mL) and petroleum ether (1 mL). After drying for 4 h under reduced pressure at 60°C, a colourless solid was obtained (98 mg, 0.31 mmol, 31%). M.p.: 158°C; IR (KBr): ṽ [cm^-1^] = [1675, 1655, 1634] (C = O / C = N), 1452, 1049; ^1^H-NMR (DMSO-*d*_6_, 500 MHz): δ (ppm) = 1.77 (p, *J* = 6.8 Hz, 2H, *C*-CH_2_), 1.89 (p, *J* = 6.8 Hz, 2H, *C*-CH_2_), 2.47 (br s, 2H, azepine-CH_2_), 2.95 (br s, 2H, azepine-CH_2_), 3.31 (t, *J* = 6.8 Hz, 2H, pyrrolidine-*N*-CH_2_), 3.42 (t, *J* = 6.8 Hz, 2H, pyrrolidine-*N*-CH_2_), 3.92 (s, 3H, *O*-CH_3_), 4.40 (br s, 2H, azepine-*N*-CH_2_), 7.25 (td, *J* = 7.5 / 1.1 Hz, 1H, ArH), 7.30 (dd, *J* = 8.2 / 1.2 Hz, 1H, ArH), 7.38 (dd, *J* = 7.6 / 1.6 Hz, 1H, ArH), 7.44–7.51 (m, 1H); ^13^C-NMR (DMSO-*d*_6_, 126 MHz): δ (ppm) = 61.7 (CH_3_), 23.6, 25.6, 29.4, 30.7, 44.9, 45.6, 50.9 (CH_2_), 123.2, 125.6, 129.3, 130.4 (CH), 129.3, 142.0, 157.3, 165.5, 171.2 (C); C_17_H_21_N_3_O_3_ (315.37); CHN: calc. C 64.74, H 6.71, N 13.32, found. C 64.60, H 6.65, N 13.24; APCI-MS (ASAP, +): m/z (%): 316 [M+H]^+^ (85), 284 [M-31]^+^ (41), 245 [M-70]^+^ (100); HPLC (grad.): 98.7% at 254 nm, t_M+S_ = 8.6 min, t_M_ (DMSO) = 1.2 min, λ_max_ [nm] = 221; HPLC (isocr.): 99.2% at 254 nm, t_M+S_ = 4.8 min, t_M_ (DMSO) = 1.2 min (ACN/water 30:70), λ_max_ [nm] = 220.

*(E/Z)-2-[5-(Methoxyimino)-2-oxo-2*,*3*,*4*,*5-tetrahydro-1H-benzo[b]azepin-1-yl]-N-[2-(piperazin-1-yl)ethyl]acetamide dihydrochloride (5d)*. According to general procedure 6 from *tert*-butyl (*E/Z*)-4-{2-[2-(5-(methoxyimino)-2-oxo-2,3,4,5-tetrahydro-1*H*-benzo[*b*]azepin-1-yl)acetamido]ethyl}piperazine-1-carboxylate (**5b**, 118 mg, 0.250 mmol) in trifluoroacetic acid (3.2 mL, 42 mmol) and dichloromethane (10 mL) (22 h). After removal of the solvent by distillation under reduced pressure, the brown residue was mixed with sodium hydroxide solution (6 mL, 4%) and extracted with ethyl acetate (3x 10 mL). A yellow solid (0.06 g) was obtained after solvent distillation. Precipitation as hydrochloride in 2-propanol (1 mL) with hydrogen chloride in 2-propanol (10 drops) and *n*-hexane (5 mL) overnight at room temperature. The mixture was subjected to reduced pressure and then dried at 60°C under reduced pressure for 4 h. A yellow solid was obtained (22 mg, 0.049 mmol, 20%). Decomp.: starting at 100°C; IR (KBr): ṽ [cm^-1^] = 3435 (N-H), 2935 (-C-H), 1653 (C = O), 1455, 1384; ^1^H-NMR (DMSO-*d*_6_, 500 MHz): δ (ppm) = 2.47 (br s, azepine-CH_2_), 2.95 (br s, 2H, azepine-CH_2_), 3.22 (br s, CH_2_), 3.30 (br s, CH_2_), 3.46 (br s, CH_2_), 3.52 (br s, CH_2_), 3.71 (br s, CH_2_), 3.92 (s, 3H, *O*-CH_3_), 4.29 (br s, 2H, azepine-*N*-CH_2_), 7.26 (td, *J* = 7.5 / 1.2 Hz, 1H, ArH), 7.38 (dd, *J* = 7.6 / 1.6 Hz, 2H, ArH), 7.49 (ddd, *J* = 8.7 / 7.3 / 1.6 Hz, 1H, ArH), 8.43 (br s, 1H, amide-NH), 9.76 (br s, 2H, NH_2_^+^), 11.83 (br s, 1H, NH^+^); One signal overlaps with the C*H*D_2_ signal of DMSO-*d*_5_ (H,C-HSQC cross peak 2.5; 30.8); The signals between 3.21 ppm and 3.71 ppm overlap with each other and the HDO signal; The signals between 2.47 and 3.71 were assigned with help of H,C-HSQC; ^13^C-NMR (DMSO-*d*_6_, 126 MHz): δ (ppm) = 61.8 (CH_3_), 29.3, 30.8, 33.4 (w), 39.7 (superimposed onto the DMSO-*d*_6_ signal, assigned by H,C-HSQC), 47.9, 51.9, 54.8 (w) (CH_2_), 123.4, 125.7, 129.3, 130.5 (CH), 129.1, 141.9, 157.2, 168.5, 171.5 (C); The structure was confirmed by 2D-NMR spectroscopy (H,H-COSY, H,C-HSQC); C_19_H_29_Cl_2_N_5_O_3_ (446.37); HRMS (ESI, +): m/z [M+H]^+^ calc. 374.21867, found 374.21888; APCI-MS (ASAP, +, soft): m/z (%): 374 [M+H]^+^ (100); ESI-MS (DI, +): m/z (%): 396 [M+Na]^+^ (3), 374 [M+H]^+^ (100); HPLC (isocr.): 93.7% at 254 nm, t_M+S_ = 6.2 min, t_M_ (DMSO) = 1.2 min (ACN/buffer pH 2.7 10:90), λ_max_ [nm] = 222.

*3-Chloro-9-iodo-7*,*12-dihydrobenzo[2*,*3]azepino[4*,*5-b]indol-6(5H)-one (7a)*. According to general procedure 1 from 8-chloro-3,4-dihydro-1*H*-benzo[*b*]azepin-2,5-dione (**6**, 1.89 g, 9.02 mmol) and 4-iodophenylhydrazine (2.52 g, 10.8 mmol) in glacial acetic acid (90 mL). After 75 min at 70°C, concentrated sulfuric acid (0.9 mL) was added, whereupon the temperature briefly rose to 78°C and the beige suspension turned dark brown. The mixture was stirred at 70°C for another 75 min. After cooling to room temperature, the suspension was poured onto sodium acetate solution (5%, 180 mL) and stored in the refrigerator overnight. After vacuum filtration, recrystallisation from ethanol (57 mL) and drying (3 h, 60°C), a light brown solid was obtained (2.778 g, 6.798 mmol, 75%). Decomp.: starting at 270°C; IR (KBr): ṽ [cm^-1^] = 3221 (N-H), 1647 (C = O), 1466, 1397, 791; ^1^H-NMR (DMSO-*d*_6_, 600 MHz): δ (ppm) = 3.54 (s, 2H, azepine-CH_2_), 7.29 (dd, *J* = 8.5 / 0.6 Hz, 1H, ArH), 7.31 (d, *J* = 2.1 Hz, 1H, ArH), 7.36 (dd, *J* = 8.4 / 2.2 Hz, 1H, ArH), 7.43 (dd, *J* = 8.5 / 1.7 Hz, 1H, ArH), 7.74 (d, *J* = 8.4 Hz, 1H, ArH), 8.09 (d, *J* = 1.7 Hz, 1H, ArH), 10.22 (s, 1H, azepine-NH), 11.83 (s, 1H, indole-NH); ^13^C-NMR (DMSO-*d*_6_, 151 MHz): δ (ppm) = 31.2 (CH_2_), 113.8, 121.5, 123.4, 126.6, 128.5, 130.2 (CH), 82.8, 107.0, 121.1, 129.0, 132.3, 132.4, 136.4, 136.7, 171.3 (C); C_16_H_10_ClIN_2_O (408.62); HRMS (ESI, +): m/z [M+Na]^+^ calc. 430.94186, found 430.94193; APCI-MS (ASAP, +): m/z (%): 409 [M+H]^+^ (100); HPLC (grad.): 95.3% at 254 nm, 95.1% at 280 nm, t_M+S_ = 11.4 min, t_M_ (DMSO) = 1.2 min, λ_max_ [nm] = 240, 320; HPLC (isocr.): 98.4% at 254 nm, 99.1% at 280 nm, t_M+S_ = 3.4 min, t_M_ (DMSO) = 1.0 min (ACN/water 60:40), λ_max_ [nm] = 239, 321.

*3*,*9-Dichloro-7*,*12-dihydrobenzo[2*,*3]azepino[4*,*5-b]indol-6(5H)-one (7b)*. According to general procedure 1 from 8-chloro-3,4-dihydro-1*H*-benzo[*b*]azepin-2,5-dione (**6**, 1.89 g, 9.02 mmol), 4-chlorophenylhydrazine hydrochloride (1.93 g, 10.8 mmol) and anhydrous sodium acetate (0.93 g, 11 mmol) in glacial acetic acid (90 mL). After 90 min at 70°C, concentrated sulfuric acid (0.9 mL) was added, whereupon the brown suspension darkened. The mixture was stirred at 70°C for another 2.5 h. After cooling to room temperature, the suspension was poured onto sodium acetate solution (5%, 180 mL) and stored in the refrigerator overnight. After vacuum filtration, recrystallisation from ethanol (96 mL) and drying (4 h, 60°C), an off-white solid was obtained (2.092 g, 6.596 mmol, 73%). Decomp.: ≥350°C (starting at 402°C); IR (KBr): ṽ [cm^-1^] = [3428, 3223] (N-H), 1649 (C = O), 1468, 1397; ^1^H-NMR (DMSO-*d*_6_, 600 MHz): δ (ppm) = 3.56 (s, 2H, azepine-CH_2_), 7.18 (dd, *J* = 8.6 / 2.1 Hz, 1H, ArH), 7.32 (d, *J* = 2.2 Hz, 1H, ArH), 7.37 (dd, *J* = 8.4 / 2.2 Hz, 1H, ArH), 7.44 (d, *J* = 8.5 Hz, 1H, ArH), 7.75 (d, *J* = 8.4 Hz, 1H, ArH), 7.79 (d, *J* = 2.0 Hz, 1H, ArH), 10.22 (s, 1H, azepine-NH), 11.85 (s, 1H, indole-NH); ^13^C-NMR (DMSO-*d*_6_, 151 MHz): δ (ppm) = 31.3 (CH_2_), 112.9, 117.4, 121.5, 122.2, 123.4, 128.5 (CH), 107.4, 121.2, 123.9, 127.5, 132.3, 133.1, 135.8, 136.7, 171.3 (C); C_16_H_10_Cl_2_N_2_O (317.17); CHN: calc. C 60.59, H 3.18, N 8.83, found C 60.64, H 3.23, N 8.50; APCI-MS (ASAP, +, soft): m/z (%): 317 [M+H]^+^ (100); HPLC (grad.): 95.2% at 254 nm, 93.8% at 280 nm, t_M+S_ = 11.0 min, t_M_ (DMSO) = 1.2 min, λ_max_ [nm] = 237, 319; HPLC (isocr.): 98.2% at 254 nm, 98.9% at 280 nm, t_M+S_ = 5.2 min, t_M_ (DMSO) = 1.2 min (ACN/water 50:50), λ_max_ [nm] = 238, 319.

*3-Chloro-9-trifluoromethyl-7*,*12-dihydrobenzo[2*,*3]azepino[4*,*5-b]indol-6(5H)-one (7c)*. According to general procedure 1 from 8-chloro-3,4-dihydro-1*H*-benzo[*b*]azepin-2,5-dione (**6**, 1.28 g, 6.10 mmol) and 4-trifluoromethylphenylhydrazine (1.29 g, 7.32 mmol) in glacial acetic acid (60 mL). After 75 min at 70°C, concentrated sulfuric acid (0.6 mL) was added, whereupon the yellow suspension turned brown. The mixture was stirred at 70°C for another 2 h. After cooling to room temperature, the suspension was poured onto sodium acetate solution (5%, 120 mL) and stored in the refrigerator for 3 d. After vacuum filtration, recrystallisation from ethanol (91 mL) and drying (4 h, 60°C), a yellow solid was obtained (1.230 g, 3.507 mmol, 57%). Decomp.: ≥350°C (350°C); IR (KBr): ṽ [cm^-1^] = [3435, 3210] (N-H), 1646 (C = O), 1309, 1103; ^1^H-NMR (DMSO-*d*_6_, 600 MHz): δ (ppm) = 3.65 (s, 2H, azepine-CH_2_), 7.33 (d, *J* = 2.2 Hz, 1H, ArH), 7.39 (dd, *J* = 8.4 / 2.2 Hz, 1H, ArH), 7.47 (ddq, *J* = 8.5 / 1.8 / 0.5 Hz, 1H, ArH), 7.62 (dq, *J* = 8.6 / 0.7 Hz, 1H, ArH), 7.78 (d, *J* = 8.4 Hz, 1H, ArH), 8.16 (dq, *J* = 1.6 / 0.7 Hz, 1H, ArH), 10.27 (s, 1H, azepine-NH), 12.12 (s, 1H, indole-NH); ^13^C-NMR (DMSO-*d*_6_, 151 MHz): δ (ppm) = 31.2 (CH_2_), 112.1, 116.0 (q, *J* = 4.4 Hz), 118.5 (q, *J* = 3.7 Hz), 121.5, 123.5, 128.6 (CH), 108.6, 120.1 (q, *J* = 31.3 Hz), 121.0, 125.0 (q, *J* = 271.1 Hz), 125.7, 132.6, 133.6, 136.8, 138.8, 171.3 (C); ^19^F-NMR (DMSO-*d*_6_, 471 MHz): δ (ppm) = -58.03; C_17_H_10_ClF_3_N_2_O (350.73); CHN: calc. C 58.22, H 2.87, N 7.99, found C 58.21, H 2.91, N 7.76; ESI-MS (DI, -): m/z (%): 349 [M-H]^-^ (100); HPLC (grad.): 95.4% at 254 nm, 93.6% at 280 nm, t_M+S_ = 11.2 min, t_M_ (DMSO) = 1.2 min, λ_max_ [nm] = 234, 318; HPLC (isocr.): 99.7% at 254 nm, 99.4% at 280 nm, t_M+S_ = 6.2 min, t_M_ (DMSO) = 1.0 min (ACN/water 50:50), λ_max_ [nm] = 233, 319.

*tert-Butyl 2-(3-chloro-9-iodo-6-oxo-7*,*12-dihydrobenzo[2*,*3]azepino[4*,*5-b]indol-5(6H)-yl)acetate (8a)*. According to general procedure 2 from 3-chloro-9-iodo-7,12-dihydrobenzo[3,2]azepino[4,5-*b*]indol-6(5*H*)-one (**7a**, 2.25 g, 5.51 mmol) with potassium *tert*-butoxide (0.93 g, 8.3 mmol) in dry tetrahydrofuran (100 mL). After 75 min, *tert*-butyl bromoacetate (0.24 mL, 1.6 mmol) was added, then the mixture was stirred at room temperature for 31 h. The solvent was distilled off under reduced pressure, water was added (150 mL) and the mixture was extracted with dichloromethane (3x 150 mL). The resulting solid from the combined evaporated organic phases (5.56 g) was purified by column chromatography (silica gel, eluent ethyl acetate/petroleum ether 2:5 → 1:0). A brown solid was obtained and recrystallised from acetone (56 mL). Drying (60°C, 2 h) led to an off-white solid (1.138 g, 2.177 mmol, 40%). Decomp.: starting at 228°C; IR (KBr): ṽ [cm^-1^] = 3290 (N-H), [1741, 1649] (C = O), 1369, 1227, 1162; ^1^H-NMR (DMSO-*d*_6_, 600 MHz): δ (ppm) = 1.25 (s, 9H, 3x CH_3_), 3.07 (br s, 1H, azepine-CH_2_), 3.98 (br s, 1H, azepine-CH_2_), 4.42 (br s, 2H, *N*-CH_2_), 7.31 (d, *J* = 8.4 Hz, 1H, ArH), 7.44 (dd, *J* = 8.5 / 1.7 Hz, 1H, ArH), 7.49 (dd, *J* = 8.4 / 2.1 Hz, 1H, ArH), 7.58 (d, *J* = 2.1 Hz, 1H, ArH), 7.73 (d, *J* = 8.4 Hz, 1H, ArH), 8.11 (d, *J* = 1.6 Hz, 1H, ArH), 11.97 (s, 1H, indole-NH); ^13^C-NMR (DMSO-*d*_6_, 151 MHz): δ (ppm) = 27.4 (3C) (CH_3_), 30.8 (w), 52.6 (CH_2_), 114.0, 123.8, 125.2, 126.7, 128.6, 130.2 (CH), 80.8, 82.8, 108.7, 124.2, 128.5, 132.4 (2C), 136.3, 140.2, 168.0, 170.0 (C); The structure was confirmed by 2D-NMR spectroscopy (H,H-COSY, H,C-HSQC, H,C-HMBC); The signals at 132.4 ppm corresponding to the quaternary C of the paullone scaffold (3 and 4a) are isochronous by coincidence; C_22_H_20_ClIN_2_O_3_ (522.77); HRMS (ESI, +): m/z [M+Na]^+^ calc. 545.00994, found 545.00997; ESI-MS (DI, -): m/z (%): 521 [M-H]^-^ (100), 465 [M-57]^-^ (20); HPLC (grad.): 99.4% at 254 nm, 99.4% at 280 nm, t_M+S_ = 13.0 min, t_M_ (DMSO) = 1.2 min, λ_max_ [nm] = 238, 320; HPLC (isocr.): 99.7% at 254 nm, 99.9% at 280 nm, t_M+S_ = 3.9 min, t_M_ (DMSO) = 1.2 min (ACN/water 70:30), λ_max_ [nm] = 241, 317.

*tert-Butyl 2-(3*,*9-dichloro-6-oxo-7*,*12-dihydrobenzo[2*,*3]azepino[4*,*5-b]indol-5(6H)-yl)acetate (8b)*. According to general procedure 2 from 3,9-dichloro-7,12-dihydrobenzo[2,3]azepino[4,5-*b*]indol-6(5*H*)-one (**7b**, 1.776 g, 5.600 mmol) with potassium *tert*-butoxide (754 mg, 6.72 mmol) in tetrahydrofuran (100 mL). After 1 h at room temperature, the mixture was cooled to 0°C and a solution of *tert*-butyl bromoacetate (0.91 mL, 6.2 mmol) in THF (10 mL) was added, then the mixture was stirred at 0°C for 1 h, then at room temperature for 22 h. The solvent was distilled off under reduced pressure, water was added (150 mL) and the mixture was extracted with dichloromethane (3x 150 mL). The resulting light brown solid (2.98 g) was recrystallised from ethanol (84.5 mL). After drying (60°C, 4 h), a light yellow solid was obtained (1.132 g, 2.625 mmol, 47%). Decomp.: starting at 213°C; IR (KBr): ṽ [cm^-1^] = 3336 (N-H), [1736, 1654] (C = O), 1230, 1163; ^1^H-NMR (DMSO-*d*_6_, 500 MHz): δ (ppm) = 1.25 (s, 9H, 3x CH_3_), 3.08 (br s, 1H, azepine-CH_2_), 3.97 (br s, 1H, azepine-CH_2_), 4.42 (br s, 2H, *N*-CH_2_), 7.19 (dd, *J* = 8.6 / 2.1 Hz, 1H, ArH), 7.46 (d, *J* = 8.6 Hz, 1H, ArH), 7.50 (dd, *J* = 8.5 / 2.1 Hz, 1H, ArH), 7.59 (d, *J* = 2.1 Hz, 1H, ArH), 7.74 (d, *J* = 8.4 Hz, 1H, ArH), 7.81 (d, *J* = 2.1 Hz, 1H, ArH), 11.99 (s, 1H, indole-NH); ^13^C-NMR (DMSO-*d*_6_, 126 MHz): δ (ppm) = 27.4 (3C) (CH_3_), 30.9 (w), 52.7 (CH_2_), 113.1, 117.6, 122.3, 123.8, 125.2, 128.6 (CH), 80.8, 109.2, 123.9, 124.3, 127.0, 132.5, 133.1, 135.7, 140.2, 168.0, 170.1 (C); The structure was confirmed by 2D-NMR spectroscopy (H,H-COSY, H,C-HSQC); C_22_H_20_Cl_2_N_2_O_3_ (431.31); CHN: calc. C 61.26, H 4.67, N 6.50, found C 61.46, H 4.70, N 6.21; ESI-MS (DI, -): m/z (%): 429 [M-H]^-^ (100), 373 [M-57]^-^ (22); HPLC (grad.): 98.6% at 254 nm, 99.3% at 280 nm, t_M+S_ = 12.6 min, t_M_ (DMSO) = 1.2 min, λ_max_ [nm] = 238, 316; HPLC (isocr.): 99.5% at 254 nm, 99.6% at 280 nm, t_M+S_ = 3.4 min, t_M_ (DMSO) = 1.3 min (ACN/water 70:30), λ_max_ [nm] = 235, 318.

*tert-Butyl 2-(3-chloro-6-oxo-9-trifluoromethyl-7*,*12-dihydrobenzo[2*,*3]azepino[4*,*5-b]indol-5(6H)-yl)acetate (8c)*. According to general procedure 2 from 3-chloro-9-trifluoromethyl-7,12-dihydrobenzo[2,3]azepino[4,5-*b*]indol-6(5*H*)-one (**7c**, 1.159 g, 3.305 mmol) with potassium *tert*-butoxide (447 mg, 3.98 mmol) in tetrahydrofuran (60 mL). After 1 h at room temperature, the mixture was cooled to 0°C and a solution of *tert*-butyl bromoacetate (0.54 mL, 3.7 mmol) in THF (18 mL) was added, then the mixture was stirred at 0°C for 1 h, then at room temperature for 21 h. The solvent was distilled off under reduced pressure, water was added (150 mL) and the mixture was extracted with dichloromethane (3x 150 mL). An insoluble residue (1.27 g) was filtered off. The insoluble residue and the residue from the evaporated combined organic phases (0.73 g) were recrystallised from ethyl acetate (27 mL) and dried (60°C, 4 h) to yield an off-white solid (953 mg, 2.05 mmol, 62%). Decomp.: starting at 248°C; IR (KBr): ṽ [cm^-1^] = 3313 (N-H), [1737, 1651] (C = O), 1306, 1160, 1104; ^1^H-NMR (DMSO-*d*_6_, 500 MHz): δ (ppm) = 1.24 (s, 9H, 3x CH_3_), 3.13 (br s, 1H, azepine-CH_2_), 4.12 (br s, 1H, azepine-CH_2_), 4.44 (br s, 2H, *N*-CH_2_), 7.48 (dd, 1H, ArH), 7.52 (dd, *J* = 8.4 / 2.1 Hz, 1H, ArH), 7.61 (d, *J* = 2.1 Hz, 1H, ArH), 7.64 (dt, *J* = 8.6 / 0.8 Hz, 1H, ArH), 7.77 (d, *J* = 8.4 Hz, 1H, ArH), 8.18 (dd, *J* = 1.9 / 0.9 Hz, 1H, ArH), 12.27 (s, 1H, indole-NH); ^13^C-NMR (DMSO-*d*_6_, 126 MHz): δ (ppm) = 27.4 (3C) (CH_3_), 30.8 (w in DEPT135), 52.6 (CH_2_), 112.3, 116.2 (q, J = 4.2 Hz), 118.6 (q, J = 3.6 Hz), 123.9, 125.3, 128.7 (CH), 80.9, 110.4, 120.17 (apparent d, J = 31.2 Hz), 124.1, 125.4 (apparent d, J = 271.5 Hz), 132.7, 133.6, 138.7, 140.4, 168.0, 170.1 (C); The signal at 125.3 ppm appears doubled in intensity in the CPD-experiment, but not in the DEPT135-experiment: a CH- and a C-signal are isochronous by coincidence; C_23_H_20_ClF_3_N_2_O_3_ (464.87); CHN: calc. C 59.43, H 4.34, N 6.03, found C 59.35, H 4.31, N 5.80; ESI-MS (DI, -): m/z (%): 463 [M-H]^-^ (100), 407 [M-57]^-^ (27); HPLC (grad.): 98.6% at 254 nm, 98.5% at 280 nm, t_M+S_ = 12.7 min, t_M_ (DMSO) = 1.2 min, λ_max_ [nm] = 239, 315; HPLC (isocr.): 99.4% at 254 nm, 99.6% at 280 nm, t_M+S_ = 4.7 min, t_M_ (DMSO) = 1.4 min (ACN/water 65:35), λ_max_ [nm] = 235, 316.

*2-(3-Chloro-9-iodo-6-oxo-7*,*12-dihydrobenzo[2*,*3]azepino[4*,*5-b]indol-5(6H)-yl)acetic acid (9a)*. According to general procedure 3 under nitrogen from *tert*-butyl 2-(3-chloro-9-iodo-6-oxo-7,12-dihydrobenzo[2,3]azepino[4,5-*b*]indol-5(6*H*)-yl)acetate (**8a**, 680 mg, 1.30 mmol) with trifluoroacetic acid (4.0 mL, 52 mmol) in dichloromethane (16 mL) (5.5 h). The solvent was removed under vacuum and the residue (0.76 g) was recrystallised from ethanol (7.5 mL) and dried (60°C, 7 h) to yield a pearlescent solid (498 mg). 183 mg of the solid was recrystallised from THF (5 mL) and water (10 mL) and dried (60°C, 7.5 h) to obtain an off-white solid (170 mg, 0.364 mmol, 28%). Decomp.: starting at 260°C; IR (KBr): ṽ [cm^-1^] = 3317 (N-H), [1707, 1644] (C = O), 1408, 1362, 1303; ^1^H-NMR (DMSO-*d*_6_, 500 MHz): δ (ppm) = 3.10 (br s, 1H, azepine-CH_2_), 3.98 (br s, 1H, azepine-CH_2_), 4.38 (br s, 2H, *N*-CH_2_), 7.31 (d, *J* = 8.5 Hz, 1H, ArH), 7.45 (dd, *J* = 8.5 / 1.7 Hz, 1H, ArH), 7.49 (dd, *J* = 8.4 / 2.1 Hz, 1H, ArH), 7.57 (d, *J* = 2.1 Hz, 1H, ArH), 7.73 (d, *J* = 8.5 Hz, 1H, ArH), 8.12 (d, *J* = 1.6 Hz, 1H, ArH), 11.97 (s, 1H, indole-NH), 12.81 (br s, 1H, COOH); ^13^C-NMR (DMSO-*d*_6_, 126 MHz): δ (ppm) = 30.9, 52.2 (CH_2_), 114.0, 123.6, 125.2, 126.8, 128.6, 130.3 (CH), 82.9, 108.8, 124.0, 128.5, 132.4, 132.4, 136.3, 140.5, 170.2, 170.6 (C); C_18_H_12_ClIN_2_O_3_ (466.66); HRMS (ESI, +): m/z [M+Na]^+^ calc. 488.94733, found 488.94700; APCI-MS (ASAP, -): m/z (%): 465 [M-H]^-^ (100); ESI-MS (DI, -): m/z (%): 465 [M-H]^-^ (100), 385 [M-81]^-^ (22); HPLC (isocr.): 96.4% at 254 nm, 96.4% at 280 nm, t_M+S_ = 4.3 min, t_M_ (DMSO) = 1.1 min (ACN/buffer pH 2.7 50:50), λ_max_ [nm] = 234, 321.

*2-(3*,*9-Dichloro-6-oxo-7*,*12-dihydrobenzo[2*,*3]azepino[4*,*5-b]indol-5(6H)-yl)acetic acid (9b)*. According to general procedure 3 under nitrogen from *tert*-butyl 2-(3,9-dichloro-6-oxo-7,12-dihydrobenzo[2,3]azepino[4,5-*b*]indol-5(6*H*)-yl)acetate (**8b**, 1.078 g, 2.500 mmol) with trifluoroacetic acid (7.6 mL, 99 mmol) in dichloromethane (32 mL) (5 h). The solvent was removed under vacuum and the residue (1.12 g) was recrystallised from ethanol (12 mL) and dried (60°C, 7 h) to yield a light yellow solid (874 mg). 595 mg of the solid was recrystallised from THF (3.5 mL) and water (10 mL) and dried (60°C, 8 h) to obtain a light yellow solid (547 mg, 1.46 mmol, 58%). Decomp.: 287–289°C; IR (KBr): ṽ [cm^-1^] = [3440, 3356, 3322] (N-H / O-H), 1724 (C = O, carboxylic acid), 1655 (C = O, lactam), 1408, 1306, 1242; ^1^H-NMR (DMSO-*d*_6_, 600 MHz): δ (ppm) = 3.12 (br s, 1H, azepine-CH_2_), 3.98 (br s, 1H, azepine-CH_2_), 4.39 (br s, 2H, *N*-CH_2_), 7.19 (dd, *J* = 8.6 / 2.1 Hz, 1H, ArH), 7.46 (dd, *J* = 8.6 / 0.4 Hz, 1H, ArH), 7.49 (dd, *J* = 8.5 / 2.1 Hz, 1H, ArH), 7.58 (d, *J* = 2.1 Hz, 1H, ArH), 7.73 (d, *J* = 8.4 Hz, 1H, ArH), 7.82 (d, *J* = 2.0 Hz, 1H, ArH), 11.98 (s, 1H, indole-NH), 12.81 (br s, 1H, COOH); ^13^C-NMR (DMSO-*d*_6_, 151 MHz): δ (ppm) = 30.9, 52.2 (CH_2_), 113.1, 117.6, 122.3, 123.6, 125.2, 128.6 (CH), 109.3, 123.9, 124.1, 127.0, 132.4, 133.1, 135.7, 140.5, 170.2, 170.6 (C); C_18_H_12_Cl_2_N_2_O_3_ (375.21); HRMS (ESI, +): m/z [M+Na]^+^ calc. 397.01172, found 397.01199; APCI-MS (ASAP, -): m/z (%): 373 [M-H]^-^ (100); ESI-MS (DI, -): m/z (%): 373 [M-H]^-^ (100), 293 [M-81]^-^ (25); HPLC (isocr.): 97.1% at 254 nm, 97.3% at 280 nm, t_M+S_ = 3.4 min, t_M_ (DMSO) = 1.1 min (ACN/buffer pH 2.7 50:50), λ_max_ [nm] = 232, 319.

*2-(3-Chloro-6-oxo-9-trifluoromethyl-7*,*12-dihydrobenzo[2*,*3]azepino[4*,*5-b]indol-5(6H)-yl)acetic acid (9c)*. According to general procedure 3 under nitrogen from *tert*-butyl 2-(3-chloro-6-oxo-9-trifluoromethyl-7,12-dihydrobenzo[2,3]azepino[4,5-*b*]indol-5(6*H*)-yl)acetate (**8c**, 906 mg, 1.95 mmol) with trifluoroacetic acid (6.3 mL, 82 mmol) in dichloromethane (20 mL) (18 h). The solvent was removed under vacuum and the residue (1.05 g) was recrystallised from ethanol (5 mL) and dried (60°C, 5 h) to yield a colourless solid (285 mg). 170 mg of the solid was recrystallised from THF (2 mL) and water (5 mL) and dried (60°C, 7.5 h) to obtain a colourless solid (145 mg, 0.355 mmol, 18%). Decomp.: starting at 264°C; IR (KBr): ṽ [cm^-1^] = [3367, 3312] (N-H / O-H), 1721 (C = O, carboxylic acid), 1655 (C = O, lactam), 1308, 1115; ^1^H-NMR (DMSO-*d*_6_, 500 MHz): δ (ppm) = 3.17 (br s, 1H, azepine-CH_2_), 4.13 (br s, 1H, azepine-CH_2_), 4.40 (s, 2H, *N*-CH_2_), 7.48 (dd, *J* = 8.7 / 1.8 Hz, 1H, ArH), 7.52 (dd, *J* = 8.4 / 2.1 Hz, 1H, ArH), 7.60 (d, *J* = 2.1 Hz, 1H, ArH), 7.64 (dt, *J* = 8.5 / 0.8 Hz, 1H, ArH), 7.77 (d, *J* = 8.4 Hz, 1H, ArH), 8.19 (dd, *J* = 1.9 / 0.9 Hz, 1H, ArH), 12.27 (s, 1H, indole-NH), 12.81 (br s, 1H, COOH); ^13^C-NMR (DMSO-*d*_6_, 126 MHz): δ (ppm) = 30.9 (w in DEPT135), 52.2 (CH_2_), 112.3, 116.2 (q, *J* = 3.7 Hz), 118.6 (q, *J* = 3.0 Hz), 123.7, 125.3, 128.7 (CH), 110.5, 120.2 (d, *J* = 31.2 Hz), 123.9, 125.4 (d, *J* = 271.0 Hz), 132.7, 133.6, 138.7, 140.6, 170.3, 170.6 (C); The signal at 125.3 ppm appears doubled in intensity in the CPD-experiment, but not in the DEPT135-experiment: a CH- and a C-signal are isochronous by coincidence; C_19_H_12_ClF_3_N_2_O_3_ (408.76); HRMS (ESI, +): m/z [M+Na]^+^ calc. 431.03808, found 431.03841; APCI-MS (ASAP, -): m/z (%): 407 [M-H]^-^ (100); ESI-MS (DI, -): m/z (%): 407 [M-H]^-^ (100), 327 [M-81]^-^ (21); HPLC (isocr.): 99.2% at 254 nm, 98.3% at 280 nm, t_M+S_ = 3.9 min, t_M_ (DMSO) = 1.1 min (ACN/buffer pH 2.7 50:50), λ_max_ [nm] = 233, 315.

*tert-Butyl {2-[2-(3-chloro-9-iodo-6-oxo-7*,*12-dihydrobenzo[2*,*3]azepino[4*,*5-b]indol-5(6H)-yl)acetamido]ethyl}(methyl)carbamate (10a)*. According to general procedure 4 from 2-(3-chloro-9-iodo-6-oxo-7,12-dihydrobenzo[2,3]azepino[4,5-*b*]indol-5(6*H*)-yl)acetic acid (**9a**, 303 mg, 0.650 mmol), EDCl (162 mg, 0.845 mmol), HOBt (105 mg, 0.780 mmol), DIPEA (135 μL, 0.780 mmol) and *N*-(*tert*-butoxycarbonyl)-*N*-methylethane-1,2-diamine (159 mg, 0.913 mmol) in DMF (5 mL) (21.5 h). Water (50 mL) was added and the mixture was stored in the refrigerator (4°C) overnight to yield a colourless precipitate which was filtrated off and dried (6 h, 60°C) to yield a light yellow solid (361 mg, 0.580 mmol, 89%). Decomp.: starting at 236°C; IR (KBr): ṽ [cm^-1^] = 3326 (N-H), [1685, 1641] (C = O), 1159; ^1^H-NMR (DMSO-*d*_6_, 500 MHz): δ (ppm) = 1.39 (s, 9H, 3x CH_3_), 2.80 and 2.82 (br, 3H, *N*-CH_3_, peak split due to rotamers), 3.08 (br s, azepine-CH_2_), 3.21 (br s, *N*-CH_2_), 3.86–4.23 (m, *N*-CH_2_ / azepine-CH_2_), 4.34 (br s, 1H, *N*-CH_2_), 7.31 (d, *J* = 8.5 Hz, 1H, ArH), 7.45 (dd, *J* = 8.5 / 1.8 Hz, 1H, ArH), 7.47 (dd, *J* = 8.5 / 2.1 Hz, 1H, ArH), 7.67 (d, *J* = 2.1 Hz, 1H, ArH), 7.71 (d, *J* = 8.4 Hz, 1H, ArH), 8.11 (d, *J* = 1.8 Hz, 1H, ArH), 8.19 and 8.24 (br, 1H, NH, peak split due to rotamers), 11.95 (s, 1H, indole-NH); The signals between 3.08 ppm and 4.34 ppm overlap with each other and the HDO signal; ^13^C-NMR (DMSO-*d*_6_, 126 MHz): δ (ppm) = 28.0 (3C), [34.5 (w), 34.6 (w) split due to rotamers] (CH_3_), 31.0, [36.9 (w), 37.3 (w) split due to rotamers], [47.1 (w), 47.8 (w) split due to rotamers], 53.5 (CH_2_), 114.0, 124.0, 125.1, 126.8, 128.5 (2C), 130.3 (CH), 78.4, 82.8, 108.9, 123.8 (br, w), 132.3 (w), 132.4, 136.3, 141.0, [154.6 (w), 154.9 (w) split due to rotamers], 168.3, 170.0 (C); The structure was confirmed by 2D-NMR spectroscopy (H,H-COSY, H,C-HSQC, H,C-HMBC); The signals at 128.5 ppm corresponding to carbons in position 1 (CH) and 7b (C) of the paullone scaffold are isochronous by coincidence; C_26_H_28_ClIN_4_O_4_ (622.89); CHN: calc. C 50.14, H 4.53, N 8.99, found C 50.13, H 4.51, N 8.61; ESI-MS (DI, -): m/z (%): 621 [M-H]^-^ (100), 521 [M-101]^-^ (8); HPLC (grad.): 95.7% at 254 nm, 96.2% at 280 nm, t_M+S_ = 12.1 min, t_M_ (DMSO) = 1.2 min, λ_max_ [nm] = 235, 321; HPLC (isocr.): 95.9% at 254 nm, 97.7% at 280 nm, t_M+S_ = 4.7 min, t_M_ (DMSO) = 1.3 min (ACN/water 60:40), λ_max_ [nm] = 241, 317.

*tert-Butyl {2-[2-(3*,*9-dichloro-6-oxo-7*,*12-dihydrobenzo[2*,*3]azepino[4*,*5-b]indol-5(6H)-yl)acetamido]ethyl}(methyl)carbamate (10b)*. According to general procedure 4 from 2-(3,9-dichloro-6-oxo-7,12-dihydrobenzo[2,3]azepino[4,5-*b*]indol-5(6*H*)-yl)acetic acid (**9b**, 282 mg, 0.752 mmol), EDCl (187 mg, 0.975 mmol), HOBt (122 mg, 0.900 mmol), DIPEA (156 μL, 0.900 mmol) and *N*-(*tert*-butoxycarbonyl)-*N*-methylethane-1,2-diamine (230 mg, 1.32 mmol) in DMF (5 mL) (20 h). Water (50 mL) was added and the mixture was stored in the refrigerator (4°C) overnight to yield a light yellow precipitate which was recrystallised from acetone (15.8 mL) and dried (6 h, 60°C) to obtain a light yellow solid (204 mg, 0.384 mmol, 51%). Decomp.: starting at 237°C; IR (KBr): ṽ [cm^-1^] = 3322 (N-H), [1686, 1642] (C = O), 1365, 1160; ^1^H-NMR (DMSO-*d*_6_, 500 MHz): δ (ppm) = 1.39 (s, 9H, 3x CH_3_), 2.80 and 2.82 (br, 3H, *N*-CH_3_, peak split due to rotamers), 3.02–3.36 (m, *N*-CH_2_ / azepine-CH_2_), 4.01 (br s, *N*-CH_2_ / azepine-CH_2_), 4.34 (br s, 1H, *N*-CH_2_), 7.19 (dd, *J* = 8.6 / 2.1 Hz, 1H, ArH), 7.44–7.51 (m, 2H, ArH, d and dd superimposed), 7.68 (d, *J* = 2.1 Hz, 1H, ArH), 7.72 (d, *J* = 8.3 Hz, 1H, ArH), 7.81 (d, *J* = 2.0 Hz, 1H, ArH), 8.19 and 8.24 (br, 1H, NH, peak split due to rotamers), 11.97 (s, 1H, indole-NH); The signals between 3.00 ppm and 4.34 ppm overlap with each other and the HDO signal; ^13^C-NMR (DMSO-*d*_6_, 126 MHz): δ (ppm) = 28.0, [34.48 (w), 34.50 (w) split due to rotamers] (CH_3_), 31.1, [36.9 (br, w), 37.3 (br, w) split due to rotamers], [47.1 (br, w), 47.8 (br, w) split due to rotamers], 53.5 (w in DEPT135) (CH_2_), 113.1, 117.6, 122.3, 124.0 (br), 125.1, 128.5 (CH), 78.4, 109.4, 123.9 (2C), 127.0, 132.4, 133.1, 135.7, 141.0, 168.3, 170.0; No signal is observed for the carbonyl-C of the carbamate, which appears as a weak signal between 154 and 156 ppm in comparable N-substituted paullone derivatives; The structure was confirmed by 2D-NMR spectroscopy (H,H-COSY, H,C-HSQC, H,C-HMBC); The signals at 123.9 ppm corresponding to the quaternary carbons in position 9 and 12b of the paullone scaffold are isochronous by coincidence; C_26_H_28_Cl_2_N_4_O_4_ (531.43); CHN: calc. C 58.76, H 5.31, N 10.54, found C 58.73, H 5.27, N 10.17; ESI-MS (DI, -): m/z (%): 529 [M-H]^-^ (100), 401 [M-129]^-^ (25); HPLC (grad.): 95.4% at 254 nm, 95.9% at 280 nm, t_M+S_ = 11.7 min, t_M_ (DMSO) = 1.2 min, λ_max_ [nm] = 234, 319; HPLC (isocr.): 96.1% at 254 nm, 96.1% at 280 nm, t_M+S_ = 3.8 min, t_M_ (DMSO) = 1.3 min (ACN/water 60:40), λ_max_ [nm] = 233, 319.

*tert-Butyl {2-[2-(3-chloro-6-oxo-9-trifluoromethyl-7*,*12-dihydrobenzo[2*,*3]azepino[4*,*5-b]indol-5(6H)-yl)acetamido]ethyl}(methyl)carbamate (10c)*. According to general procedure 4 from 2-(3-chloro-6-oxo-9-trifluormethyl-7,12-dihydrobenzo[2,3]azepino[4,5-*b*]indol-5(6*H*)-yl)acetic acid (**9c**, 265 mg, 0.648 mmol), EDCl (162 mg, 0.845 mmol), HOBt (105 mg, 0.780 mmol), DIPEA (135 μL, 0.780 mmol) and *N*-(*tert*-butoxycarbonyl)-*N*-methylethane-1,2-diamine (250 mg, 1.43 mmol) in DMF (5 mL) (17 h). Water (50 mL) was added and the mixture was stored in the refrigerator (4°C) for 3 h to yield a precipitate which was recrystallised from ethyl acetate (35 mL) and dried (4 h, 60°C) to obtain an off-white solid (297 mg, 0.526 mmol, 81%). Decomp.: 251–252°C; IR (KBr): ṽ [cm^-1^] = 3306 (N-H), 1642 (C = O), 1305, 1161, 1114; ^1^H-NMR (DMSO-*d*_6_, 500 MHz): δ (ppm) = 1.38 (s, 9H, 3x CH_3_), 2.79 and 2.81 (br, 3H, *N*-CH_3_, peak split due to rotamers), 3.04–3.28 (m, 5H, *N*-CH_2_ / azepine-CH_2_, superimposed with the HDO signal at 3.33 ppm), 4.12 (br s, *N*-CH_2_ / azepine-CH_2_), 4.35 (br s, *N*-CH_2_), 7.45–7.53 (m, dd superimposed to an apparent td, 2H, ArH), 7.64 (apparent dt, *J* = 8.6 / 0.7 Hz, 1H, ArH), 7.70 (d, *J* = 2.1 Hz, 1H, ArH), 7.75 (d, *J* = 8.4 Hz, 1H, ArH), 8.19 (dq, *J* = 1.5 / 0.7 Hz, 1H, ArH), 8.19 and 8.24 (br, 1H, amide-NH, peak split due to rotamers, superimposed onto the ArH signal at 8.19 ppm), 12.25 (s, 1H, indole-NH); The signals at 4.12 ppm and 4.35 ppm overlap with each other; ^13^C-NMR (DMSO-*d*_6_, 126 MHz): δ (ppm) = 28.0 (3C), [34.5 (w), 34.6 (w) split due to rotamers] (CH_3_), 31.0, [36.9 (br, w), 37.3 (br, w) split due to rotamers], [47.1 (br, w), 47.8 (br, w) split due to rotamers], 53.5 (CH_2_), 112.3, 116.2 (q, *J* = 4.5 Hz), 118.6 (q, *J* = 3.2 Hz), 124.0, 125.1, 128.6 (CH), 78.4, 110.6, 120.2 (q, *J* = 31.3 Hz), [123.7 (br, w), 123.8 (br, w) split due to rotamers], 125.3, 125.4 (q, *J* = 271.9 Hz), 132.6, 133.6, 138.7, 141.2, [154.6 (br, w), 154.9 (br, w) split due to rotamers], 168.2, 170.0 (C); The structure was confirmed by 2D-NMR spectroscopy (H,H-COSY, H,C-HSQC, H,C-HMBC); C_27_H_28_ClF_3_N_4_O_4_ (564.99); CHN: calc. C 57.40, H 5.00, N 9.92, found C 57.62, H 4.97, N 9.64; APCI-MS (ASAP, -, soft): m/z (%): 563 [M-H]^-^ (100), 463 [M-101]^-^ (47), 435 [M-129]^-^ (94); ESI-MS (DI, -): m/z (%): 563 [M-H]^-^ (100), 463 [M-101]^-^ (8); HPLC (grad.): 99.5% at 254 nm, 98.7% at 280 nm, t_M+S_ = 11.9 min, t_M_ (DMSO) = 1.2 min, λ_max_ [nm] = 234, 316; HPLC (isocr.): 98.8% at 254 nm, 98.1% at 280 nm, t_M+S_ = 4.2 min, t_M_ (DMSO) = 1.0 min (ACN/water 60:40), λ_max_ [nm] = 234, 316.

*tert-Butyl 2-(2-oxo-2*,*3*,*4*,*5-tetrahydro-1H-benzo[b]azepin-1-yl)acetate (12)*. According to general procedure 2, 1,3,4,5-tetrahydro-2*H*-benzo[*b*]azepin-2-one (**11**, 240 mg, 1.49 mmol) in dry tetrahydrofuran (28 mL) with potassium *tert*-butoxide (340 mg, 3.03 mmol); after 1 h, *tert*-butyl bromoacetate (0.24 mL, 1.6 mmol) was added, then the mixture was stirred at room temperature for 2.5 h. The solvent was distilled off under reduced pressure and the residue was taken up in dichloromethane (130 mL) and washed with water (130 mL). The aqueous phase was extracted three times with dichloromethane (130 mL each). The solvent from the combined organic phases was removed under reduced pressure. Recrystallisation of the residue from petroleum ether (1 mL) at 67°C in an oil bath yielded colourless crystals (340 mg, 1.23 mmol, 83%). M.p.: 86–88°C; IR (KBr): ṽ [cm^-1^] = 1734 (C = O, carboxylic acid ester), 1673 (C = O, lactam), 1663, 1236, 1159; ^1^H-NMR (DMSO-*d*_6_, 500 MHz): δ (ppm) = 1.36 (s, 9H, 3x CH_3_), 2.08 (br s, 2H, azepine-CH), 2.16 (t, *J* = 6.6 Hz, 2H, azepine-CH), 2.84 (br s, 2H, azepine-CH), 4.39 (s, 2H, *N*-CH_2_), 7.18 (td, *J* = 7.3 / 1.5 Hz, 1H, ArH), 7.22–7.34 (m, 3H, ArH); ^13^C-NMR (DMSO-*d*_6_, 126 MHz): δ (ppm) = 27.5 (3C) (CH_3_), 28.1, 29.0, 32.3, 50.1 (CH_2_), 122.0, 125.9, 127.4, 129.2 (CH), 80.9, 135.2, 142.1, 168.1, 171.8 (C); C_16_H_21_NO_3_ (275.35); CHN: calc. C 69.79, H 7.69, N 5.09, found C 70.15, H 7.44, N 4.96; APCI-MS (ASAP, +): m/z (%): 551 [2M+H]^+^ (1), 276 [M+H]^+^ (4), 220 [M-55]^+^ (100), 202 [M-73]^+^ (15), 174 [M-101]^+^ (38), 146 [M-129]^+^ (55); HPLC (grad.): 99.4% at 254 nm, t_M+S_ = 11.2 min, t_M_ (DMSO) = 1.2 min, λ_max_ [nm] = 239.

*2-(2-Oxo-2*,*3*,*4*,*5-tetrahydro-1H-benzo[b]azepin-1-yl)acetic acid (13)*. According to a procedure from Li *et al*. [52]. Aqueous phosphoric acid 85% (0.38 mL, 5.6 mmol) was added dropwise to a solution of the *tert*-butyl ester **12** (300 mg, 1.09 mmol) in toluene (0.3 mL). The viscous mixture was stirred at room temperature for 4 h. Water (20 mL) was added and the mixture was extracted with ethyl acetate (3x 20 mL). After removal of the solvent by distillation under reduced pressure from the combined organic phases, the residue was recrystallised from ethyl acetate (1.3 mL) and ethanol (8 drops) to give colourless crystals (110 mg, 0.502 mmol, 46%). M.p.: 153–154°C; IR (KBr): ṽ [cm^-1^] = 1739 (C = O, carboxylic acid), 1619 (C = O, lactam), 1596, 1210, 767; ^1^H-NMR (DMSO-*d*_6_, 400 MHz): δ (ppm) = 1.97–2.21 (m, 4H, 2x azepine-CH_2_), 2.84 (br s, 2H, azepine-CH_2_), 4.41 (s, 2H, *N*-CH_2_), 7.18 (td, *J* = 7.1 / 1.8 Hz, 1H, ArH), 7.23–7.35 (m, 3H, ArH), 12.73 (br s, 1H, COOH); ^13^C-NMR (DMSO-*d*_6_, 101 MHz): δ (ppm) = 28.3, 29.0, 32.4, 49.5 (CH_2_), 122.2, 126.0, 127.4, 129.3 (CH), 135.4, 142.4, 170.6, 171.9 (C); C_12_H_13_NO_3_ (219.24); CHN: calc. C 65.74, H 5.98, N 6.39, found C 65.47, H 5.91, N 6.29; APCI-MS (ASAP, +): m/z (%): 220 [M+H]^+^ (100), 202 [M-18]^+^ (15), 174 [M-46]^+^ (40), 146 [M-74]^+^ (62); HPLC (isocr.): 99.7% at 254 nm, t_M+S_ = 3.0 min, t_M_ (DMSO) = 1.2 min (ACN/buffer pH 2.7 30:70), λ_max_ [nm] = 238.

*tert-Butyl 2-(3*,*3-dimethyl-2-oxo-2*,*3*,*4*,*5-tetrahydro-1H-benzo[b]azepin-1-yl)acetate (15)*. According to general procedure 2 under nitrogen atmosphere. Potassium *tert*-butoxide (494 mg, 4.40 mmol) was added to a suspension of 3,3-dimethyl-1,3,4,5-tetrahydro-2*H*-benzo[*b*]azepin-2-one (**14**, 416 mg, 2.20 mmol) in tetrahydrofuran (44 mL) and stirred for 60 min. *tert*-butyl bromoacetate (0.353 mL, 2.42 mmol) was added and stirred for 9 h at room temperature. The solvent was distilled off and the remaining residue was dissolved in dichloromethane (50 mL) and washed with water. The aqueous phase was extracted with dichloromethane (3x 50 mL). Purification by column chromatography of the organic phase isolate (silica gel, eluent ethyl acetate/petroleum ether 1:4) yielded colourless crystals (333 mg, 1.10 mmol, 50%). M.p.: 122–123°C; IR (KBr): ṽ [cm^-1^] = 1748 (C = O, carboxylic acid ester), 1648 (C = O, lactam), 1366, 1227, 1157; ^1^H-NMR (chloroform-*d*, 300 MHz): δ (ppm) = 0.96 (s, 6H, 2x CH_3_), 1.45 (s, 9H, 3x CH_3_), 2.04 (t, *J* = 7.1 Hz, 2H, azepine-CH_2_), 2.88 (t, *J* = 7.1 Hz, 2H, azepine-CH_2_), 4.40 (s, 2H, *N*-CH_2_), 7.05 (dd, *J* = 8.1 / 1.4 Hz, 1H, ArH), 7.09–7.21 (m, 2H, ArH), 7.22–7.28 (m, 1H, ArH); ^13^C-NMR (chloroform-*d*, 76 MHz): δ (ppm) = 28.1 (3C), 28.4 (2C) (CH_3_), 30.5, 45.5, 53.1 (CH_2_), 121.6, 125.8, 127.6, 128.8 (CH), 42.0, 81.6, 136.1, 143.4, 168.6, 177.1 (C); C_18_H_25_NO_3_ (303.40); CHN: calc. C 71.26, H 8.31, N 4.62, found C 71.46, H 8.34, N 4.57; APCI-MS (ASAP, +): m/z (%): 248 [M-55]^+^ (100), 174 [M-129]^+^ (65), 164 [M-139]^+^ (51), 118 [M-185]^+^ (33); HPLC (grad.): 99.8% at 254 nm, t_M+S_ = 12.6 min, t_M_ (DMSO) = 1.2 min, λ_max_ [nm] = 242; HPLC (isocr.): 99.5% at 254 nm, t_M+S_ = 5.7 min, t_M_ (DMSO) = 1.0 min (ACN/water 60:40), λ_max_ [nm] = 242.

*2-(3*,*3-Dimethyl-2-oxo-2*,*3*,*4*,*5-tetrahydro-1H-benzo[b]azepin-1-yl)acetic acid (16)*. According to general procedure 3 under nitrogen from *tert*-butyl 2-(3,3-dimethyl-2-oxo-2,3,4,5-tetrahydro-1*H*-benzo[*b*]azepin-1-yl)acetate (**15**, 243 mg, 0.800 mmol) in dry dichloromethane (20 mL) with trifluoroacetic acid (2.5 mL, 33 mmol) at room temperature (22 h). The solvent was removed under vacuum and the residue crystallised in the desiccator overnight. The colorless crystals were washed with petroleum ether, mortared and dried (60°C, 2 h) (151 mg, 0.611 mmol, 76%). M.p.: 122–123°C; IR (KBr): ṽ [cm^-1^] = 1741 (C = O, carboxylic acid), 1616 (C = O, lactam), 1594, 1394, 1247; ^1^H-NMR (DMSO-*d*_6_, 600 MHz): δ (ppm) = 0.84 (br s, 6H, 2x CH_3_), 1.95 (t, *J* = 7.1 Hz, 2H, azepine-CH_2_), 3.34 (br s, 2H, azepine-CH_2_), 4.40 (s, 2H, *N*-CH_2_), 7.14 (td, *J* = 7.4 / 1.2 Hz, 1H, ArH), 7.19 (dd, *J* = 8.0 / 1.2 Hz, 1H, ArH), 7.25 (dd, *J* = 7.5 / 1.6 Hz, 1H, ArH), 7.29 (ddd, *J* = 8.1 / 7.3 / 1.6 Hz, 1H, ArH), 12.63 (br s, 1H, COOH, complete H/D exchange); ^13^C-NMR (DMSO-*d*_6_, 151 MHz): δ (ppm) = 28.0 (2C, br) (CH_3_), 29.5, 45.0, 51.8 (CH_2_), 121.9, 125.6, 127.5, 128.5 (CH), 41.3, 135.6, 143.0, 170.7, 175.9 (C); C_14_H_17_NO_3_ (247.29); CHN: calc. C 68.00, H 6.93, N 5.66, found C 68.18, H 6.97, N 5.71; APCI-MS (ASAP, +): m/z (%): 248 [M+H]^+^ (100), 220 [M-27]^+^ (27), 174 [M-73]^+^ (45), 164 [M-83]^+^ (73); HPLC (isocr.): 99.8% at 254 nm, t_M+S_ = 5.7 min, t_M_ (DMSO) = 1.2 min (ACN/buffer pH 2.7 35:65), λ_max_ [nm] = 242.

*tert-Butyl 2-(6-oxo-6*,*7-dihydro-5H-dibenzo[b*,*d]azepin-5-yl)acetate (18)*. According to a procedure from Nelissen *et al*. [57] under nitrogen atmosphere. A suspension of 5,7-dihydro-6*H*-dibenzo[*b*,*d*]azepin-6-one (**17**, 3.610 g, 17.25 mmol) in DMF (10 mL) was cooled to 0°C. Potassium *tert*-butoxide (3.872 g, 34.50 mmol) in DMF (10 mL) was added. After 30 min *tert*-butyl bromoacetate (2.55 mL, 17.3 mmol) was added dropwise (1 h). The mixture was stirred at room temperature (21 h), then heated to 40°C and water (40 mL) was added in portions (1 h). The precipitate was filtered off and the filtrate was extracted with ethyl acetate (3x 100 mL). The organic phase was subjected to reduced pressure until a yellow solid remained. The two solids were combined and purified by column chromatography (silica gel, eluent ethyl acetate/petroleum ether 1:2 → 1:0). The obtained brown oil (1.07 g) crystallised in the desiccator and was dried (60°C, 2 h) to form a yellow solid (0.926 mg, 2.86 mmol, 17%). M.p.: 93–95°C; IR (KBr): ṽ [cm^-1^] = [1720, 1665] (C = O), 1368, 1157, 767; ^1^H-NMR (DMSO-*d*_6_, 500 MHz): δ (ppm) = 1.26 (s, 9H, 3x CH_3_), 3.36 (d, *J* = 12.6 Hz, 1H, azepine-CH_2_), 3.54 (d, *J* = 12.7 Hz, 1H, azepine-CH_2_), 4.30 (d, *J* = 17.1 Hz, 1H, *N*-CH_2_, part B of an AB system), 4.33 (d, *J* = 17.1 Hz, 1H, *N*-CH_2_, part A of an AB system), 7.33–7.48 (m, 5H, ArH), 7.48–7.55 (m, 1H, ArH), 7.60–7.69 (m, 2H, ArH); ^13^C-NMR (DMSO-*d*_6_, 126 MHz): δ (ppm) = 27.4 (3C) (CH_3_), 41.0, 51.4 (CH_2_), 122.5, 125.5, 127.6, 127.7, 128.1, 128.5, 128.7, 129.9 (CH), 80.7, 133.2, 134.8, 136.1, 140.3, 167.9, 170.1 (C); C_20_H_21_NO_3_ (323.39); CHN: calc. C 74.28, H 6.55, N 4.33, found C 73.97, H 6.58, N 4.14; APCI-MS (ASAP, +, soft): m/z (%): 324 [M+H]^+^ (5), 268 [M-55]^+^ (100); HPLC (grad.): 98.9% at 254 nm, t_M+S_ = 11.8 min, t_M_ (DMSO) = 1.2 min, λ_max_ [nm] = 234; HPLC (isocr.): 98.6% at 254 nm, t_M+S_ = 4.0 min, t_M_ (DMSO) = 1.4 min (ACN/water 60:40), λ_max_ [nm] = 236.

*2-(6-Oxo-6*,*7-dihydro-5H-dibenzo[b*,*d]azepin-5-yl)acetic acid (19)*. According to general procedure 3 under argon from *tert*-butyl 2-(6-oxo-6,7-dihydro-5*H*-dibenzo[*b*,*d*]azepin-5-yl)acetate (**18**, 898 mg, 2.78 mmol) in dry dichloromethane (35 mL) and trifluoroacetic acid (9.0 mL, 0.12 mol) at room temperature (23 h). The solvent was evaporated under vacuum to obtain a residue that crystallised upon storage in the desiccator. The beige crystals were washed with acetone and dried (4 h, 60°C) (644 mg, 2.41 mmol, 87%). M.p.: 219–221°C; IR (KBr): ṽ [cm^-1^] = 1734 (C = O, carboxylic acid), 1621 (C = O, lactam), 1594 (C = C, Ar), 1443, 1417, 1387, 1201, 1164, 764; ^1^H-NMR (DMSO-*d*_6_, 500 MHz): δ (ppm) = 3.37 (d, *J* = 12.6 Hz, 1H, azepine-CH_2_), 3.54 (d, *J* = 12.7 Hz, 1H, azepine-CH_2_), 4.22 (d, *J* = 17.5 Hz, 1H, *N*-CH_2_, part B of an AB system), 4.36 (d, *J* = 17.5 Hz, 1H, *N*-CH_2_, part A of an AB system), 7.33–7.53 (m, 6H, ArH), 7.59–7.68 (m, 2H, ArH), 12.79 (br s, 1H, COOH); ^13^C-NMR (DMSO-*d*_6_, 126 MHz): δ (ppm) = 41.1, 50.7 (CH_2_), 122.5, 125.5, 127.6 (2C, isochronous by coincidence), 128.1, 128.5, 128.7, 129.8 (CH), 133.0, 134.8, 136.1, 140.5, 170.1, 170.4 (C); C_16_H_13_NO_3_ (267.28); HRMS (ESI, +): m/z [M+H]^+^ calc. 268.09682, found 268.09699; APCI-MS (ASAP, -): m/z (%): 266 [M-H]^-^ (28), 222 [M-45]^-^ (42), 166 [M-101]^-^ (100); ESI-MS (DI, +): m/z (%): 290 [M+Na]^+^ (100); HPLC (isocr.): 99.0% at 254 nm, t_M+S_ = 6.9 min, t_M_ (DMSO) = 1.1 min (ACN/buffer pH 2.7 30:70), λ_max_ [nm] = 231.

*tert-Butyl (E/Z)-2-(5-(methoxyimino)-2-oxo-2*,*3*,*4*,*5-tetrahydro-1H-benzo[b]azepin-1-yl)acetate (21)*. According to general procedure 2 under nitrogen atmosphere, potassium *tert*-butoxide (7.27 g, 64.8 mmol) was added to a suspension of (*E/Z*)-5-methoxyimino-1,3,4,5-tetrahydro-2*H*-benzo[*b*]azepin-2-one (**20**, 5.53 g, 27.1 mmol) in dry tetrahydrofuran (200 mL). The orange suspension was stirred for 1 h at room temperature. Then, *tert*-butyl bromoacetate (4.4 mL, 30 mmol) was added and stirring was continued for 95 h at room temperature. After 21 h, THF (200 mL) was added and after 26 h *tert*-butyl bromoacetate (4.4 mL, 30 mmol) was added. The solvent was removed and the brown residue was taken up in ethyl acetate (150 mL) and washed with water (2x 100 mL). Sodium sulfate decahydrate was added for salting-out. The resulting brown liquid (12.04 g) was purified by column chromatography (silica gel, eluent ethyl acetate/petroleum ether 1:2). A yellow oil was obtained (7.41 g), which was treated with several portions of petroleum ether after storage in a desiccator and onset of crystallisation. After drying, at 60°C for 1 h at reduced pressure and in the desiccator for 3 days, a colourless solid was obtained (6.272 g, 19.70 mmol, 73%). M.p.: 75–76°C; IR (KBr): ṽ [cm^-1^] = 1740 (C = O), 1673 (C = O), 1369, 1224, 1154, 1047; ^1^H-NMR (DMSO-*d*_6_, 400 MHz): δ (ppm) = 1.36 (s, 9H, 3x CH_3_), 2.47 (br s, 2H, azepine-CH_2_), 2.94 (br s, 2H, azepine-CH_2_), 3.91 (s, 3H, *O*-CH_3_), 4.32 (s, 2H, *N*-CH_2_), 7.23–7.32 (m, 2H, ArH), 7.41 (dd, *J* = 7.9 / 1.7 Hz, 1H, ArH), 7.46–7.55 (m, 1H, ArH); ^13^C-NMR (DMSO-*d*_6_, 101 MHz): δ (ppm) = 27.5 (3C), 61.8 (CH_3_), 29.2, 30.5, 51.1 (CH_2_), 122.9, 125.9, 129.4, 129.6 (CH), 81.0, 130.6, 141.3, 157.1, 167.8, 171.4 (C); C_17_H_22_N_2_O_4_ (318.37); CHN: calc. C 64.13, H 6.97, N 8.80, found C 64.14, H 6.86, N 8.67; APCI-MS (ASAP, +): m/z (%): 319 [M+H]^+^ (21), 263 [M-55]^+^ (82); 231 [M-87]^+^ (100); HPLC (grad.): 99.7% at 254 nm, t_M+S_ = 10.7 min, t_M_ (DMSO) = 1.2 min, λ_max_ [nm] = 221, 238; HPLC (isocr.): 99.9% at 254 nm, t_M+S_ = 4.5 min, t_M_ (DMSO) = 1.5 min (ACN/water 50:50), λ_max_ [nm] = 224.

*(E/Z)-2-(5-(Methoxyimino)-2-oxo-2*,*3*,*4*,*5-tetrahydro-1H-benzo[b]azepin-1-yl)acetic acid (22)*. According to general procedure 3 under nitrogen from *tert*-butyl (E/Z)-2-(5-(methoxyimino)-2-oxo-2,3,4,5-tetrahydro-1*H*-benzo[*b*]azepin-1-yl)acetate (**21**, 6.129 g, 19.25 mmol) in dry dichloromethane (200 mL) with trifluoroacetic acid (59 mL, 0.77 mol) at room temperature (8.5 h). The solvent was removed under vacuum and the light brown oil was mixed with water (5 mL) and sodium hydroxide solution was added drop-wise (8% w/w). A pale yellow precipitate formed, which was vacuum filtered and dried at reduced pressure at 60°C for 4 h (4.588 g, 17.49 mmol, 91%). M.p.: 180–181°C; IR (KBr): ṽ [cm^-1^] = 3217 (O-H), 1730 (C = O), 1663 (C = O), 1048, 754; ^1^H-NMR (DMSO-*d*_6_, 400 MHz): δ (ppm) = 2.48 (br s, 2H, azepine-CH_2_), 2.95 (br s, 2H, azepine-CH_2_), 3.91 (s, 3H, *O*-CH_3_), 4.32 (s, 2H, *N*-CH_2_), 7.23–7.33 (m, 2H, ArH), 7.40 (dd, *J* = 7.6 / 1.7 Hz, 1H, ArH), 7.46–7.54 (m, 1H, ArH), 12.78 (br s, 1H, COOH); ^13^C-NMR (DMSO-*d*_6_, 101 MHz): δ (ppm) = 61.8 (CH_3_), 29.2, 30.6, 50.4 (CH_2_), 122.9, 125.9, 129.5, 130.6 (CH), 129.4, 141.5, 157.1, 170.3, 171.5 (C); C_13_H_14_N_2_O_4_ (262.27); CHN: calc. C 59.54, H 5.38, N 10.68, found C 59.92, H 5.37, N 10.25; APCI-MS (ASAP, -, soft): m/z (%): 261 [M-H]^-^ (100); HPLC (isocr.): 98.3% at 254 nm, t_M+S_ = 6.3 min, t_M_ (DMSO) = 1.2 min (ACN/buffer pH 2.7 20:80), λ_max_ [nm] = 223.

## Supporting information

S1 File Figs 1–42. ^1^H-NMR spectra of benzazepinones and paullones tested in biological assays. (PDF)

S2 File Figs 1–11. ^1^H-NMR spectra of selected compounds after H/D-exchange. (PDF)

S3 File HPLC chromatograms of all biologically evaluated compounds presented in this paper. (ZIP)

S4 File Acquisition data and fids for the ^1^H-NMR experiments of all biologically evaluated compounds presented in this paper. (ZIP)

S5 File Acquisition data and fids for ^1^H-NMR experiments after H/D-exchange by D_2_O treatment of selected compounds presented in this paper. (ZIP)

S1 Table Physicochemical properties, water solubility and pharmacokinetics predicted data for benzazepinones and paullones tested in biological assays. (PDF)

S1 Graphical abstract (TIF)
