# Supplementary material for: The indole motif is essential for the antitrypanosomal activity of N5-substituted paullones
Source: PLoS One. 2023 Nov 30;18(11):e0292946. doi: 10.1371/journal.pone.0292946 (PMC10688702; doi:10.1371/journal.pone.0292946)
Supplement: S1 File — (PDF) [file pone.0292946.s001.pdf]

The indole motif is essential for the antitrypanosomal activity of N^5-substituted paullones

Irina Ihnatenko, Marco J. Müller, Oliver C. F. Orban, Jens C. Lindhof, Diego Benítez, Cecilia Ortíz, Estefanía Dibello, Leonardo L. Seidl, Marcelo A. Comini and Conrad Kunick*

S1 File. Figs 1–42. ^{1}H-NMR spectra of benzazepinones and paullones tested in biological assays.

Table of contents: ^{1}H-NMR spectra of benzazepinones and paullones tested in biological assays.

S1 File. Fig 1. ^{1}H-NMR spectrum of compound 1f.......................... 3

S1 File. Fig 2. ^{1}H-NMR spectrum of compound 1g.......................... 4

S1 File. Fig 3. ^{1}H-NMR spectrum of compound 1h.......................... 5

S1 File. Fig 4. ^{1}H-NMR spectrum of compound 2b.......................... 6

S1 File. Fig 5. ^{1}H-NMR spectrum of compound 2c.......................... 7

S1 File. Fig 6. ^{1}H-NMR spectrum of compound 2d.......................... 8

S1 File. Fig 7. ^{1}H-NMR spectrum of compound 2e.......................... 9

S1 File. Fig 8. ^{1}H-NMR spectrum of compound 2f..........................10

S1 File. Fig 9. ^{1}H-NMR spectrum of compound 2g.........................11

S1 File. Fig 10. ^{1}H-NMR spectrum of compound 2h......................12

S1 File. Fig 11. ^{1}H-NMR spectrum of compound 2i.......................13

S1 File. Fig 12. ^{1}H-NMR spectrum of compound 2j.......................14

S1 File. Fig 13. ^{1}H-NMR spectrum of compound 2k......................15

S1 File. Fig 14. ^{1}H-NMR spectrum of compound 2o......................16

S1 File. Fig 15. ^{1}H-NMR spectrum of compound 2p..................... 17

S1 File. Fig 16. ^{1}H-NMR spectrum of compound 2q..................... 18

S1 File. Fig 17. ^{1}H-NMR spectrum of compound 2r...................... 19

S1 File. Fig 18. ^{1}H-NMR spectrum of compound 2s..................... 20

S1 File. Fig 19. ^{1}H-NMR spectrum of compound 2t...................... 21

S1 File. Fig 20. ^{1}H-NMR spectrum of compound 2u..................... 22

S1 File. Fig 21. ^{1}H-NMR spectrum of compound 2v..................... 23

S1 File. Fig 22 ^{1}H-NMR spectrum of compound 3b...................... 24

S1 File. Fig 23. ^{1}H-NMR spectrum of compound 3c..................... 25

S1 File. Fig 24. ^{1}H-NMR spectrum of compound 3d..................... 26

S1 File. Fig 25. ^{1}H-NMR spectrum of compound 3e..................... 27

S1 File. Fig 26. ^{1}H-NMR spectrum of compound 4b..................... 28

S1 File. Fig 27. ^{1}H-NMR spectrum of compound 4c..................... 29

S1 File. Fig 28. ^{1}H-NMR spectrum of compound 4d..................... 30

S1 File. Fig 29. ^{1}H-NMR spectrum of compound 5b. 31

S1 File. Fig 30. ^{1}H-NMR spectrum of compound 5c......................32

S1 File. Fig 31. ^{1}H-NMR spectrum of compound 5d. 33

S1 File. Fig 32. ^{1}H-NMR spectrum of compound 9a. 34

S1 File. Fig 33. ^{1}H-NMR spectrum of compound 9b. 35

S1 File. Fig 34. ^{1}H-NMR spectrum of compound 9c......................36

S1 File. Fig 35. ^{1}H-NMR spectrum of compound 10a. 37

S1 File. Fig 36. ^{1}H-NMR spectrum of compound 10b. 38

S1 File. Fig 37. ^{1}H-NMR spectrum of compound 10c....................39

S1 File. Fig 38. ^{1}H-NMR spectrum of compound 18. 40

S1 File. Fig 39. ^{1}H-NMR spectrum of compound 19. 41

S1 File. Fig 40. ^{1}H-NMR spectrum of compound 20. 42

S1 File. Fig 41. ^{1}H-NMR spectrum of compound 21. 43

S1 File. Fig 42. ^{1}H-NMR spectrum of compound 22. 44

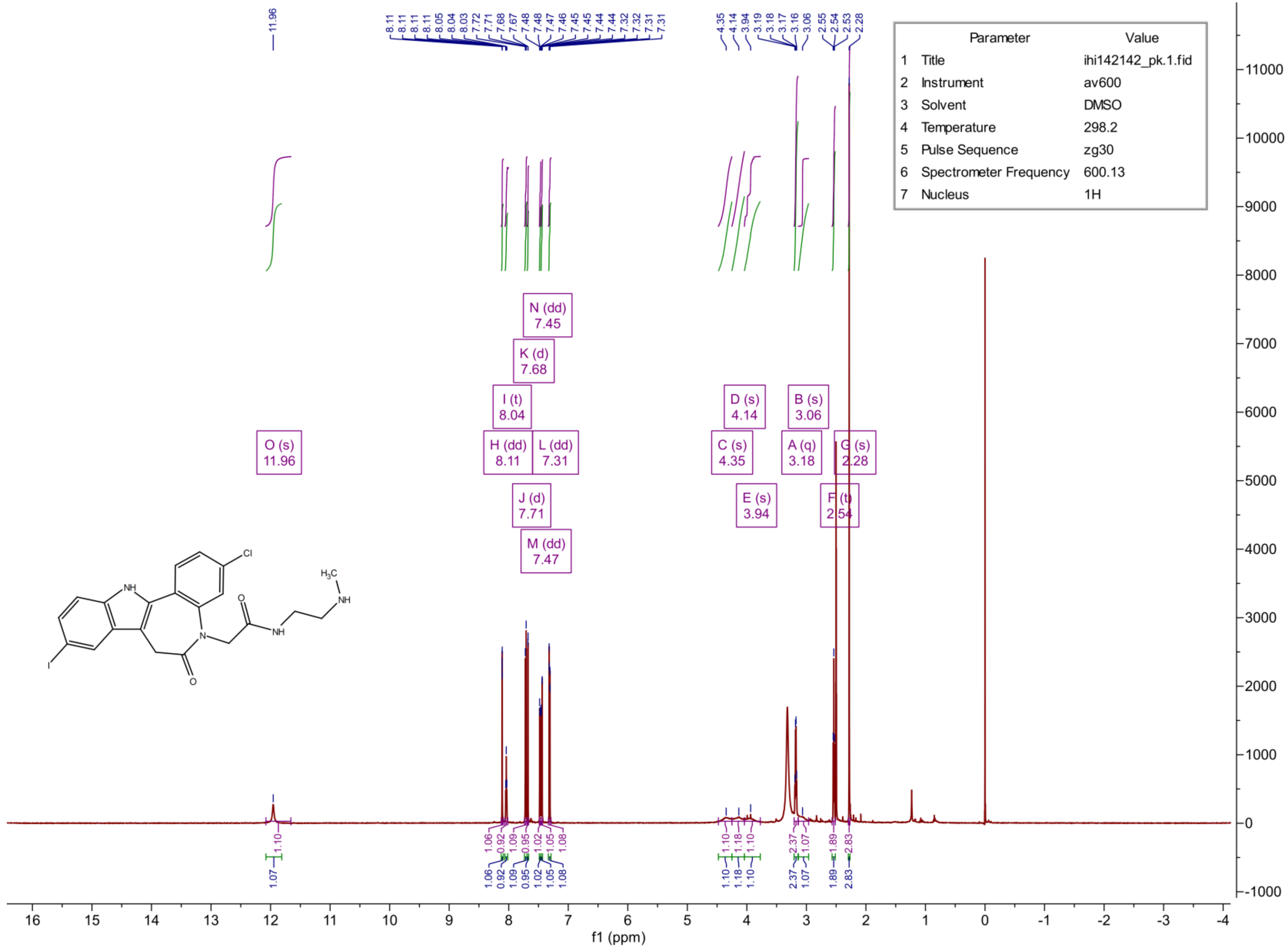

S1 File. Fig 1. ^{1}H-NMR spectrum of compound 1f.

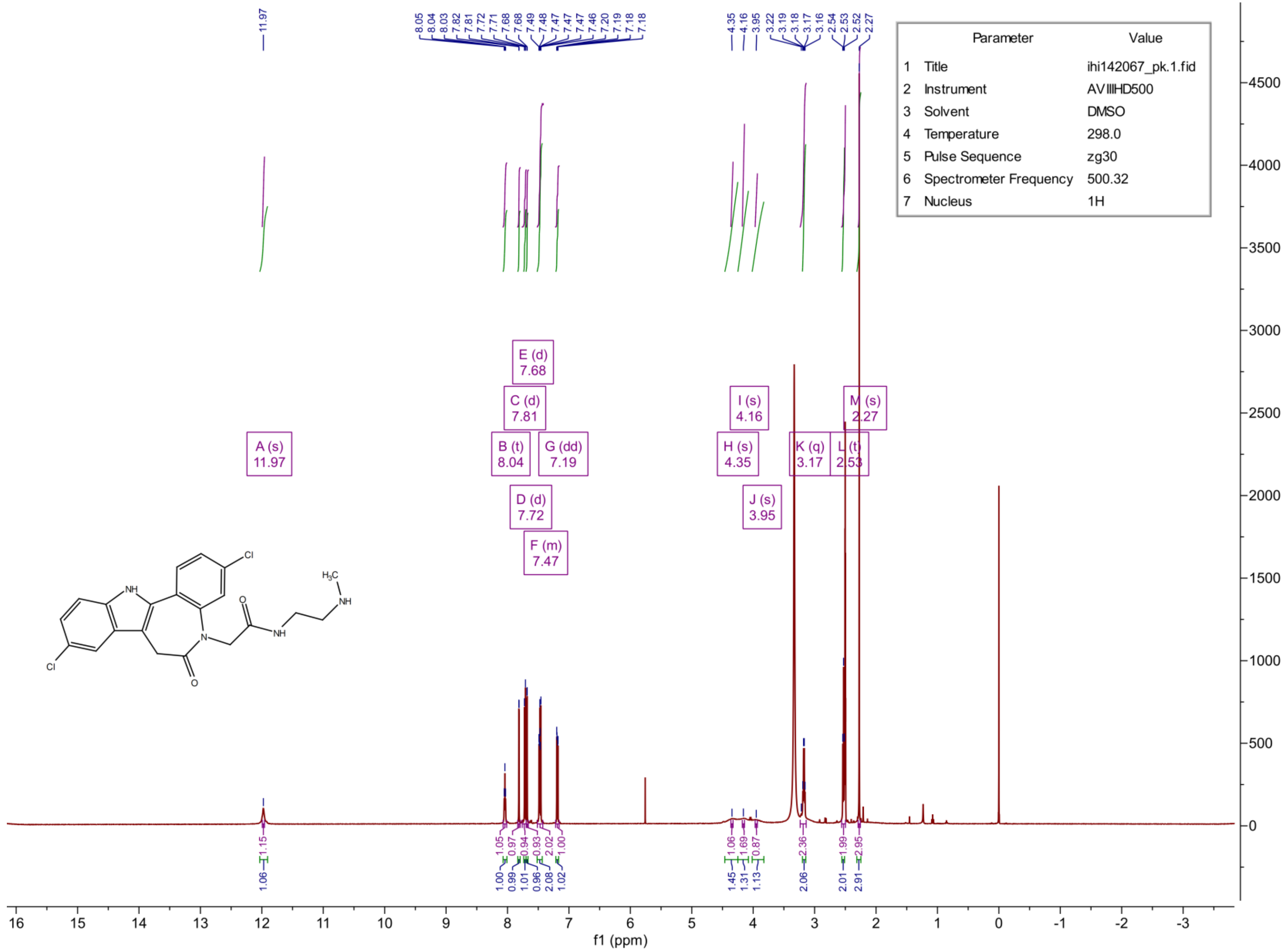

S1 File. Fig 2. ^{1}H-NMR spectrum of compound 1g.

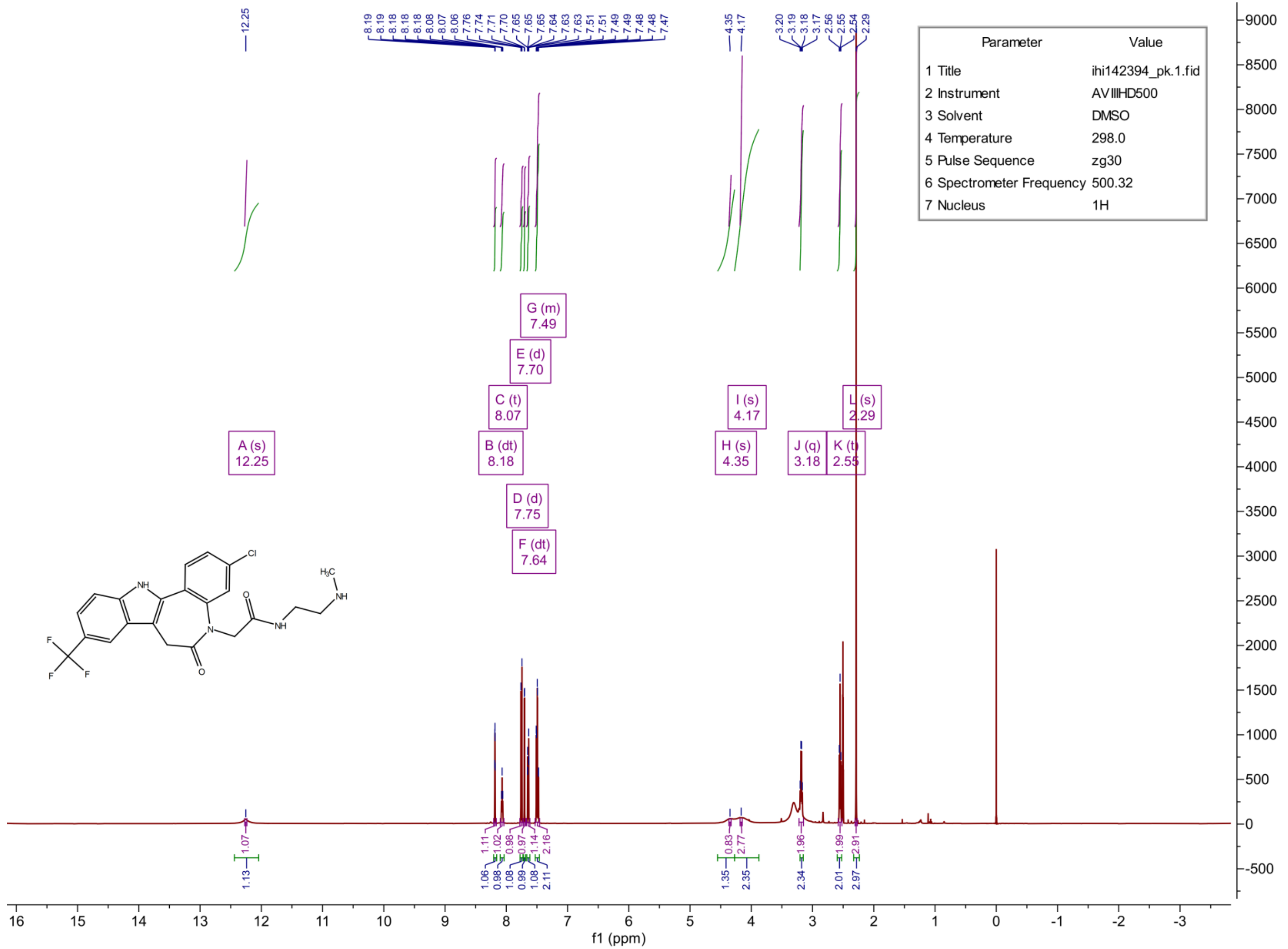

S1 File. Fig 3. ^{1}H-NMR spectrum of compound 1h.

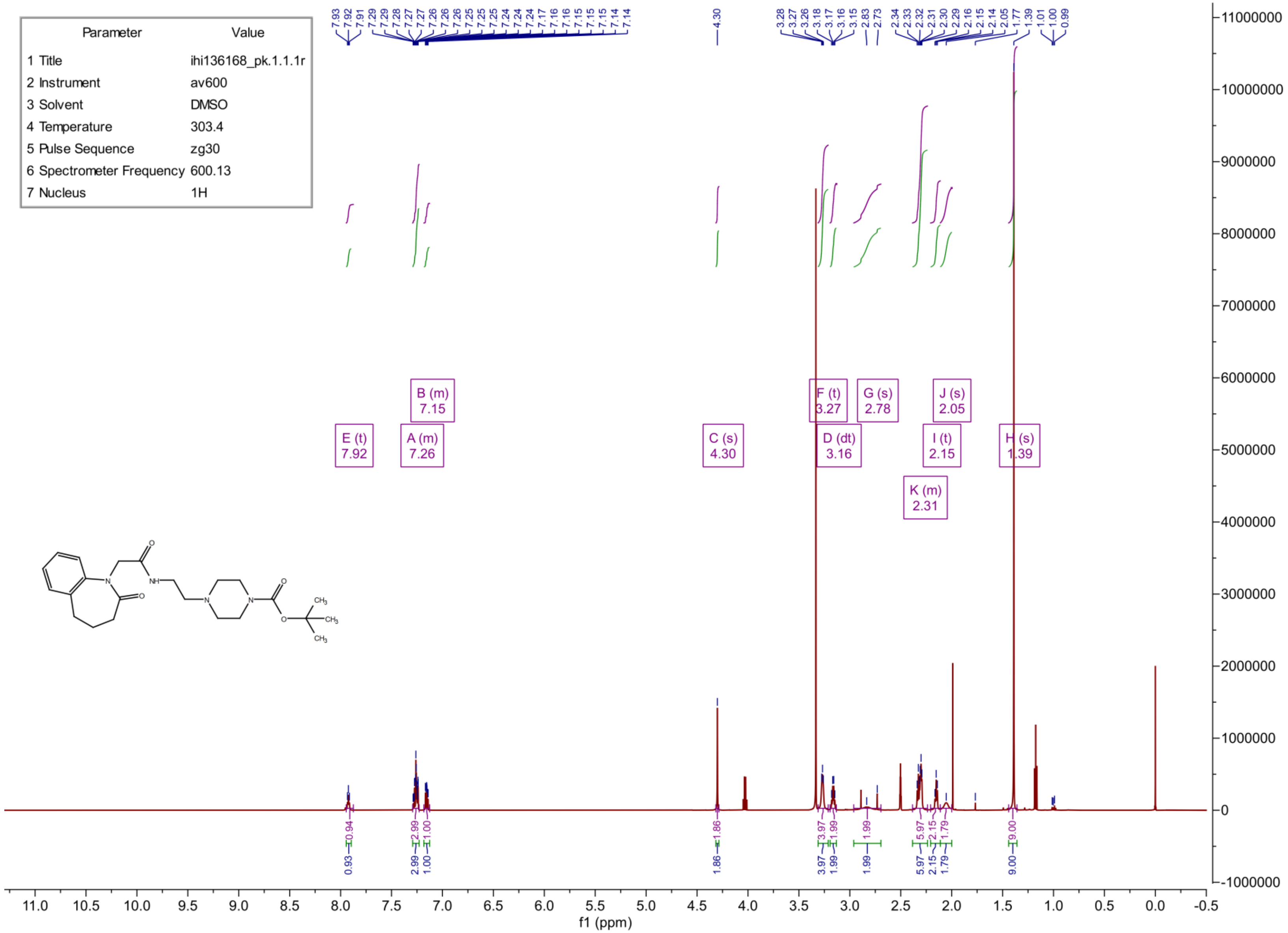

S1 File. Fig 4. ^{1}H-NMR spectrum of compound 2b.

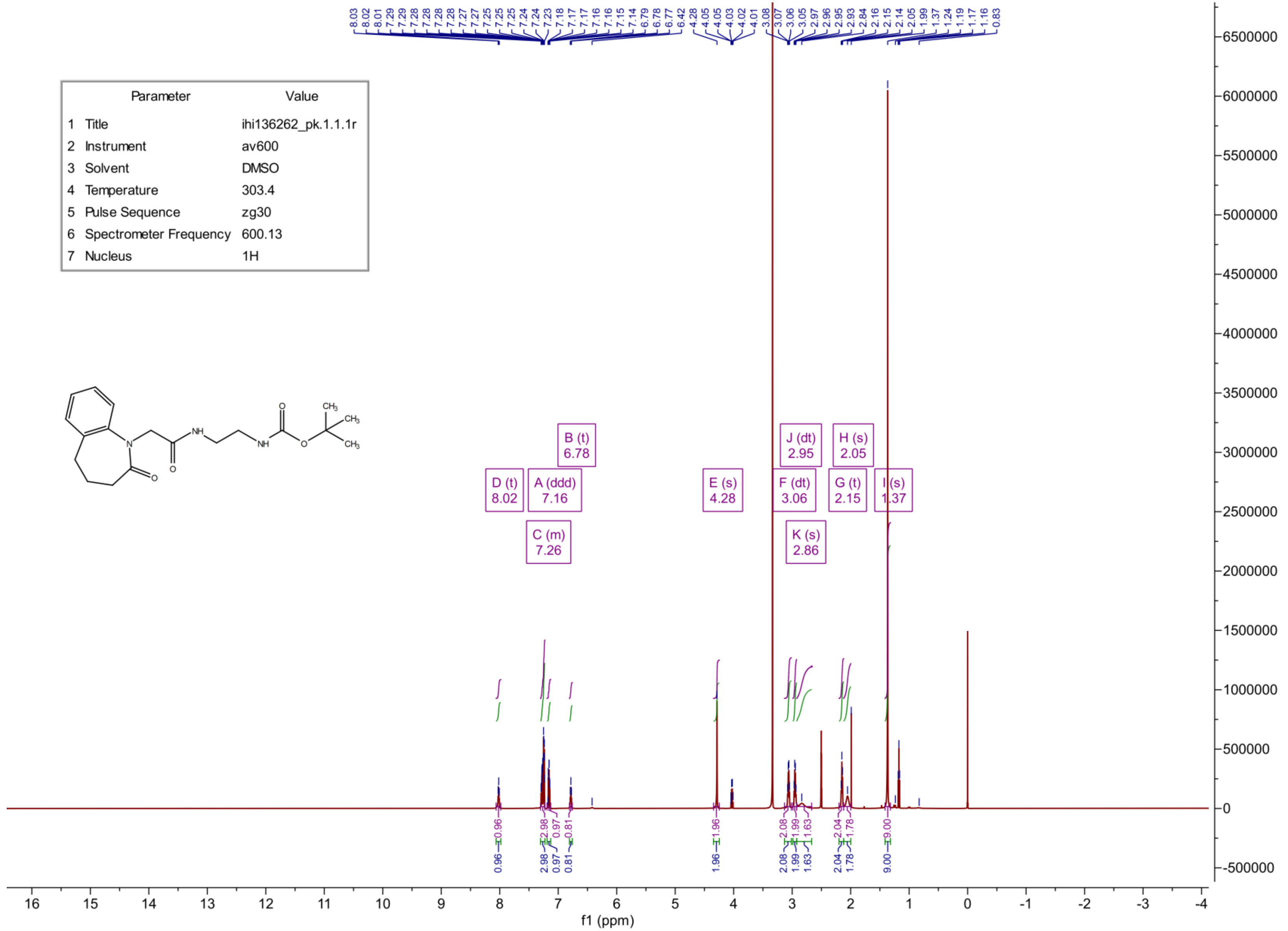

S1 File. Fig 5. ^{1}H-NMR spectrum of compound 2c.

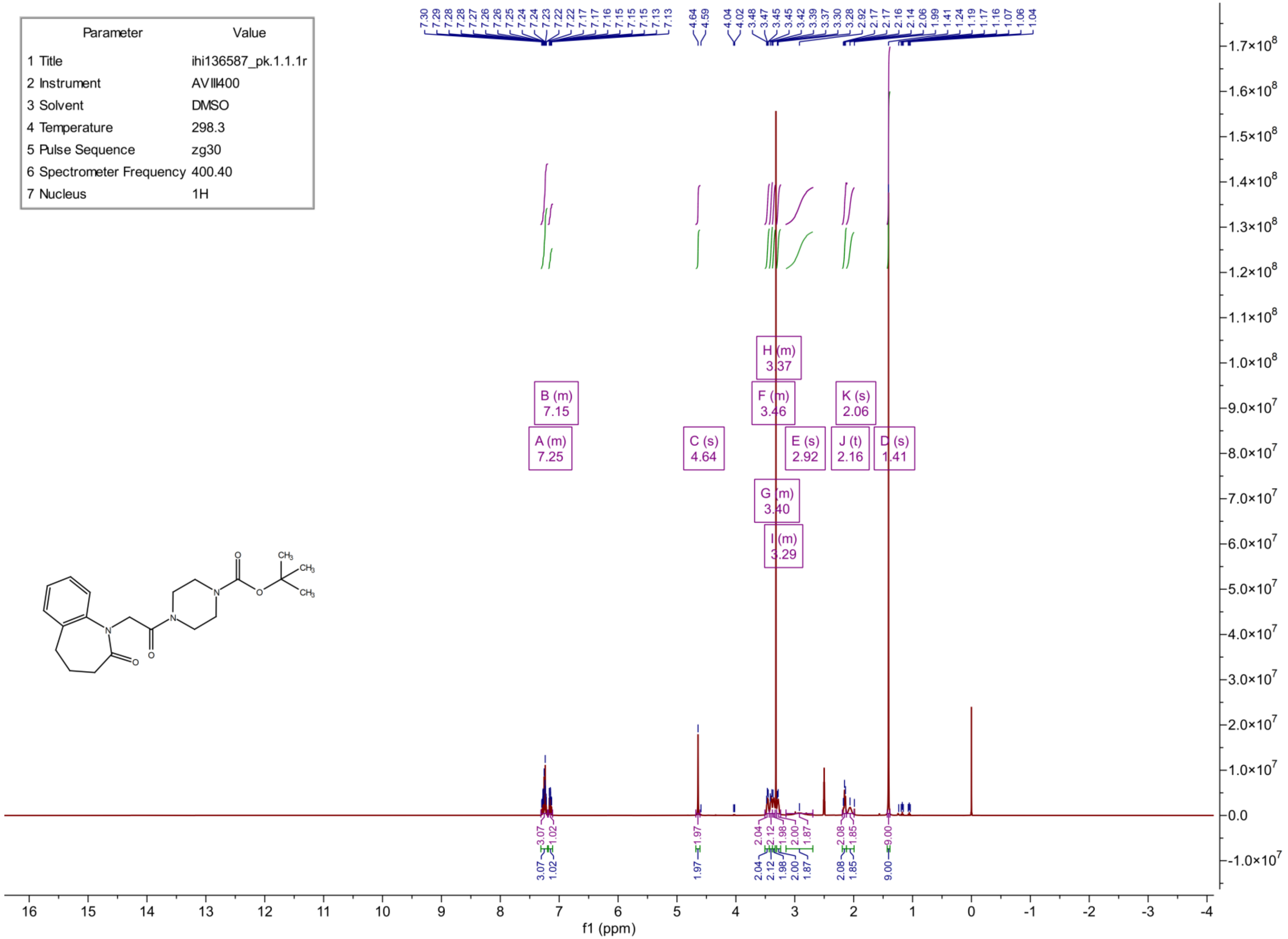

S1 File. Fig 6. ^{1}H-NMR spectrum of compound 2d.

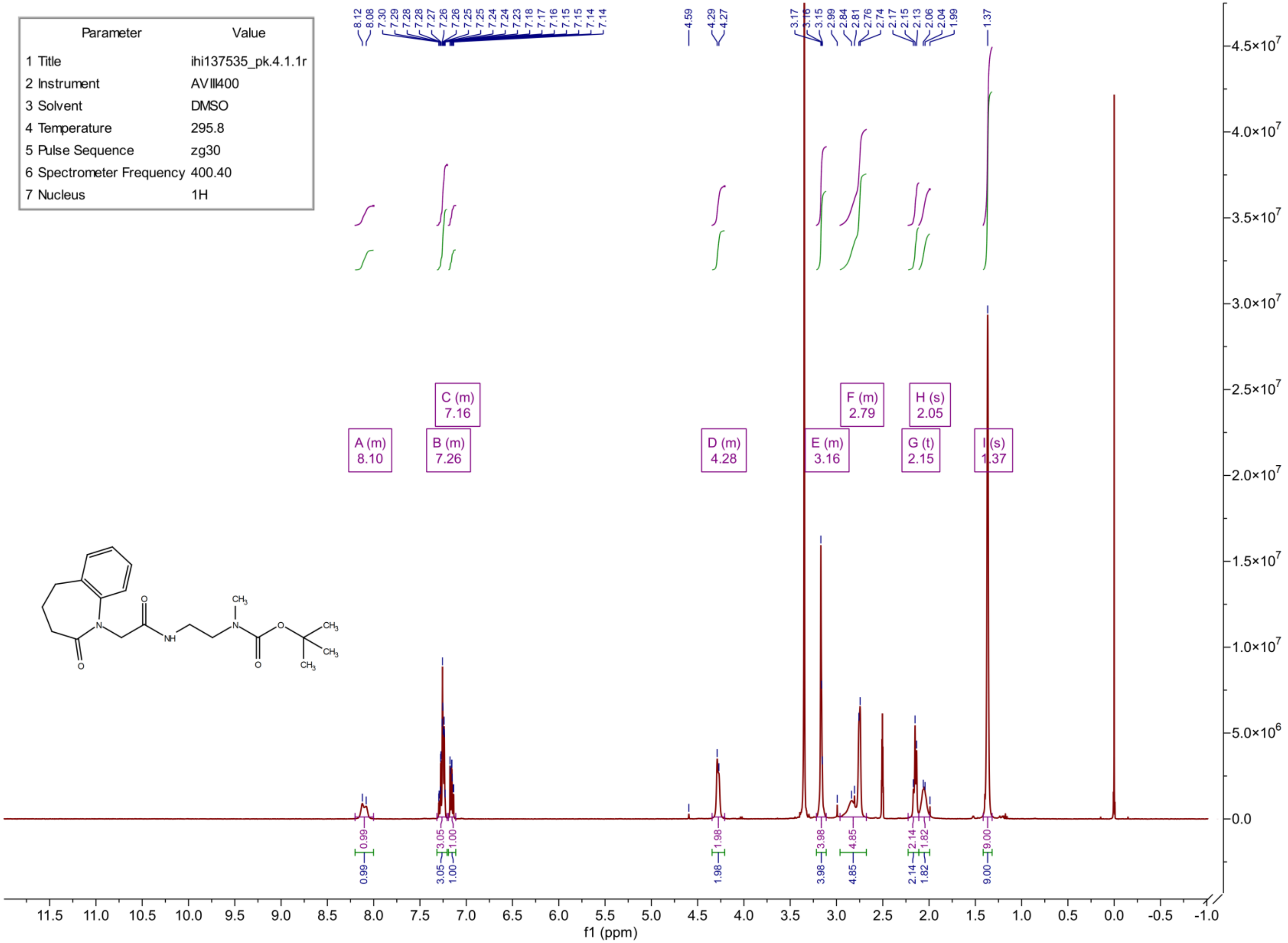

S1 File. Fig 7. ^{1}H-NMR spectrum of compound 2e.

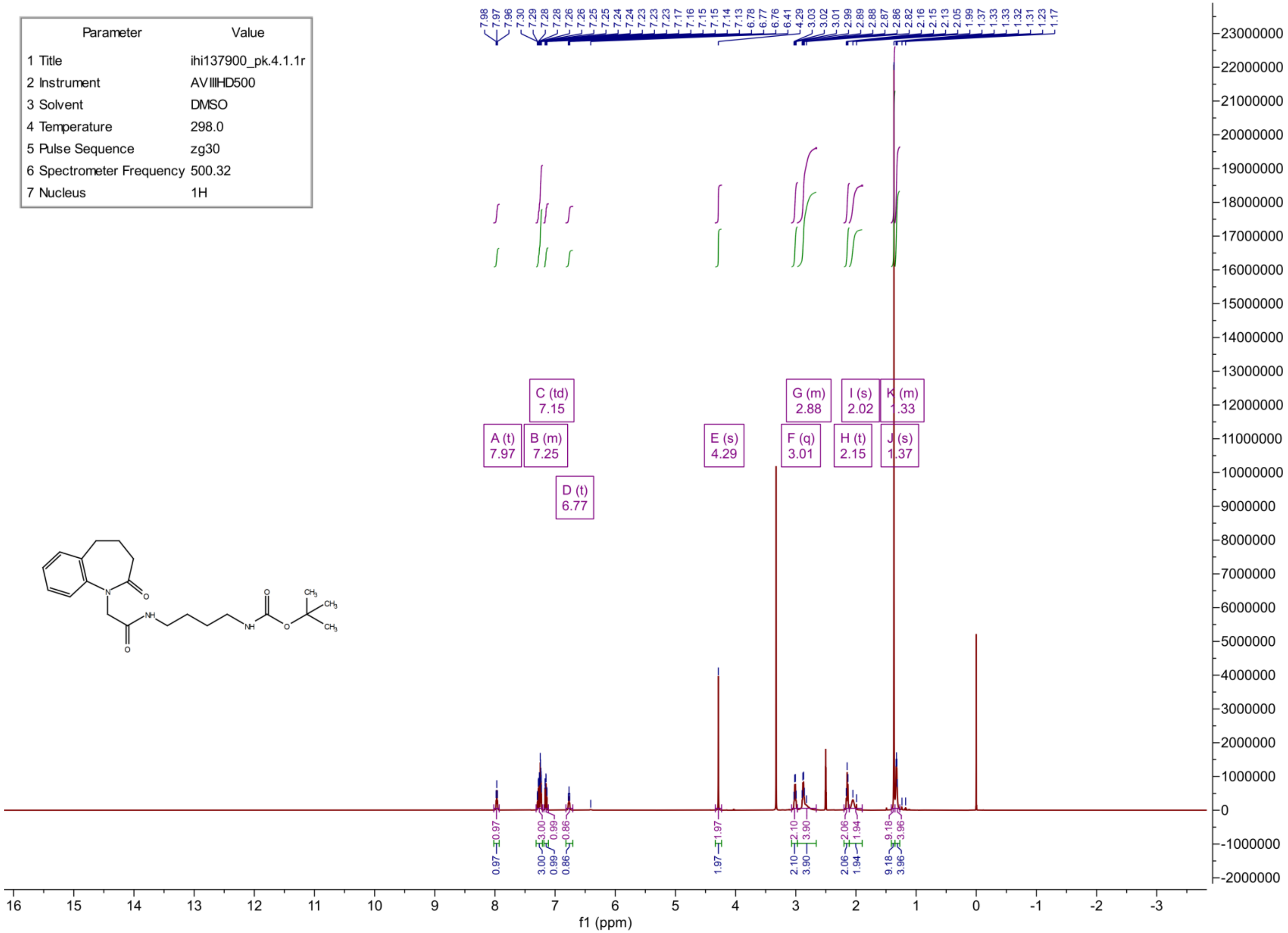

S1 File. Fig 8. ^{1}H-NMR spectrum of compound 2f.

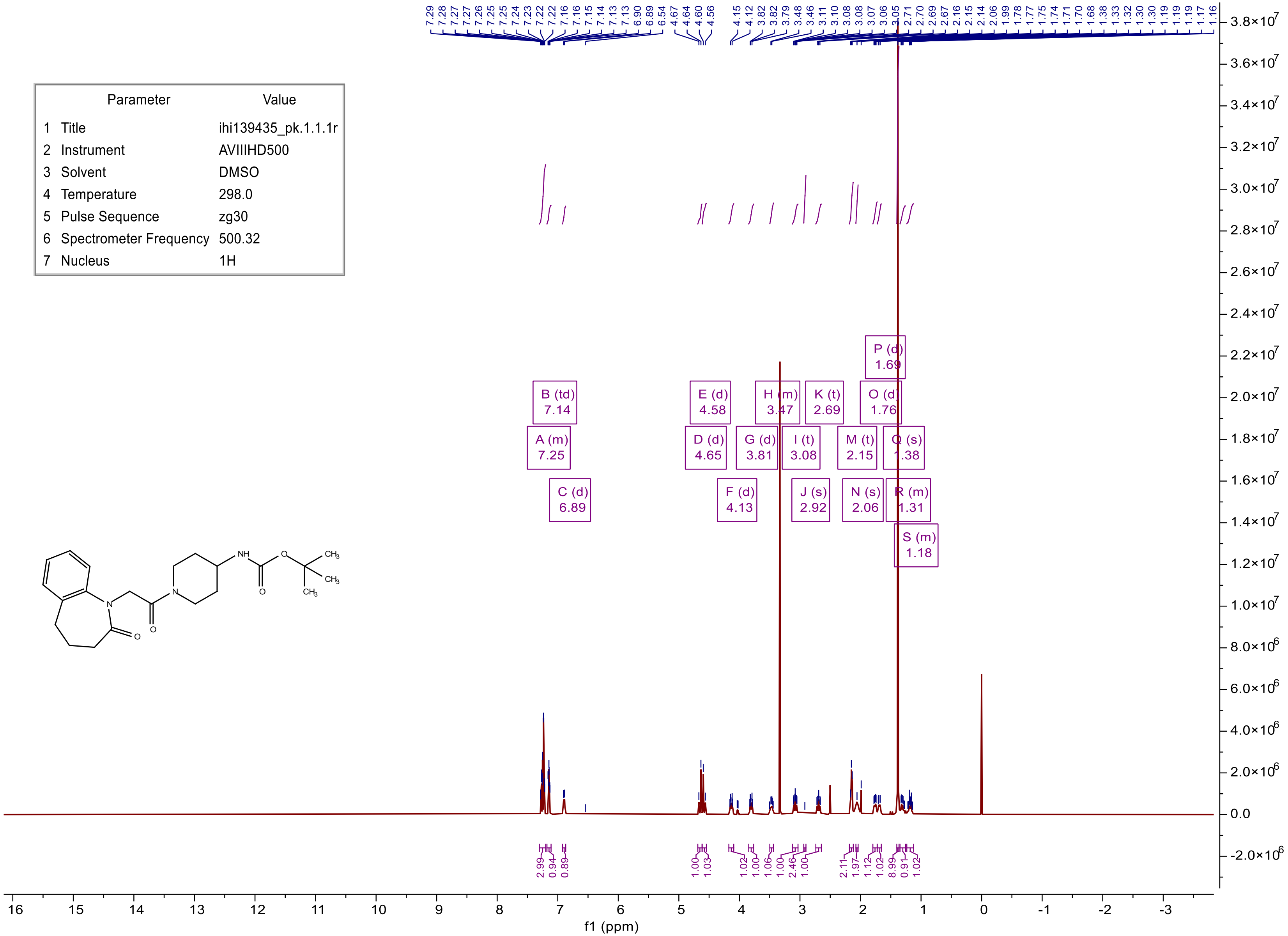

	Parameter	Value
1	Title	ihi139435_pk.1.1.1r
2	Instrument	AVIIIHD500
3	Solvent	DMSO
4	Temperature	298.0
5	Pulse Sequence	zg30
6	Spectrometer Frequency	500.32
7	Nucleus	1H

S1 File. Fig 9. ^{1}H-NMR spectrum of compound 2g.

	Parameter	Value
1	Title	ihi136368_pk.1.1.1r
2	Instrument	AVIIIHD500
3	Solvent	DMSO
4	Temperature	298.0
5	Pulse Sequence	zg30
6	Spectrometer Frequency	500.32
7	Nucleus	1H

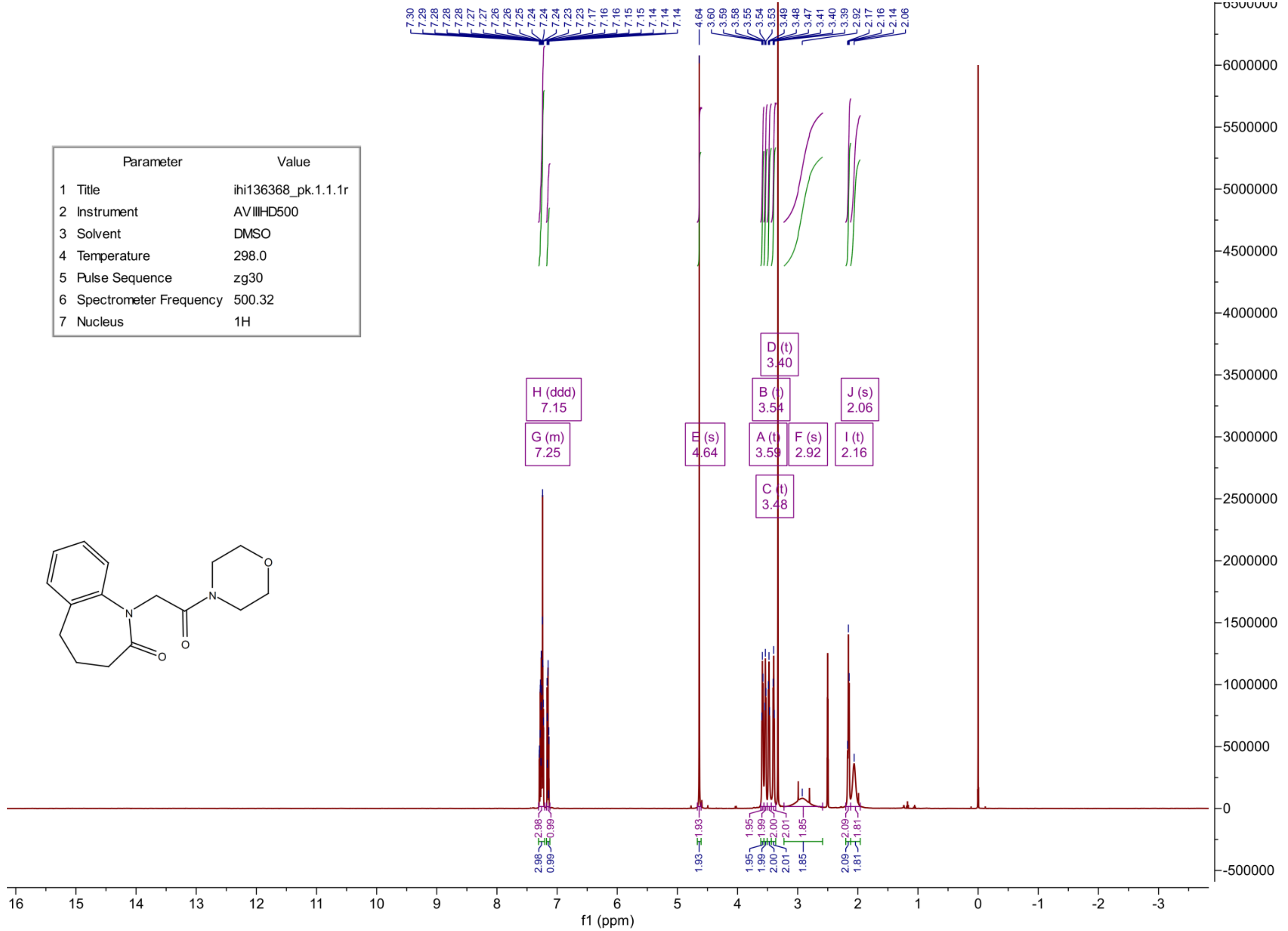

S1 File. Fig 10. ^{1}H-NMR spectrum of compound 2h.

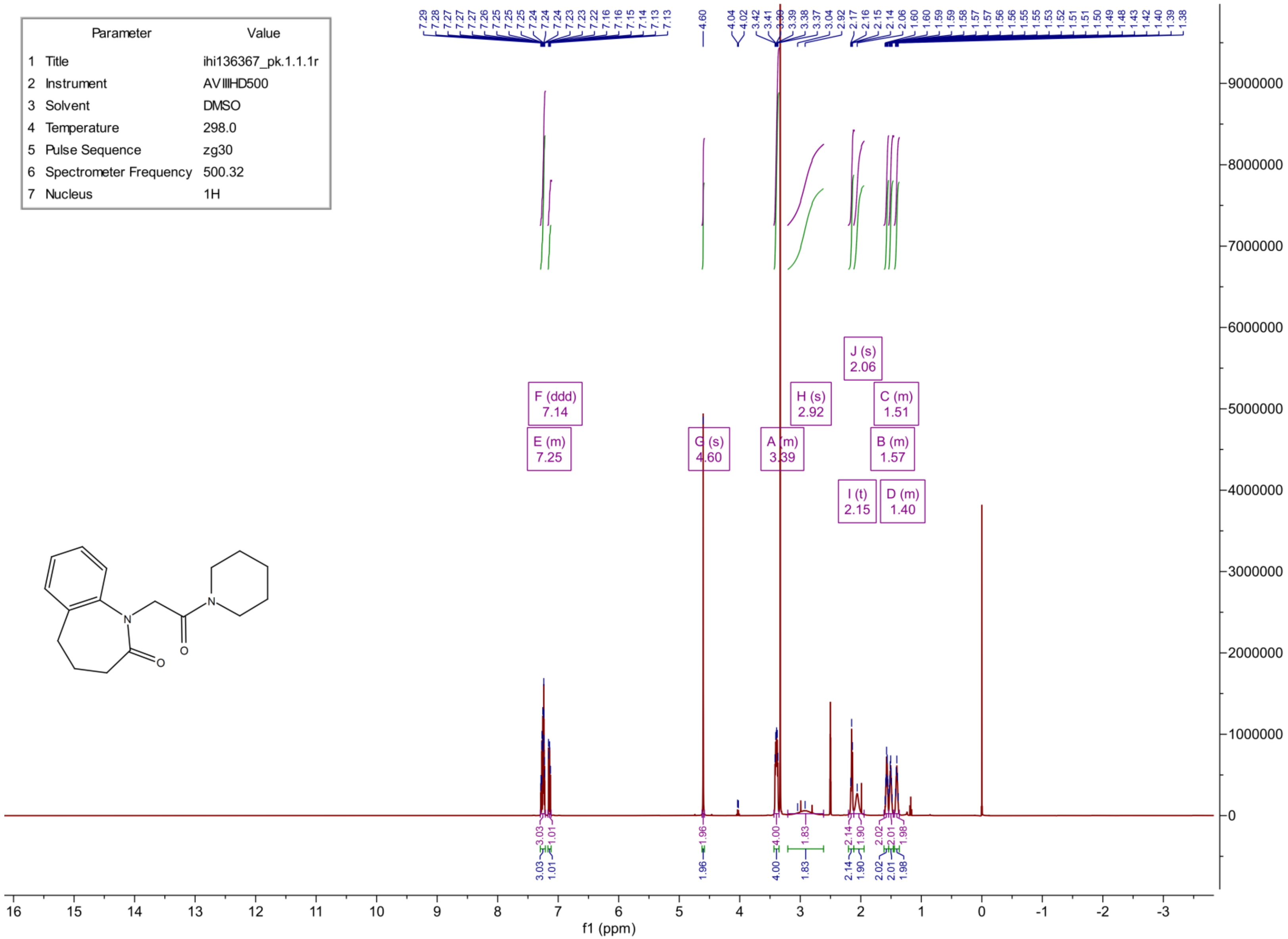

S1 File. Fig 11. ^{1}H-NMR spectrum of compound 2i.

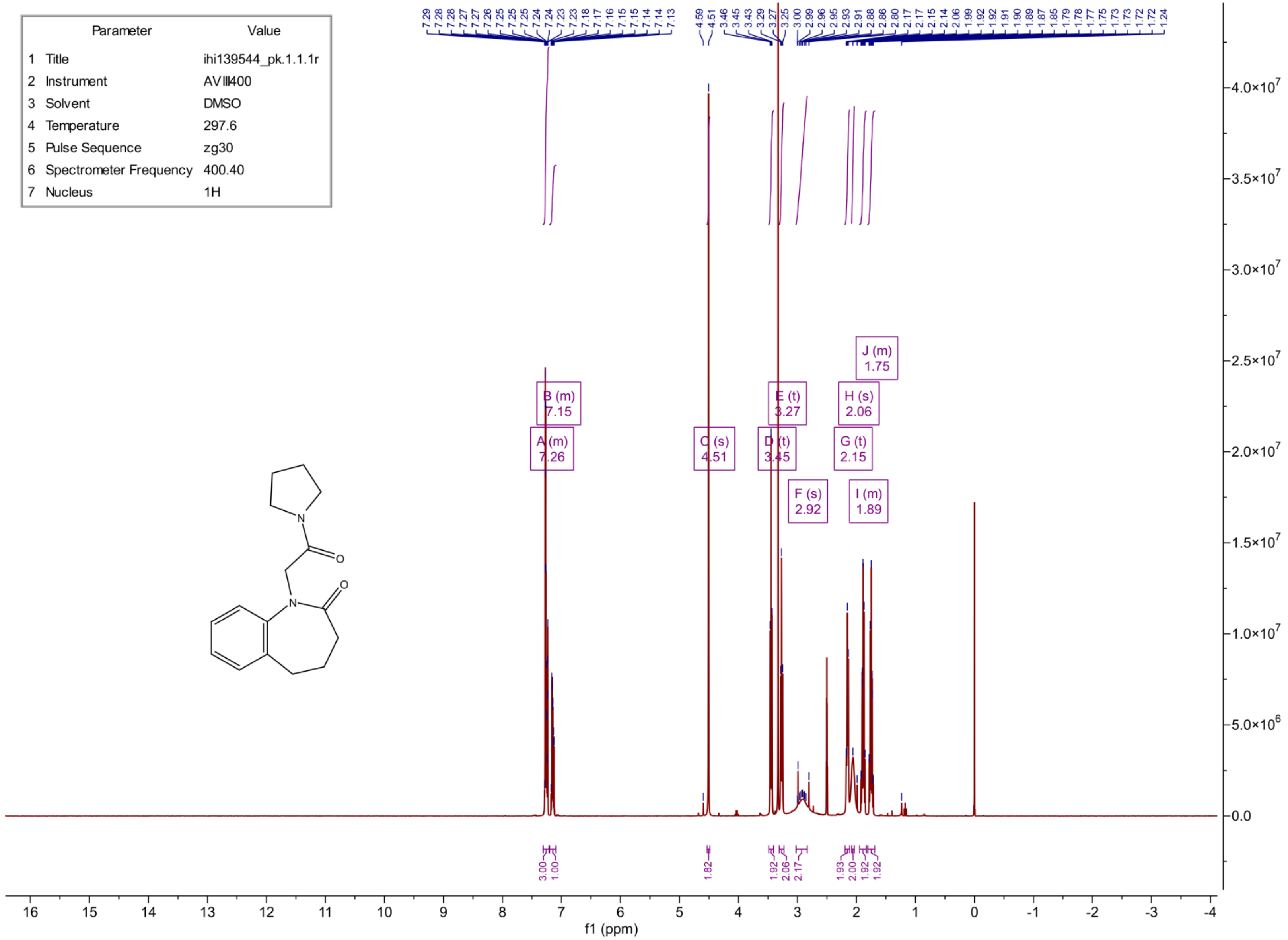

S1 File. Fig 12. ^{1}H-NMR spectrum of compound 2j.

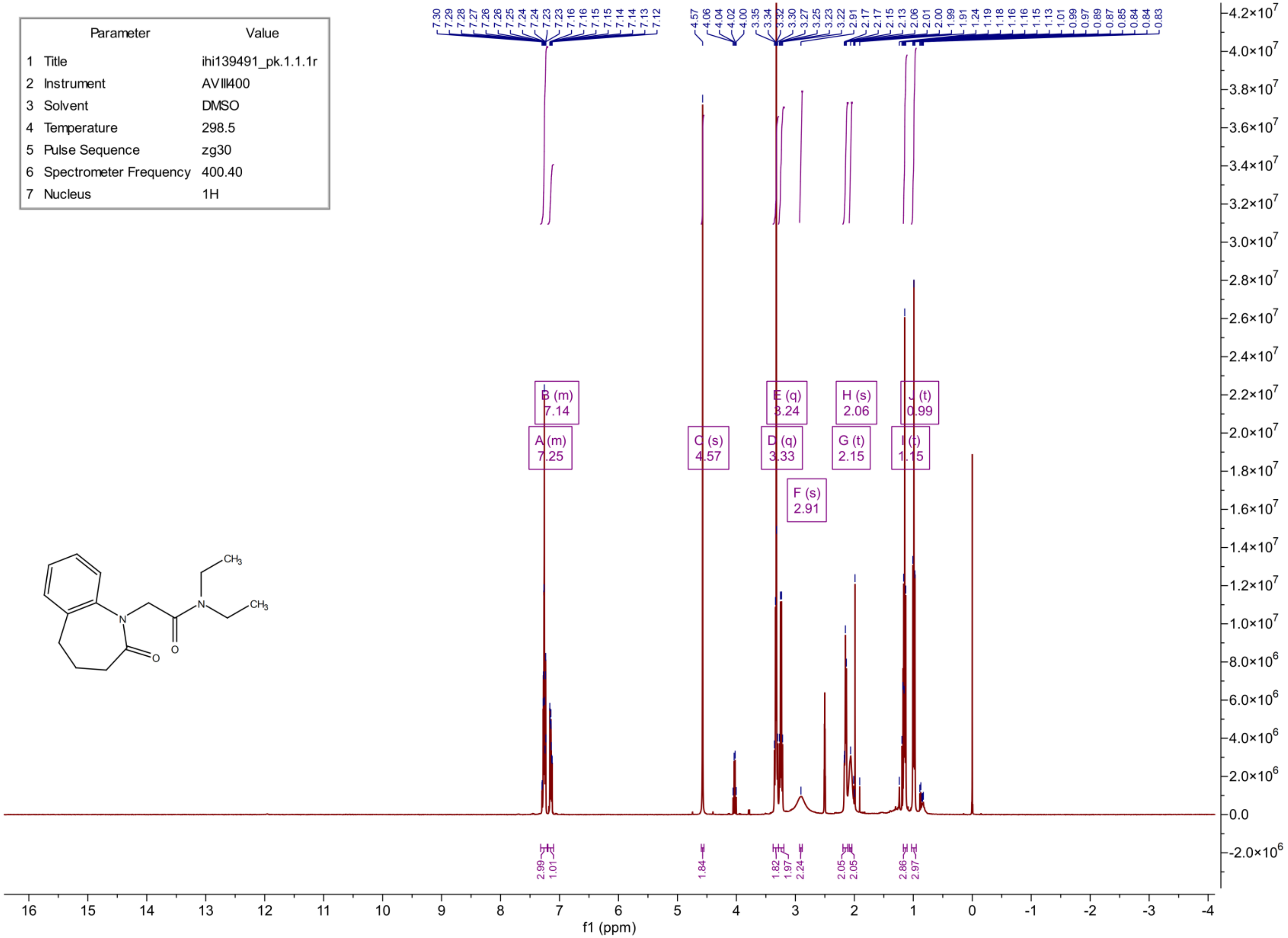

S1 File. Fig 13. ^{1}H-NMR spectrum of compound 2k.

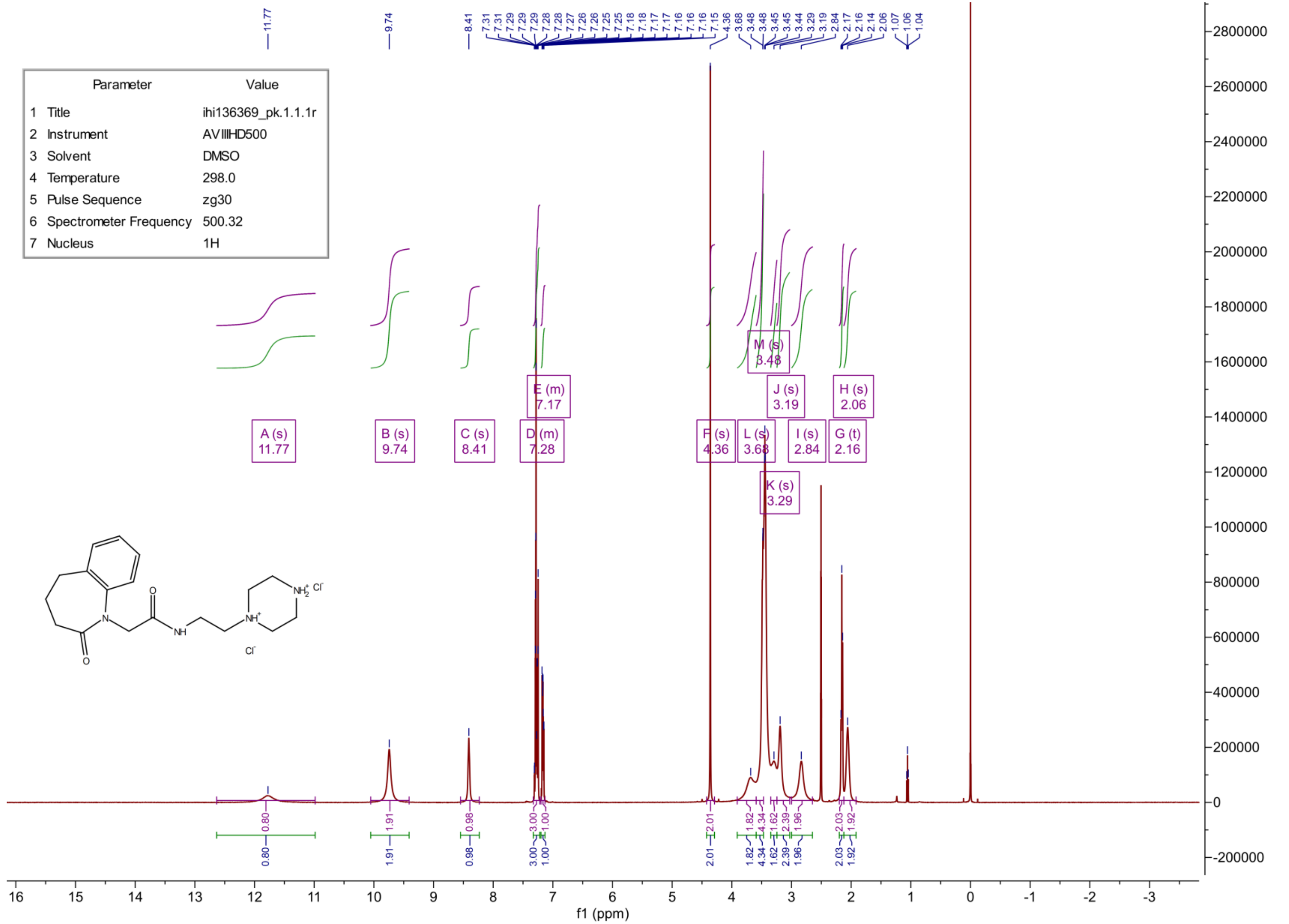

S1 File. Fig 14. ^{1}H-NMR spectrum of compound 2o.

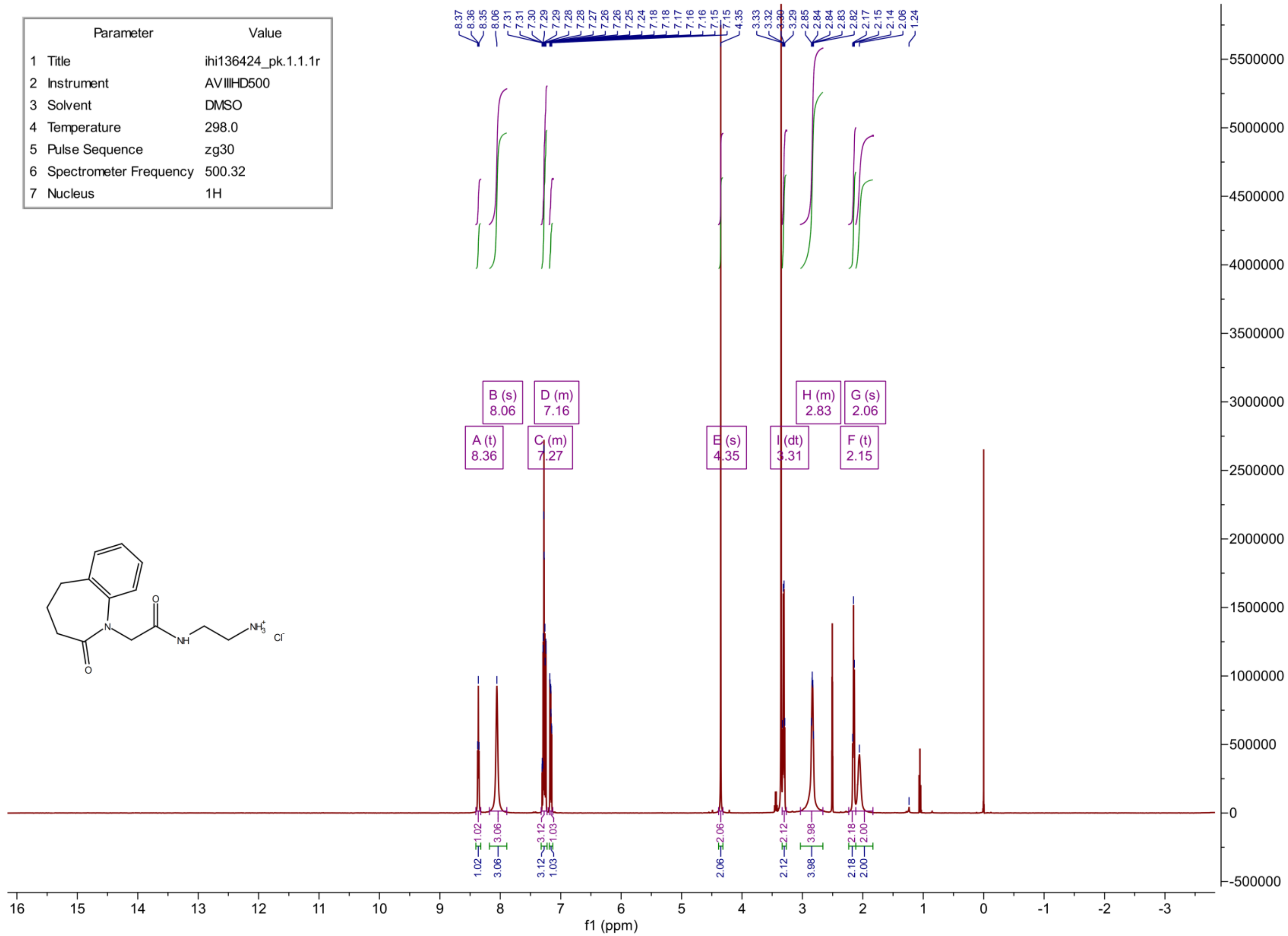

	Parameter	Value
1	Title	ihi136424_pk.1.1.1r
2	Instrument	AVIIIHD500
3	Solvent	DMSO
4	Temperature	298.0
5	Pulse Sequence	zg30
6	Spectrometer Frequency	500.32
7	Nucleus	1H

S1 File. Fig 15. ^{1}H-NMR spectrum of compound 2p.

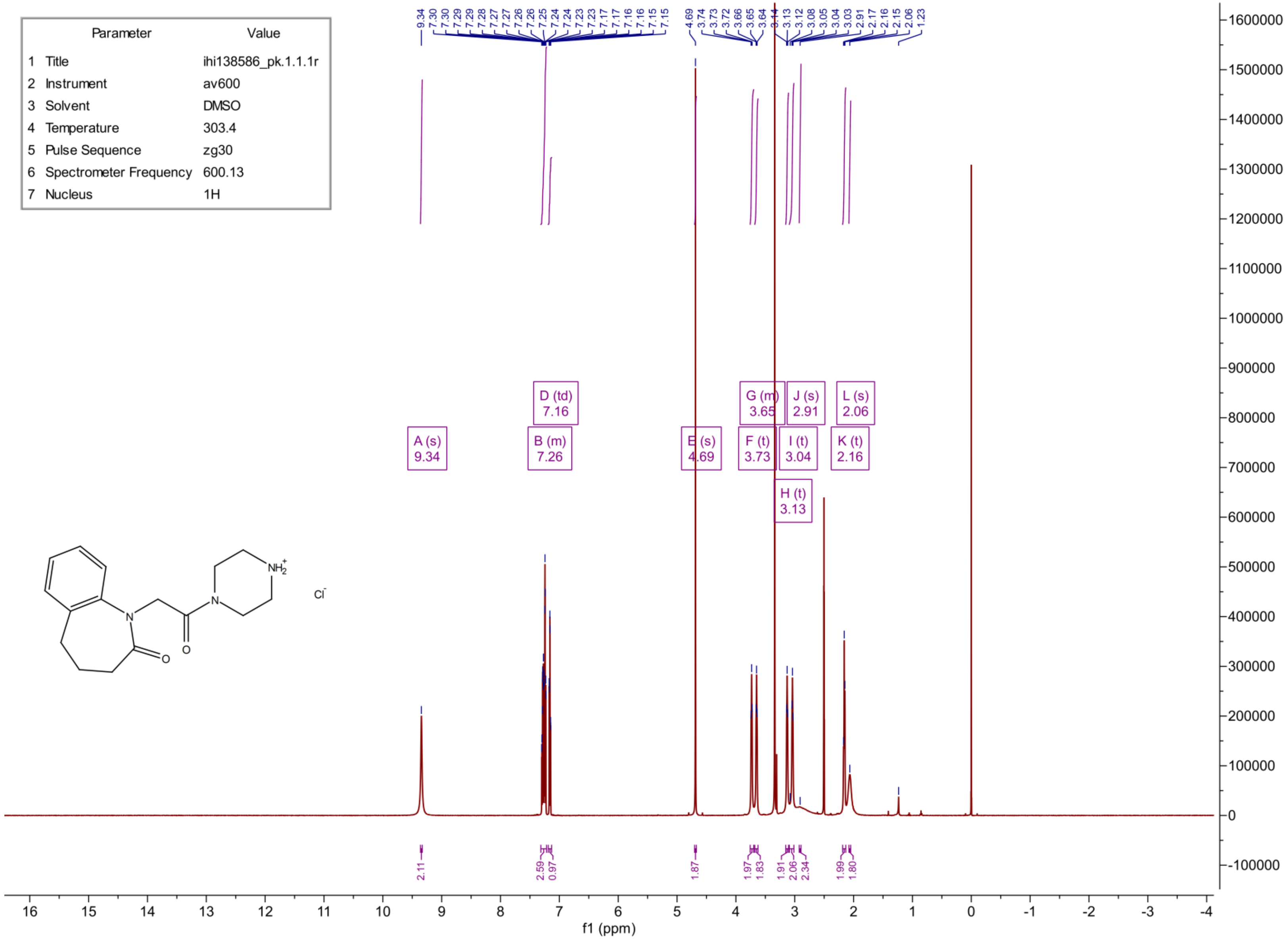

S1 File. Fig 16. ^{1}H-NMR spectrum of compound 2q.

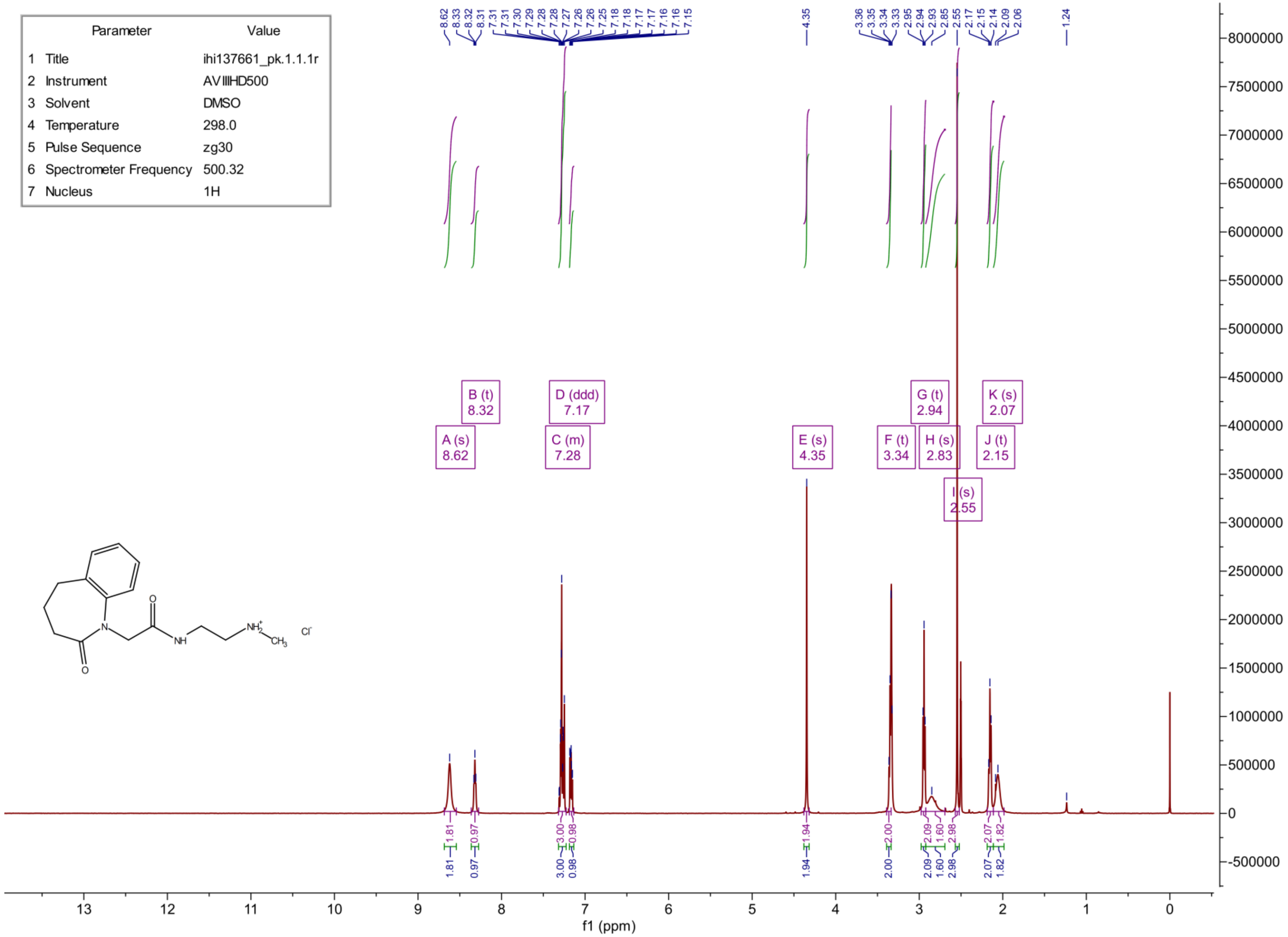

S1 File. Fig 17. ^{1}H-NMR spectrum of compound 2r.

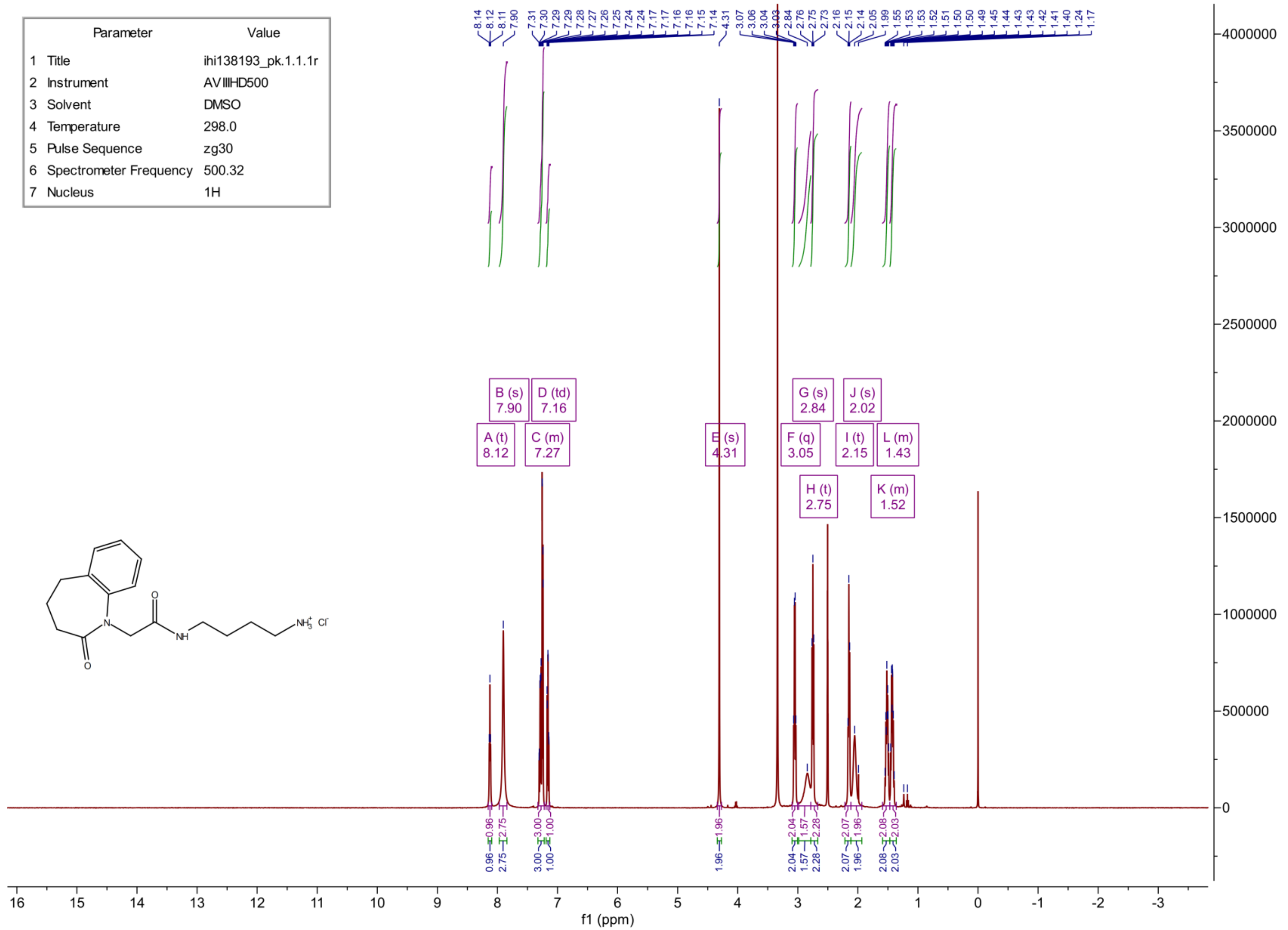

S1 File. Fig 18. ^{1}H-NMR spectrum of compound 2s.

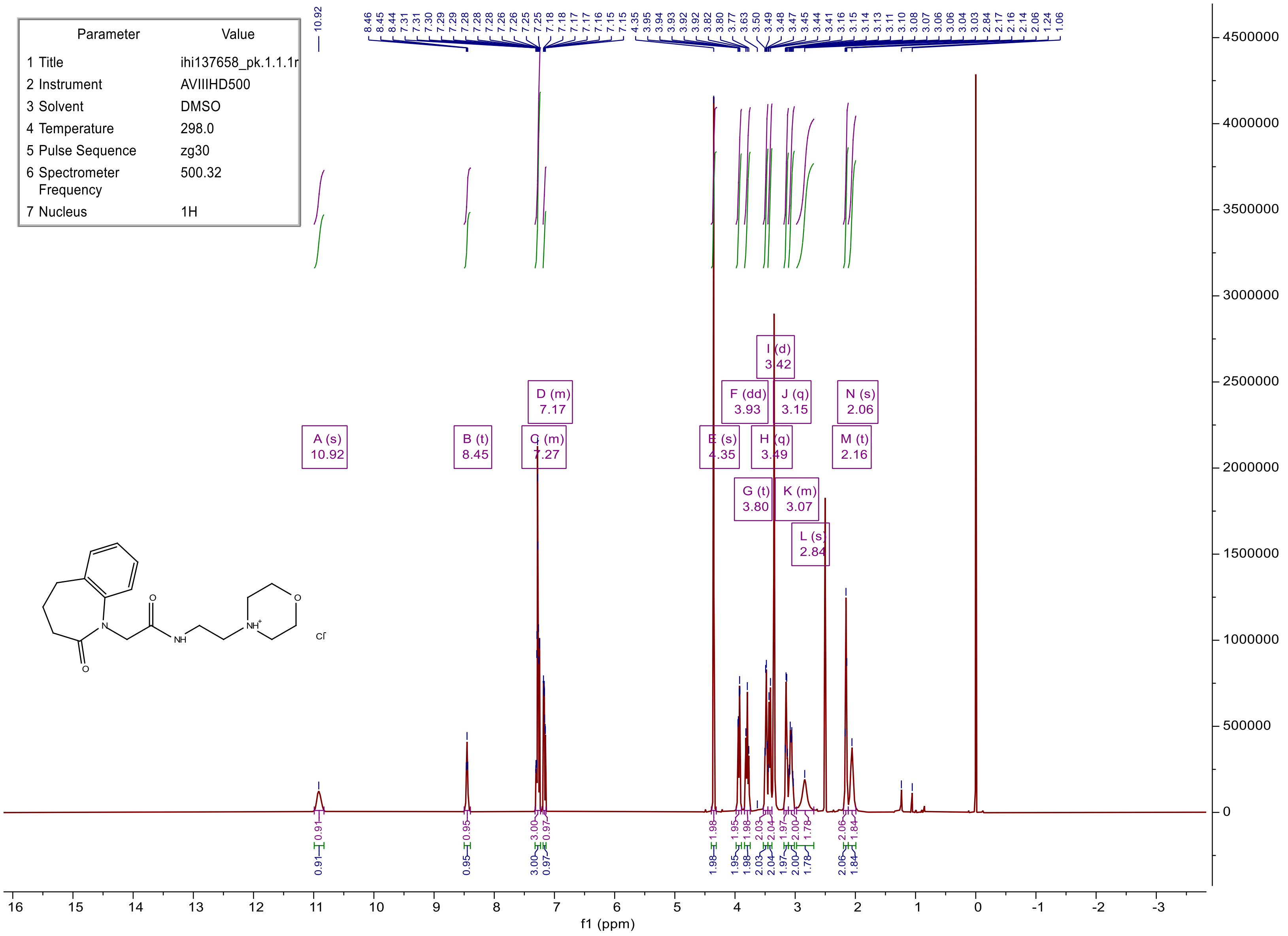

S1 File. Fig 19. ^{1}H-NMR spectrum of compound 2t.

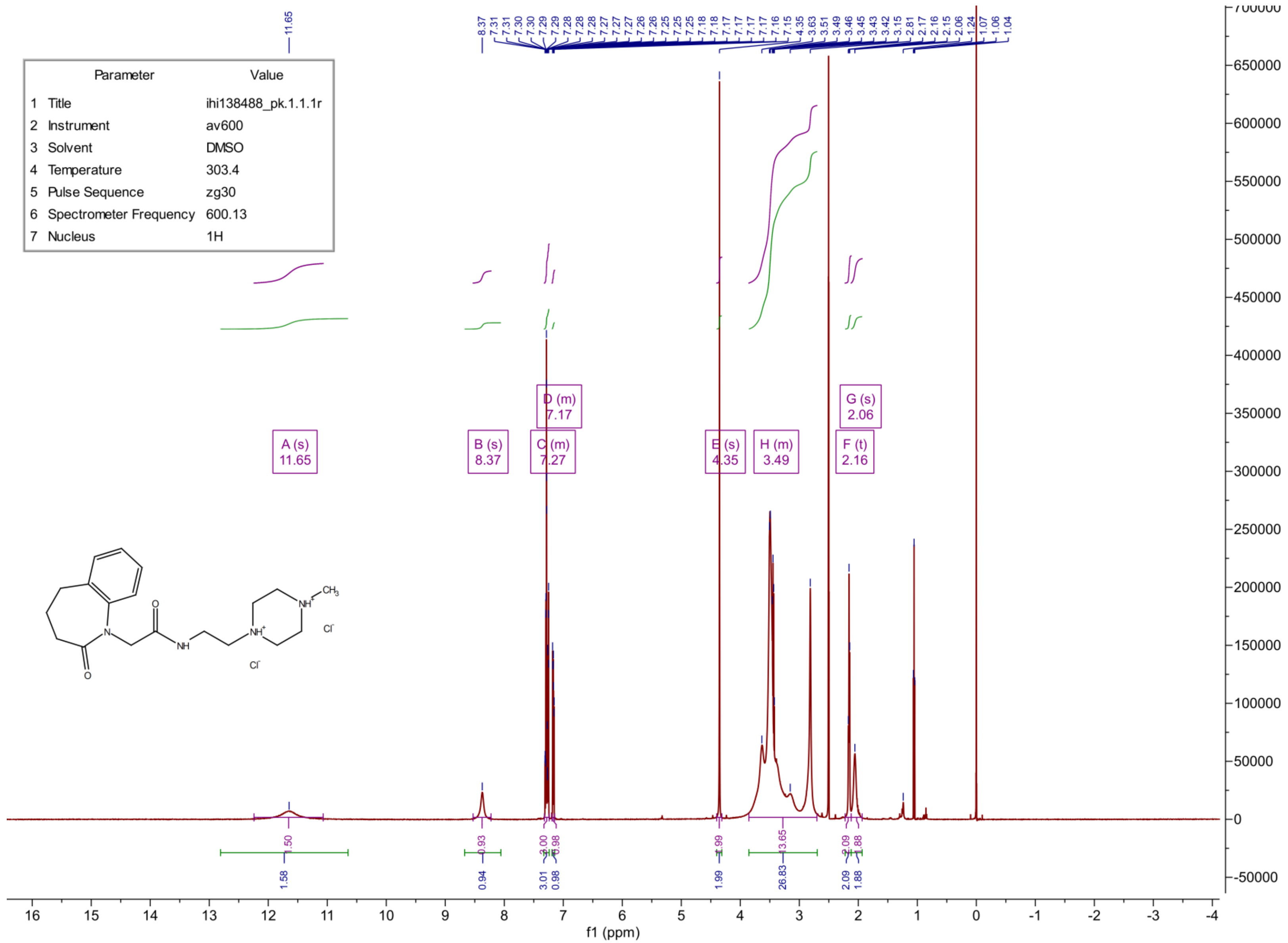

S1 File. Fig 20. ^{1}H-NMR spectrum of compound 2u.

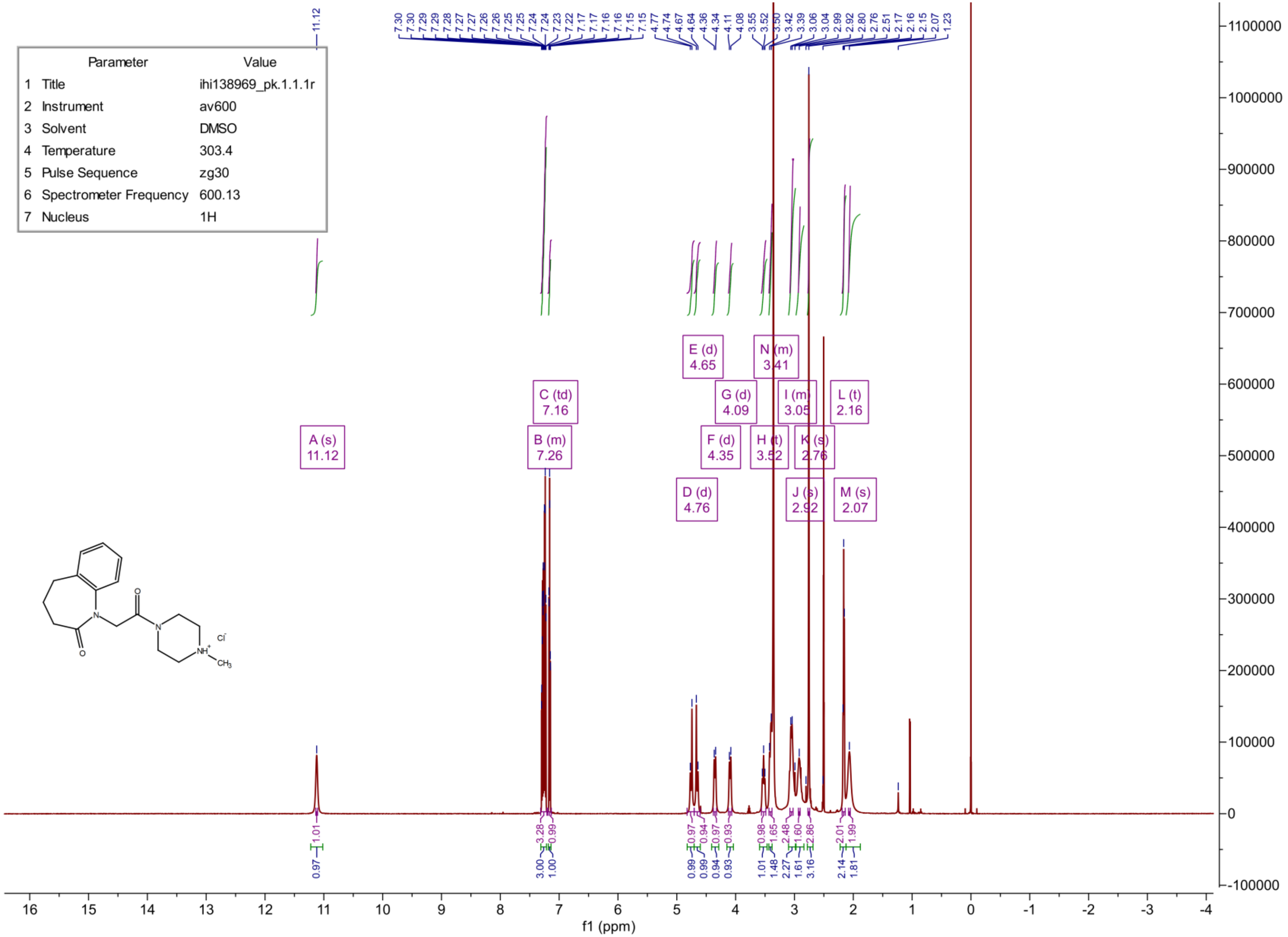

S1 File. Fig 21. ^{1}H-NMR spectrum of compound 2v.

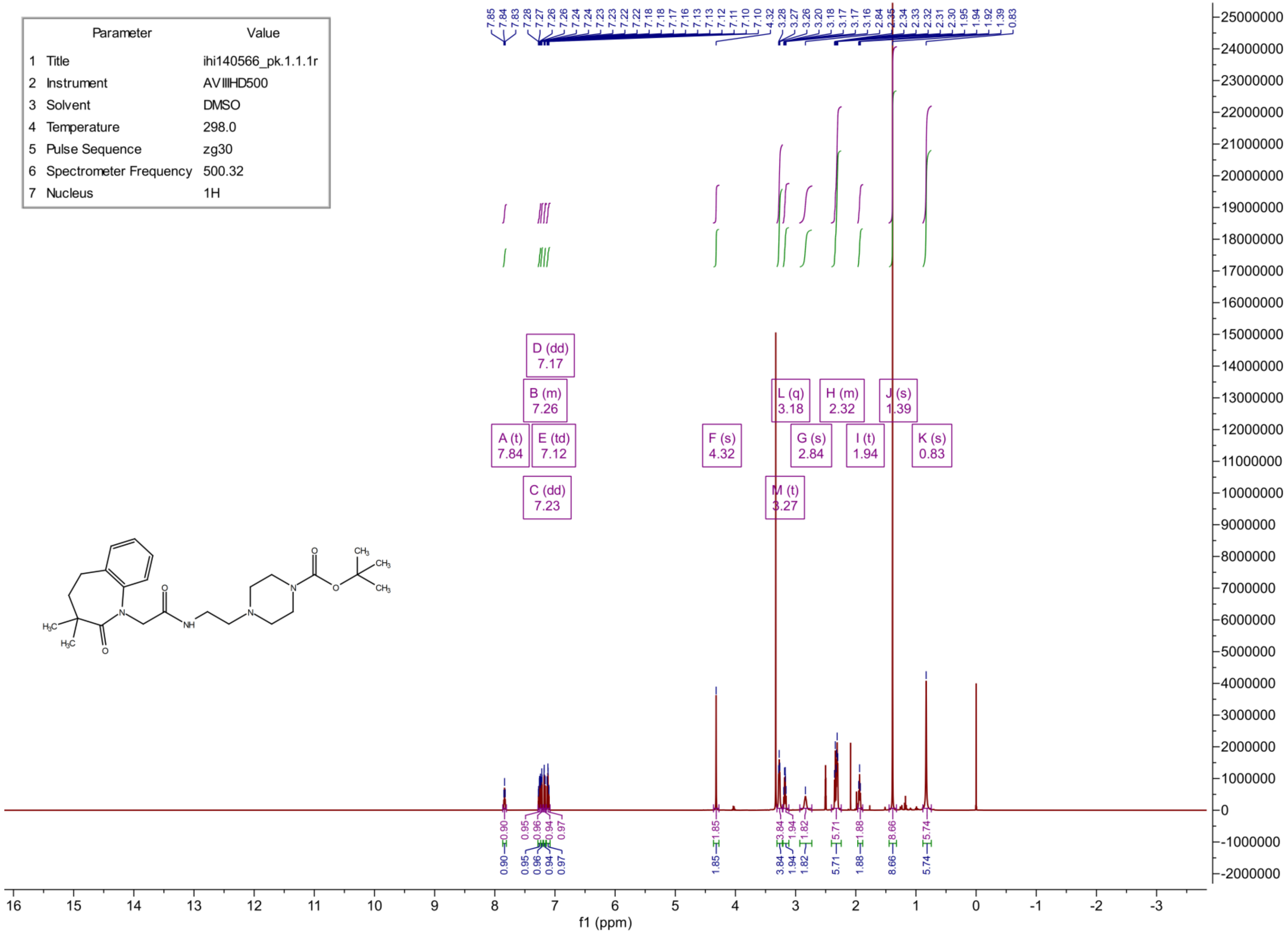

S1 File. Fig 22 ^{1}H-NMR spectrum of compound 3b.

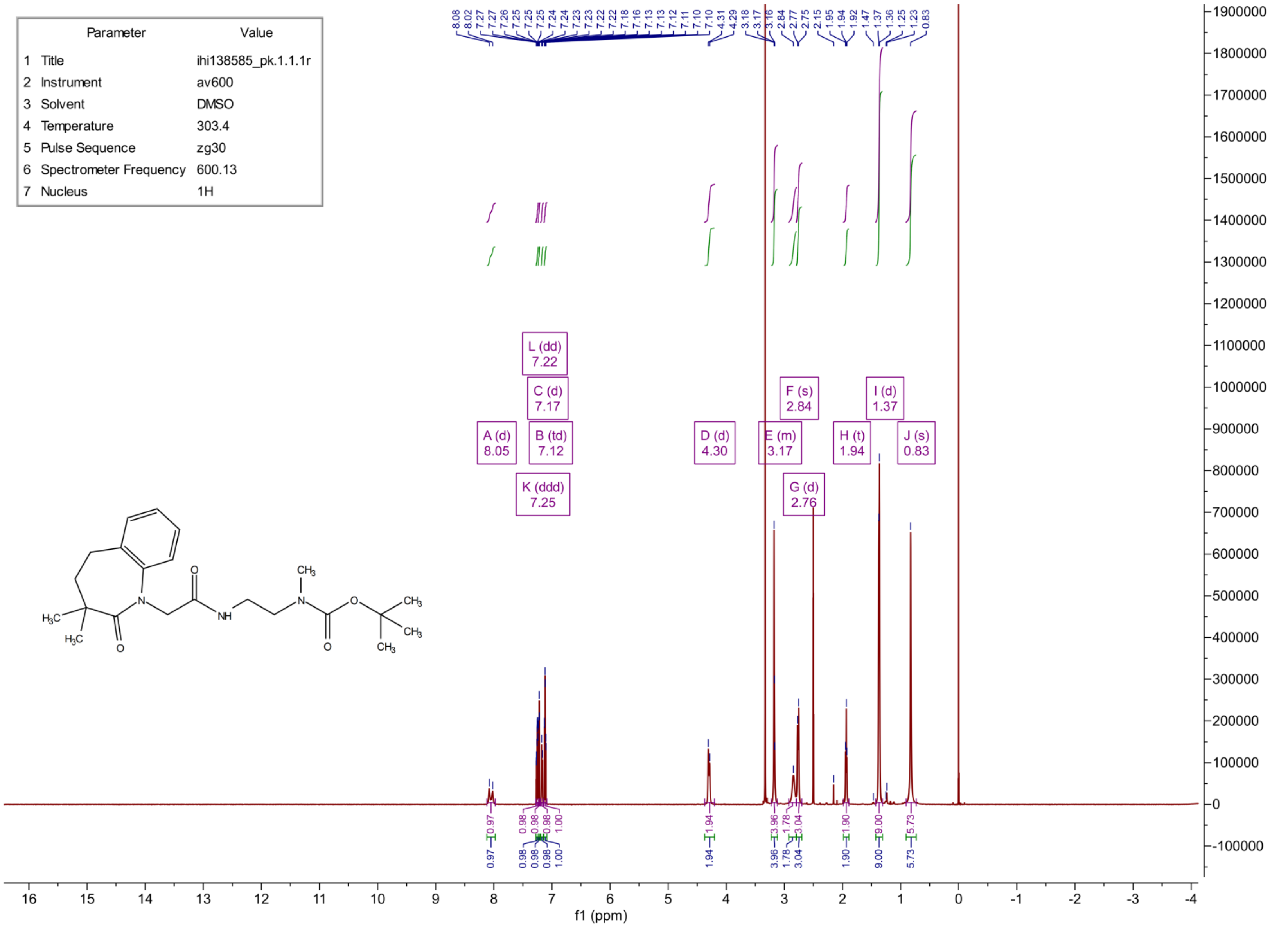

S1 File. Fig 23. ^{1}H-NMR spectrum of compound 3c.

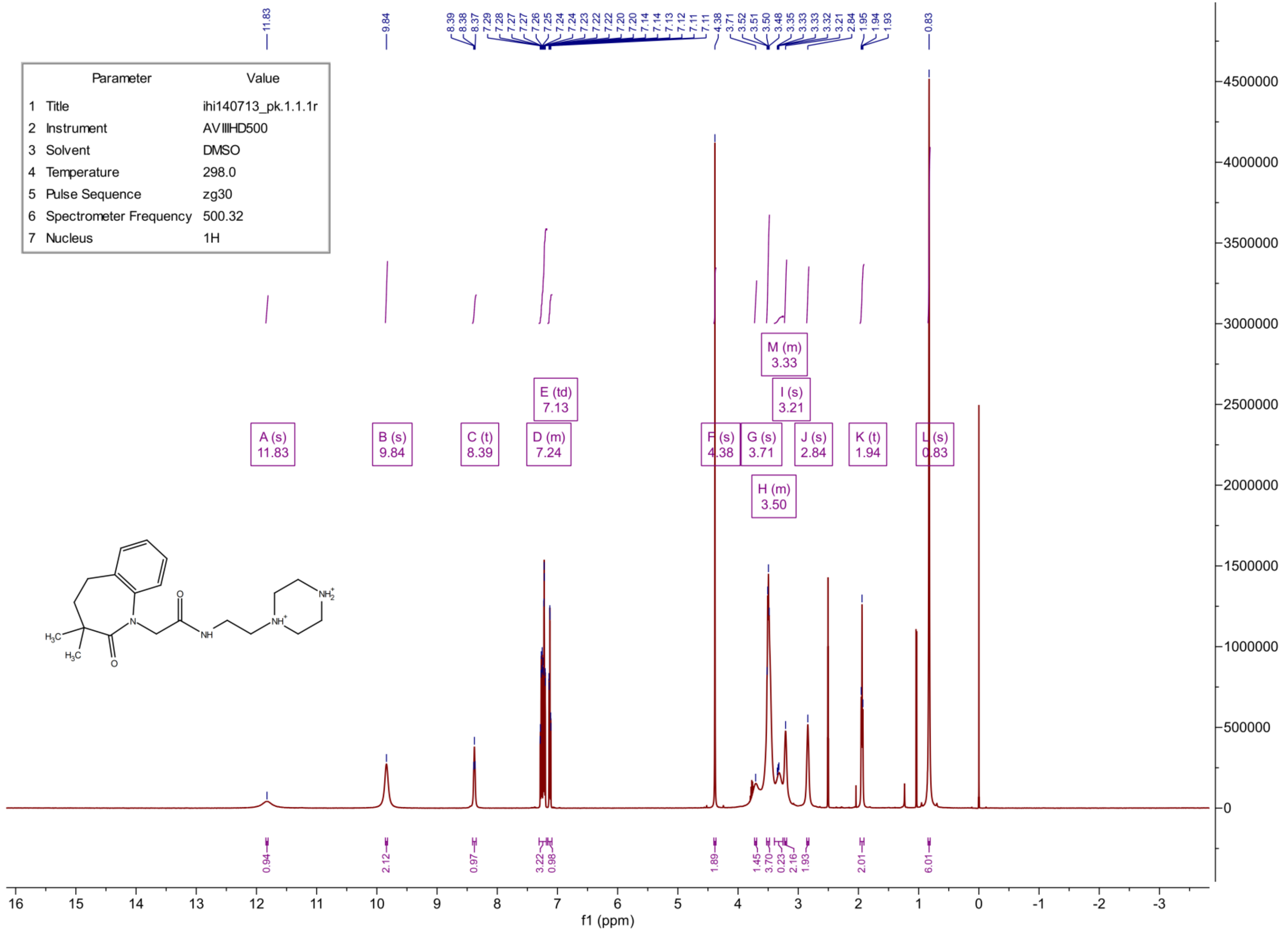

S1 File. Fig 24. ^{1}H-NMR spectrum of compound 3d.

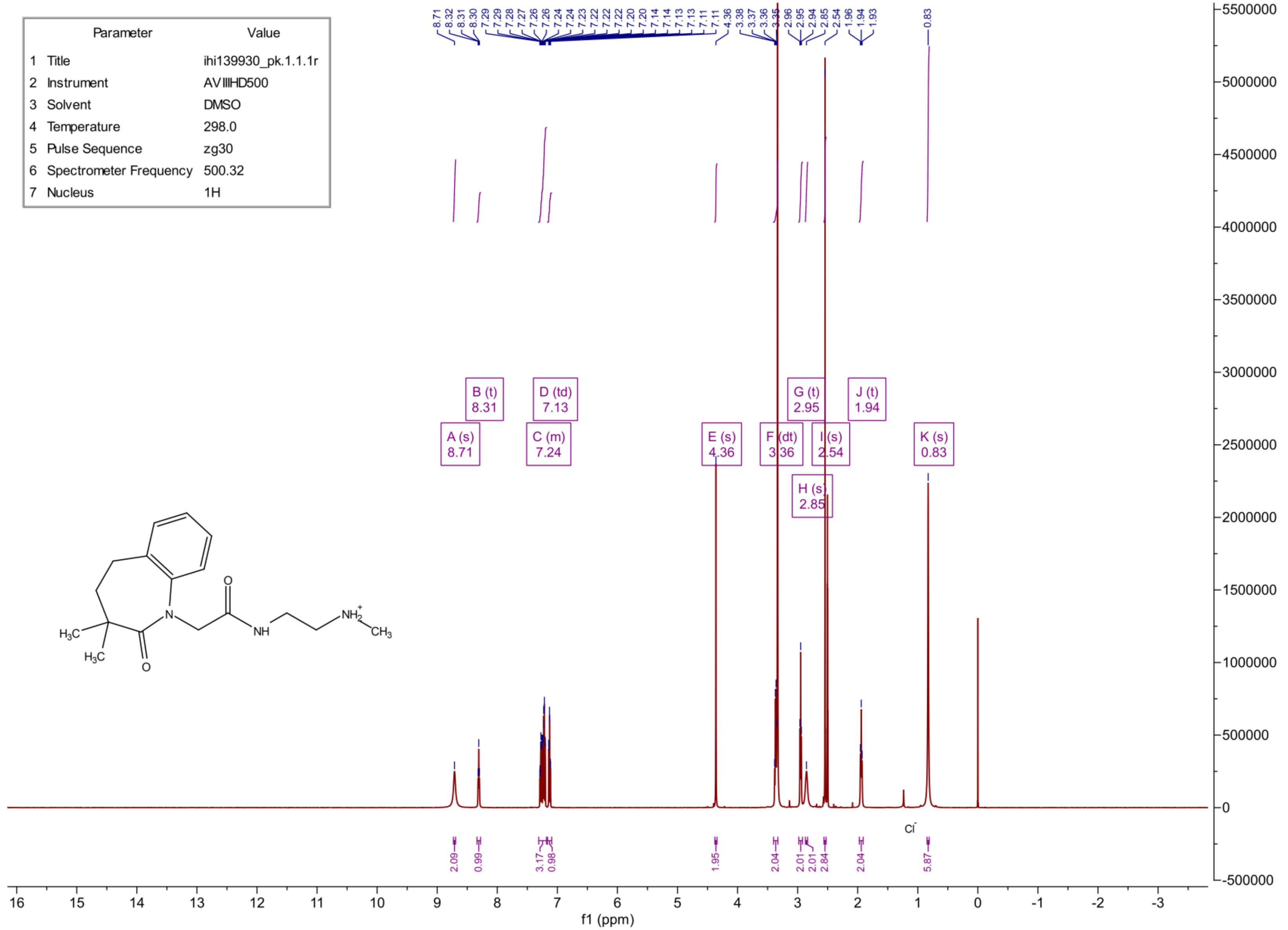

S1 File. Fig 25. ^{1}H-NMR spectrum of compound 3e.

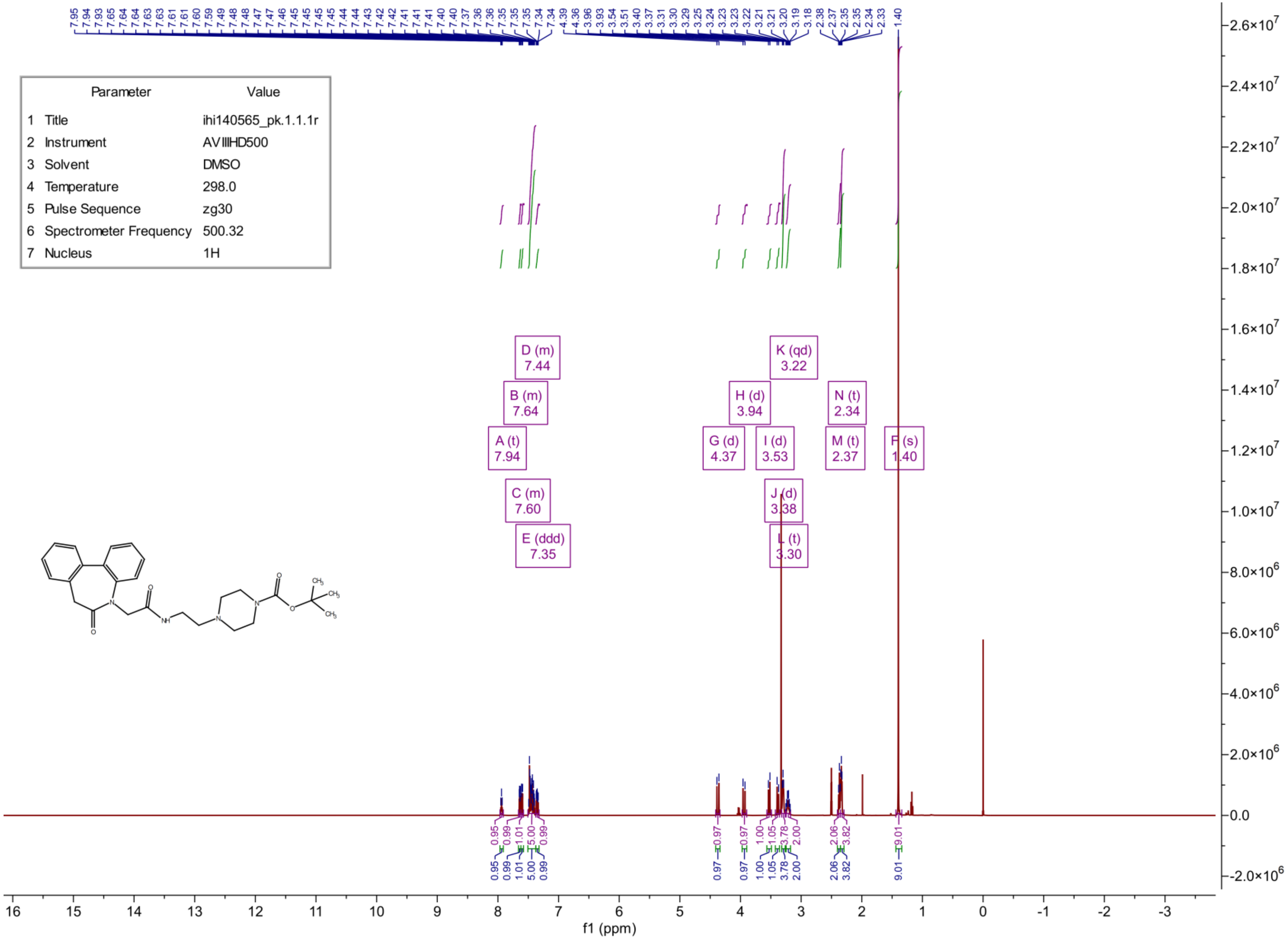

S1 File. Fig 26. ^{1}H-NMR spectrum of compound 4b.

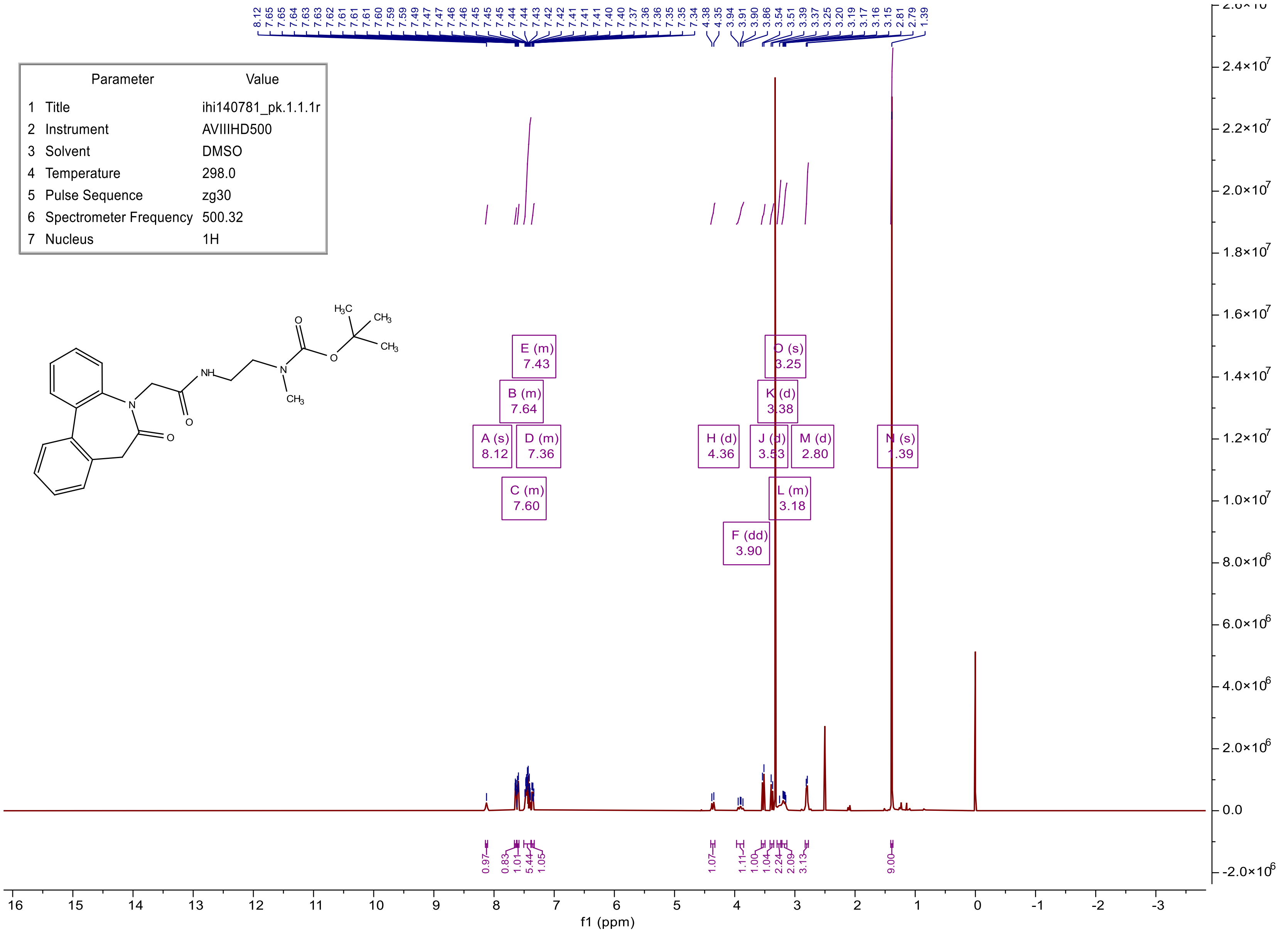

S1 File. Fig 27. ^{1}H-NMR spectrum of compound 4c.

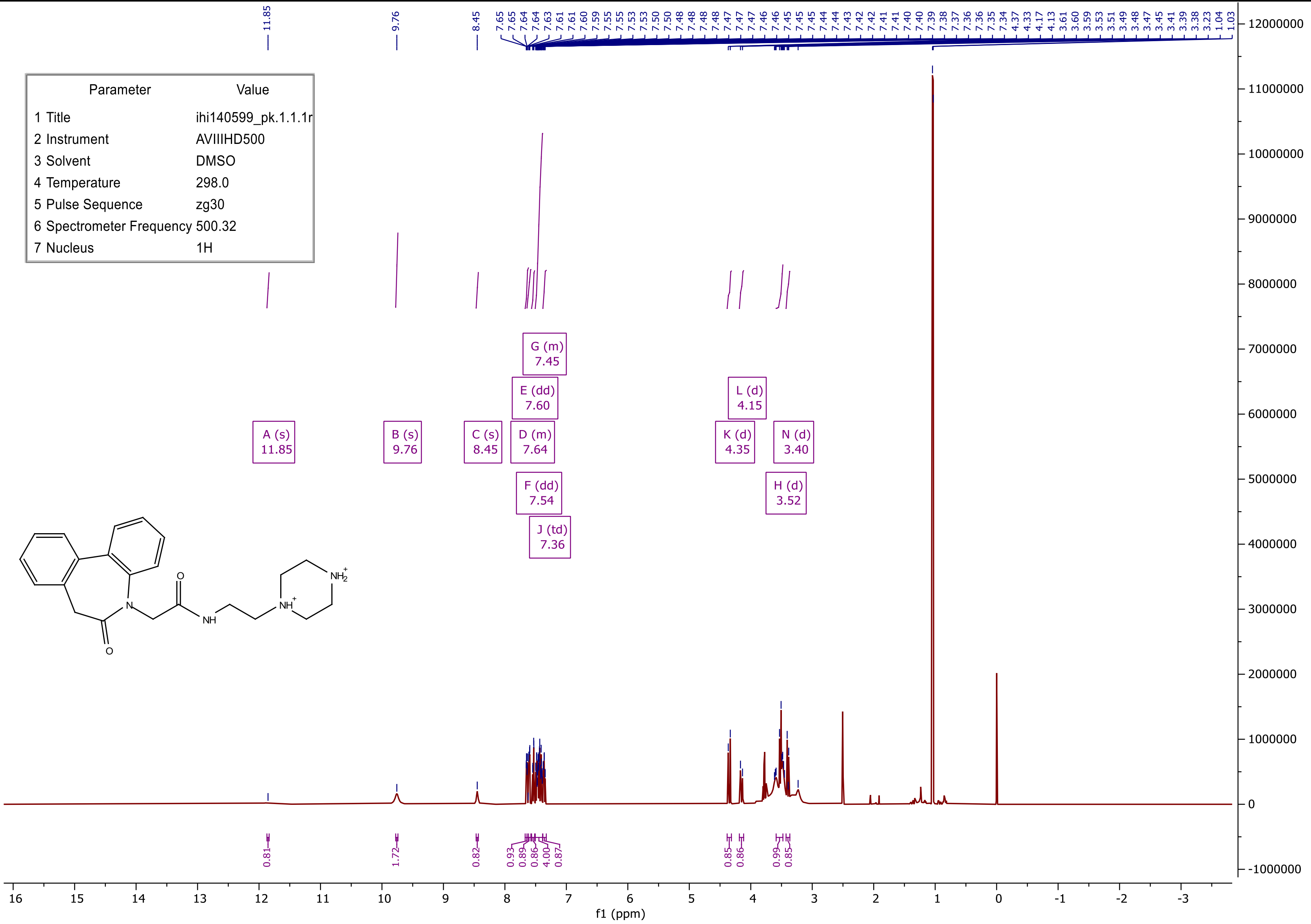

S1 File. Fig 28. ^{1}H-NMR spectrum of compound 4d.

	Parameter	Value
1	Title	ihi140665_pk.1.1.1r
2	Instrument	AVIIIHD500
3	Solvent	DMSO
4	Temperature	298.0
5	Pulse Sequence	zg30
6	Spectrometer Frequency	500.32
7	Nucleus	1H

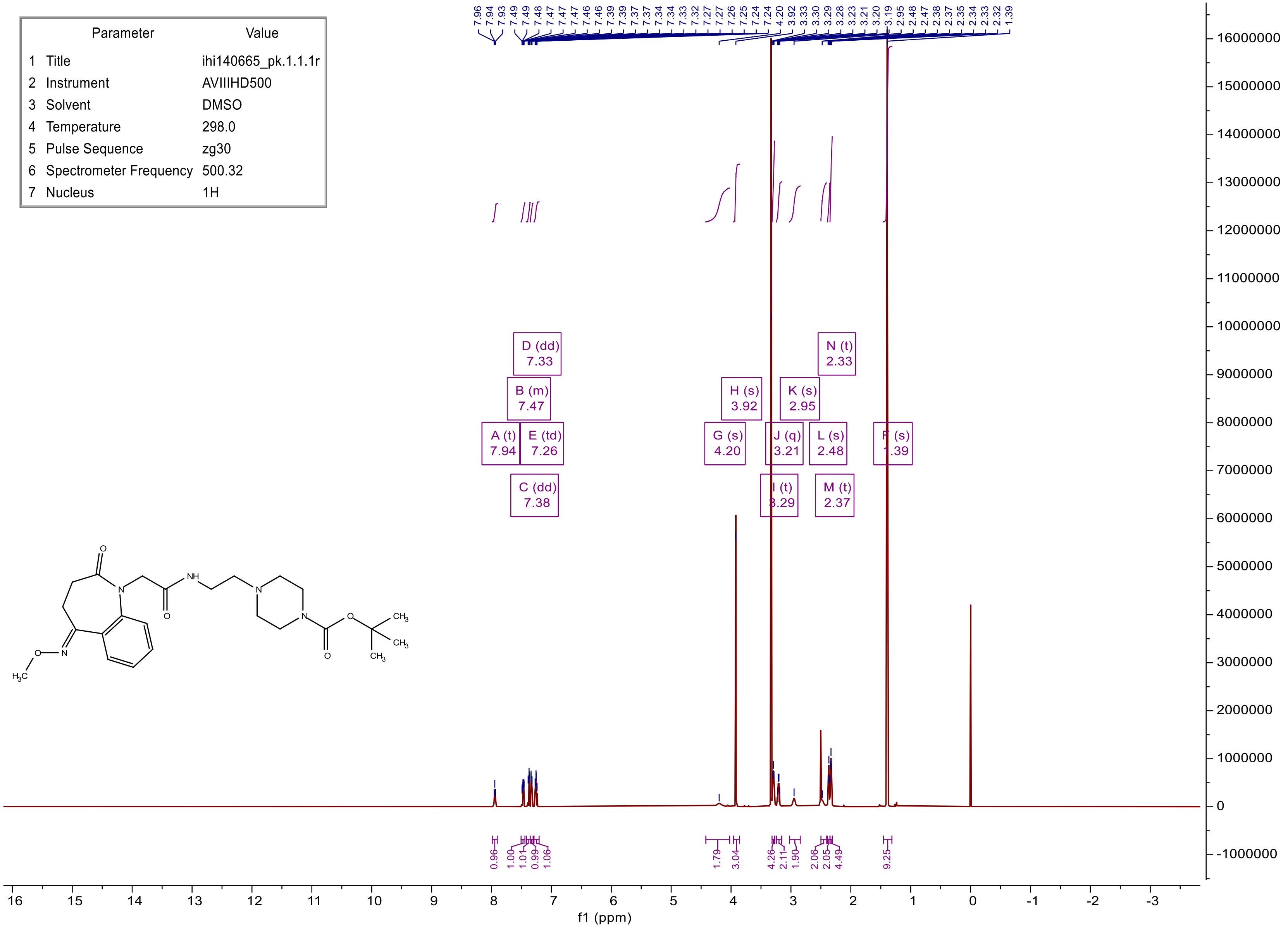

S1 File. Fig 29. ^{1}H-NMR spectrum of compound 5b.

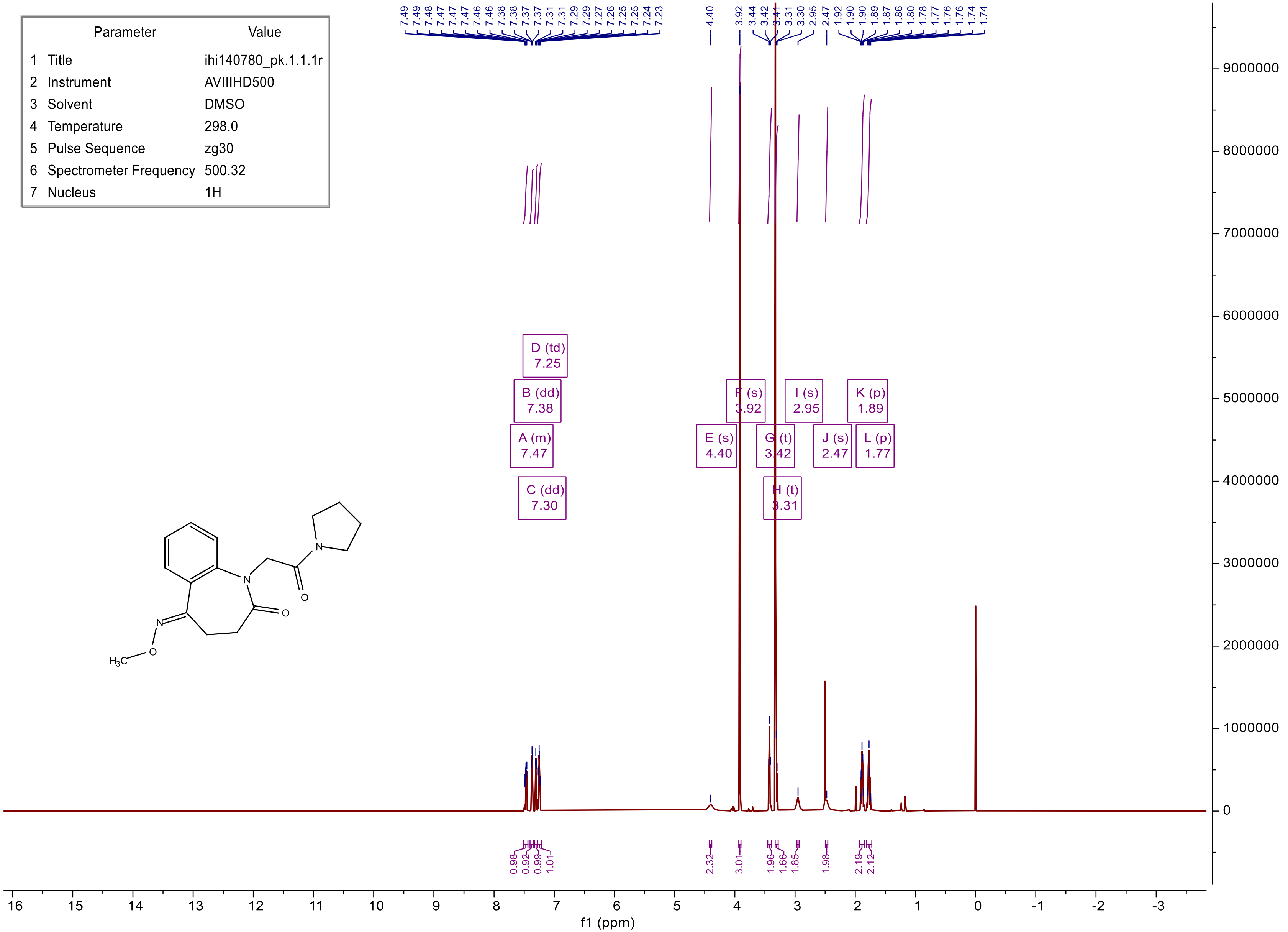

S1 File. Fig 30. ^{1}H-NMR spectrum of compound 5c.

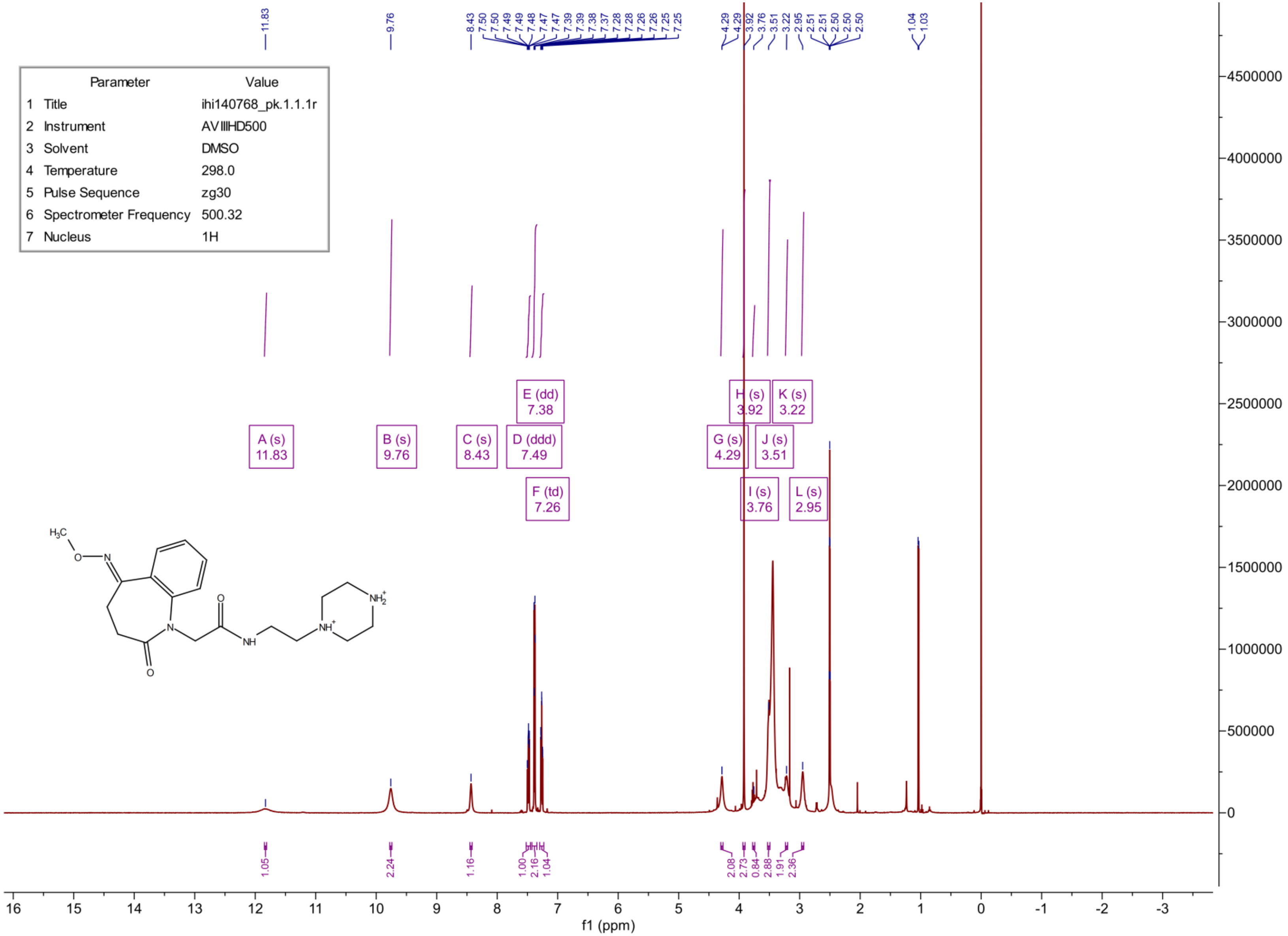

S1 File. Fig 31. ^{1}H-NMR spectrum of compound 5d.

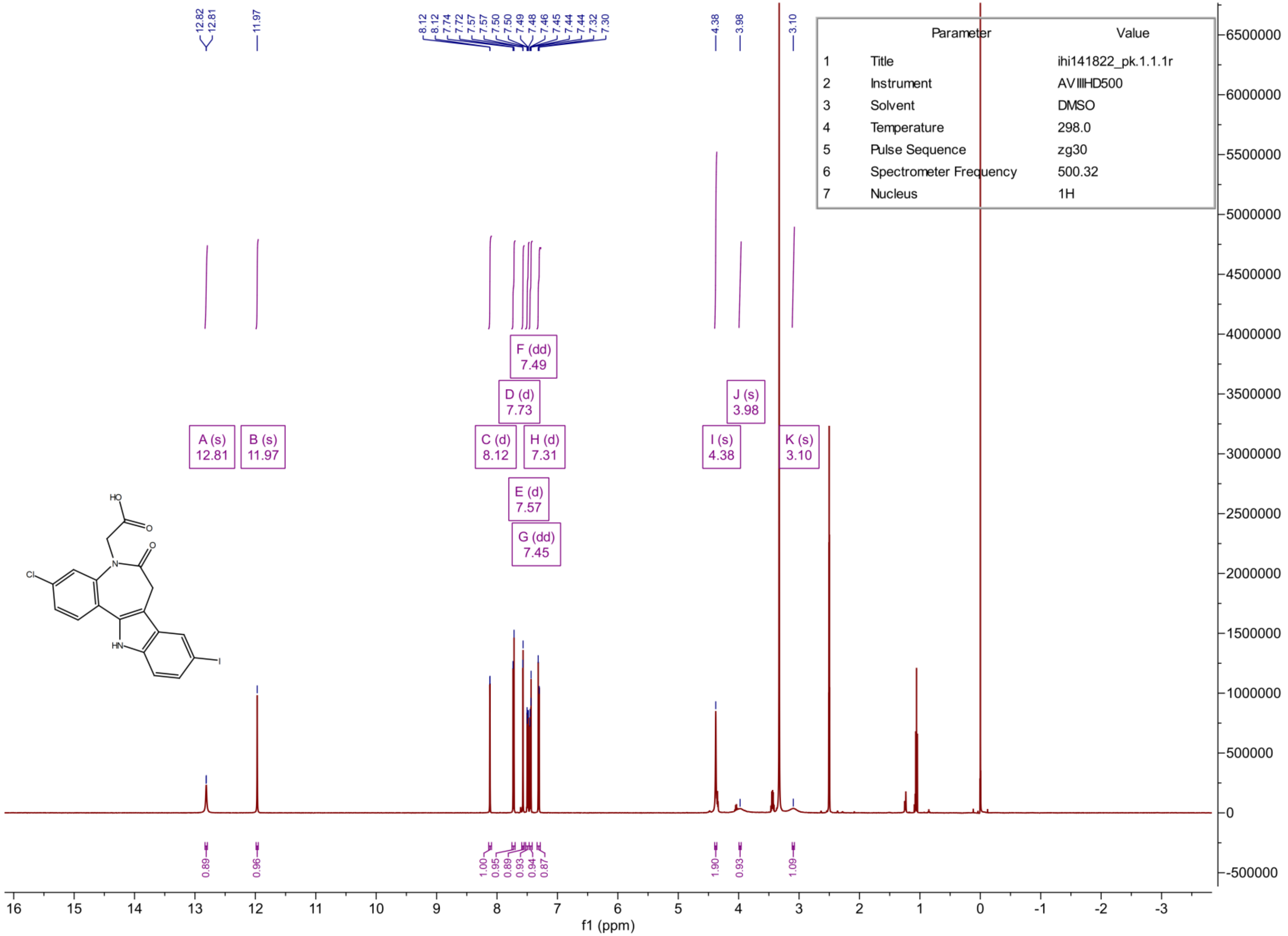

S1 File. Fig 32. ^{1}H-NMR spectrum of compound 9a.

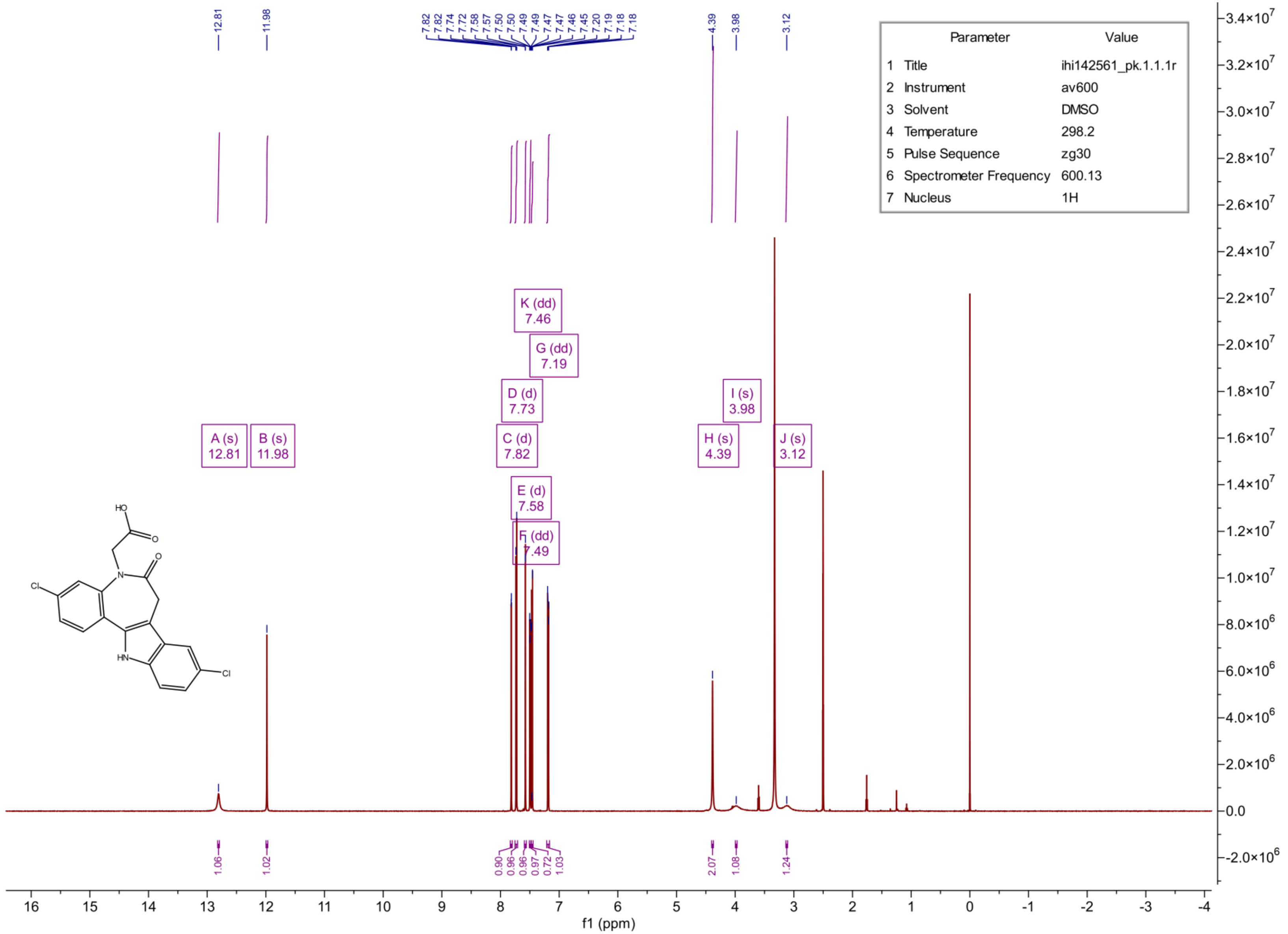

S1 File. Fig 33. ^{1}H-NMR spectrum of compound 9b.

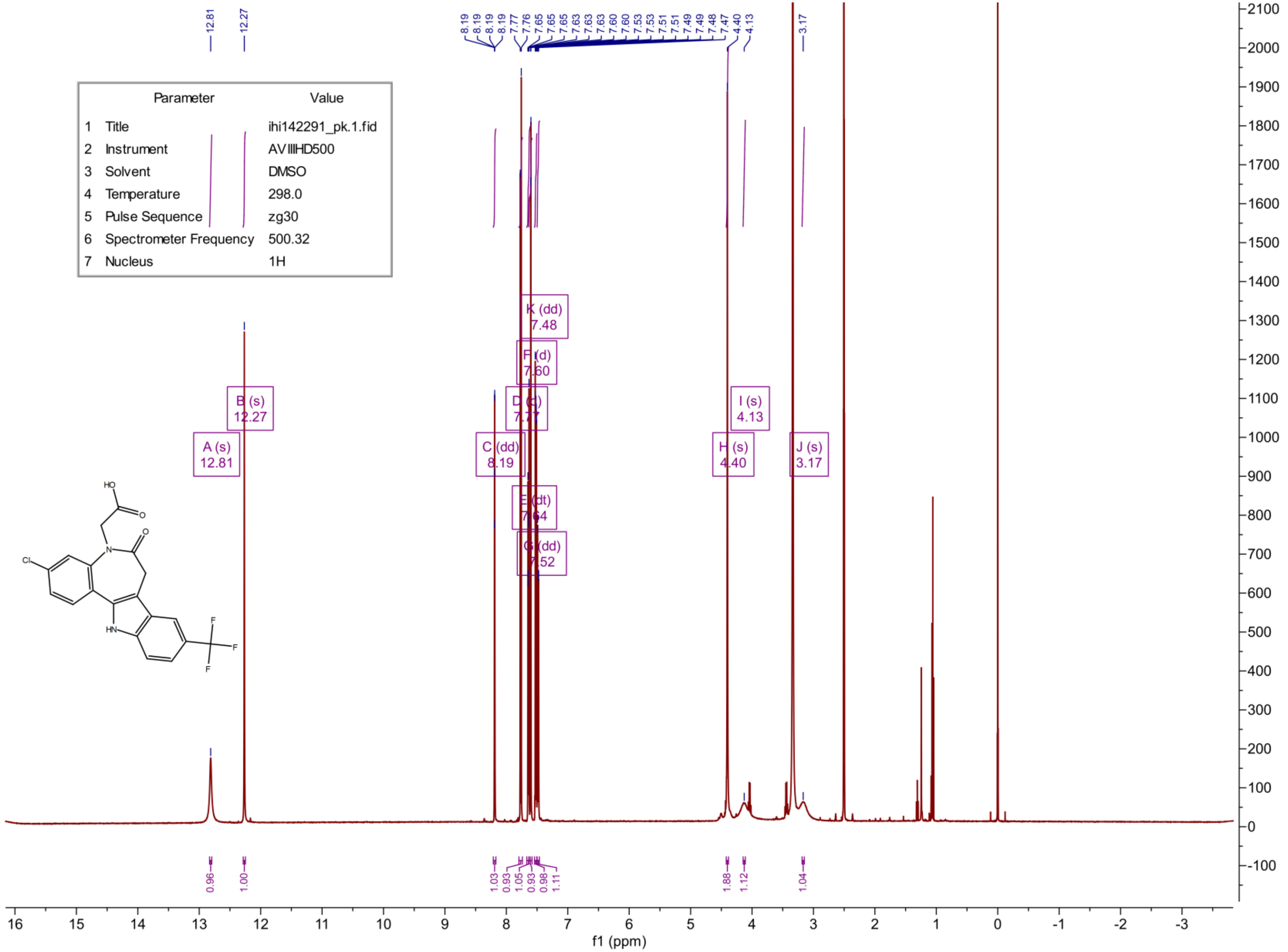

S1 File. Fig 34. ^{1}H-NMR spectrum of compound 9c.

Parameter		Value
1	Title	ihi141896_pk.1.1.1r
2	Instrument	AVIIIHD500
3	Solvent	DMSO
4	Temperature	298.0
5	Pulse Sequence	zg30
6	Spectrometer Frequency	500.32
7	Nucleus	1H

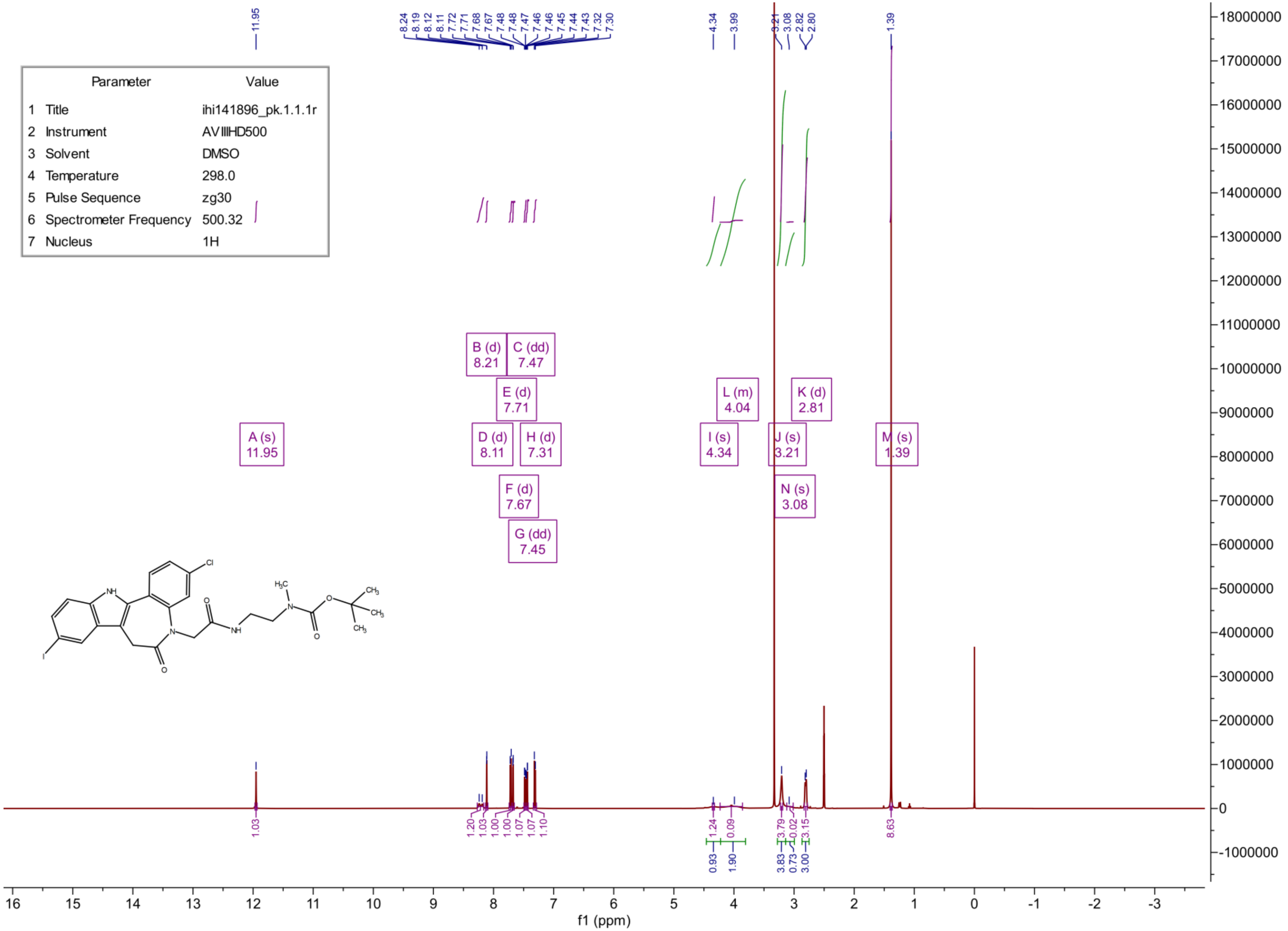

S1 File. Fig 35. ^{1}H-NMR spectrum of compound 10a.

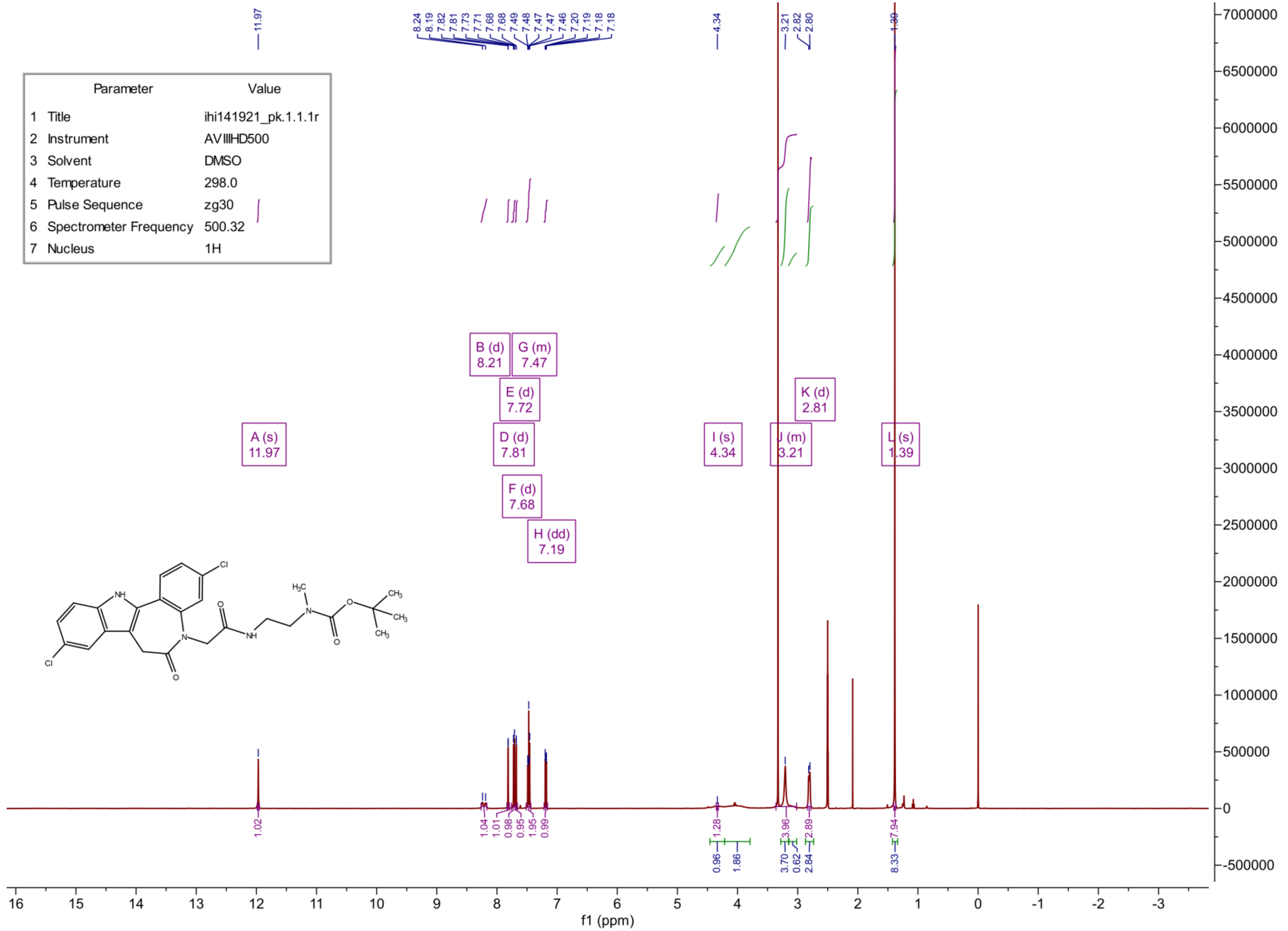

S1 File. Fig 36. ^{1}H-NMR spectrum of compound 10b.

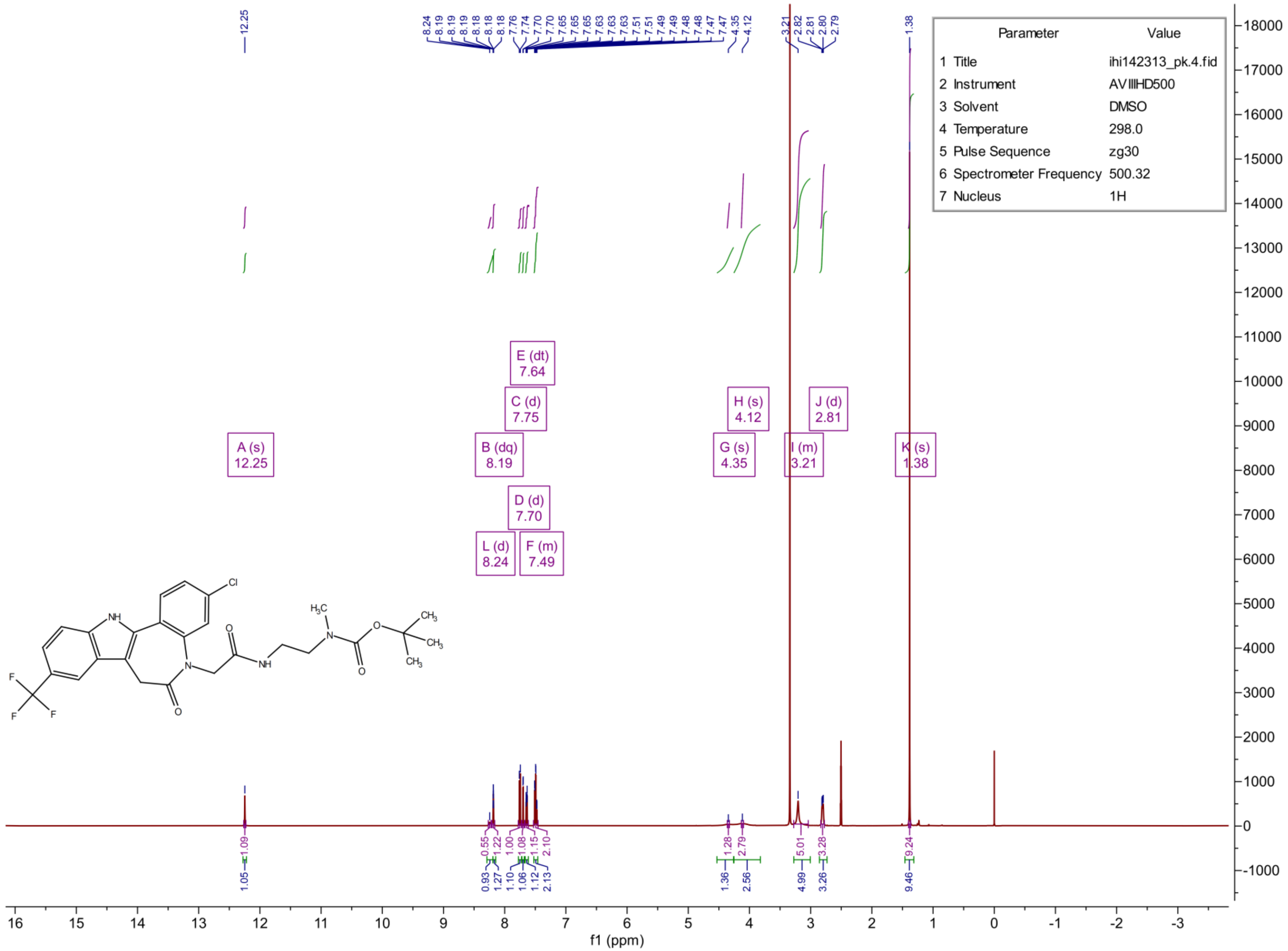

S1 File. Fig 37. ^{1}H-NMR spectrum of compound 10c.

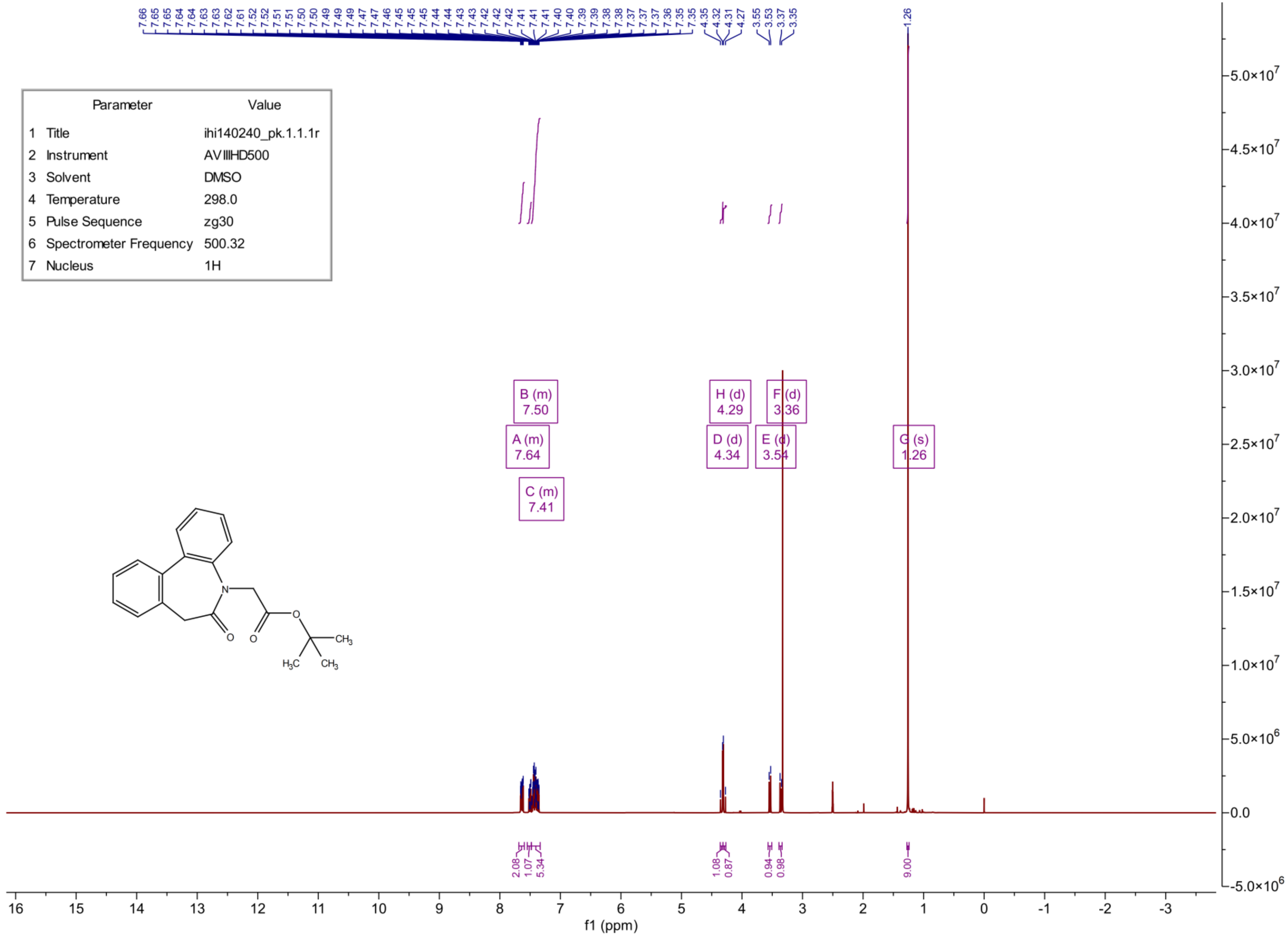

S1 File. Fig 38. ¹H-NMR spectrum of compound 18.

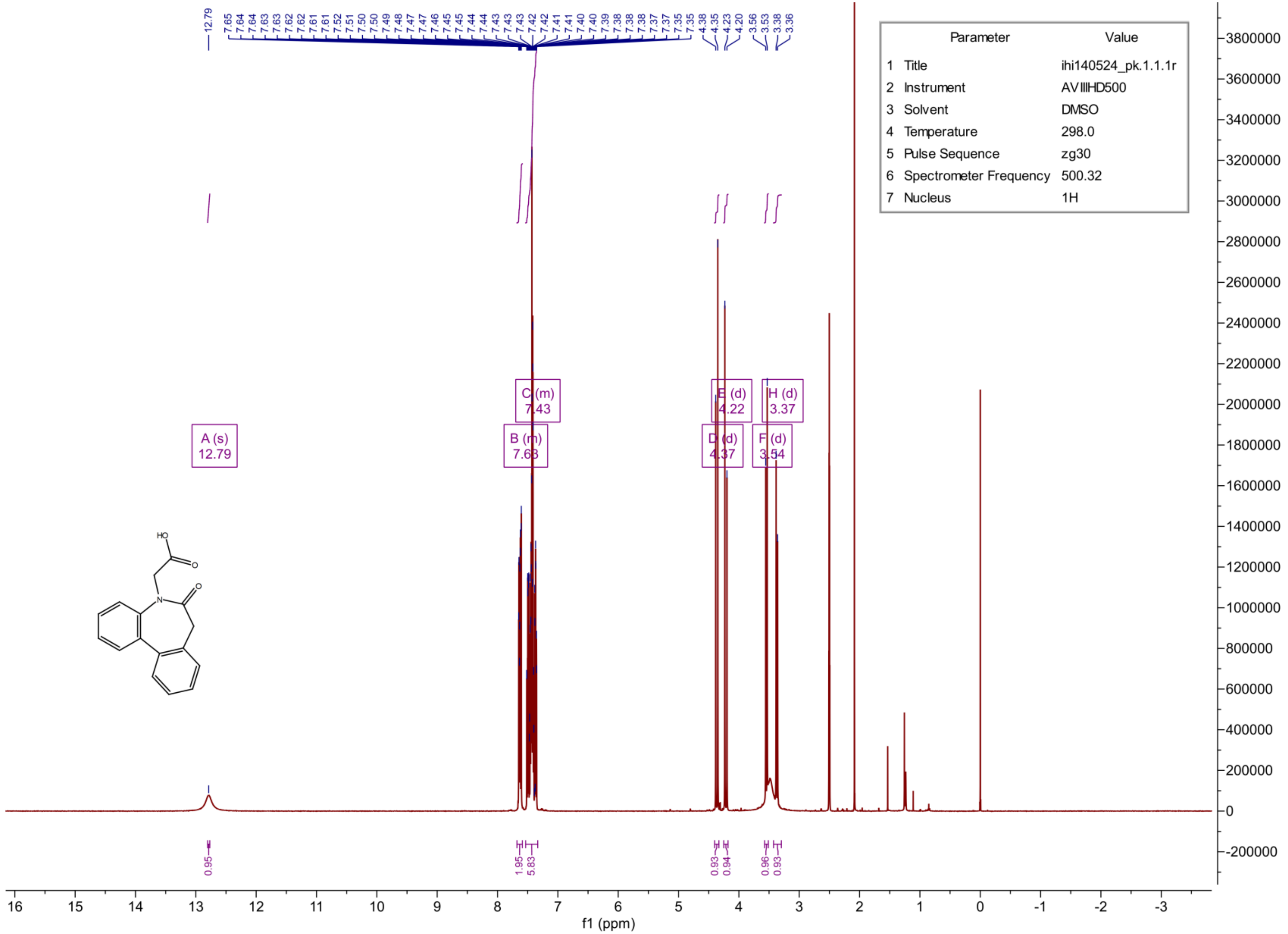

S1 File. Fig 39. ^{1}H-NMR spectrum of compound 19.

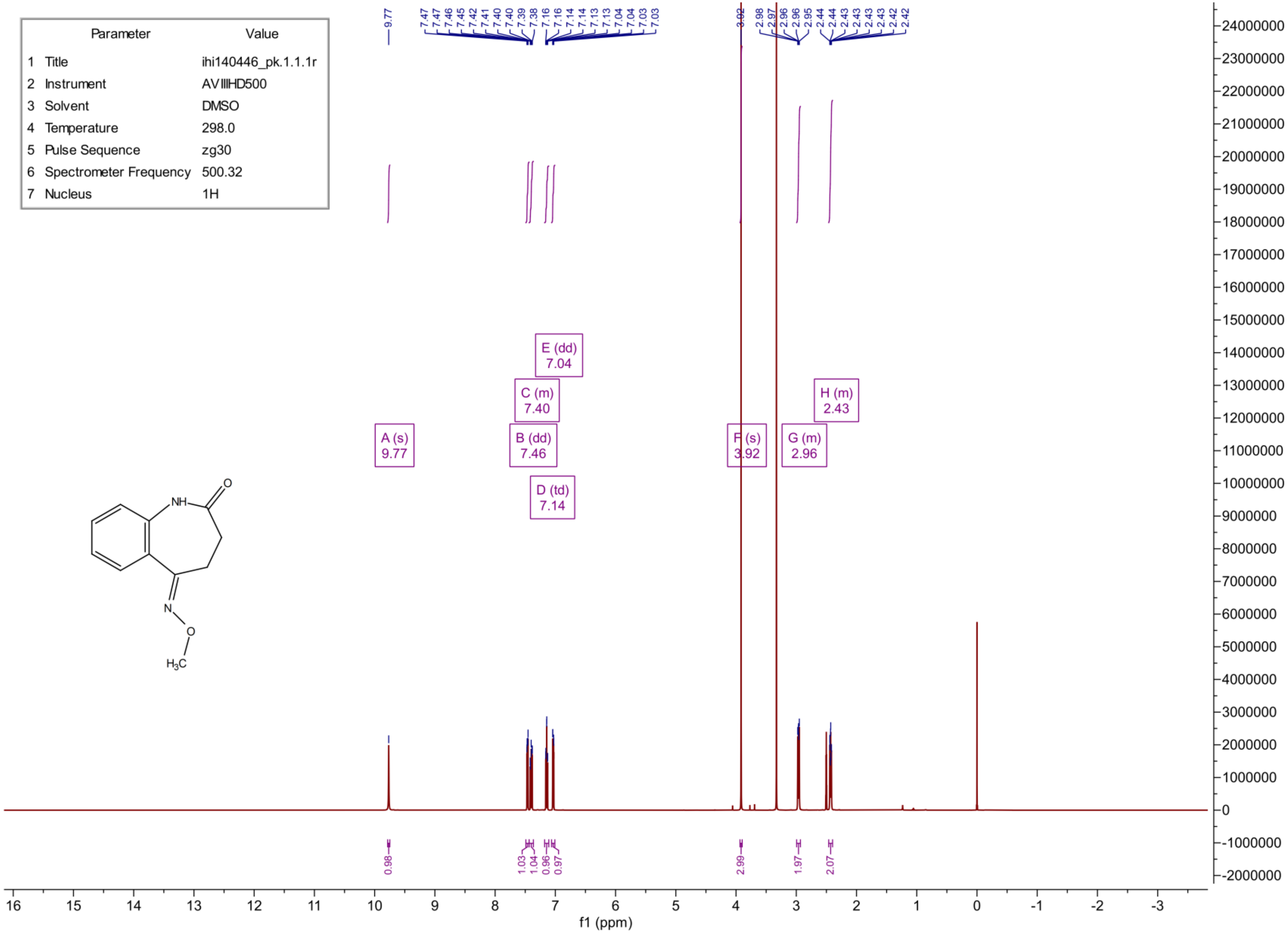

S1 File. Fig 40. ^{1}H-NMR spectrum of compound 20.

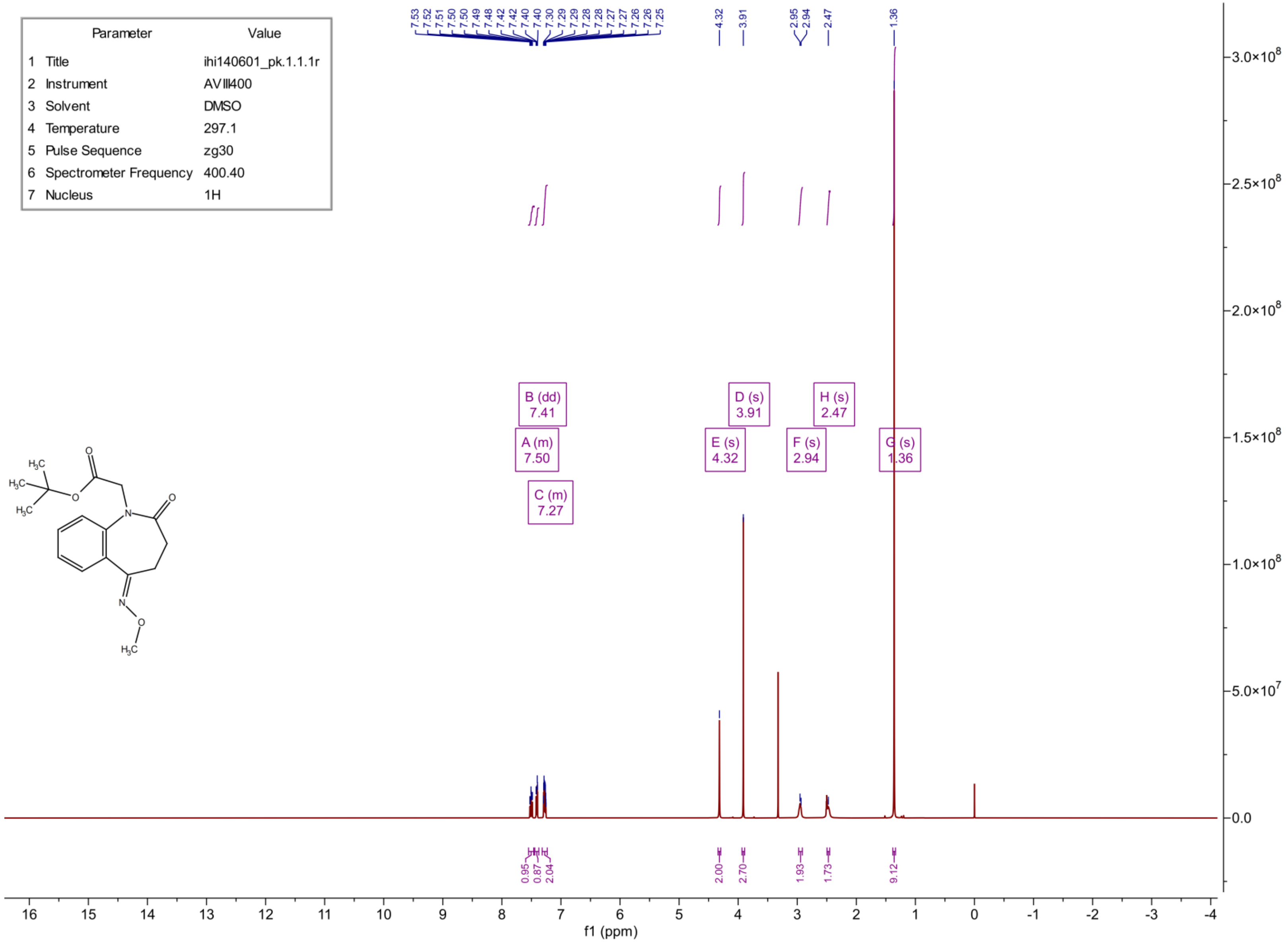

S1 File. Fig 41. ^{1}H-NMR spectrum of compound 21.

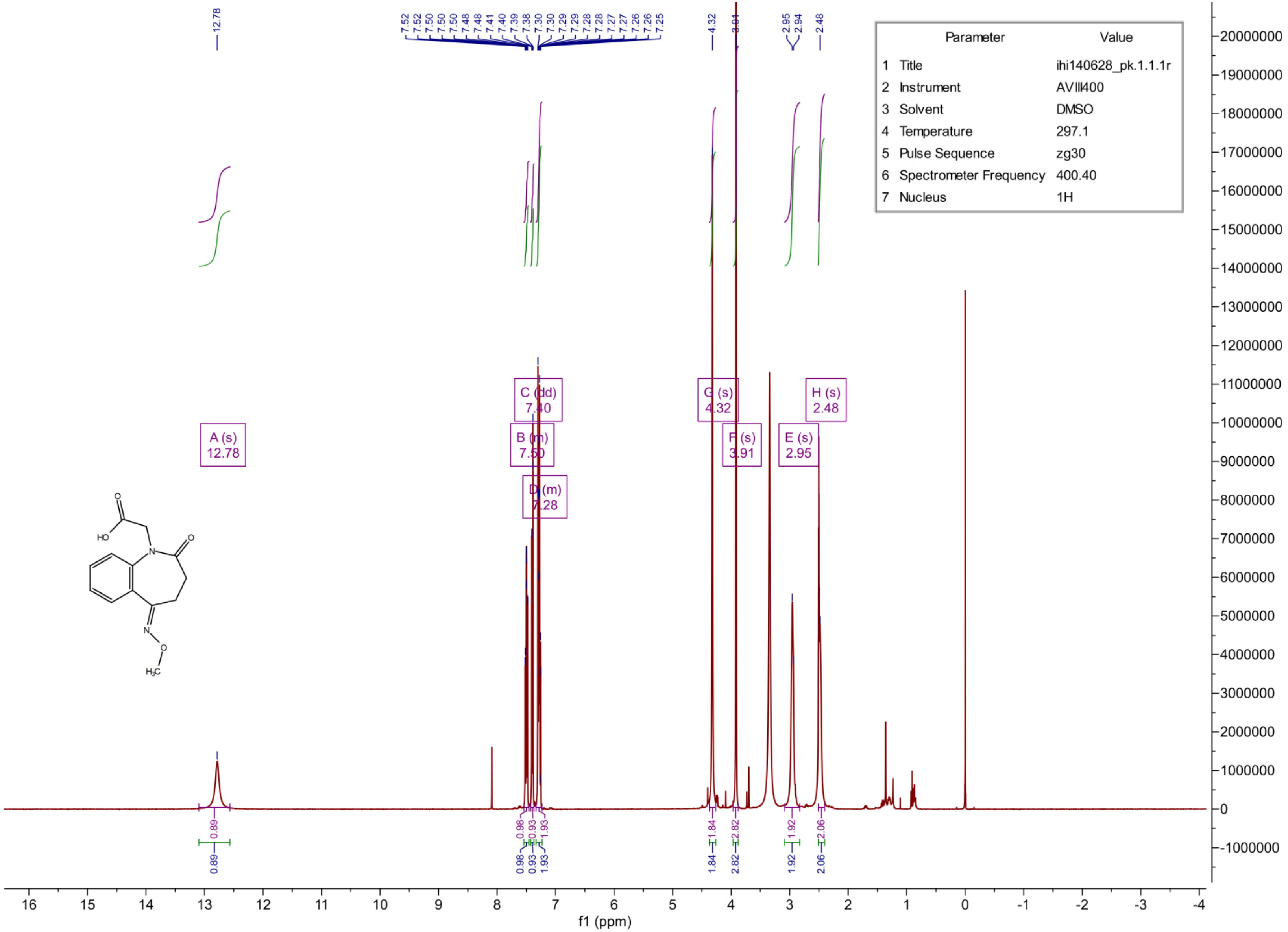

S1 File. Fig 42. ^{1}H-NMR spectrum of compound 22.
