# Supplementary material for: The indole motif is essential for the antitrypanosomal activity of N5-substituted paullones
Source: PLoS One. 2023 Nov 30;18(11):e0292946. doi: 10.1371/journal.pone.0292946 (PMC10688702; doi:10.1371/journal.pone.0292946)
Supplement: S2 File — (PDF) [file pone.0292946.s002.pdf]

The indole motif is essential for the antitrypanosomal activity of N^5-substituted paullones

Irina Ihnatenko, Marco J. Müller, Oliver C. F. Orban, Jens C. Lindhof, Diego Benítez, Cecilia Ortíz, Estefanía Dibello, Leonardo L. Seidl, Marcelo A. Comini and Conrad Kunick*

S3 File. Figs 1–11. ^{1}H-NMR spectra of selected compounds after H/D-exchange.

Table of contents: ^{1}H-NMR spectra of selected compounds after H/D-exchange.

S3 File. Fig 1. ^{1}H-NMR spectrum of compound 1h after H/D-exchange. 3

S3 File. Fig 2. ^{1}H-NMR spectrum of compound 2q after H/D-exchange. 4

S3 File. Fig 3. ^{1}H-NMR spectrum of compound 2r after H/D-exchange. 5

S3 File. Fig 4. ^{1}H-NMR spectrum of compound 2s after H/D-exchange. 6

S3 File. Fig 5. ^{1}H-NMR spectrum of compound 2t after H/D-exchange. 7

S3 File. Fig 6. ^{1}H-NMR spectrum of compound 2u after H/D-exchange. 8

S3 File. Fig 7. ^{1}H-NMR spectrum of compound 2v after H/D-exchange. 9

S3 File. Fig 8. ^{1}H-NMR spectrum of compound 3d after H/D-exchange. 10

S3 File. Fig 9. ^{1}H-NMR spectrum of compound 3e after H/D-exchange. 11

S3 File. Fig 10. ^{1}H-NMR spectrum of compound 5b after H/D-exchange. 12

S3 File. Fig 11. ^{1}H-NMR spectrum of compound 16 after H/D-exchange. 13

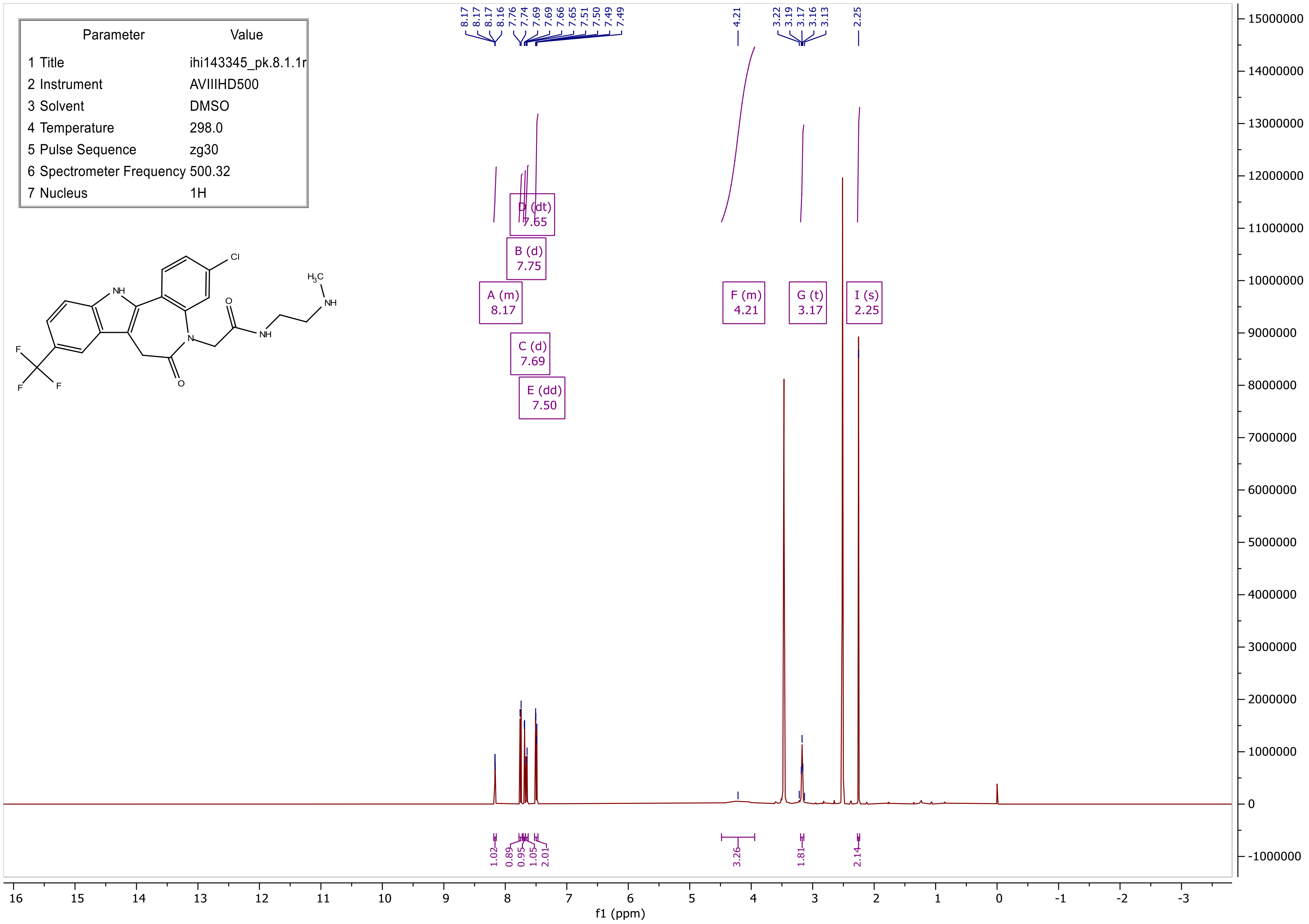

S3 File. Fig 1. ^{1}H-NMR spectrum of compound 1h after H/D-exchange.

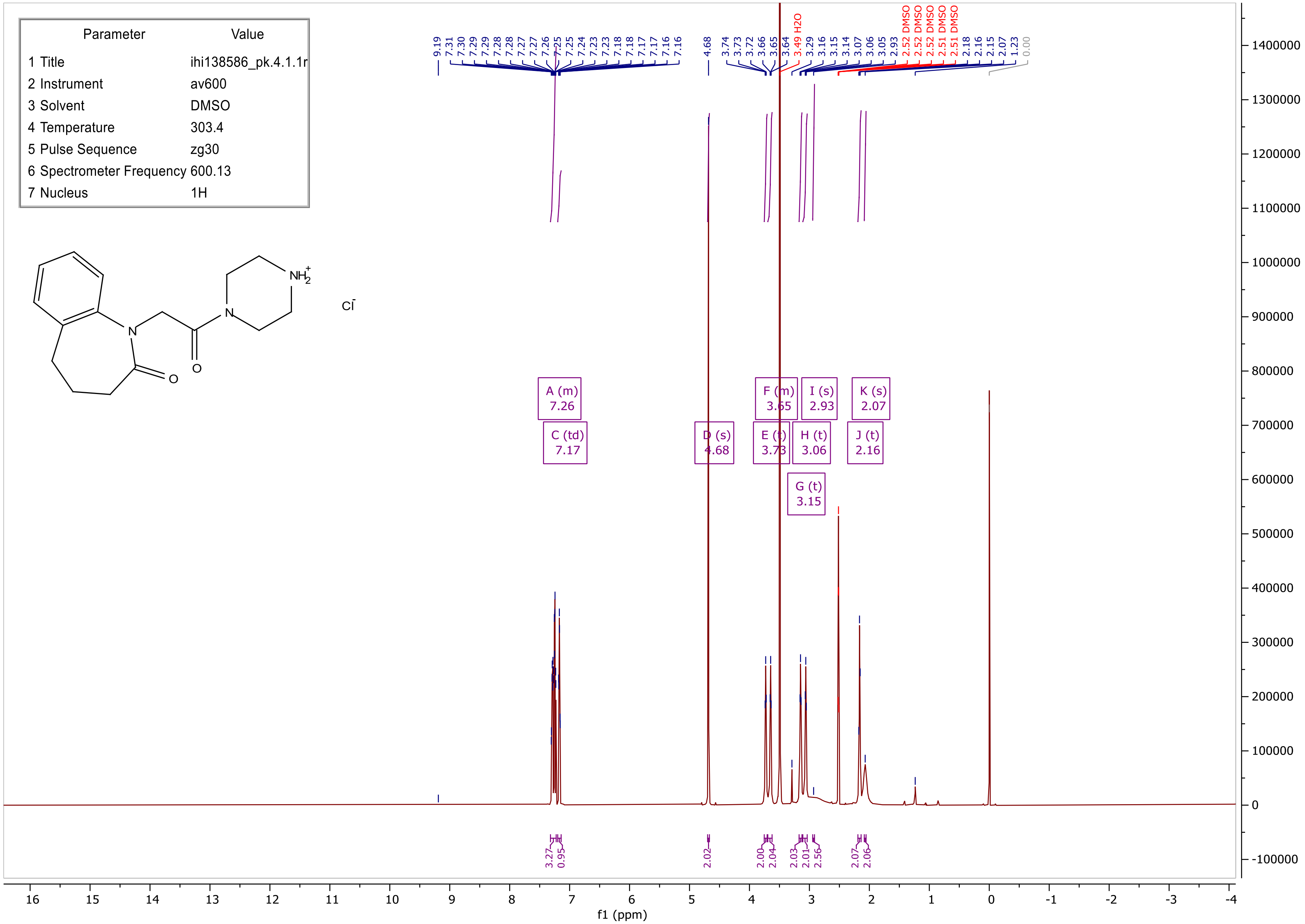

S3 File. Fig 2. [1]**H-NMR spectrum of compound 2q after H/D-exchange.**

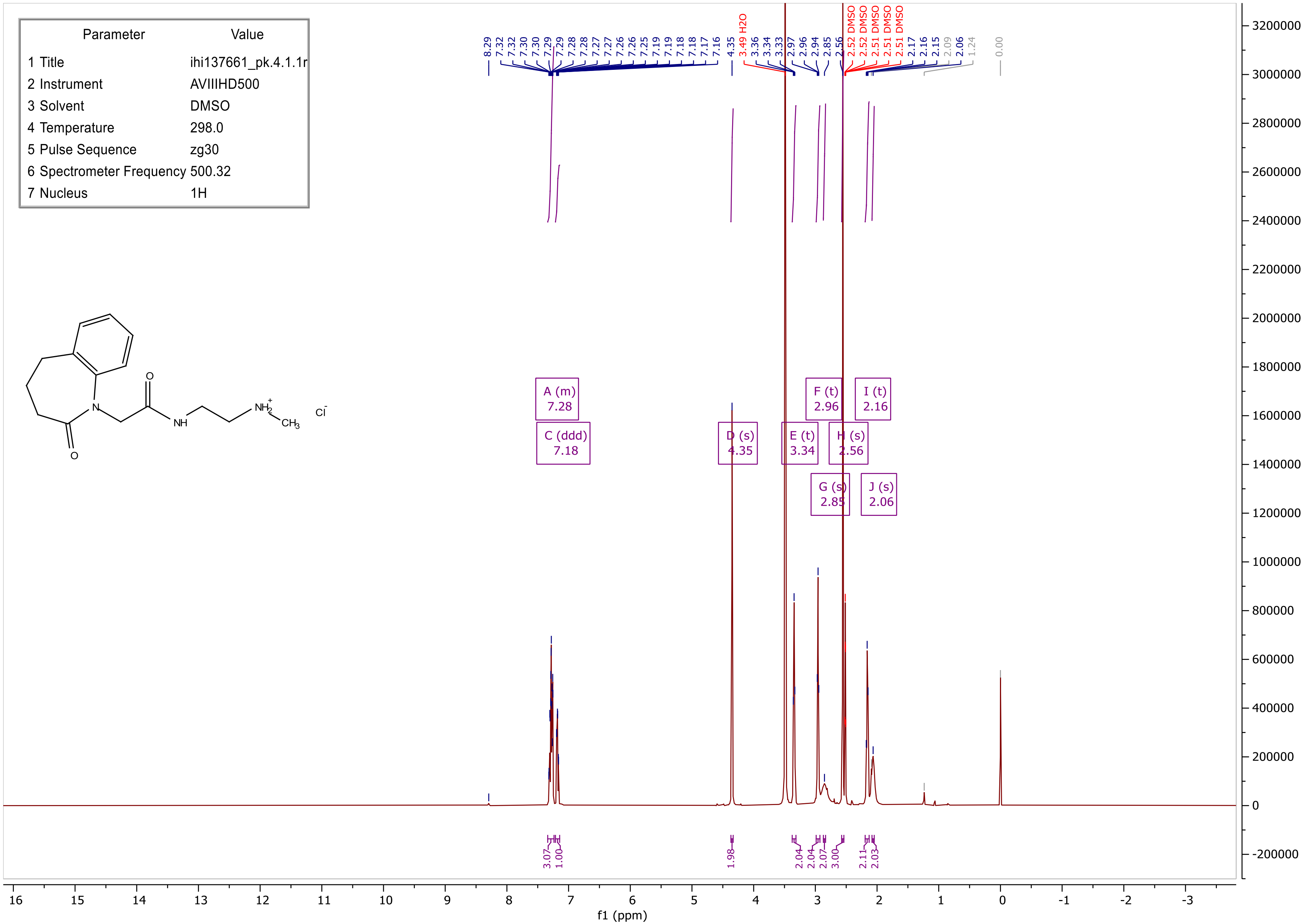

S3 File. Fig 3. ^{1}H-NMR spectrum of compound 2r after H/D-exchange.

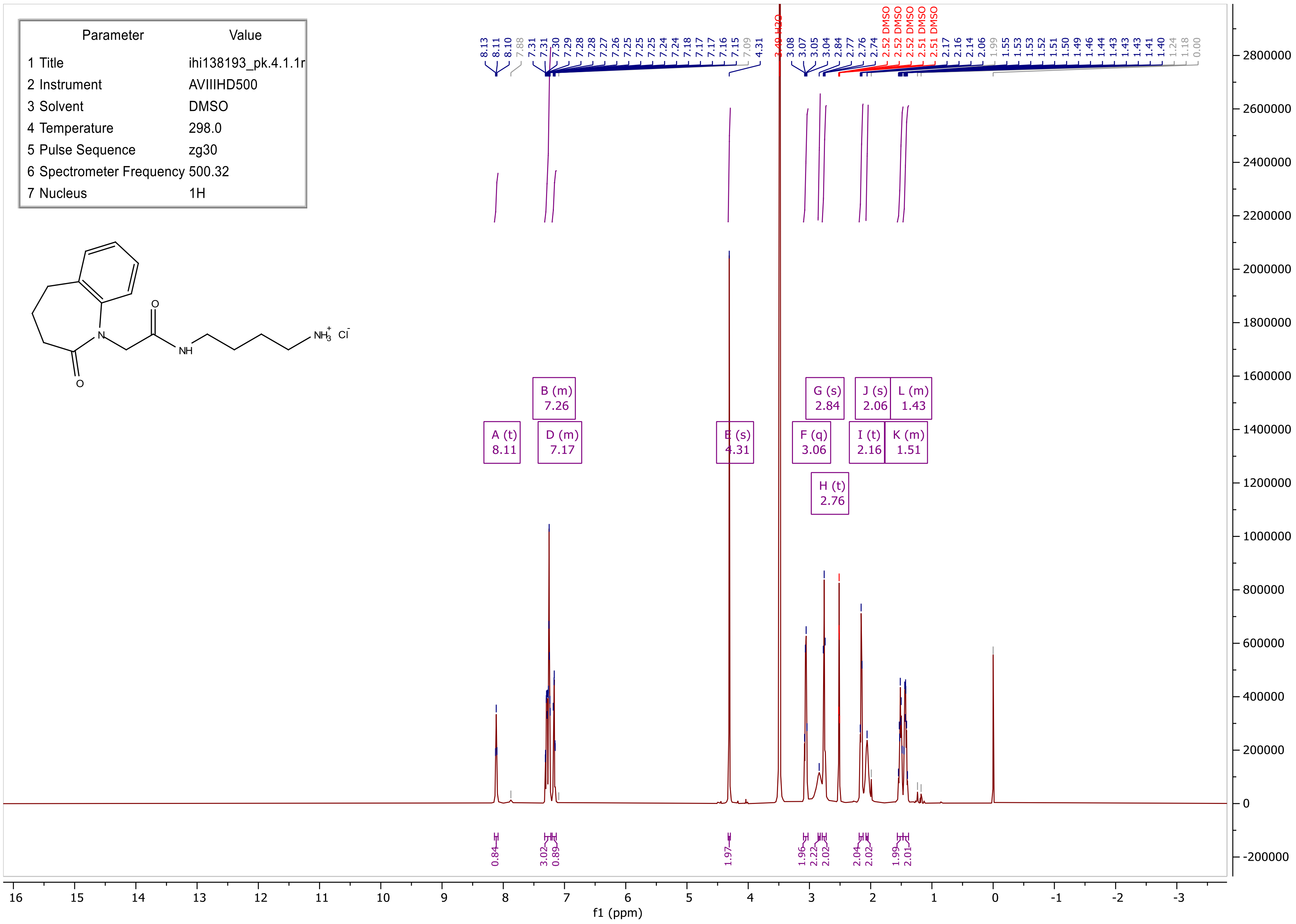

S3 File. Fig 4. **^{1}H-NMR spectrum of compound 2s after H/D-exchange.**

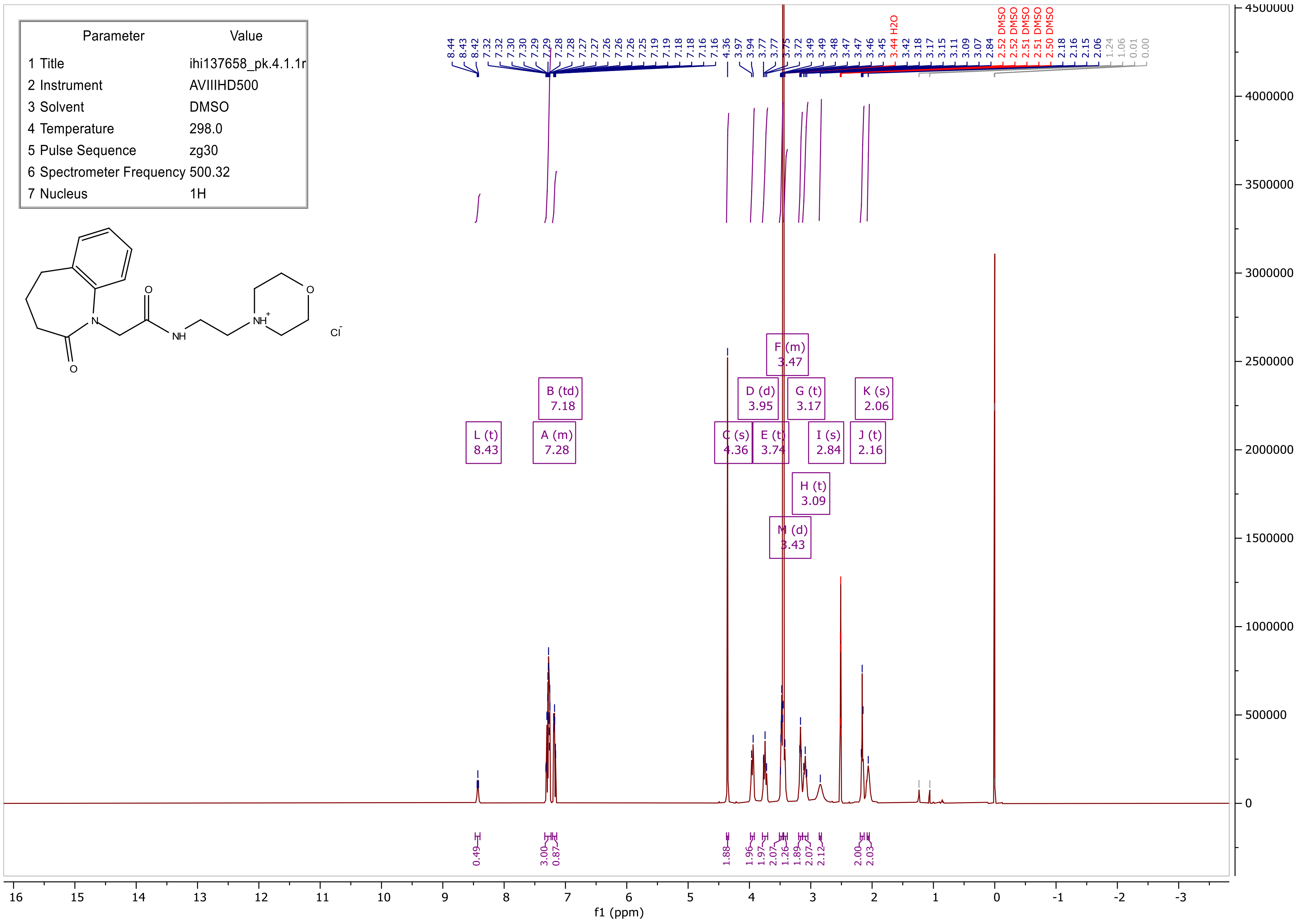

S3 File. Fig 5. ^{1}H-NMR spectrum of compound 2t after H/D-exchange.

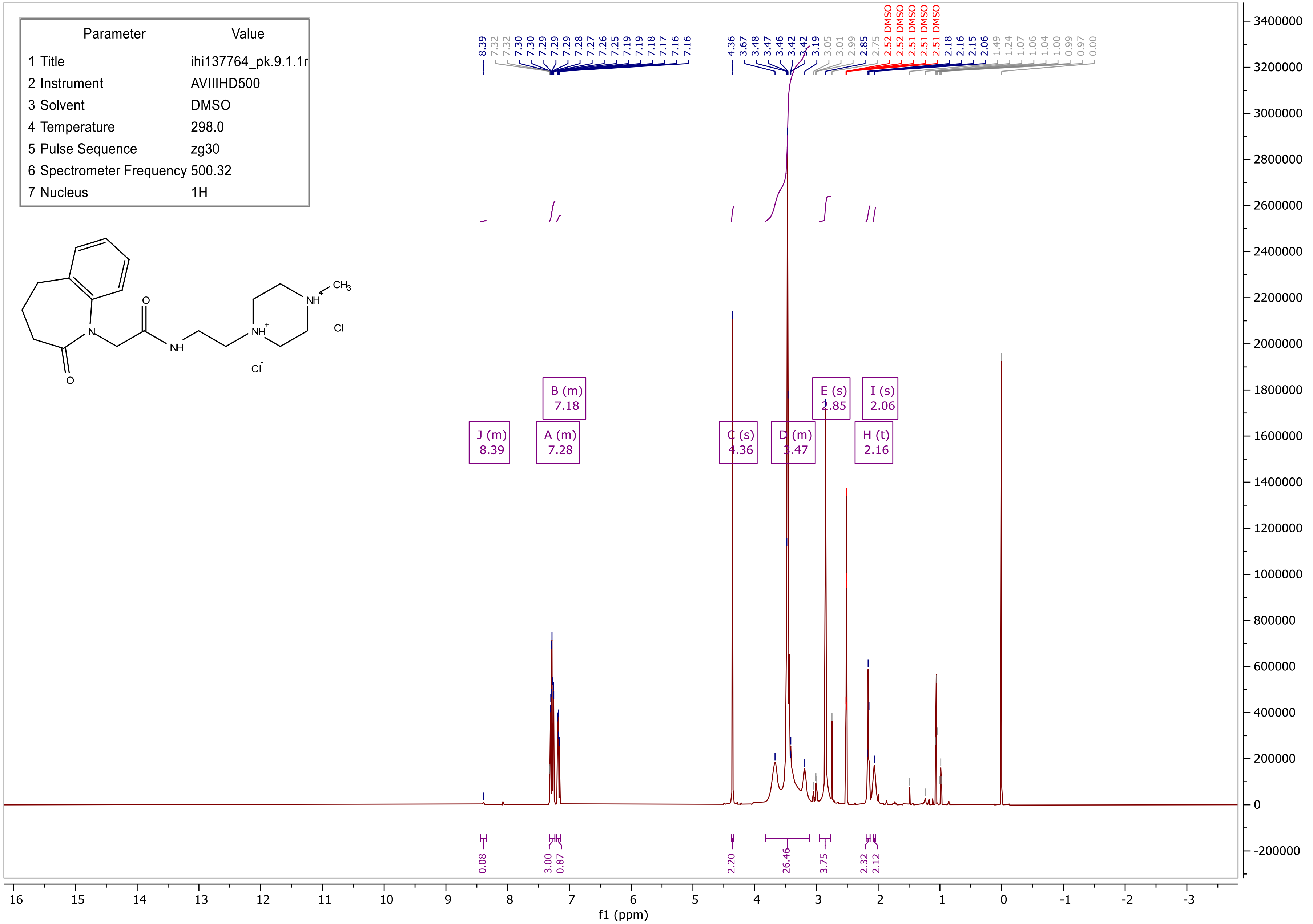

S3 File. Fig 6. ^{1}H-NMR spectrum of compound 2u after H/D-exchange.

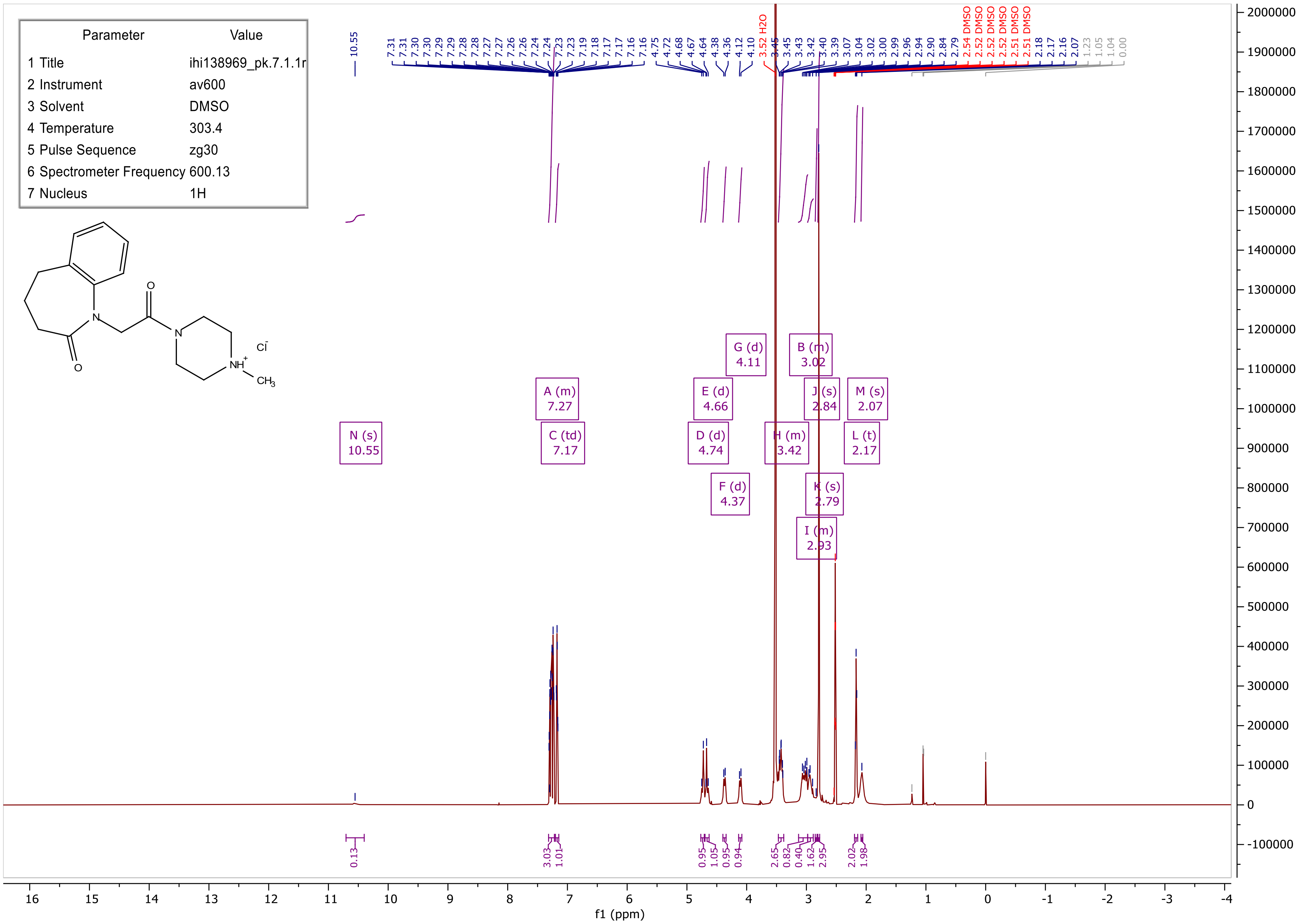

S3 File. Fig 7. **¹H-NMR spectrum of compound 2v after H/D-exchange.**

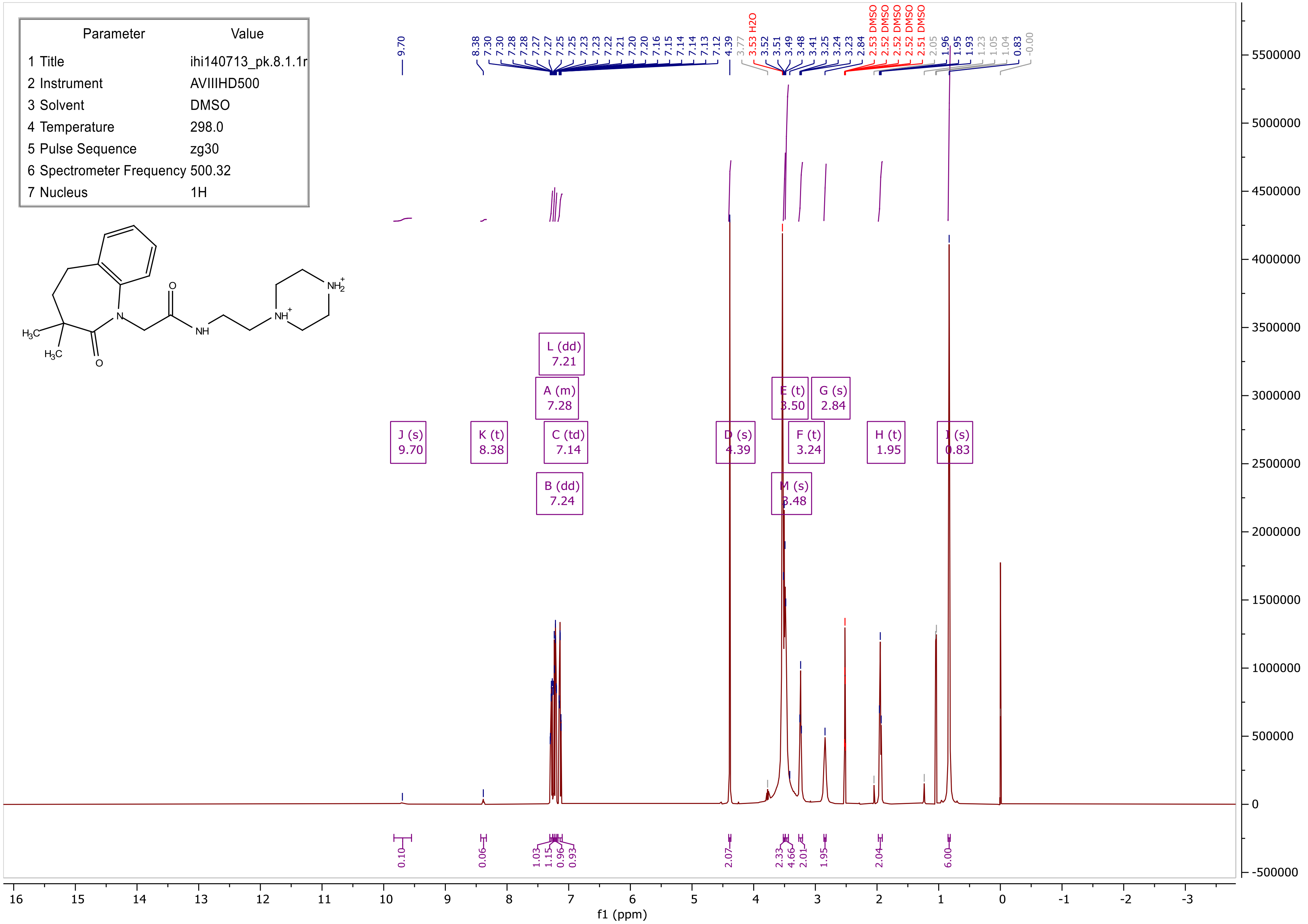

S3 File. Fig 8. ^{1}H-NMR spectrum of compound 3d after H/D-exchange.

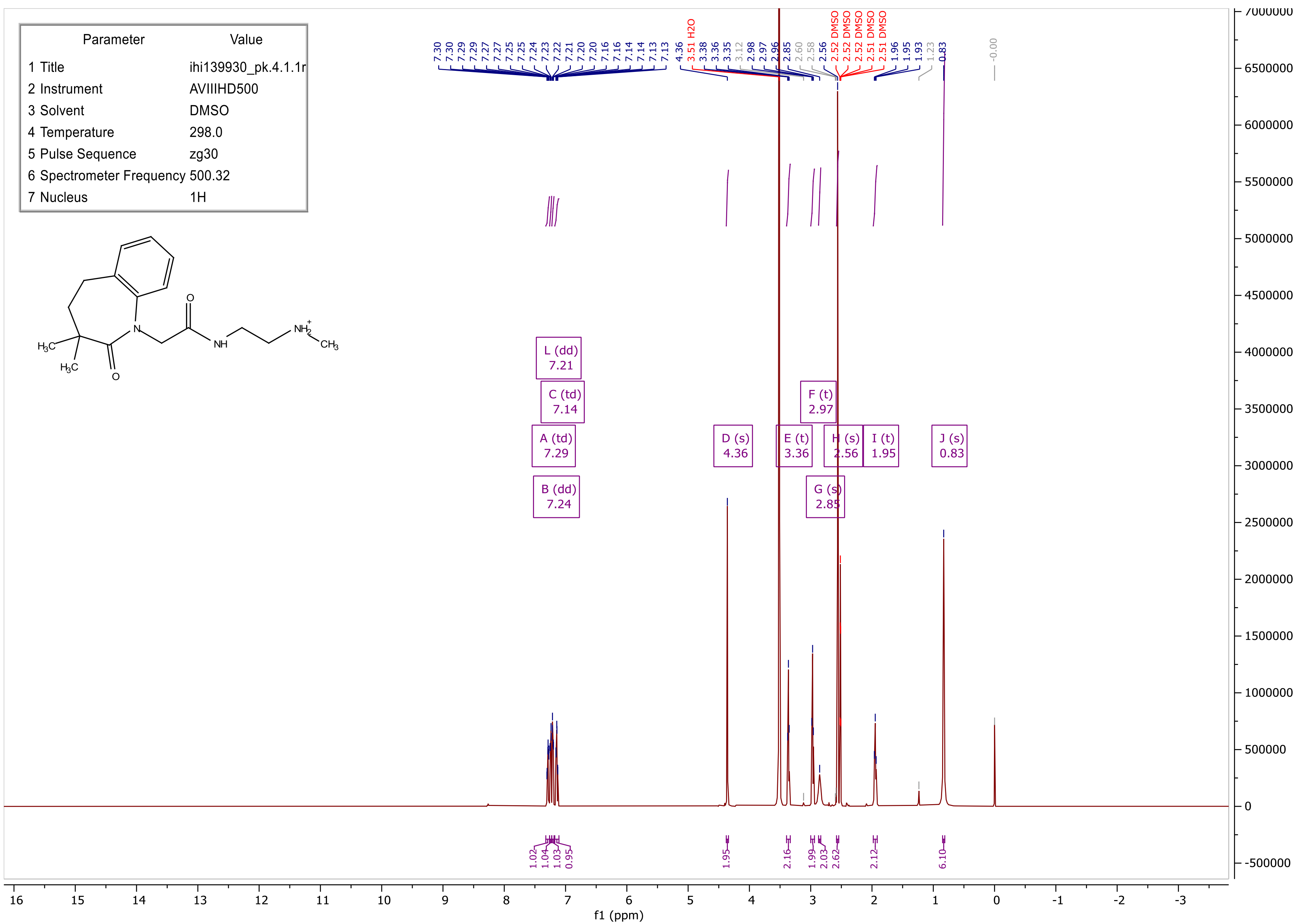

S3 File. Fig 9. ^{1}H-NMR spectrum of compound 3e after H/D-exchange.

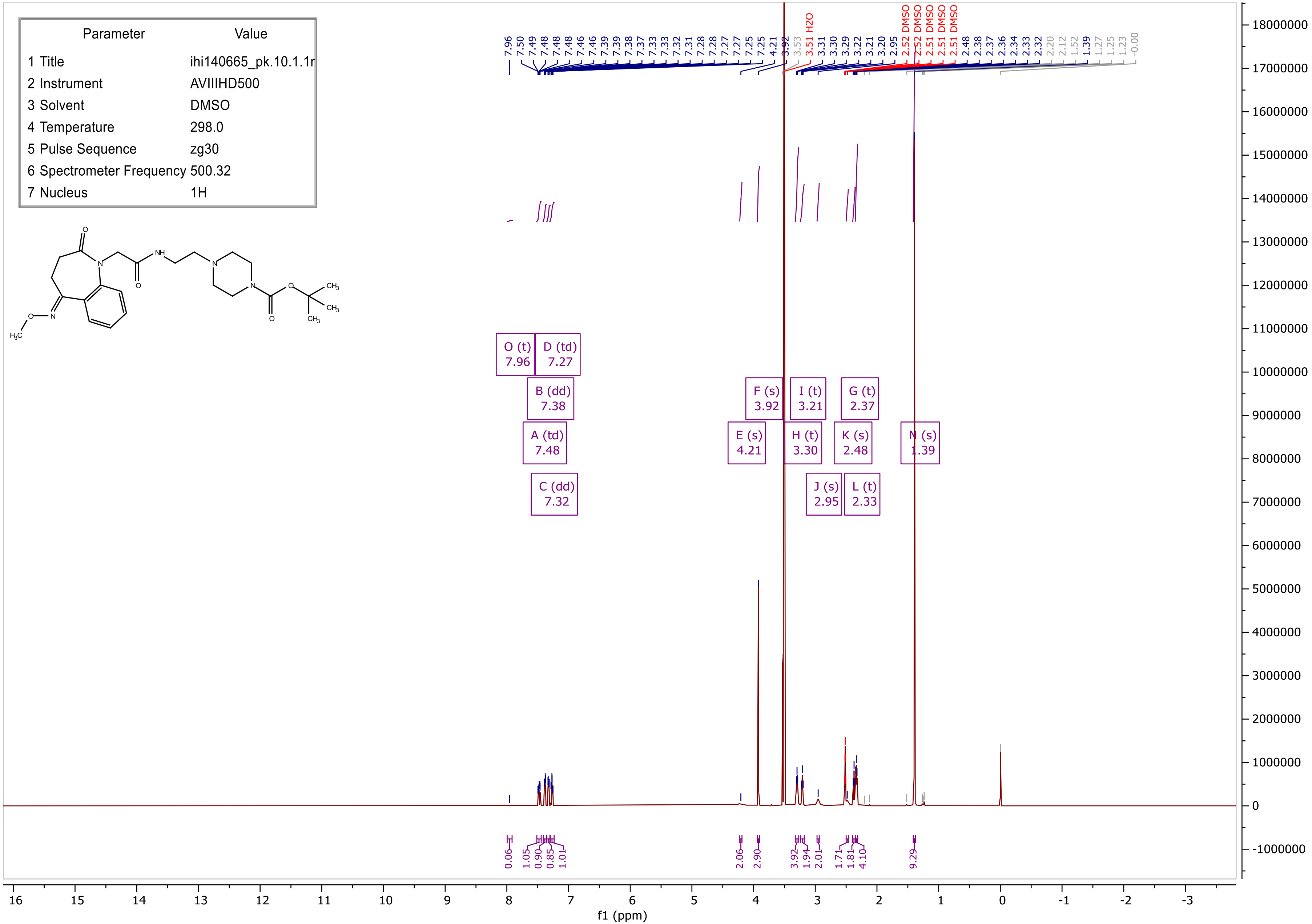

S3 File. Fig 10. ¹H-NMR spectrum of compound 5b after H/D-exchange.

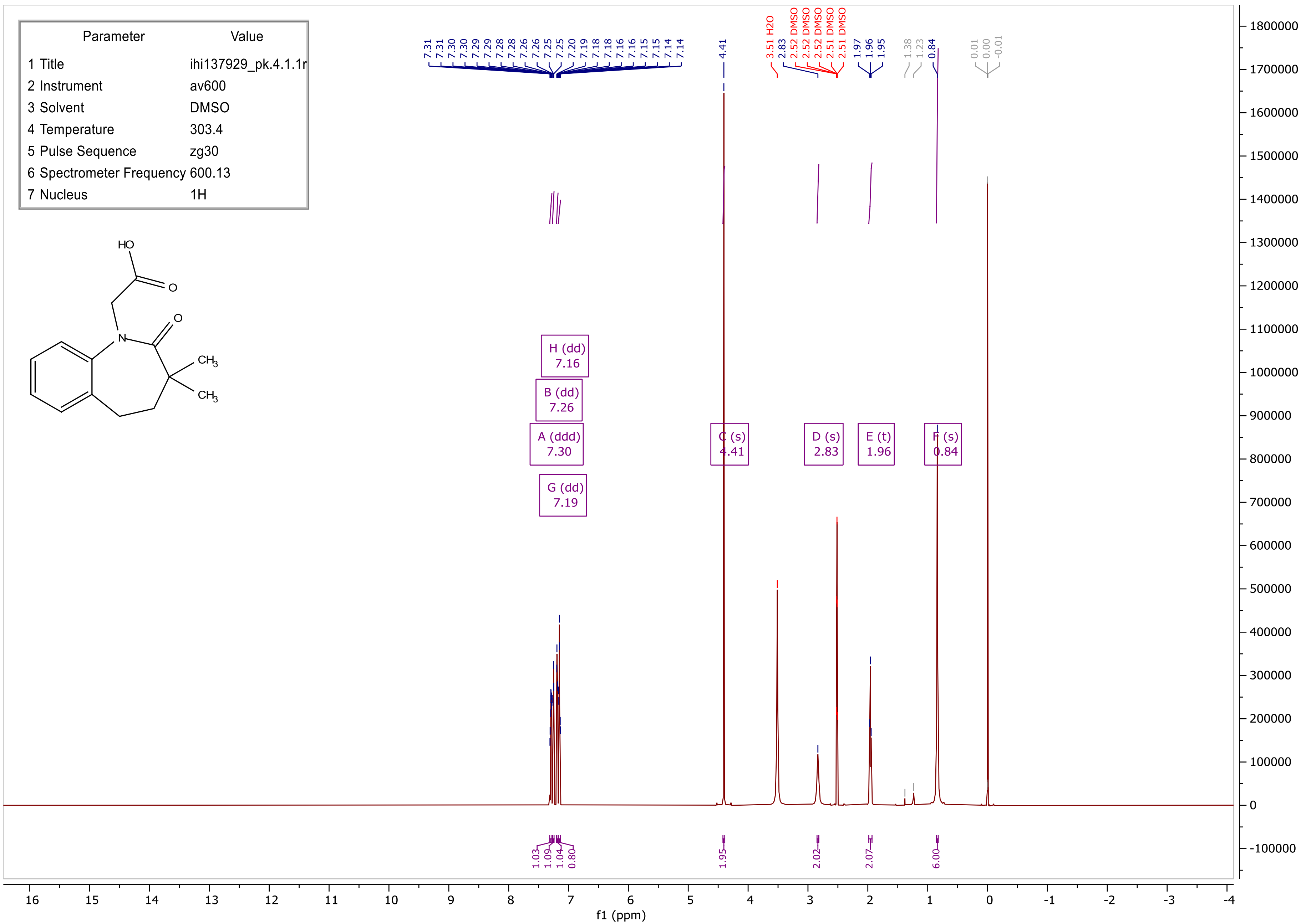

S3 File. Fig 11. ¹H-NMR spectrum of compound 16 after H/D-exchange.
