# Supplementary material for: The indole motif is essential for the antitrypanosomal activity of N5-substituted paullones
Source: PLoS One. 2023 Nov 30;18(11):e0292946. doi: 10.1371/journal.pone.0292946 (PMC10688702; doi:10.1371/journal.pone.0292946)

TU Braunschweig Institut für Pharmazeutische Chemie

Method Name: C:\EZChrom Elite\Enterprise\Projects\Reinheit_Irina\Method\ACN-H2O\ACN-H2O_90-10_1min_0,1µL.met
Data: C:\EZChrom Elite\Enterprise\Projects\Reinheit_Irina\Data\KuIna057 isokratisch_5µL_03.02.2020 15-58-29_ACN-Puffer_30-70_15min.met
User: Irina Ihnatenko
Acquired: 03.02.2020 15:59:36
Printed: 03.02.2020 16:30:06
Sample ID: KuIna057 isokratisch_5µL
Injectionvolume: 5

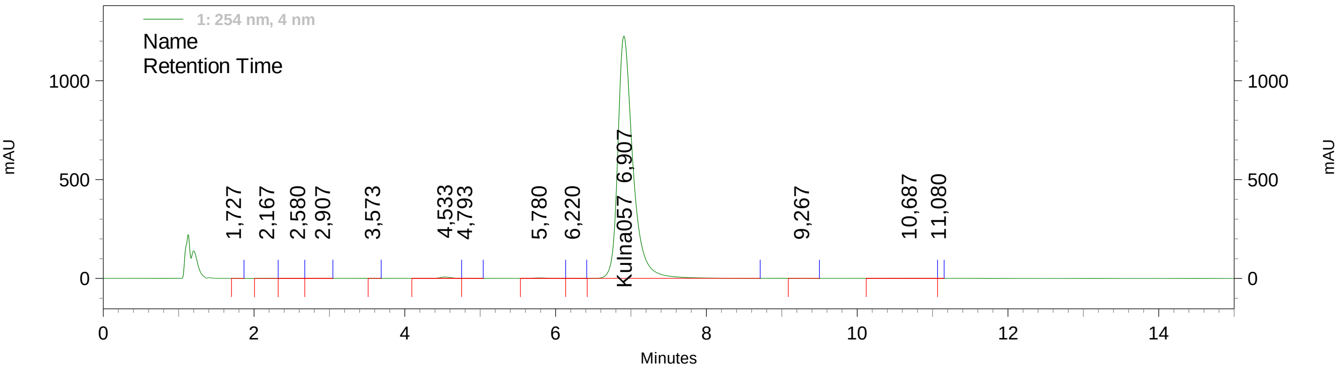

1: 254 nm. 4 nm

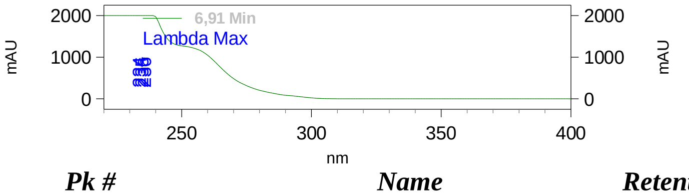

Pk #	*Name*	*Retention Time*	*Area Percent*	*Area*
1		1,727	0,004	2490
2		2,167	0,025	17435
3		2,580	0,017	11739
4		2,907	0,021	14798
5		3,573	0,004	2805
6		4,533	0,435	301132
7		4,793	0,031	21779
8		5,780	0,218	150433
9		6,220	0,021	14646
10	**KuIna057**	6,907	99,024	68473824
11		9,267	0,037	25328
12		10,687	0,161	111329
13		11,080	0,001	867

Totals			100,000	69148605

Method Name: C:\EZChrom Elite\Enterprise\Projects\Reinheit_Irina\Method\ACN-H2O\ACN-H2O_90-10_1min_0,1µL.met
Data: C:\EZChrom Elite\Enterprise\Projects\Reinheit_Irina\Data\KuIna057 isokratisch_5µL_03.02.2020 15-58-29_ACN-Puffer_30-70_15min.met
User: Irina Ihnatenko
Acquired: 03.02.2020 15:59:36
Printed: 03.02.2020 16:30:06
Sample ID: KuIna057 isokratisch_5µL
Injectionvolume: 5

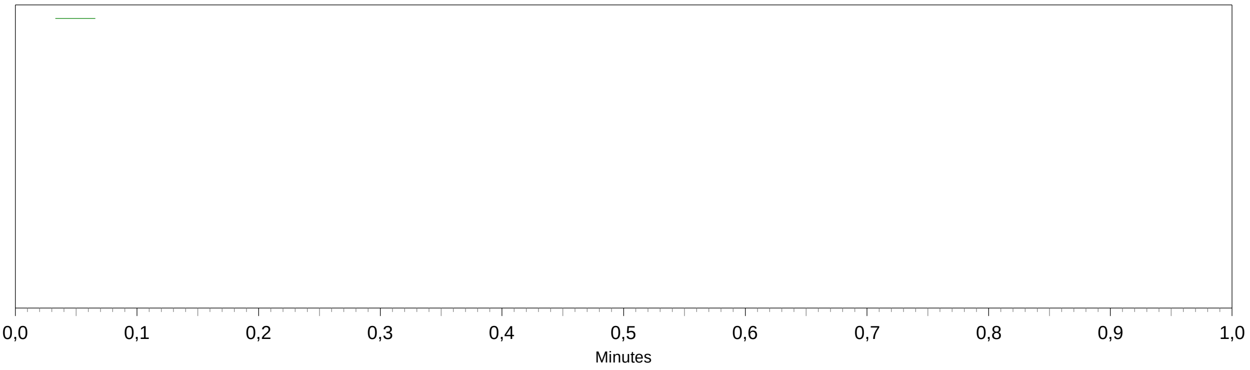

Pk #	*Name*	*Retention Time*	*Area Percent*	*Area*

Spectrum Report

Spectra of all named detected peaks

(The peak spectrum is defined as the peak apex spectrum)

Multi-Chrom 1 (1: 254 nm, 4 nm) Spectra

Retention time: 6,907 Min
Peak name: KuIna057
Lambda max: 236, 235, 234
Lambda min: 378, 343, 327

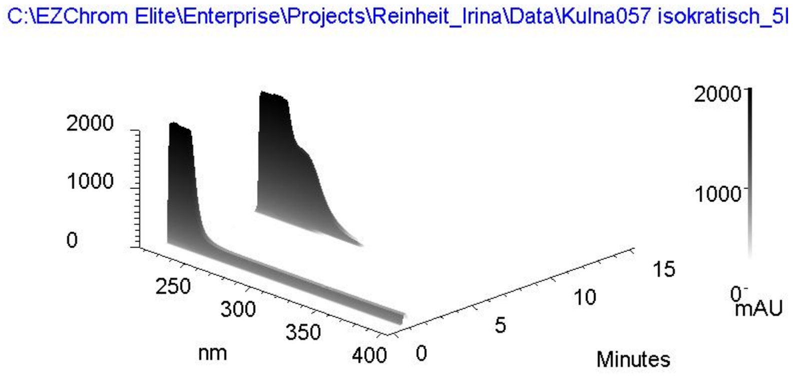

Supplement: S3 File — (ZIP) [file pone.0292946.s003.zip › S4_ZIP-File_HPLC_chromatograms/HPLC-Merck-cmpd-19-iso-254nm.pdf]
