# Supplementary material for: The indole motif is essential for the antitrypanosomal activity of N5-substituted paullones
Source: PLoS One. 2023 Nov 30;18(11):e0292946. doi: 10.1371/journal.pone.0292946 (PMC10688702; doi:10.1371/journal.pone.0292946)

TU Braunschweig Institut für Pharmazeutische Chemie

Method Name: C:\EZChrom Elite\Enterprise\Projects\Reinheit_Irina\Method\ACN-H2O\ACN-H2O_10-90_10min.met
Data: C:\EZChrom Elite\Enterprise\Projects\Reinheit_Irina\Data\KuIna097_10µL_17.08.2020 12-47-56_ACN-Puffer_35-65_15min.met
User: Irina Ihnatenko
Acquired: 17.08.2020 12:49:05
Printed: 17.08.2020 13:10:27
Sample ID: KuIna097_10µL
Injectionvolume: 10

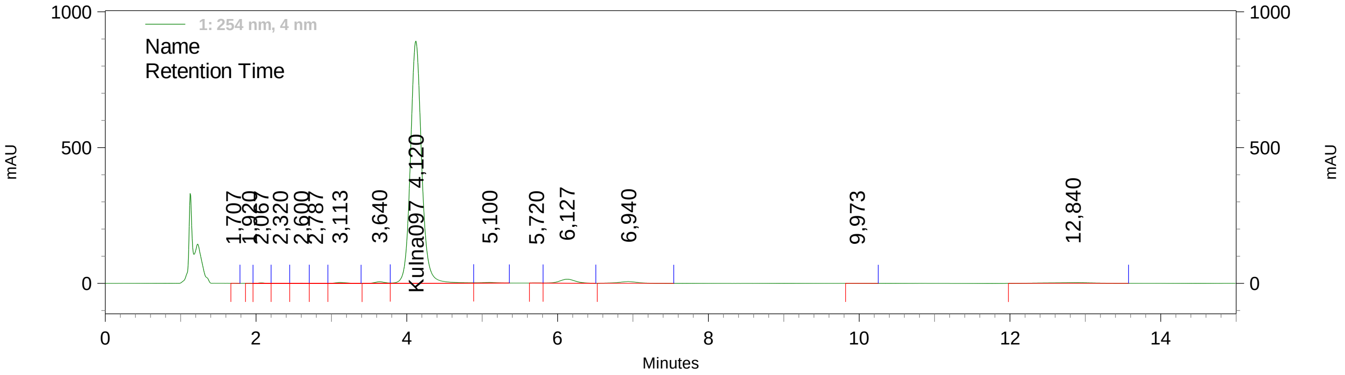

1: 254 nm. 4 nm

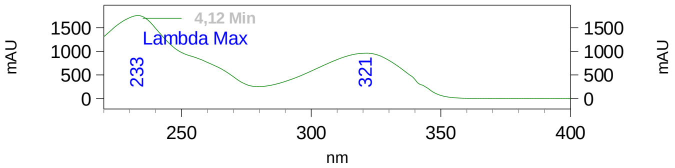

Pk #	*Name*	*Retention Time*	*Area Percent*	*Area*
1		1,707	0,007	2574
2		1,920	0,002	933
3		2,067	0,095	37248
4		2,320	0,024	9356
5		2,600	0,046	18150
6		2,787	0,035	13760
7		3,113	0,332	129717
8		3,640	0,518	202094
9	**KuIna097**	4,120	94,065	36725291
10		5,100	0,361	141093
11		5,720	0,017	6593
12		6,127	2,110	823897
13		6,940	1,105	431403
14		9,973	0,041	16084
15		12,840	1,240	484156

Totals				
			100,000	39042349

Method Name: **C:\EZChrom Elite\Enterprise\Projects\Reinheit_Irina\Method\ACN-H2O\ACN-H2O_10-90_10min.met**
Data: **C:\EZChrom Elite\Enterprise\Projects\Reinheit_Irina\Data\KuIna097_10µL_17.08.2020 12-47-56_ACN-Puffer_35-65_15min.met**
User: **Irina Ihnatenko**
Acquired: **17.08.2020 12:49:05**
Printed: **17.08.2020 13:10:27**
Sample ID: **KuIna097_10µL**
Injectionvolume: **10**

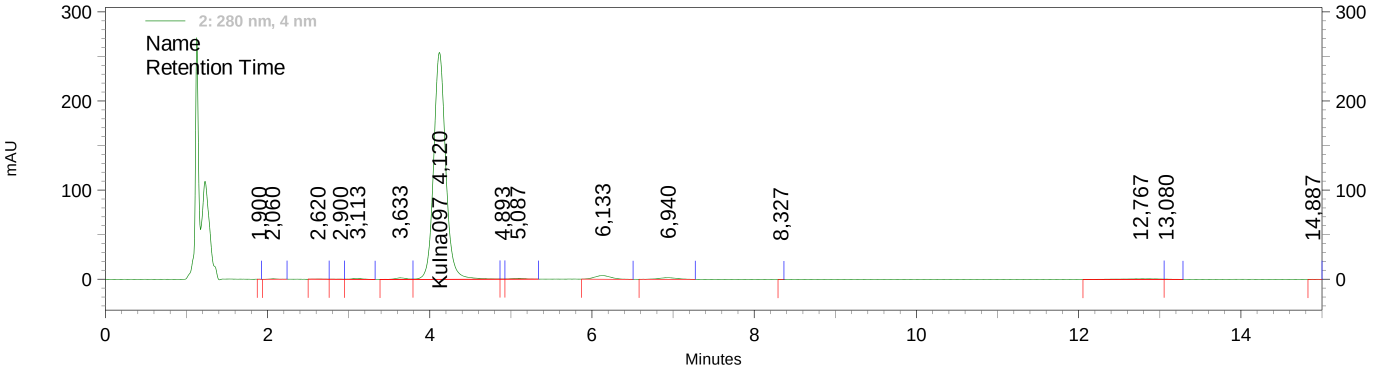

2: 280 nm, 4 nm
Results

Pk #	*Name*	*Retention Time*	*Area Percent*	*Area*
1		1,900	0,008	868
2		2,060	0,115	12879
3		2,620	0,089	9977
4		2,900	0,068	7626
5		3,113	0,366	40888
6		3,633	0,570	63705
7	**KuIna097**	4,120	94,149	10521713
8		4,893	0,044	4892
9		5,087	0,479	53540
10		6,133	2,042	228194
11		6,940	1,052	117556
12		8,327	0,007	807
13		12,767	0,901	100666
14		13,080	0,090	10032
15		14,887	0,020	2196
Totals			100,000	11175539

Spectrum Report
Spectra of all named detected peaks

(The peak spectrum is defined as the peak apex spectrum)

Multi-Chrom 1 (1: 254 nm, 4 nm) Spectra

Method Name: **C:\EZChrom Elite\Enterprise\Projects\Reinheit_Irina\Method\ACN-H2O\ACN-H2O_10-90_10min.met**
Data: **C:\EZChrom Elite\Enterprise\Projects\Reinheit_Irina\Data\KuIna097_10µL_17.08.2020 12-47-56_ACN-Puffer_35-65_15min.met**
User: **Irina Ihnatenko**
Acquired: **17.08.2020 12:49:05**
Printed: **17.08.2020 13:10:27**
Sample ID: **KuIna097_10µL**
Injectionvolume: **10**

Retention time: 4,120 Min
Peak name: KuIna097
Lambda max: 233, 321
Lambda min: 280

Multi-Chrom 2 (2: 280 nm, 4 nm) Spectra

Retention time: 4,120 Min
Peak name: KuIna097
Lambda max: 233, 321
Lambda min: 280

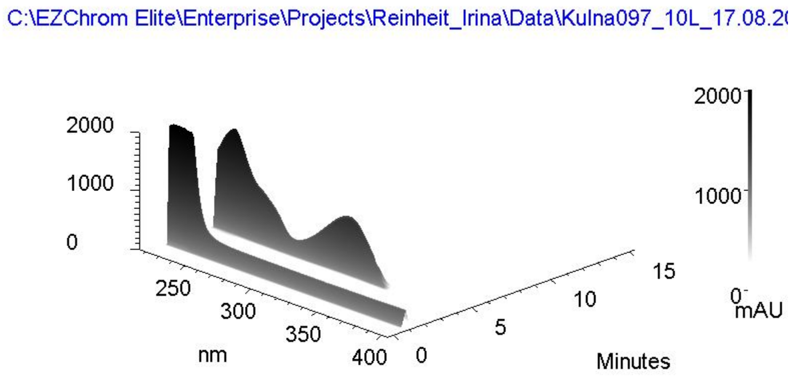

Supplement: S3 File — (ZIP) [file pone.0292946.s003.zip › S4_ZIP-File_HPLC_chromatograms/HPLC-Merck-cmpd-1f-iso-254+280nm.pdf]
