# Supplementary material for: The indole motif is essential for the antitrypanosomal activity of N5-substituted paullones
Source: PLoS One. 2023 Nov 30;18(11):e0292946. doi: 10.1371/journal.pone.0292946 (PMC10688702; doi:10.1371/journal.pone.0292946)

TU Braunschweig Institut für Pharmazeutische Chemie

Method Name: C:\EZChrom Elite\Enterprise\Projects\Reinheit_Irina\Method\ACN-H2O\ACN-H2O_10-90_10min.met
Data: C:\EZChrom Elite\Enterprise\Projects\Reinheit_Irina\Data\KuIna098_3µL_17.08.2020 13-58-12_ACN-Puffer_35-65_15min.met
User: Irina Ihnatenko
Acquired: 17.08.2020 13:59:13
Printed: 17.08.2020 14:27:43
Sample ID: KuIna098_3µL
Injectionvolume: 3

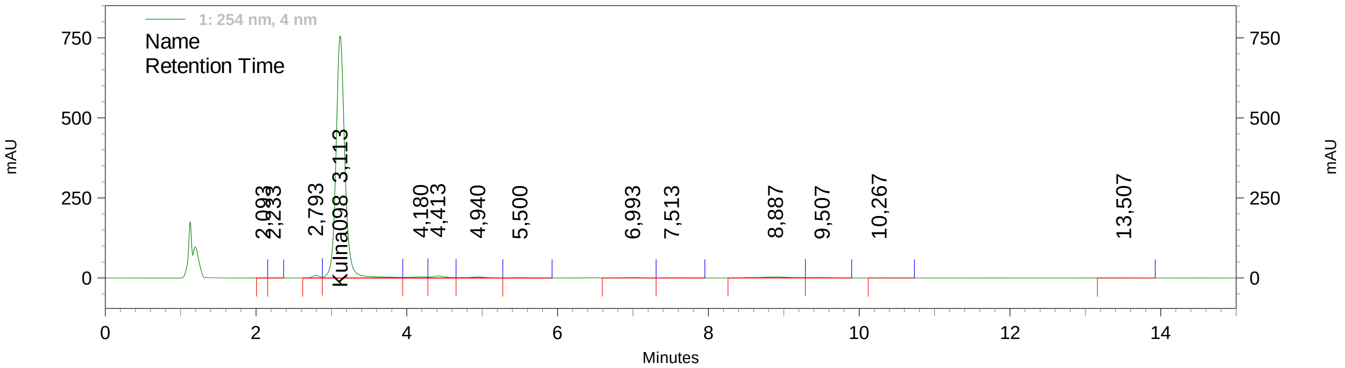

1: 254 nm. 4 nm

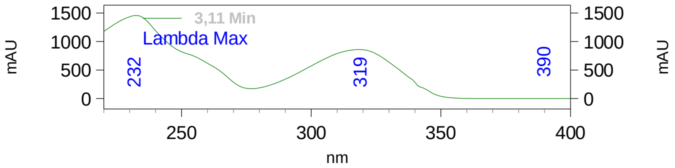

Pk #	*Name*	*Retention Time*	*Area Percent*	*Area*
1		2,093	0,022	5797
2		2,233	0,050	13308
3		2,793	0,816	216867
4	**KuIna098**	3,113	93,467	24841827
5		4,180	0,837	222538
6		4,413	1,174	312041
7		4,940	0,756	200814
8		5,500	0,270	71832
9		6,993	0,279	74269
10		7,513	0,151	40112
11		8,887	1,440	382618
12		9,507	0,399	106137
13		10,267	0,111	29596
14		13,507	0,227	60371

Totals				
			100,000	26578127

Method Name: C:\EZChrom Elite\Enterprise\Projects\Reinheit_Irina\Method\ACN-H2O\ACN-H2O_10-90_10min.met
Data: C:\EZChrom Elite\Enterprise\Projects\Reinheit_Irina\Data\KuIna098_3µL_17.08.2020 13-58-12_ACN-Puffer_35-65_15min.met
User: Irina Ihnatenko
Acquired: 17.08.2020 13:59:13
Printed: 17.08.2020 14:27:43
Sample ID: KuIna098_3µL
Injectionvolume: 3

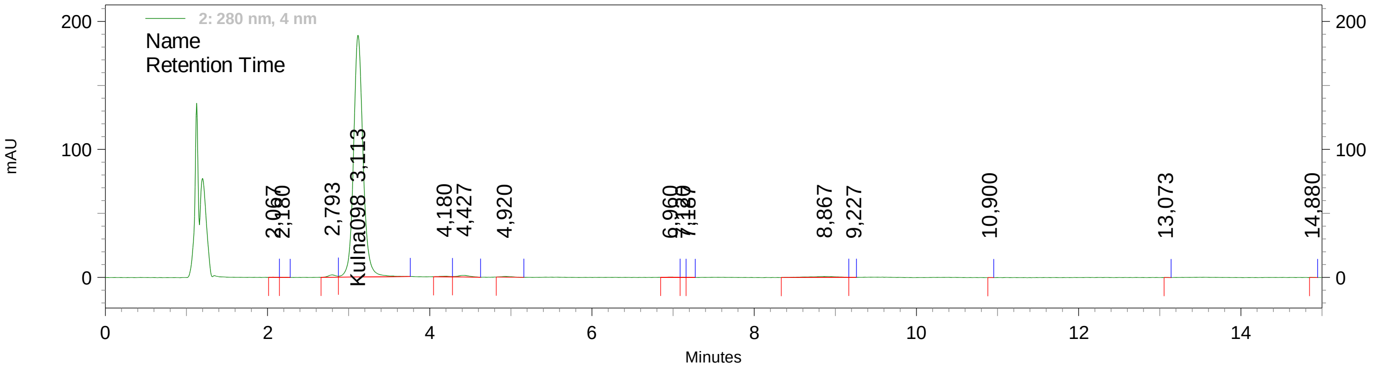

2: 280 nm, 4 nm
Results

Pk #	*Name*	*Retention Time*	*Area Percent*	*Area*
1		2,067	0,048	3096
2		2,180	0,031	2010
3		2,793	0,738	47126
4	**KuIna098**	3,113	96,395	6156082
5		4,180	0,253	16166
6		4,427	0,817	52149
7		4,920	0,329	20980
8		6,960	0,136	8675
9		7,120	0,036	2328
10		7,187	0,028	1794
11		8,867	1,124	71761
12		9,227	0,021	1363
13		10,900	0,014	890
14		13,073	0,013	826
15		14,880	0,017	1069
Totals			100,000	6386315

Spectrum Report
Spectra of all named detected peaks

(The peak spectrum is defined as the peak apex spectrum)

Multi-Chrom 1 (1: 254 nm, 4 nm) Spectra

Method Name: **C:\EZChrom Elite\Enterprise\Projects\Reinheit_Irina\Method\ACN-H2O\ACN-H2O_10-90_10min.met**
Data: **C:\EZChrom Elite\Enterprise\Projects\Reinheit_Irina\Data\KuIna098_3µL_17.08.2020 13-58-12_ACN-Puffer_35-65_15min.met**
User: **Irina Ihnatenko**
Acquired: **17.08.2020 13:59:13**
Printed: **17.08.2020 14:27:43**
Sample ID: **KuIna098_3µL**
Injectionvolume: **3**

Retention time: 3,113 Min
Peak name: KuIna098
Lambda max: 232, 319, 390
Lambda min: 385, 277

Multi-Chrom 2 (2: 280 nm, 4 nm) Spectra

Retention time: 3,113 Min
Peak name: KuIna098
Lambda max: 232, 319, 390
Lambda min: 385, 277

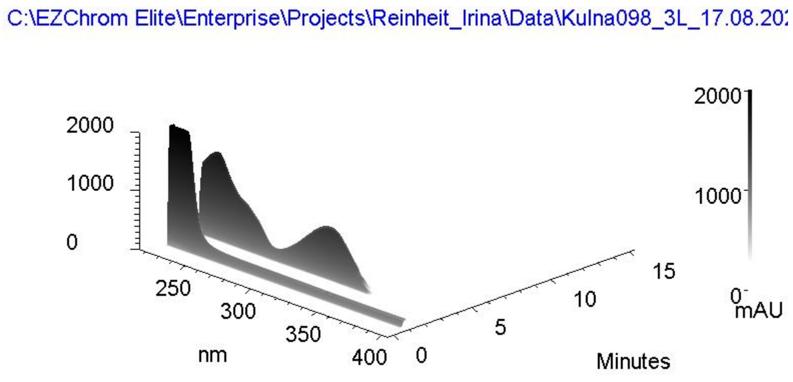

Supplement: S3 File — (ZIP) [file pone.0292946.s003.zip › S4_ZIP-File_HPLC_chromatograms/HPLC-Merck-cmpd-1g-iso-254+280nm.pdf]
