# Supplementary material for: The indole motif is essential for the antitrypanosomal activity of N5-substituted paullones
Source: PLoS One. 2023 Nov 30;18(11):e0292946. doi: 10.1371/journal.pone.0292946 (PMC10688702; doi:10.1371/journal.pone.0292946)

TU Braunschweig Institut für Pharmazeutische Chemie

Method Name: C:\EZChrom Elite\Enterprise\Projects\Reinheit_Irina\Method\ACN-H2O\ACN-H2O_10-90_10min.met
Data: C:\EZChrom Elite\Enterprise\Projects\Reinheit_Irina\Data\KuIna103_8µL_02.09.2020 15-23-16_ACN-Puffer_35-65_15min.met
User: Irina Ihnatenko
Acquired: 02.09.2020 15:24:22
Printed: 02.09.2020 15:47:51
Sample ID: KuIna103_8µL
Injectionvolume: 8

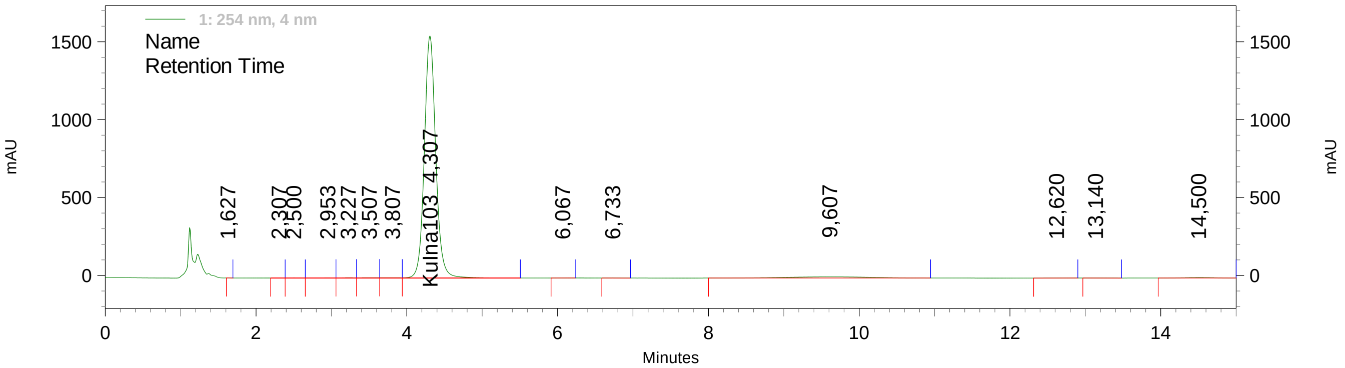

1: 254 nm. 4 nm

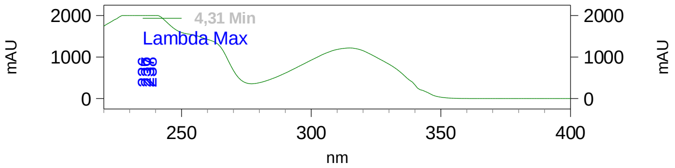

Pk #	*Name*	*Retention Time*	*Area Percent*	*Area*
1		1,627	0,007	4588
2		2,307	0,013	9001
3		2,500	0,053	36347
4		2,953	0,103	70628
5		3,227	0,135	92225
6		3,507	0,186	127375
7		3,807	0,190	129906
8	**KuIna103**	4,307	95,265	65260826
9		6,067	0,009	6475
10		6,733	0,044	30246
11		9,607	3,416	2340443
12		12,620	0,044	30256
13		13,140	0,037	25487
14		14,500	0,498	340894

Totals				
			100,000	68504697

TU Braunschweig Institut für Pharmazeutische Chemie

Method Name: C:\EZChrom Elite\Enterprise\Projects\Reinheit_Irina\Method\ACN-H2O\ACN-H2O_10-90_10min.met
Data: C:\EZChrom Elite\Enterprise\Projects\Reinheit_Irina\Data\KuIna103_8µL_02.09.2020 15-23-16_ACN-Puffer_35-65_15min.met
User: Irina Ihnatenko
Acquired: 02.09.2020 15:24:22
Printed: 02.09.2020 15:47:51
Sample ID: KuIna103_8µL
Injectionvolume: 8

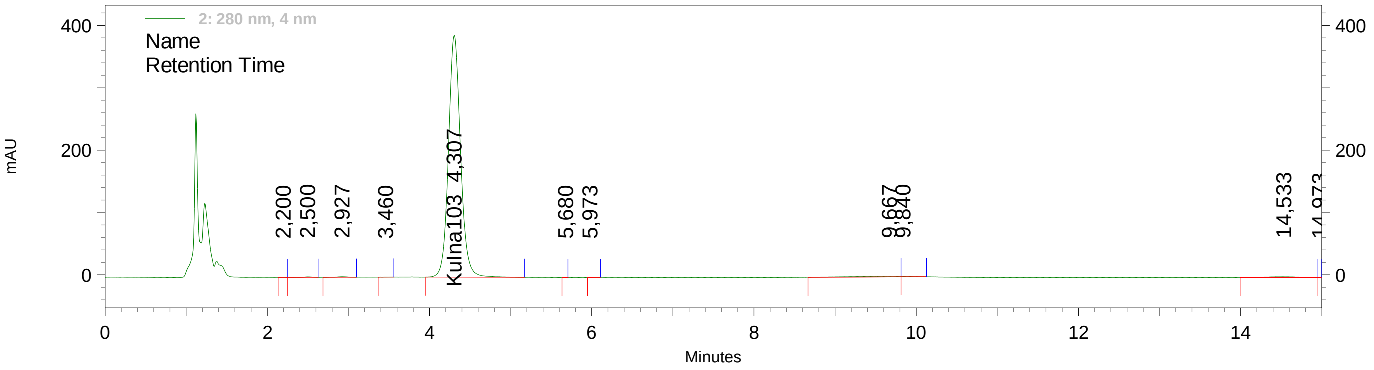

2: 280 nm, 4 nm
Results

Pk #	*Name*	*Retention Time*	*Area Percent*	*Area*
1		2,200	0,008	1295
2		2,500	0,116	19040
3		2,927	0,177	29031
4		3,460	0,014	2358
5	**KuIna103**	4,307	97,710	16062061
6		5,680	0,005	843
7		5,973	0,017	2747
8		9,667	0,998	164015
9		9,840	0,201	33020
10		14,533	0,751	123475
11		14,973	0,003	561
Totals			100,000	16438446

Spectrum Report

Spectra of all named detected peaks

(The peak spectrum is defined as the peak apex spectrum)

Multi-Chrom 1 (1: 254 nm, 4 nm) Spectra

Retention time: 4,307 Min
Peak name: KuIna103
Lambda max: 238, 237, 236
Lambda min: 392, 381, 373

Multi-Chrom 2 (2: 280 nm, 4 nm) Spectra

Method Name: **C:\EZChrom Elite\Enterprise\Projects\Reinheit_Irina\Method\ACN-H2O\ACN-H2O_10-90_10min.met**
Data: **C:\EZChrom Elite\Enterprise\Projects\Reinheit_Irina\Data\KuIna103_8µL_02.09.2020 15-23-16_ACN-Puffer_35-65_15min.met**
User: **Irina Ihnatenko**
Acquired: **02.09.2020 15:24:22**
Printed: **02.09.2020 15:47:51**
Sample ID: **KuIna103_8µL**
Injectionvolume: **8**

Retention time: 4,307 Min
Peak name: KuIna103
Lambda max: 238, 237, 236
Lambda min: 392, 381, 373

C:\EZChrom Elite\Enterprise\Projects\Reinheit_Irina\Data\KuIna103_8L_02.09.20[illegible]

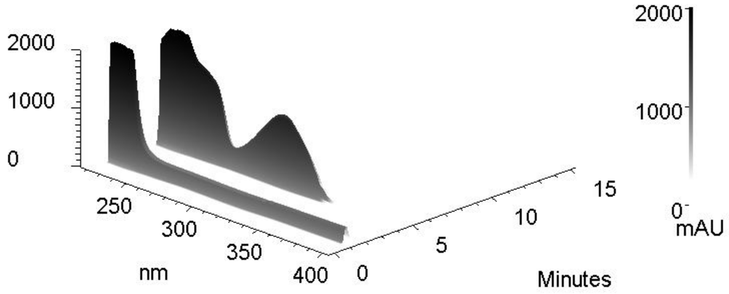

Supplement: S3 File — (ZIP) [file pone.0292946.s003.zip › S4_ZIP-File_HPLC_chromatograms/HPLC-Merck-cmpd-1h-iso-254+280nm.pdf]
