# Supplementary material for: The indole motif is essential for the antitrypanosomal activity of N5-substituted paullones
Source: PLoS One. 2023 Nov 30;18(11):e0292946. doi: 10.1371/journal.pone.0292946 (PMC10688702; doi:10.1371/journal.pone.0292946)

TU Braunschweig Institut für Pharmazeutische Chemie

Method Name: C:\EZChrom Elite\Enterprise\Projects\Reinheit_Irina\Method\ACN-H2O\ACN-H2O_90-10_1min_0,1µL.met
Data: C:\EZChrom Elite\Enterprise\Projects\Reinheit_Irina\Data\KuIna068 isokratisch_5µL_03.02.2020 19-13-05_ACN-Puffer_20-80_15min.met
User: Irina Ihnatenko
Acquired: 03.02.2020 19:14:08
Printed: 03.02.2020 19:34:29
Sample ID: KuIna068 isokratisch_5µL
Injectionvolume: 5

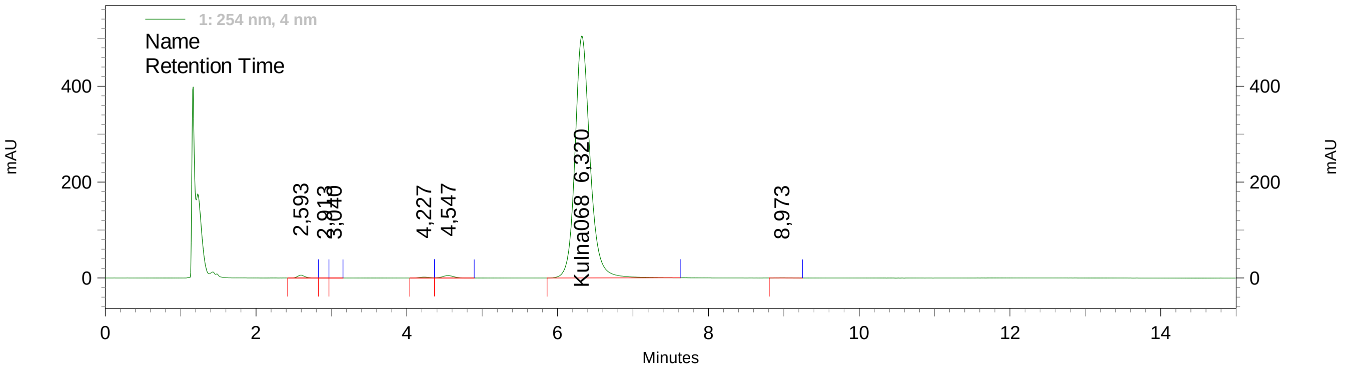

1: 254 nm. 4 nm

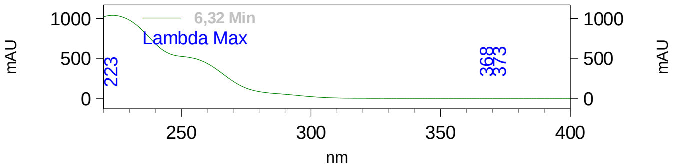

Pk #	*Name*	*Retention Time*	*Area Percent*	*Area*
1		2,593	0,554	157755
2		2,913	0,014	4089
3		3,040	0,016	4521
4		4,227	0,259	73838
5		4,547	0,764	217712
6	**KuIna068**	6,320	98,332	28019029
7		8,973	0,061	17493

Totals			100,000	28494437

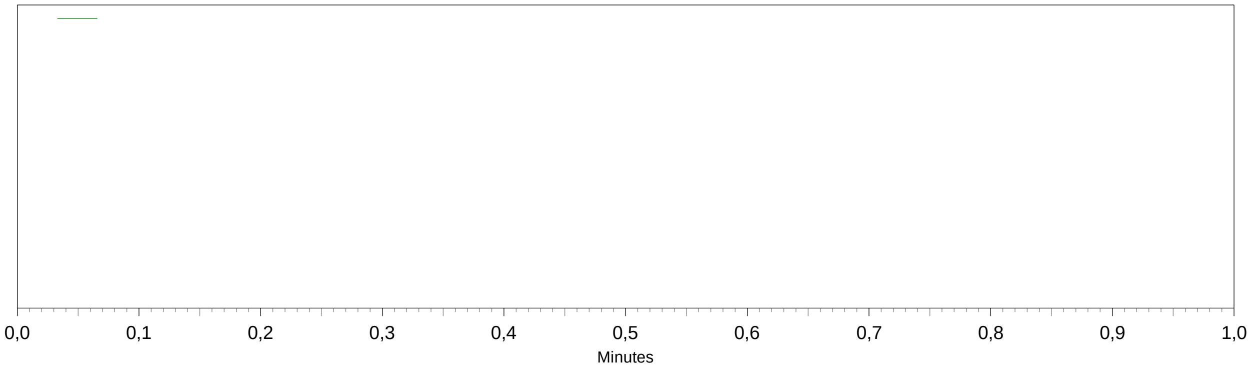

TU Braunschweig Institut für Pharmazeutische Chemie

Method Name: C:\EZChrom Elite\Enterprise\Projects\Reinheit_Irina\Method\ACN-H2O\ACN-H2O_90-10_1min_0,1µL.met
Data: C:\EZChrom Elite\Enterprise\Projects\Reinheit_Irina\Data\KuIna068 isokratisch_5µL_03.02.2020 19-13-05_ACN-Puffer_20-80_15min.met
User: Irina Ihnatenko
Acquired: 03.02.2020 19:14:08
Printed: 03.02.2020 19:34:29
Sample ID: KuIna068 isokratisch_5µL
Injectionvolume: 5

Pk #	*Name*	*Retention Time*	*Area Percent*	*Area*

Spectrum Report

Spectra of all named detected peaks

(The peak spectrum is defined as the peak apex spectrum)

Multi-Chrom 1 (1: 254 nm, 4 nm) Spectra

Retention time: 6,320 Min
Peak name: KuIna068
Lambda max: 223, 373, 368
Lambda min: 388, 347, 367

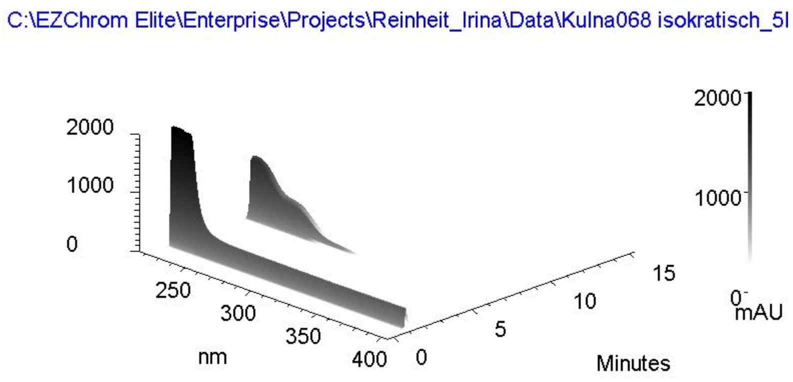

Supplement: S3 File — (ZIP) [file pone.0292946.s003.zip › S4_ZIP-File_HPLC_chromatograms/HPLC-Merck-cmpd-22-iso-254nm.pdf]
