# Supplementary material for: The indole motif is essential for the antitrypanosomal activity of N5-substituted paullones
Source: PLoS One. 2023 Nov 30;18(11):e0292946. doi: 10.1371/journal.pone.0292946 (PMC10688702; doi:10.1371/journal.pone.0292946)

TU Braunschweig Institut für Pharmazeutische Chemie

Method Name: C:\EZChrom Elite\Enterprise\Projects\Reinheit_Irina\Method\ACN-Puffer\ACN-Puffer_20-80_25min.met
Data: C:\EZChrom Elite\Enterprise\Projects\Reinheit_Irina\Data\2018-08-01\KuIna010_20µL_01.08.2018 18-06-29_ACN-Puffer_30-70_15min.met
User: Irina Ihnatenko
Acquired: 01.08.2018 18:07:51
Printed: 05.08.2018 17:31:23
Sample ID: KuIna010_20µL
Injectionvolume: 20

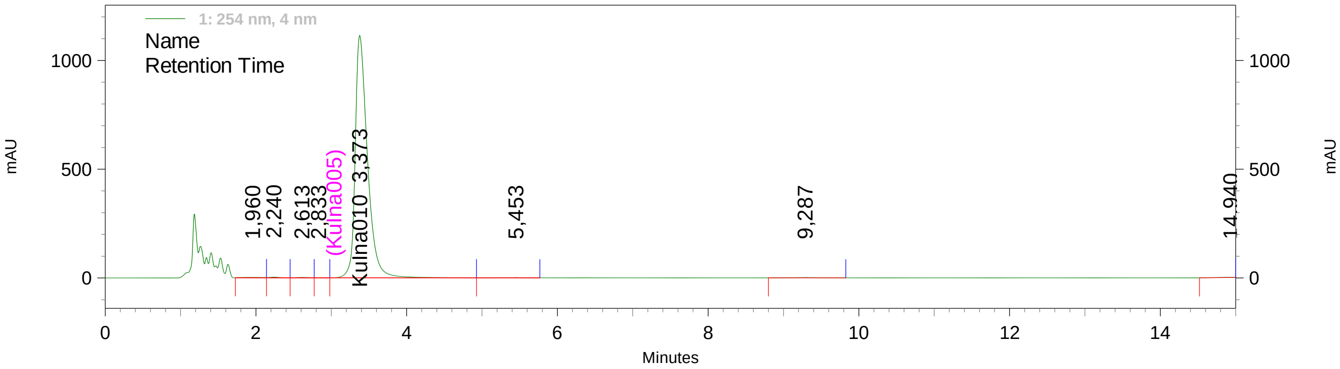

1: 254 nm, 4 nm

Results

Pk #	*Name*	*Retention Time*	*Area Percent*	*Area*
1		1,960	0,188	95916
2		2,240	0,158	80351
3		2,613	0,111	56333
4		2,833	0,026	13113
	**KuIna005**			
5	**KuIna010**	3,373	98,968	50449540
6		5,453	0,189	96303
7		9,287	0,296	150950
8		14,940	0,065	33096
Totals			100,000	50975602

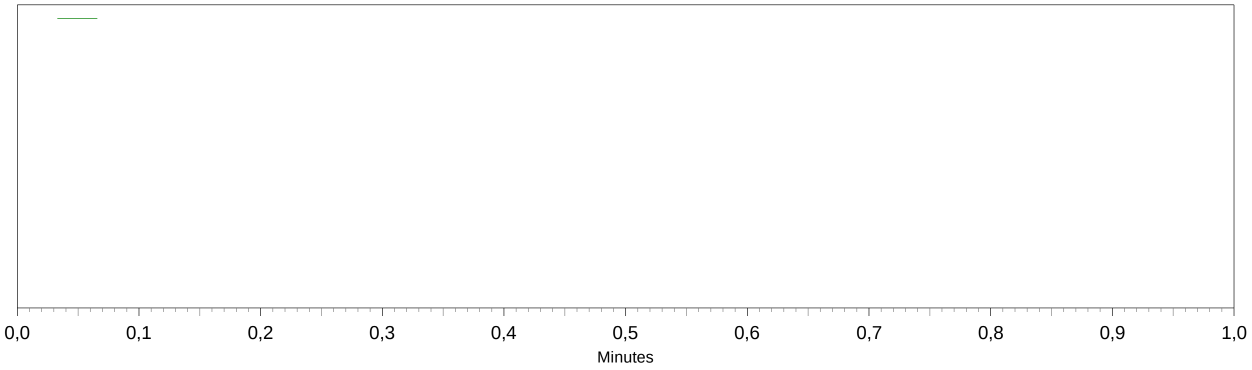

Pk #	*Name*	*Retention Time*	*Area Percent*	*Area*

Method Name: **C:\EZChrom Elite\Enterprise\Projects\Reinheit_Irina\Method\ACN-Puffer\ACN-Puffer_20-80_25min.met**
Data: **C:\EZChrom Elite\Enterprise\Projects\Reinheit_Irina\Data\2018-08-01\KuIna010_20µL_01.08.2018 18-06-29_ACN-Puffer_30-70_15min.met**
User: **Irina Ihnatenko**
Acquired: **01.08.2018 18:07:51**
Printed: **05.08.2018 17:31:23**
Sample ID: **KuIna010_20µL**
Injectionvolume: **20**

Spectrum Report

Spectra of all named detected peaks

(The peak spectrum is defined as the peak apex spectrum)

Multi-Chrom 1 (1: 254 nm, 4 nm) Spectra

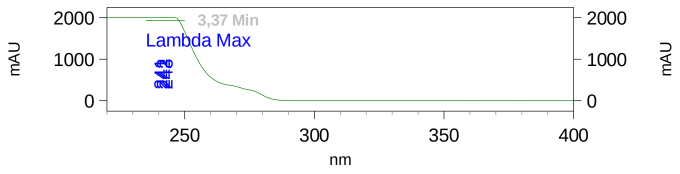

Retention time: 3,373 Min
Peak name: KuIna010
Lambda max: 243, 242, 241
Lambda min: 365, 359, 343

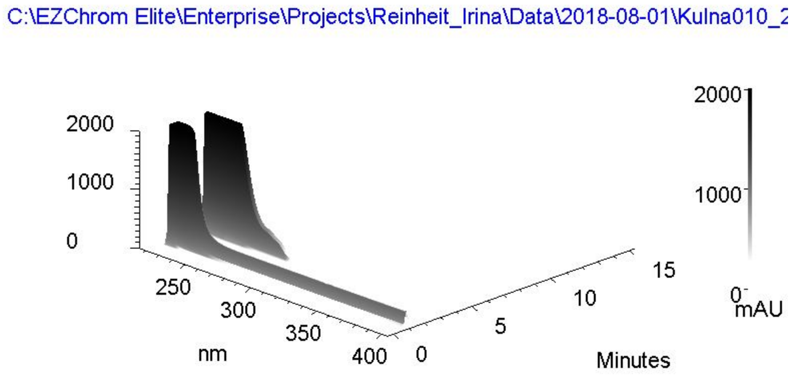

Supplement: S3 File — (ZIP) [file pone.0292946.s003.zip › S4_ZIP-File_HPLC_chromatograms/HPLC-Merck-cmpd-2b-iso-254nm.pdf]
