# Supplementary material for: The indole motif is essential for the antitrypanosomal activity of N5-substituted paullones
Source: PLoS One. 2023 Nov 30;18(11):e0292946. doi: 10.1371/journal.pone.0292946 (PMC10688702; doi:10.1371/journal.pone.0292946)

TU Braunschweig Institut für Pharmazeutische Chemie

Method Name: C:\EZChrom Elite\Enterprise\Projects\Reinheit_Irina\Method\ACN-Puffer\ACN-Puffer_20-80_25min.met
Data: C:\EZChrom Elite\Enterprise\Projects\Reinheit_Irina\Data\2018-08-01\KuIna012_10µL_01.08.2018 16-56-49_ACN-Puffer_40-60_15min.met
User: Irina Ihnatenko
Acquired: 01.08.2018 16:57:56
Printed: 05.08.2018 17:37:26
Sample ID: KuIna012_10µL
Injectionvolume: 10

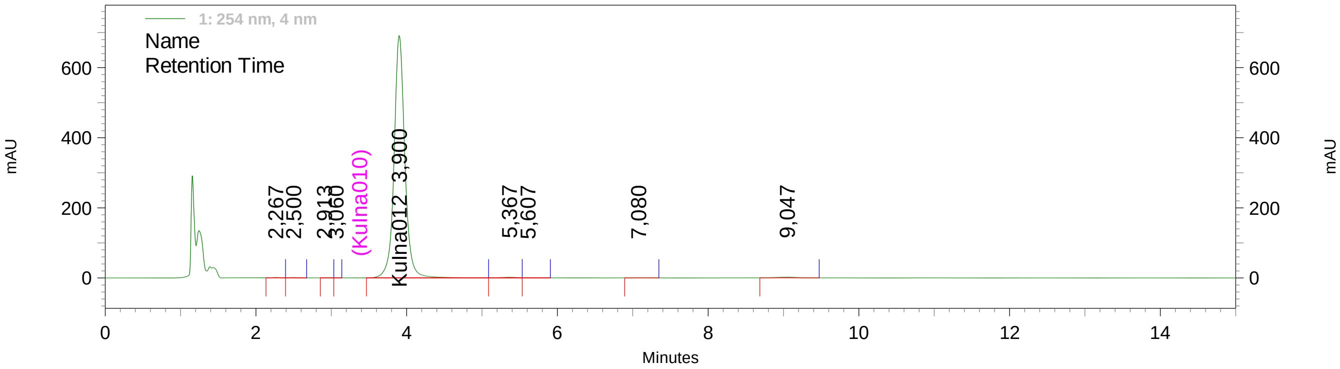

1: 254 nm, 4 nm

Results

Pk #	*Name*	*Retention Time*	*Area Percent*	*Area*
1		2,267	0,090	24901
2		2,500	0,081	22369
3		2,913	0,012	3418
4		3,060	0,003	726
	**KuIna010**			
5	**KuIna012**	3,900	98,698	27419612
6		5,367	0,359	99820
7		5,607	0,124	34549
8		7,080	0,092	25425
9		9,047	0,542	150581
Totals			100,000	27781401

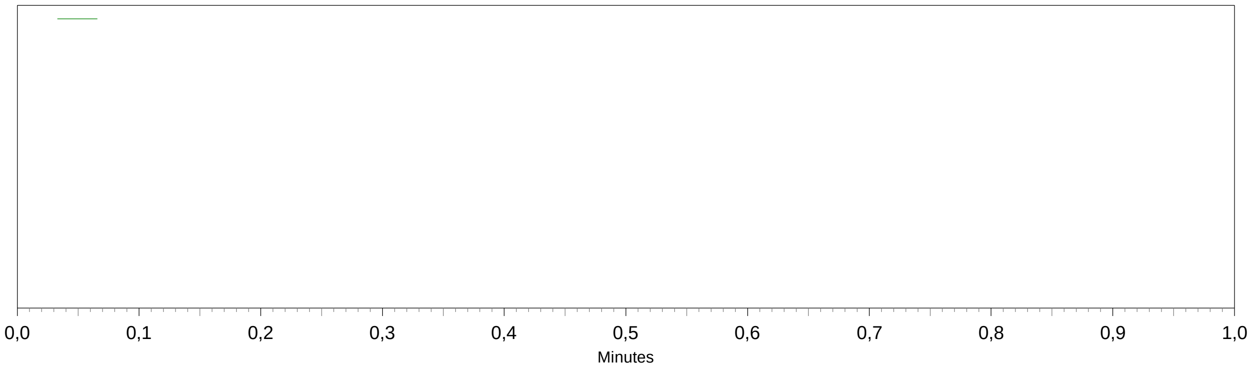

Pk #	*Name*	*Retention Time*	*Area Percent*	*Area*

Method Name: C:\EZChrom Elite\Enterprise\Projects\Reinheit_Irina\Method\ACN-Puffer\ACN-Puffer_20-80_25min.met
Data: C:\EZChrom Elite\Enterprise\Projects\Reinheit_Irina\Data\2018-08-01\KuIna012_10µL_01.08.2018 16-56-49_ACN-Puffer_40-60_15min.met
User: Irina Ihnatenko
Acquired: 01.08.2018 16:57:56
Printed: 05.08.2018 17:37:26
Sample ID: KuIna012_10µL
Injectionvolume: 10

Spectrum Report

Spectra of all named detected peaks

(The peak spectrum is defined as the peak apex spectrum)

Multi-Chrom 1 (1: 254 nm, 4 nm) Spectra

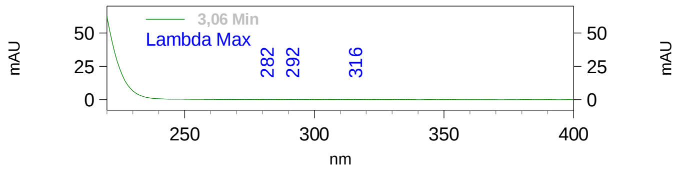

Retention time: 3,060 Min
Peak name:
Lambda max: 282, 292, 316
Lambda min: 341, 394, 372

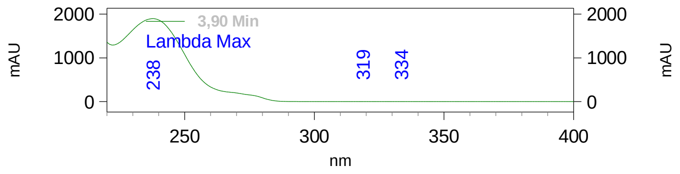

Retention time: 3,900 Min
Peak name: KuIna012
Lambda max: 238, 334, 319
Lambda min: 387, 366, 393

C:\EZChrom Elite\Enterprise\Projects\Reinheit_Irina\Data\2018-08-01\KuIna012_1

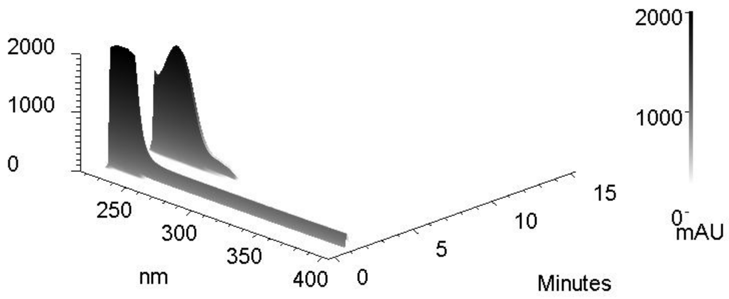

Supplement: S3 File — (ZIP) [file pone.0292946.s003.zip › S4_ZIP-File_HPLC_chromatograms/HPLC-Merck-cmpd-2c-iso-254nm.pdf]
