# Supplementary material for: The indole motif is essential for the antitrypanosomal activity of N5-substituted paullones
Source: PLoS One. 2023 Nov 30;18(11):e0292946. doi: 10.1371/journal.pone.0292946 (PMC10688702; doi:10.1371/journal.pone.0292946)

TU Braunschweig Institut für Pharmazeutische Chemie

Method Name: **C:\EZChrom Elite\Enterprise\Projects\Reinheit_Irina\Method\ACN-Puffer\ACN-Puffer_20-80_25min.met**
Data: **C:\EZChrom Elite\Enterprise\Projects\Reinheit_Irina\Data\2018-08-01\KuIna013_10µL_01.08.2018 17-50-07_ACN-Puffer_30-70_15min.met**
User: **Irina Ihnatenko**
Acquired: **01.08.2018 17:51:17**
Printed: **05.08.2018 17:50:18**
Sample ID: **KuIna013_10µL**
Injectionvolume: **10**

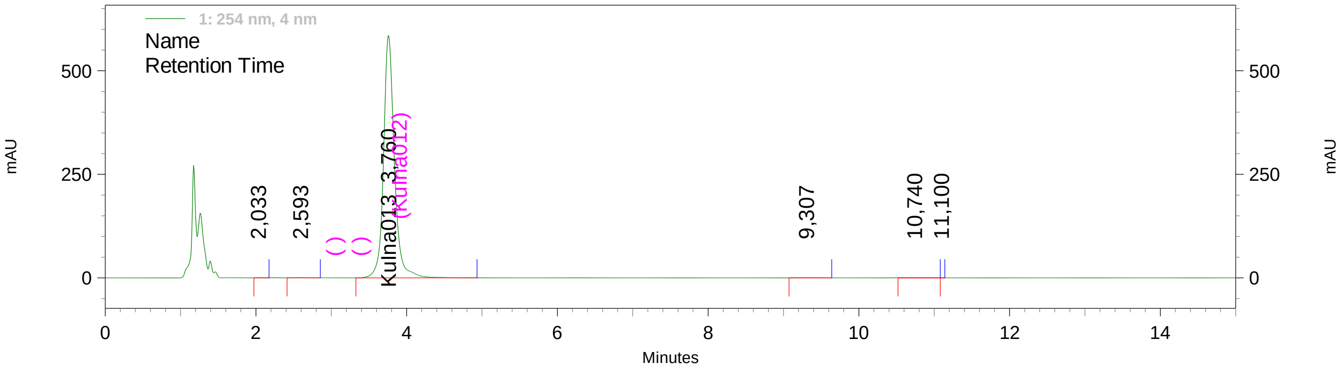

1: 254 nm, 4 nm

Results

Pk #	*Name*	*Retention Time*	*Area Percent*	*Area*
1		2,033	0,022	5215
2		2,593	0,095	22210
3	**KuIna013** **KuIna012**	3,760	99,598	23367864
4		9,307	0,155	36252
5		10,740	0,127	29896
6		11,100	0,003	645
Totals			100,000	23462082

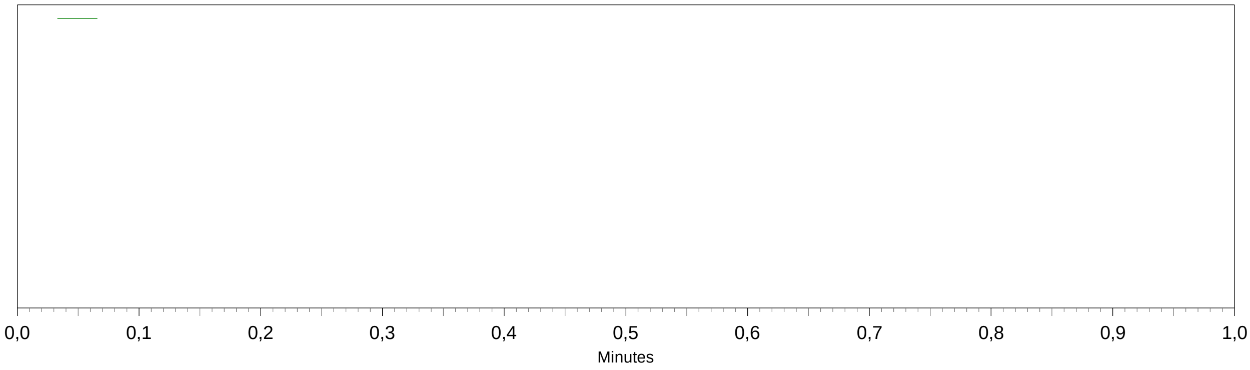

Pk #	*Name*	*Retention Time*	*Area Percent*	*Area*

Method Name: C:\EZChrom Elite\Enterprise\Projects\Reinheit_Irina\Method\ACN-Puffer\ACN-Puffer_20-80_25min.met
Data: C:\EZChrom Elite\Enterprise\Projects\Reinheit_Irina\Data\2018-08-01\KuIna013_10µL_01.08.2018 17-50-07_ACN-Puffer_30-70_15min.met
User: Irina Ihnatenko
Acquired: 01.08.2018 17:51:17
Printed: 05.08.2018 17:50:18
Sample ID: KuIna013_10µL
Injectionvolume: 10

Spectrum Report

Spectra of all named detected peaks

(The peak spectrum is defined as the peak apex spectrum)

Multi-Chrom 1 (1: 254 nm, 4 nm) Spectra

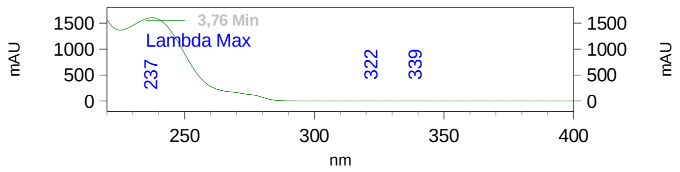

Retention time: 3,760 Min
Peak name: KuIna013
Lambda max: 237, 322, 339
Lambda min: 373, 395, 366

C:\EZChrom Elite\Enterprise\Projects\Reinheit_Irina\Data\2018-08-01\KuIna013_1

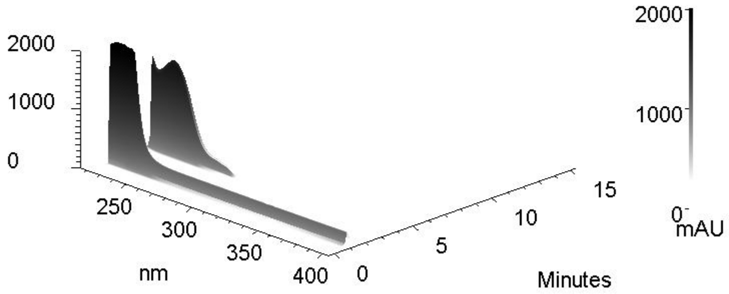

Supplement: S3 File — (ZIP) [file pone.0292946.s003.zip › S4_ZIP-File_HPLC_chromatograms/HPLC-Merck-cmpd-2h-iso-254nm.pdf]
