# Supplementary material for: The indole motif is essential for the antitrypanosomal activity of N5-substituted paullones
Source: PLoS One. 2023 Nov 30;18(11):e0292946. doi: 10.1371/journal.pone.0292946 (PMC10688702; doi:10.1371/journal.pone.0292946)

TU Braunschweig Institut für Pharmazeutische Chemie

Method Name: **C:\EZChrom Elite\Enterprise\Projects\Reinheit_Irina\Method\ACN-Puffer\ACN-Puffer_20-80_25min.met**
Data: **C:\EZChrom Elite\Enterprise\Projects\Reinheit_Irina\Data\2018-08-01\KuIna014_5µL_01.08.2018 20-10-24_ACN-Puffer_40-60_15min.met**
User: **Irina Ihnatenko**
Acquired: **01.08.2018 20:11:26**
Printed: **05.08.2018 17:55:41**
Sample ID: **KuIna014_5µL**
Injectionvolume: **5**

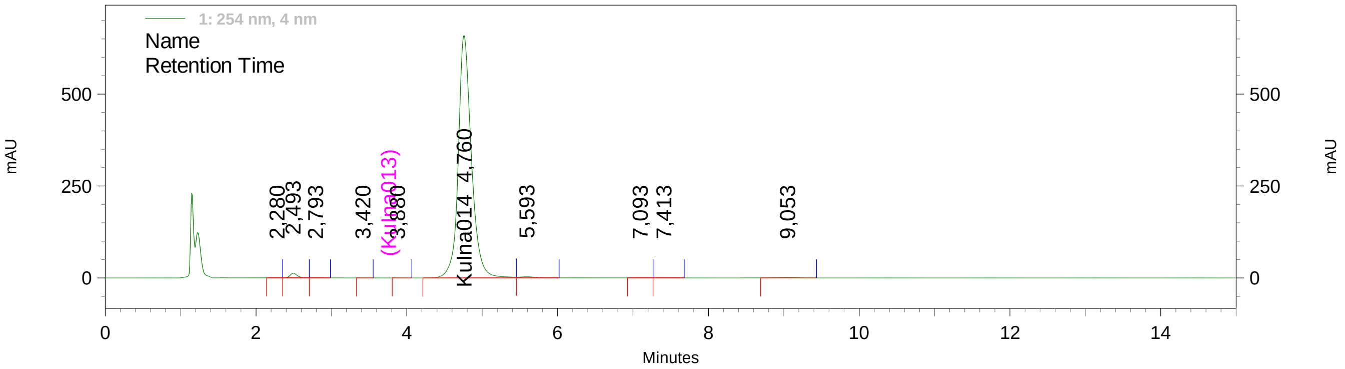

1: 254 nm, 4 nm

Results

Pk #	*Name*	*Retention Time*	*Area Percent*	*Area*
1		2,280	0,048	15642
2		2,493	1,062	346209
3		2,793	0,059	19189
4		3,420	0,016	5063
	**KuIna013**			
5		3,880	0,012	3892
6	**KuIna014**	4,760	97,898	31906291
7		5,593	0,532	173541
8		7,093	0,054	17463
9		7,413	0,049	15998
10		9,053	0,270	88129
Totals			100,000	32591417

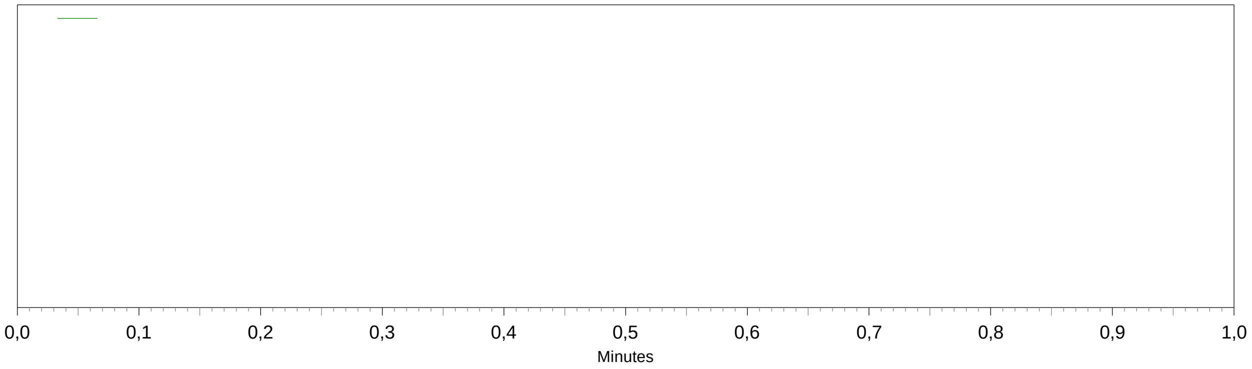

Method Name: C:\EZChrom Elite\Enterprise\Projects\Reinheit_Irina\Method\ACN-Puffer\ACN-Puffer_20-80_25min.met
Data: C:\EZChrom Elite\Enterprise\Projects\Reinheit_Irina\Data\2018-08-01\KuIna014_5µL_01.08.2018 20-10-24_ACN-Puffer_40-60_15min.met
User: Irina Ihnatenko
Acquired: 01.08.2018 20:11:26
Printed: 05.08.2018 17:55:41
Sample ID: KuIna014_5µL
Injectionvolume: 5

Pk #	*Name*	*Retention Time*	*Area Percent*	*Area*

Spectrum Report

Spectra of all named detected peaks

(The peak spectrum is defined as the peak apex spectrum)

Multi-Chrom 1 (1: 254 nm, 4 nm) Spectra

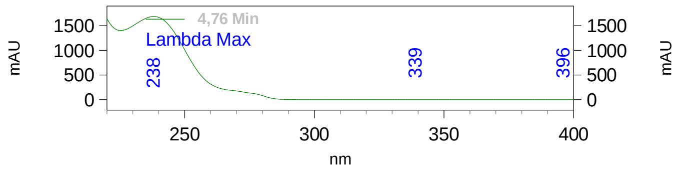

Retention time: 4,760 Min
Peak name: KuIna014
Lambda max: 238, 339, 396
Lambda min: 377, 348, 364

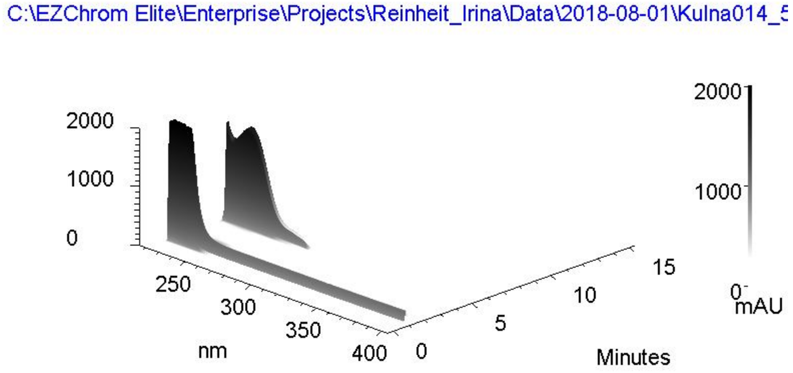

Supplement: S3 File — (ZIP) [file pone.0292946.s003.zip › S4_ZIP-File_HPLC_chromatograms/HPLC-Merck-cmpd-2i-iso-254nm.pdf]
