# Supplementary material for: The indole motif is essential for the antitrypanosomal activity of N5-substituted paullones
Source: PLoS One. 2023 Nov 30;18(11):e0292946. doi: 10.1371/journal.pone.0292946 (PMC10688702; doi:10.1371/journal.pone.0292946)

TU Braunschweig Institut für Pharmazeutische Chemie

Method Name: C:\EZChrom Elite\Enterprise\Projects\Reinheit_Irina\Method\ACN-Puffer\ACN-Puffer_20-80_25min.met
Data: C:\EZChrom Elite\Enterprise\Projects\Reinheit_Irina\Data\2018-08-02\KuIna015_20µL_02.08.2018 17-58-40_ACN-Puffer_10-90_15min.met
User: Irina Ihnatenko
Acquired: 02.08.2018 18:00:00
Printed: 05.08.2018 18:08:19
Sample ID: KuIna015_20µL
Injectionvolume: 20

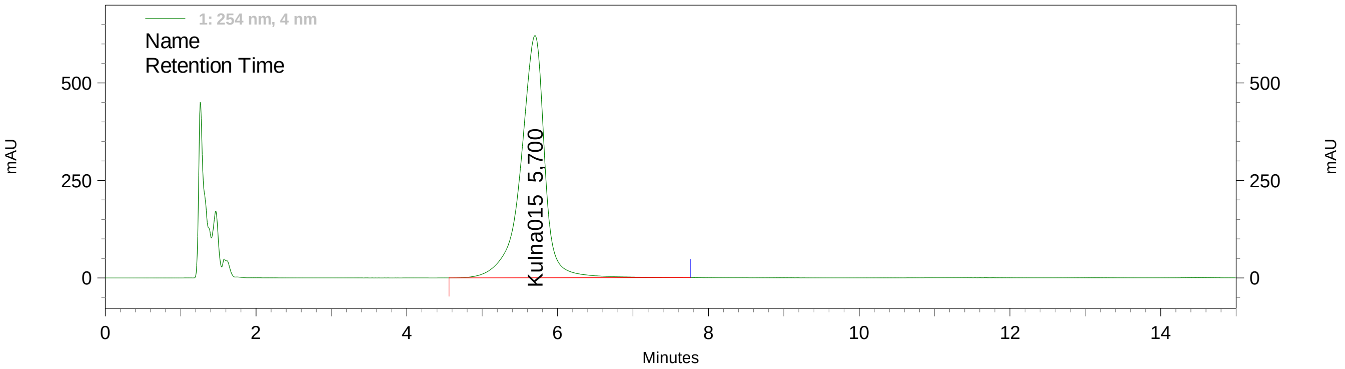

1: 254 nm, 4 nm
Results

Pk #	*Name*	*Retention Time*	*Area Percent*	*Area*
1	**KuIna015**	5,700	100,000	54825294
Totals			100,000	54825294

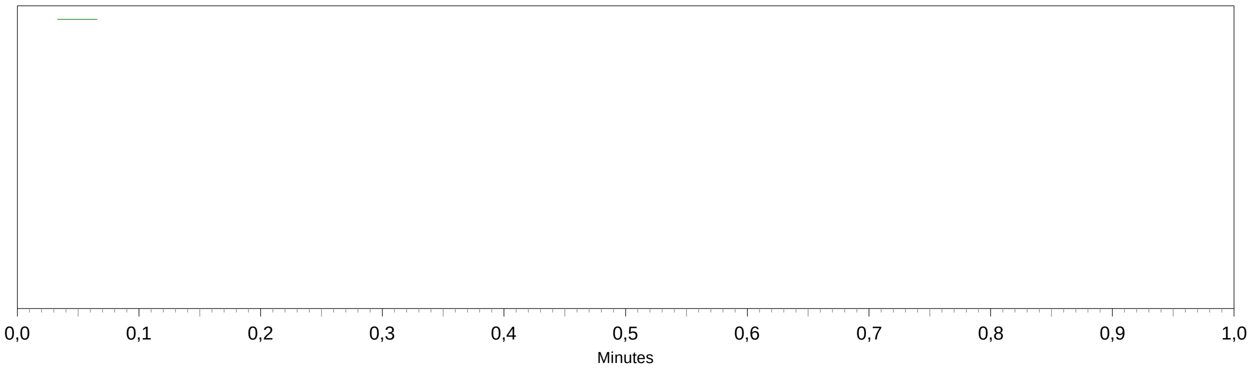

Pk #	*Name*	*Retention Time*	*Area Percent*	*Area*

Spectrum Report
Spectra of all named detected peaks

(The peak spectrum is defined as the peak apex spectrum)

Multi-Chrom 1 (1: 254 nm, 4 nm) Spectra

TU Braunschweig Institut für Pharmazeutische Chemie

Method Name: C:\EZChrom Elite\Enterprise\Projects\Reinheit_Irina\Method\ACN-Puffer\ACN-Puffer_20-80_25min.met
Data: C:\EZChrom Elite\Enterprise\Projects\Reinheit_Irina\Data\2018-08-02\KuIna015_20µL_02.08.2018 17-58-40_ACN-Puffer_10-90_15min.met
User: Irina Ihnatenko
Acquired: 02.08.2018 18:00:00
Printed: 05.08.2018 18:08:19
Sample ID: KuIna015_20µL
Injectionvolume: 20

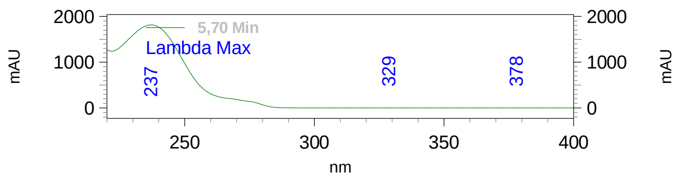

Retention time: 5,700 Min
Peak name: KuIna015
Lambda max: 237, 329, 378
Lambda min: 386, 368, 345

C:\EZChrom Elite\Enterprise\Projects\Reinheit_Irina\Data\2018-08-02\KuIna015_2

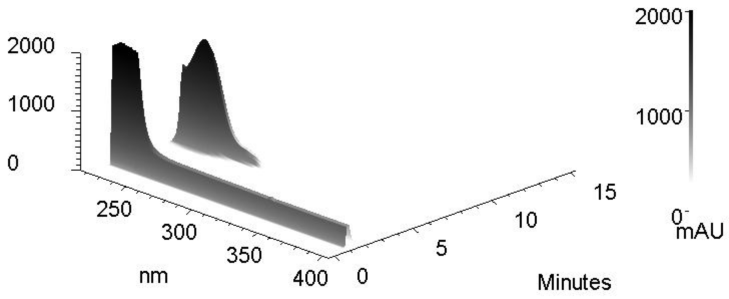

Supplement: S3 File — (ZIP) [file pone.0292946.s003.zip › S4_ZIP-File_HPLC_chromatograms/HPLC-Merck-cmpd-2o-iso-254nm.pdf]
