# Supplementary material for: The indole motif is essential for the antitrypanosomal activity of N5-substituted paullones
Source: PLoS One. 2023 Nov 30;18(11):e0292946. doi: 10.1371/journal.pone.0292946 (PMC10688702; doi:10.1371/journal.pone.0292946)

TU Braunschweig Institut für Pharmazeutische Chemie

Method Name: C:\EZChrom Elite\Enterprise\Projects\Reinheit_Irina\Method\ACN-Puffer\ACN-Puffer_20-80_25min.met
Data: C:\EZChrom Elite\Enterprise\Projects\Reinheit_Irina\Data\2018-08-02\KuIna016_20µL_02.08.2018 18-31-38_ACN-Puffer_10-90_15min.met
User: Irina Ihnatenko
Acquired: 02.08.2018 18:32:57
Printed: 05.08.2018 18:14:07
Sample ID: KuIna016_20µL
Injectionvolume: 20

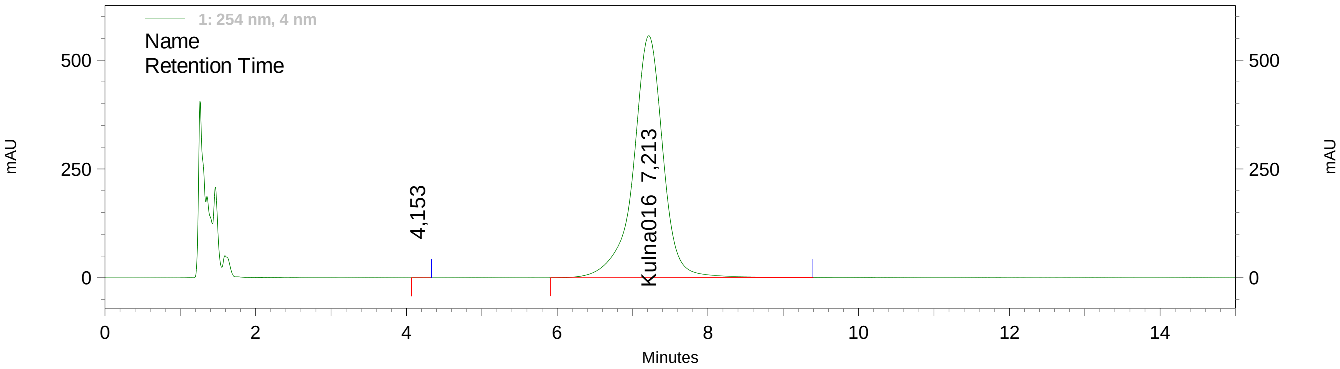

1: 254 nm, 4 nm

Results

Pk #	*Name*	*Retention Time*	*Area Percent*	*Area*
1		4,153	0,008	4753
2	**KuIna016**	7,213	99,992	60946351

Totals				
			100,000	60951104

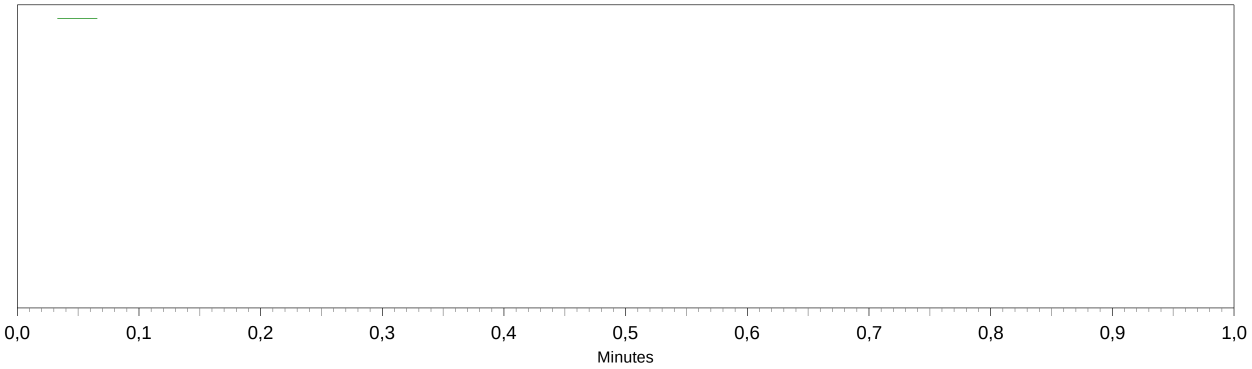

Pk #	*Name*	*Retention Time*	*Area Percent*	*Area*

Spectrum Report

Spectra of all named detected peaks

(The peak spectrum is defined as the peak apex spectrum)

TU Braunschweig Institut für Pharmazeutische Chemie

Method Name: C:\EZChrom Elite\Enterprise\Projects\Reinheit_Irina\Method\ACN-Puffer\ACN-Puffer_20-80_25min.met
Data: C:\EZChrom Elite\Enterprise\Projects\Reinheit_Irina\Data\2018-08-02\KuIna016_20µL_02.08.2018 18-31-38_ACN-Puffer_10-90_15min.met
User: Irina Ihnatenko
Acquired: 02.08.2018 18:32:57
Printed: 05.08.2018 18:14:07
Sample ID: KuIna016_20µL
Injectionvolume: 20

Multi-Chrom 1 (1: 254 nm, 4 nm) Spectra

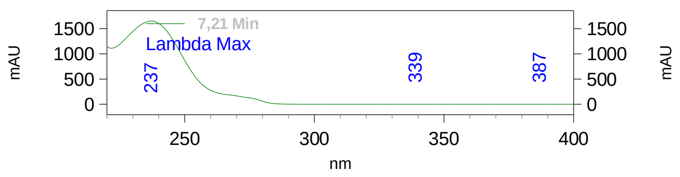

Retention time: 7,213 Min
Peak name: KuIna016
Lambda max: 237, 339, 387
Lambda min: 377, 344, 368

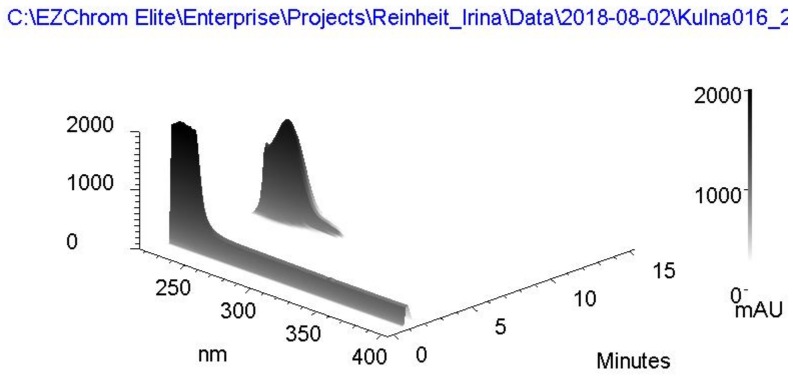

Supplement: S3 File — (ZIP) [file pone.0292946.s003.zip › S4_ZIP-File_HPLC_chromatograms/HPLC-Merck-cmpd-2p-iso-254nm.pdf]
