# Supplementary material for: The indole motif is essential for the antitrypanosomal activity of N5-substituted paullones
Source: PLoS One. 2023 Nov 30;18(11):e0292946. doi: 10.1371/journal.pone.0292946 (PMC10688702; doi:10.1371/journal.pone.0292946)

TU Braunschweig Institut für Pharmazeutische Chemie

Method Name: C:\EZChrom Elite\Enterprise\Projects\Reinheit_Irina\Method\ACN-Puffer\ACN-Puffer_05-95_15min.met
Data: C:\EZChrom Elite\Enterprise\Projects\Reinheit_Irina\Data\KuIna039_10µL_ACN-Puffer_10-90_15min.met07.05.2019 12-38-50.dat
User: Sandra Schweda
Acquired: 07.05.2019 12:40:01
Printed: 17.05.2019 16:47:05
Sample ID: KuIna039_10µL_
Injectionvolume: 10

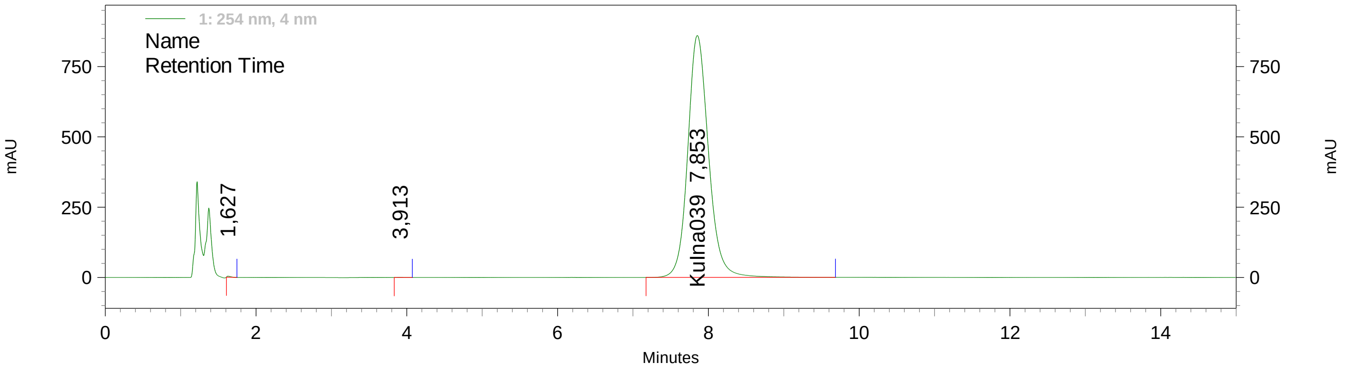

1: 254 nm, 4 nm
Results

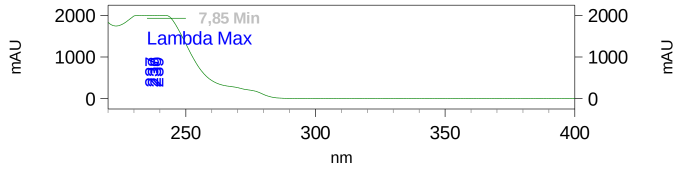

Pk #	*Name*	*Retention Time*	*Area Percent*	*Area*
1		1,627	0,075	49502
2		3,913	0,007	4856
3	**KuIna039**	7,853	99,918	65993088
Totals			100,000	66047446

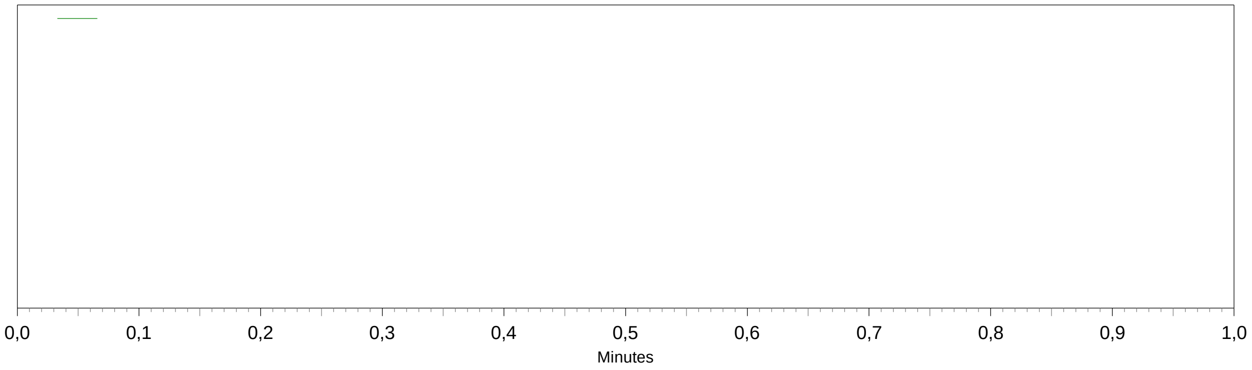

Pk #	*Name*	*Retention Time*	*Area Percent*	*Area*

Spectrum Report

Method Name: **C:\EZChrom Elite\Enterprise\Projects\Reinheit_Irina\Method\ACN-Puffer\ACN-Puffer_05-95_15min.met**
Data: **C:\EZChrom Elite\Enterprise\Projects\Reinheit_Irina\Data\KuIna039_10µL_ACN-Puffer_10-90_15min.met07.05.2019 12-38-50.dat**
User: **Sandra Schweda**
Acquired: **07.05.2019 12:40:01**
Printed: **17.05.2019 16:47:05**
Sample ID: **KuIna039_10µL_**
Injectionvolume: **10**

Spectra of all named detected peaks

(The peak spectrum is defined as the peak apex spectrum)

Multi-Chrom 1 (1: 254 nm, 4 nm) Spectra

Retention time: 7,853 Min
Peak name: KuIna039
Lambda max: 239, 238, 237
Lambda min: 396, 341, 318

C:\EZChrom Elite\Enterprise\Projects\Reinheit_Irina\Data\KuIna039_10L_ACN-Pu

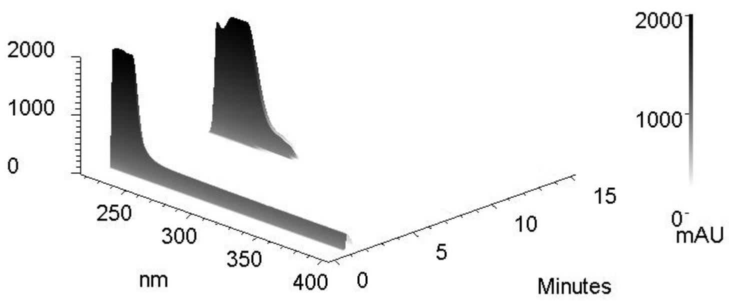

Supplement: S3 File — (ZIP) [file pone.0292946.s003.zip › S4_ZIP-File_HPLC_chromatograms/HPLC-Merck-cmpd-2q-iso-254nm.pdf]
