# Supplementary material for: The indole motif is essential for the antitrypanosomal activity of N5-substituted paullones
Source: PLoS One. 2023 Nov 30;18(11):e0292946. doi: 10.1371/journal.pone.0292946 (PMC10688702; doi:10.1371/journal.pone.0292946)

TU Braunschweig Institut für Pharmazeutische Chemie

Method Name: C:\EZChrom Elite\Enterprise\Projects\Reinheit_Irina\Method\ACN-H2O\ACN-H2O_90-10_15min.met
Data: C:\EZChrom Elite\Enterprise\Projects\Reinheit_Irina\Data\2019-02-01\KuIna032_01.02.2019 12-17-05_ACN-Puffer_10-90_15min.met
User: Irina Ihnatenko
Acquired: 01.02.2019 12:18:27
Printed: 07.02.2019 16:56:23
Sample ID: KuIna032
Injectionvolume: 20

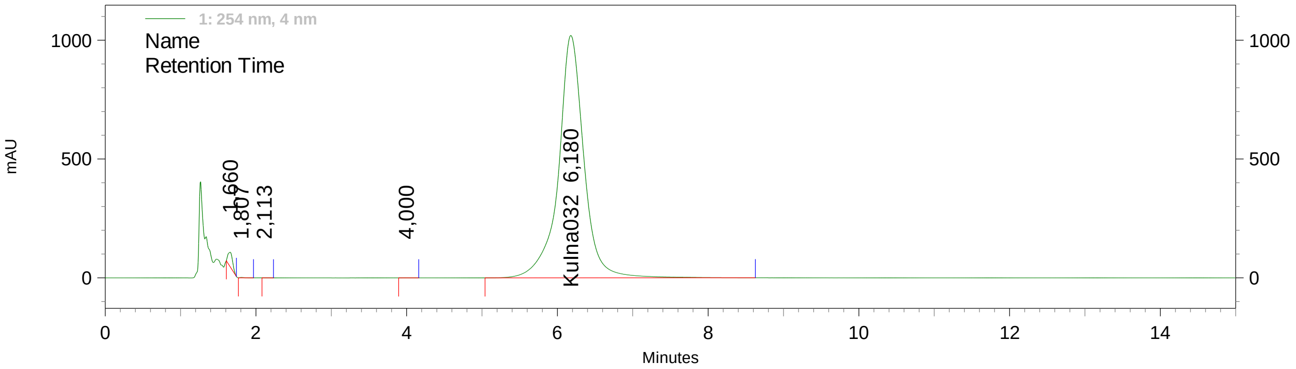

1: 254 nm, 4 nm
Results

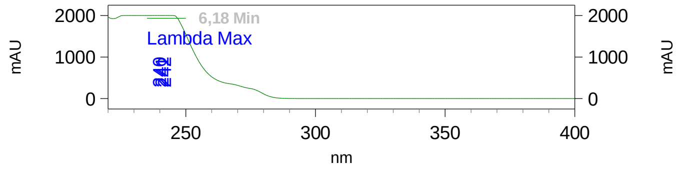

Pk #	*Name*	*Retention Time*	*Area Percent*	*Area*
1		1,660	1,076	1041905
2		1,807	0,023	22716
3		2,113	0,002	2016
4		4,000	0,005	4666
5	**KuIna032**	6,180	98,894	95803612
Totals			100,000	96874915

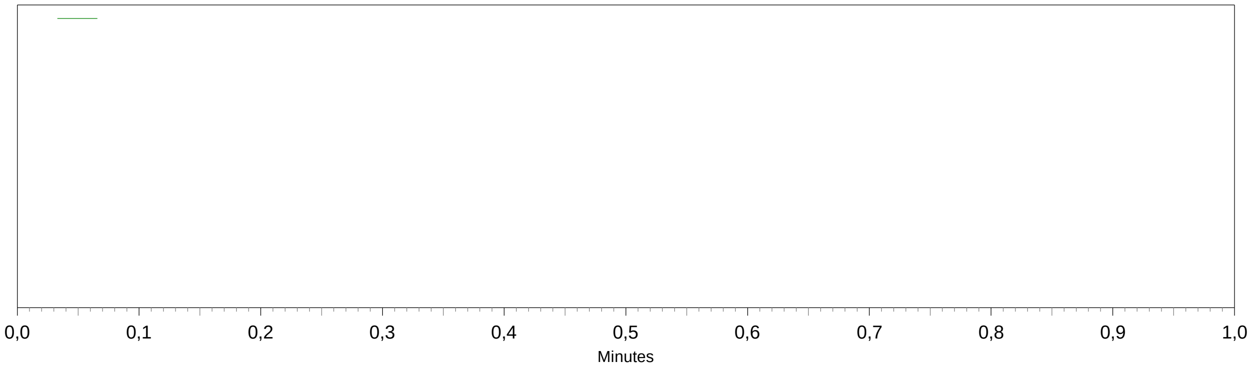

Pk #	*Name*	*Retention Time*	*Area Percent*	*Area*

Method Name: **C:\EZChrom Elite\Enterprise\Projects\Reinheit_Irina\Method\ACN-H2O\ACN-H2O_90-10_15min.met**
Data: **C:\EZChrom Elite\Enterprise\Projects\Reinheit_Irina\Data\2019-02-01\KuIna032_01.02.2019 12-17-05_ACN-Puffer_10-90_15min.met**
User: **Irina Ihnatenko**
Acquired: **01.02.2019 12:18:27**
Printed: **07.02.2019 16:56:23**
Sample ID: **KuIna032**
Injectionvolume: **20**

Spectrum Report

Spectra of all named detected peaks

(The peak spectrum is defined as the peak apex spectrum)

Multi-Chrom 1 (1: 254 nm, 4 nm) Spectra

Retention time: 6,180 Min
Peak name: KuIna032
Lambda max: 242, 241, 240
Lambda min: 365, 382, 343

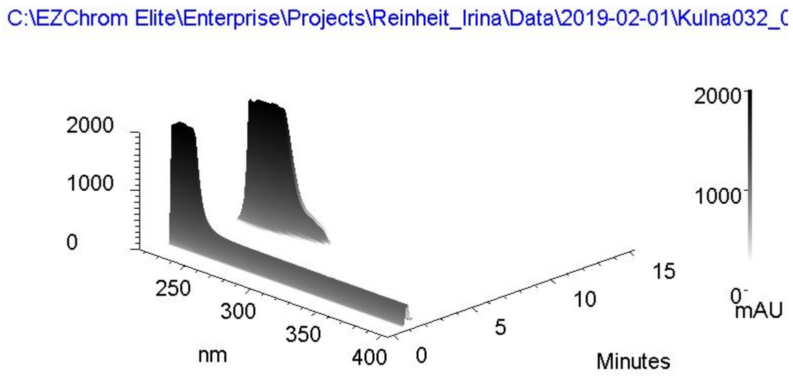

Supplement: S3 File — (ZIP) [file pone.0292946.s003.zip › S4_ZIP-File_HPLC_chromatograms/HPLC-Merck-cmpd-2r-iso-254nm.pdf]
