# Supplementary material for: The indole motif is essential for the antitrypanosomal activity of N5-substituted paullones
Source: PLoS One. 2023 Nov 30;18(11):e0292946. doi: 10.1371/journal.pone.0292946 (PMC10688702; doi:10.1371/journal.pone.0292946)

TU Braunschweig Institut für Pharmazeutische Chemie

Method Name: C:\EZChrom Elite\Enterprise\Projects\Reinheit_Irina\Method\ACN-Puffer\ACN-Puffer_05-95_15min.met
Data: C:\EZChrom Elite\Enterprise\Projects\Reinheit Sandra\Data\KuIna036_20µL_ACN-Puffer_10-90_15min.met16.04.2019 10-44-32.dat
User: Sandra Schweda
Acquired: 16.04.2019 10:45:51
Printed: 16.04.2019 11:13:06
Sample ID: KuIna036_20µL_
Injectionvolume: 20

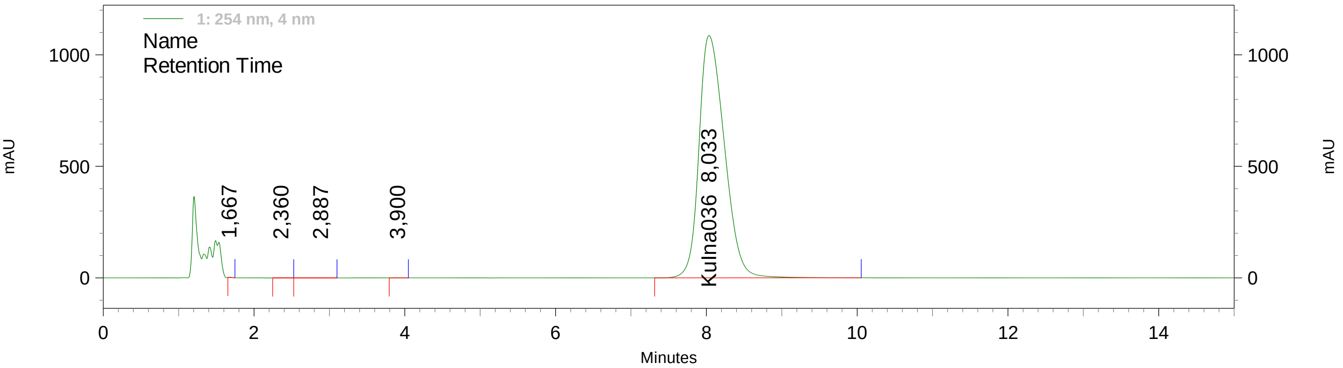

1: 254 nm. 4 nm

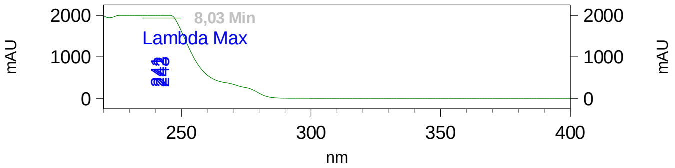

Pk #	*Name*	*Retention Time*	*Area Percent*	*Area*
1		1,667	0,012	12146
2		2,360	0,012	11727
3		2,887	0,019	19436
4		3,900	0,007	7478
5	**KuIna036**	8,033	99,950	101880252

Totals			100,000	101931039

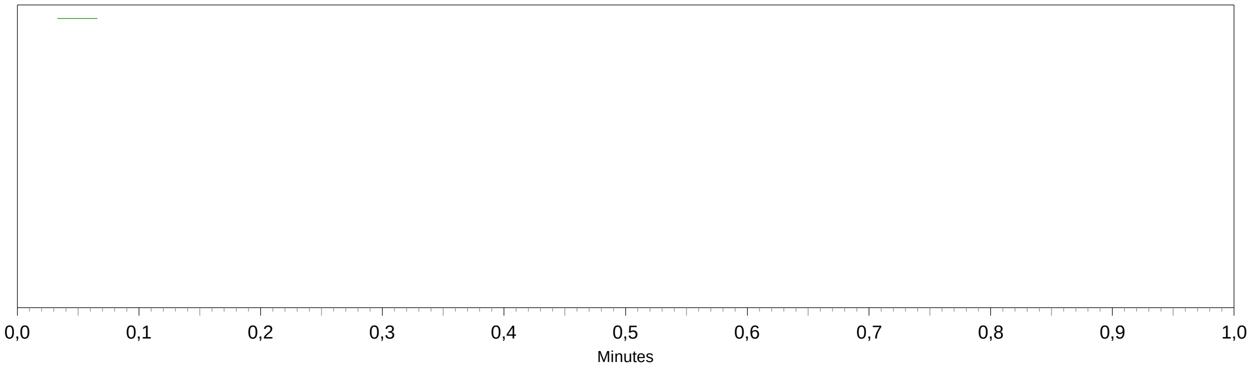

Pk #	*Name*	*Retention Time*	*Area Percent*	*Area*

Method Name: **C:\EZChrom Elite\Enterprise\Projects\Reinheit_Irina\Method\ACN-Puffer\ACN-Puffer_05-95_15min.met**
Data: **C:\EZChrom Elite\Enterprise\Projects\Reinheit Sandra\Data\KuIna036_20µL_ACN-Puffer_10-90_15min.met16.04.2019 10-44-32.dat**
User: **Sandra Schweda**
Acquired: **16.04.2019 10:45:51**
Printed: **16.04.2019 11:13:06**
Sample ID: **KuIna036_20µL_**
Injectionvolume: **20**

Spectrum Report

Spectra of all named detected peaks

(The peak spectrum is defined as the peak apex spectrum)

Multi-Chrom 1 (1: 254 nm, 4 nm) Spectra

Retention time: 8,033 Min
Peak name: KuIna036
Lambda max: 243, 242, 241
Lambda min: 394, 347, 330

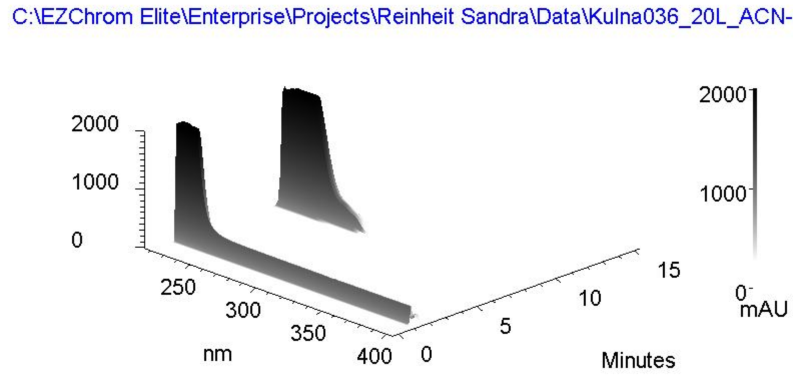

Supplement: S3 File — (ZIP) [file pone.0292946.s003.zip › S4_ZIP-File_HPLC_chromatograms/HPLC-Merck-cmpd-2s-iso-254nm.pdf]
