# Supplementary material for: The indole motif is essential for the antitrypanosomal activity of N5-substituted paullones
Source: PLoS One. 2023 Nov 30;18(11):e0292946. doi: 10.1371/journal.pone.0292946 (PMC10688702; doi:10.1371/journal.pone.0292946)

TU Braunschweig Institut für Pharmazeutische Chemie

Method Name: **C:\EZChrom Elite\Enterprise\Projects\Reinheit_Irina\Method\ACN-H2O\ACN-H2O_90-10_15min.met**
Data: **C:\EZChrom Elite\Enterprise\Projects\Reinheit_Irina\Data\2019-02-01\KuIna028_01.02.2019 13-22-26_ACN-Puffer_15-85_15min.met**
User: **Irina Ihnatenko**
Acquired: **01.02.2019 13:23:44**
Printed: **07.02.2019 16:42:52**
Sample ID: **KuIna028**
Injectionvolume: **20**

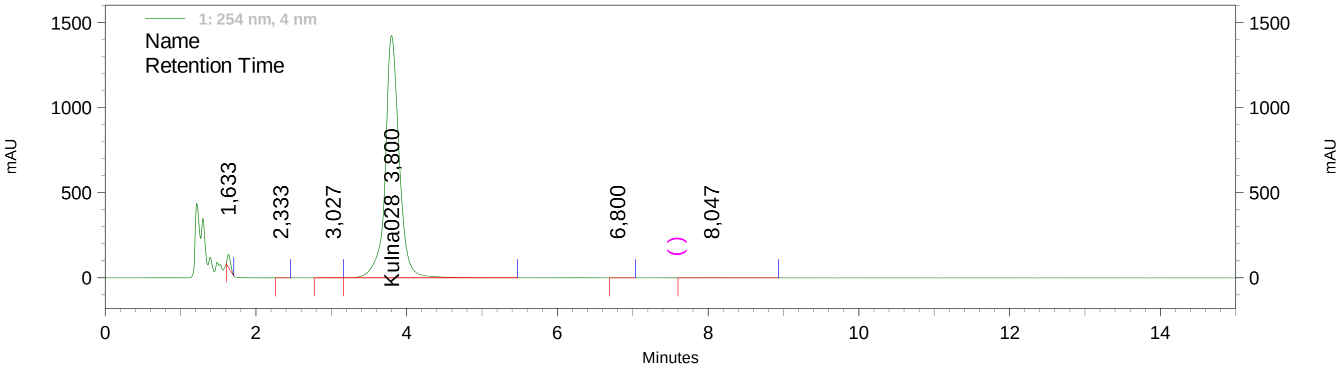

1: 254 nm, 4 nm
Results

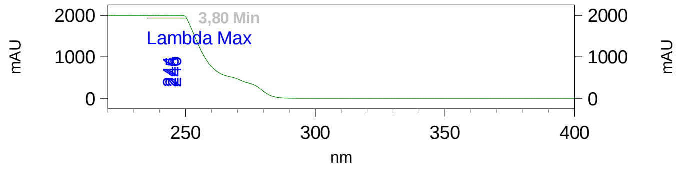

Pk #	*Name*	*Retention Time*	*Area Percent*	*Area*
1		1,633	1,236	921558
2		2,333	0,005	3658
3		3,027	0,088	65906
4	**KuIna028**	3,800	98,459	73436742
5		6,800	0,011	8106
6		8,047	0,202	150370
Totals			100,000	74586340

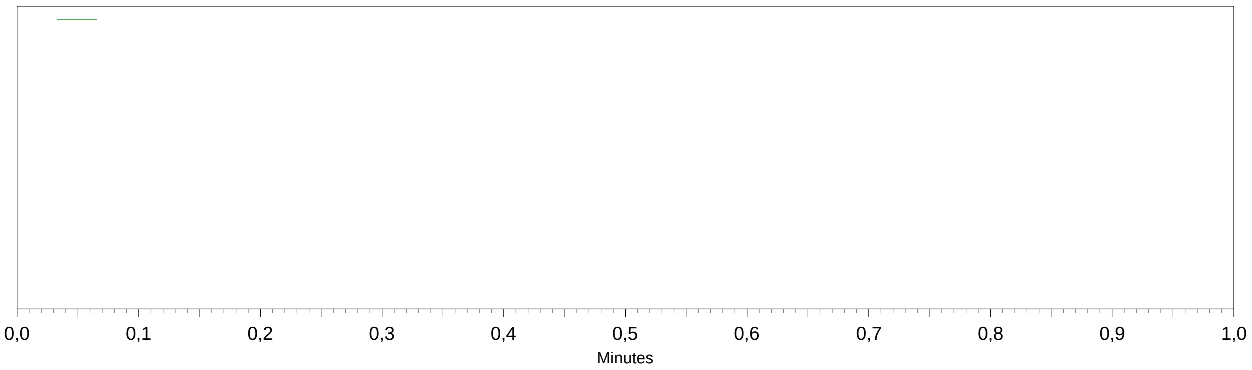

Method Name: C:\EZChrom Elite\Enterprise\Projects\Reinheit_Irina\Method\ACN-H2O\ACN-H2O_90-10_15min.met
Data: C:\EZChrom Elite\Enterprise\Projects\Reinheit_Irina\Data\2019-02-01\KuIna028_01.02.2019 13-22-26_ACN-Puffer_15-85_15min.met
User: Irina Ihnatenko
Acquired: 01.02.2019 13:23:44
Printed: 07.02.2019 16:42:52
Sample ID: KuIna028
Injectionvolume: 20

Pk #	*Name*	*Retention Time*	*Area Percent*	*Area*

Spectrum Report

Spectra of all named detected peaks

(The peak spectrum is defined as the peak apex spectrum)

Multi-Chrom 1 (1: 254 nm, 4 nm) Spectra

Retention time: 3,800 Min
Peak name: KuIna028
Lambda max: 246, 245, 244
Lambda min: 346, 353, 381

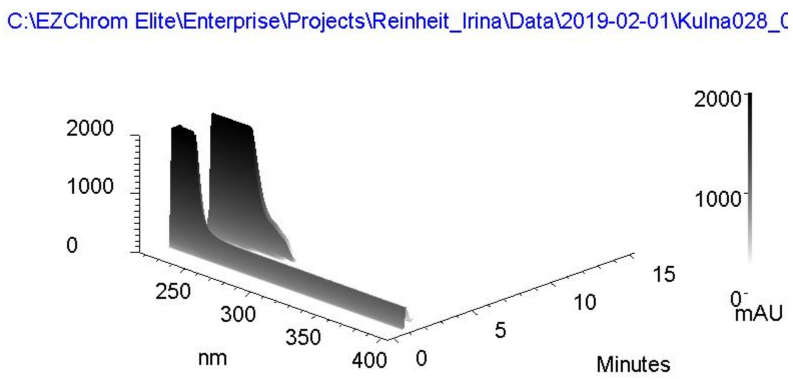

Supplement: S3 File — (ZIP) [file pone.0292946.s003.zip › S4_ZIP-File_HPLC_chromatograms/HPLC-Merck-cmpd-2t-iso-254nm.pdf]
