# Supplementary material for: The indole motif is essential for the antitrypanosomal activity of N5-substituted paullones
Source: PLoS One. 2023 Nov 30;18(11):e0292946. doi: 10.1371/journal.pone.0292946 (PMC10688702; doi:10.1371/journal.pone.0292946)

TU Braunschweig Institut für Pharmazeutische Chemie

Method Name: C:\EZChrom Elite\Enterprise\Projects\Reinheit_Irina\Method\ACN-H2O\ACN-H2O_90-10_15min.met
Data: C:\EZChrom Elite\Enterprise\Projects\Reinheit_Irina\Data\2019-02-01\KuIna034_01.02.2019 12-33-39_ACN-Puffer_10-90_15min.met
User: Irina Ihnatenko
Acquired: 01.02.2019 12:34:59
Printed: 07.02.2019 17:08:00
Sample ID: KuIna034
Injectionvolume: 20

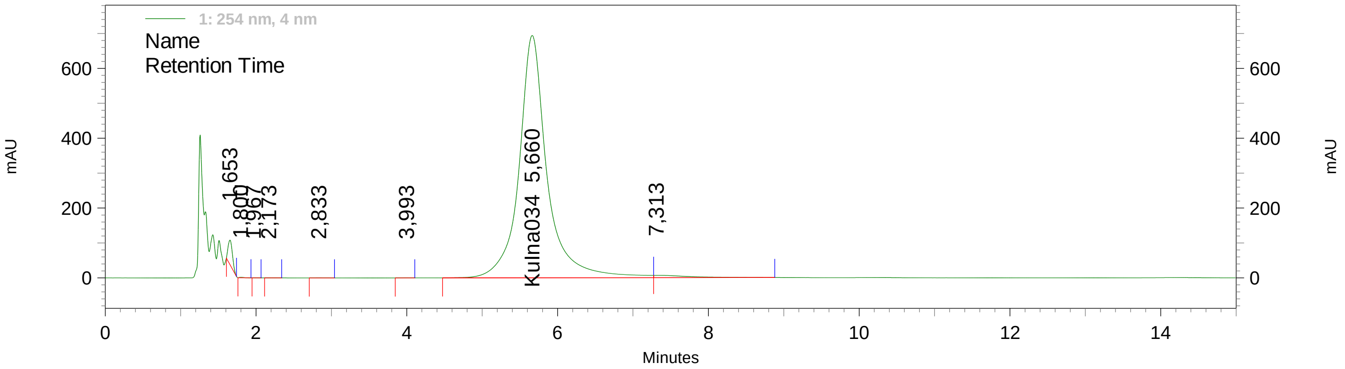

1: 254 nm, 4 nm
Results

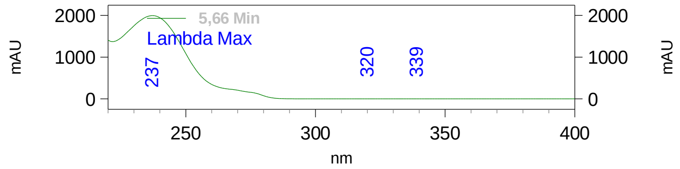

Pk #	Name	Retention Time	Area Percent	Area
1		1,653	1,525	1126488
2		1,800	0,028	20352
3		1,967	0,002	1337
4		2,173	0,003	2576
5		2,833	0,016	11568
6		3,993	0,010	7020
7	**KuIna034**	5,660	97,230	71819202
8		7,313	1,187	876620
Totals			100,000	73865163

Method Name: **C:\EZChrom Elite\Enterprise\Projects\Reinheit_Irina\Method\ACN-H2O\ACN-H2O_90-10_15min.met**
Data: **C:\EZChrom Elite\Enterprise\Projects\Reinheit_Irina\Data\2019-02-01\KuIna034_01.02.2019 12-33-39_ACN-Puffer_10-90_15min.met**
User: **Irina Ihnatenko**
Acquired: **01.02.2019 12:34:59**
Printed: **07.02.2019 17:08:00**
Sample ID: **KuIna034**
Injectionvolume: **20**

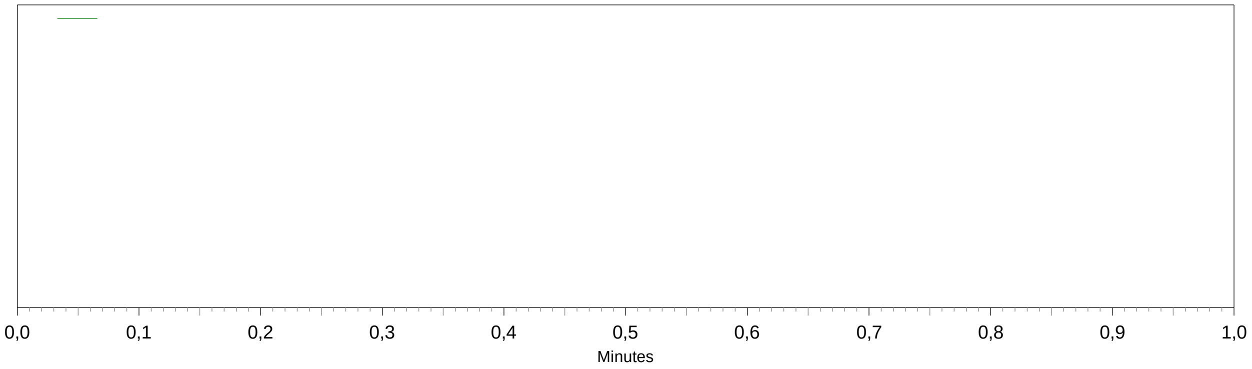

Pk #	*Name*	*Retention Time*	*Area Percent*	*Area*

Spectrum Report

Spectra of all named detected peaks

(The peak spectrum is defined as the peak apex spectrum)

Multi-Chrom 1 (1: 254 nm, 4 nm) Spectra

Retention time: 5,660 Min
Peak name: KuIna034
Lambda max: 237, 339, 320
Lambda min: 343, 370, 398

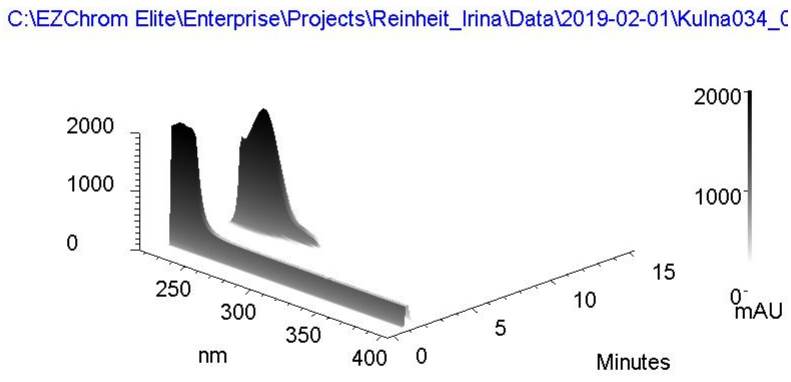

Supplement: S3 File — (ZIP) [file pone.0292946.s003.zip › S4_ZIP-File_HPLC_chromatograms/HPLC-Merck-cmpd-2u-iso-254nm.pdf]
