# Supplementary material for: The indole motif is essential for the antitrypanosomal activity of N5-substituted paullones
Source: PLoS One. 2023 Nov 30;18(11):e0292946. doi: 10.1371/journal.pone.0292946 (PMC10688702; doi:10.1371/journal.pone.0292946)

TU Braunschweig Institut für Pharmazeutische Chemie

Method Name: C:\EZChrom Elite\Enterprise\Projects\Reinheit Christian\Method\Puffer\Gradient20_80.met

Data: C:\EZChrom Elite\Enterprise\Projects\Reinheit_Irina\Data\KuIna042_10µL__26.06.2019 14-22-19_ACN-Puffer_10-90_15min.met
User: Irina Ihnatenko
Acquired: 26.06.2019 14:23:30
Printed: 26.06.2019 15:13:30
Sample ID: KuIna042_10µL_
Injectionvolume: 10

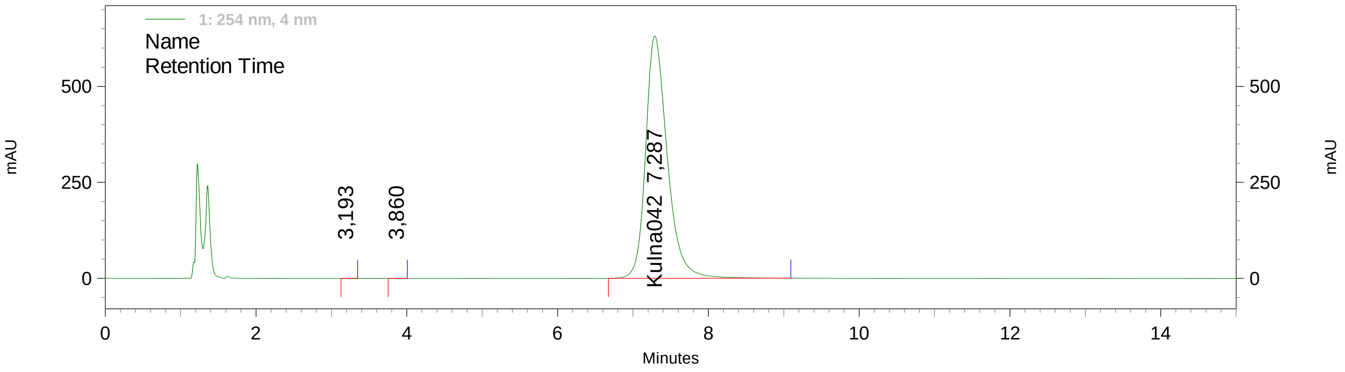

1: 254 nm, 4 nm
Results

Pk #	*Name*	*Retention Time*	*Area Percent*	*Area*
1		3,193	0,007	3610
2		3,860	0,010	4819
3	**KuIna042**	7,287	99,983	50512343
Totals			100,000	50520772

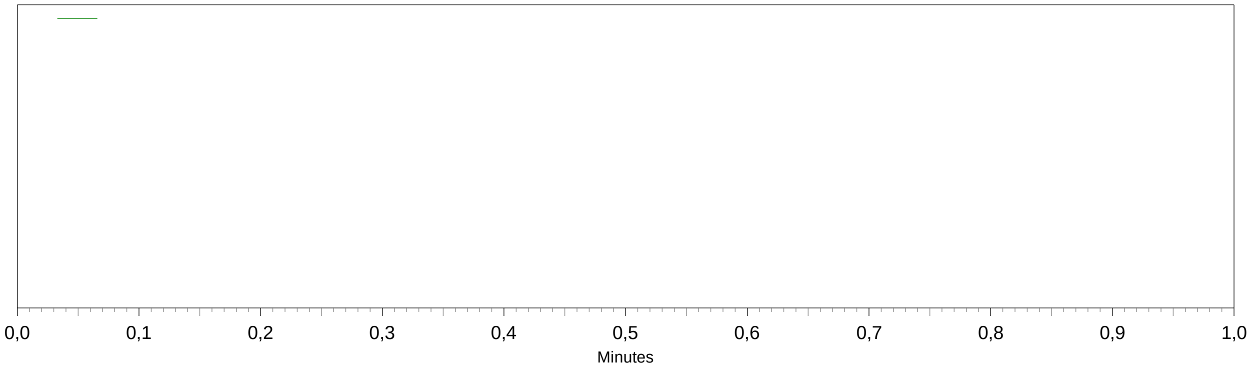

Pk #	*Name*	*Retention Time*	*Area Percent*	*Area*

Spectrum Report

Spectra of all named detected peaks

(The peak spectrum is defined as the peak apex spectrum)

Method Name: **C:\EZChrom Elite\Enterprise\Projects\Reinheit Christian\Method\Puffer\Gradient20_80.met**

Data: **C:\EZChrom Elite\Enterprise\Projects\Reinheit_Irina\Data\KuIna042_10µL__26.06.2019 14-22-19_ACN-Puffer_10-90_15min.met**
User: **Irina Ihnatenko**
Acquired: **26.06.2019 14:23:30**
Printed: **26.06.2019 15:13:30**
Sample ID: **KuIna042_10µL_**
Injectionvolume: **10**

Multi-Chrom 1 (1: 254 nm, 4 nm) Spectra

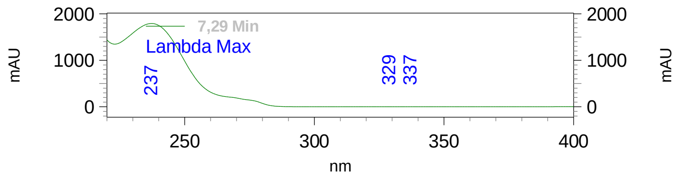

Retention time: 7,287 Min
Peak name: KuIna042
Lambda max: 237, 337, 329
Lambda min: 342, 323, 312

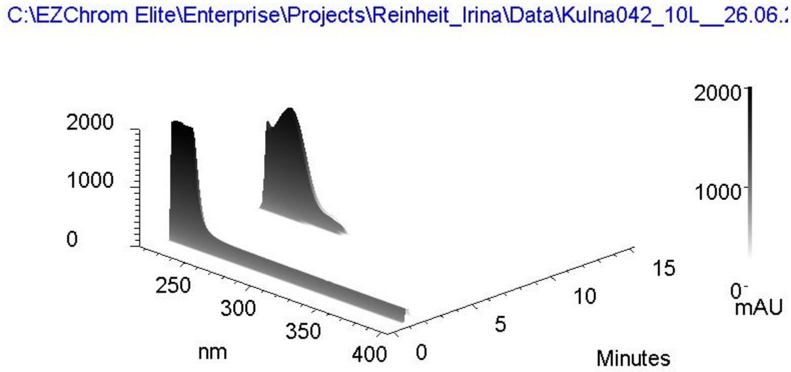

Supplement: S3 File — (ZIP) [file pone.0292946.s003.zip › S4_ZIP-File_HPLC_chromatograms/HPLC-Merck-cmpd-2v-iso-254nm.pdf]
