# Supplementary material for: The indole motif is essential for the antitrypanosomal activity of N5-substituted paullones
Source: PLoS One. 2023 Nov 30;18(11):e0292946. doi: 10.1371/journal.pone.0292946 (PMC10688702; doi:10.1371/journal.pone.0292946)

TU Braunschweig Institut für Pharmazeutische Chemie

Method Name: C:\EZChrom Elite\Enterprise\Projects\Reinheit_Irina\Method\ACN-H2O\ACN-H2O_90-10_1min_0,1µL.met
Data: C:\EZChrom Elite\Enterprise\Projects\Reinheit_Irina\Data\KuIna063 isokratisch_10µL_03.02.2020 17-51-54_ACN-Puffer_35-65_15min.met
User: Irina Ihnatenko
Acquired: 03.02.2020 17:53:07
Printed: 03.02.2020 19:16:47
Sample ID: KuIna063 isokratisch_10µL
Injectionvolume: 10

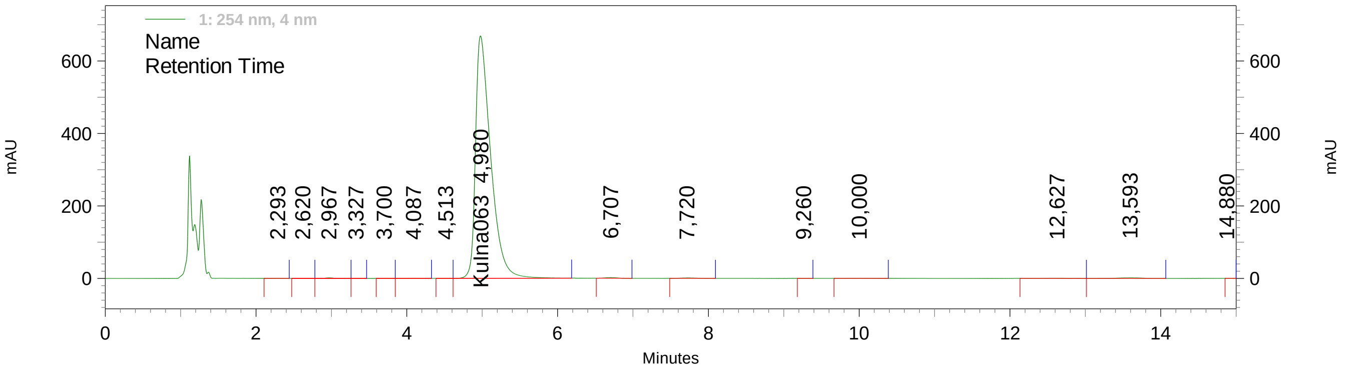

1: 254 nm. 4 nm

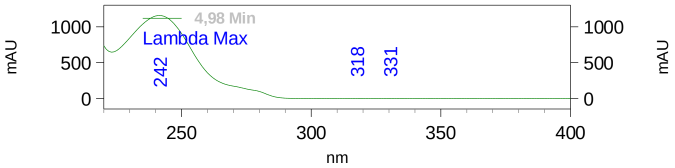

Pk #	*Name*	*Retention Time*	*Area Percent*	*Area*
1		2,293	0,025	9468
2		2,620	0,030	11231
3		2,967	0,147	55858
4		3,327	0,009	3367
5		3,700	0,041	15432
6		4,087	0,072	27447
7		4,513	0,024	9005
8	**KuIna063**	4,980	98,468	37319201
9		6,707	0,218	82475
10		7,720	0,187	70976
11		9,260	0,009	3384
12		10,000	0,116	44010
13		12,627	0,208	78925
14		13,593	0,442	167637
15		14,880	0,003	1226

Totals				
			100,000	37899642

Method Name: C:\EZChrom Elite\Enterprise\Projects\Reinheit_Irina\Method\ACN-H2O\ACN-H2O_90-10_1min_0,1µL.met
Data: C:\EZChrom Elite\Enterprise\Projects\Reinheit_Irina\Data\KuIna063 isokratisch_10µL_03.02.2020 17-51-54_ACN-Puffer_35-65_15min.met
User: Irina Ihnatenko
Acquired: 03.02.2020 17:53:07
Printed: 03.02.2020 19:16:47
Sample ID: KuIna063 isokratisch_10µL
Injectionvolume: 10

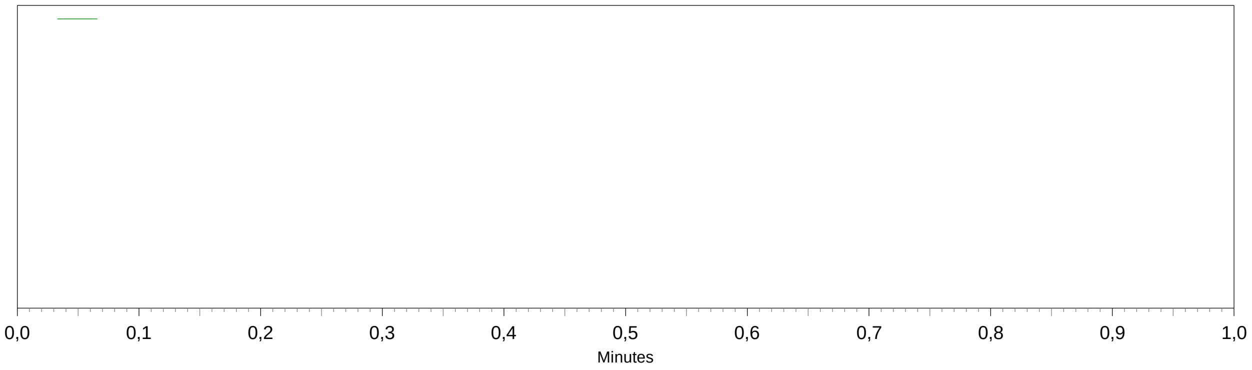

Pk #	*Name*	*Retention Time*	*Area Percent*	*Area*

Spectrum Report
Spectra of all named detected peaks

(The peak spectrum is defined as the peak apex spectrum)

Multi-Chrom 1 (1: 254 nm, 4 nm) Spectra

Retention time: 4,980 Min
Peak name: KuIna063
Lambda max: 242, 318, 331
Lambda min: 381, 370, 390

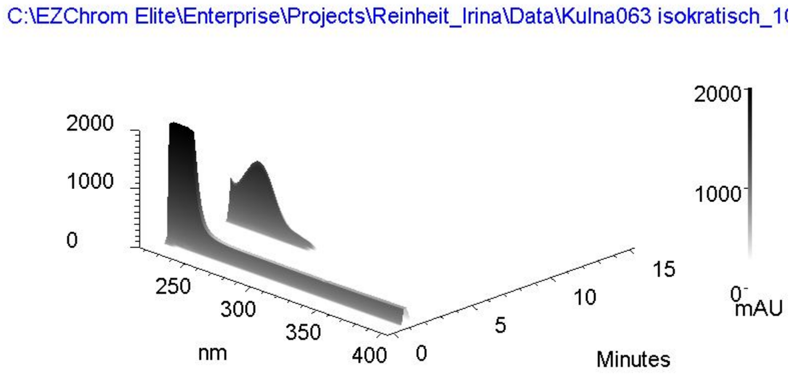

Supplement: S3 File — (ZIP) [file pone.0292946.s003.zip › S4_ZIP-File_HPLC_chromatograms/HPLC-Merck-cmpd-3b-iso-254nm.pdf]
