# Supplementary material for: The indole motif is essential for the antitrypanosomal activity of N5-substituted paullones
Source: PLoS One. 2023 Nov 30;18(11):e0292946. doi: 10.1371/journal.pone.0292946 (PMC10688702; doi:10.1371/journal.pone.0292946)

TU Braunschweig Institut für Pharmazeutische Chemie

Method Name: **C:\EZChrom Elite\Enterprise\Projects\Reinheit_Irina\Method\ACN-H2O\ACN-H2O_90-10_1min_0,1µL.met**
Data: **C:\EZChrom Elite\Enterprise\Projects\Reinheit_Irina\Data\KuIna066 isokratisch_10µL_03.02.2020 18-24-03_ACN-Puffer_20-80_15min.met**
User: **Irina Ihnatenko**
Acquired: **03.02.2020 18:25:13**
Printed: **03.02.2020 19:41:29**
Sample ID: **KuIna066 isokratisch_10µL**
Injectionvolume: **10**

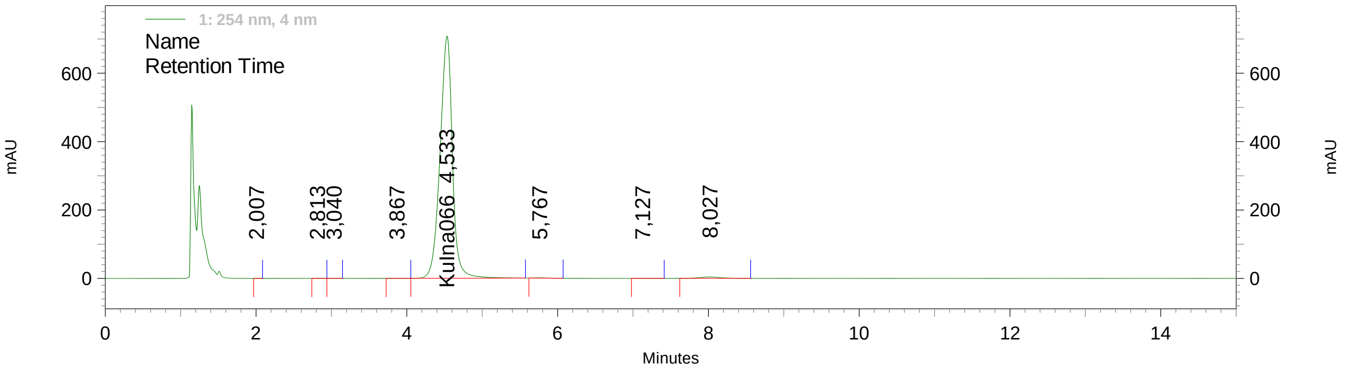

1: 254 nm. 4 nm

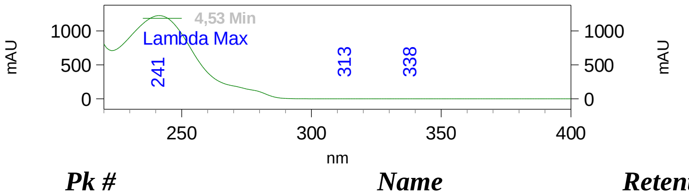

Pk #	*Name*	*Retention Time*	*Area Percent*	*Area*
1		2,007	0,006	1935
2		2,813	0,023	7377
3		3,040	0,019	6256
4		3,867	0,063	20456
5	**KuIna066**	4,533	98,810	32310880
6		5,767	0,096	31305
7		7,127	0,030	9706
8		8,027	0,955	312249

Totals				
			100,000	32700164

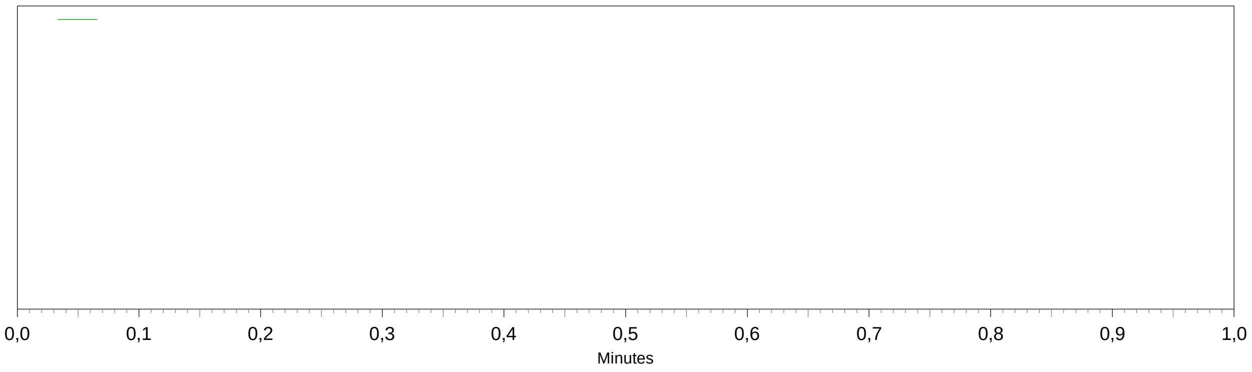

Method Name: **C:\EZChrom Elite\Enterprise\Projects\Reinheit_Irina\Method\ACN-H2O\ACN-H2O_90-10_1min_0,1µL.met**
Data: **C:\EZChrom Elite\Enterprise\Projects\Reinheit_Irina\Data\KuIna066 isokratisch_10µL_03.02.2020 18-24-03_ACN-Puffer_20-80_15min.met**
User: **Irina Ihnatenko**
Acquired: **03.02.2020 18:25:13**
Printed: **03.02.2020 19:41:29**
Sample ID: **KuIna066 isokratisch_10µL**
Injectionvolume: **10**

Pk #	*Name*	*Retention Time*	*Area Percent*	*Area*

Spectrum Report

Spectra of all named detected peaks

(The peak spectrum is defined as the peak apex spectrum)

Multi-Chrom 1 (1: 254 nm, 4 nm) Spectra

Retention time: 4,533 Min
Peak name: KuIna066
Lambda max: 241, 338, 313
Lambda min: 384, 355, 347

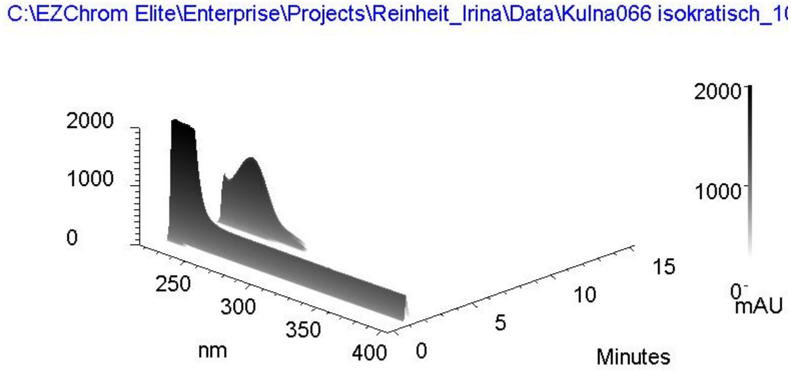

Supplement: S3 File — (ZIP) [file pone.0292946.s003.zip › S4_ZIP-File_HPLC_chromatograms/HPLC-Merck-cmpd-3d-iso-254nm.pdf]
