# Supplementary material for: The indole motif is essential for the antitrypanosomal activity of N5-substituted paullones
Source: PLoS One. 2023 Nov 30;18(11):e0292946. doi: 10.1371/journal.pone.0292946 (PMC10688702; doi:10.1371/journal.pone.0292946)

TU Braunschweig Institut für Pharmazeutische Chemie

Method Name: C:\EZChrom Elite\Enterprise\Projects\Reinheit_Irina\Method\ACN-H2O\ACN-H2O_90-10_1min_0,1µL.met
Data: C:\EZChrom Elite\Enterprise\Projects\Reinheit_Irina\Data\KuIna044_5µL_04.11.2019 18-26-27_ACN-Puffer_25-75_15min.met
User: Irina Ihnatenko
Acquired: 04.11.2019 18:27:33
Printed: 04.11.2019 19:11:18
Sample ID: KuIna044_5µL
Injectionvolume: 5

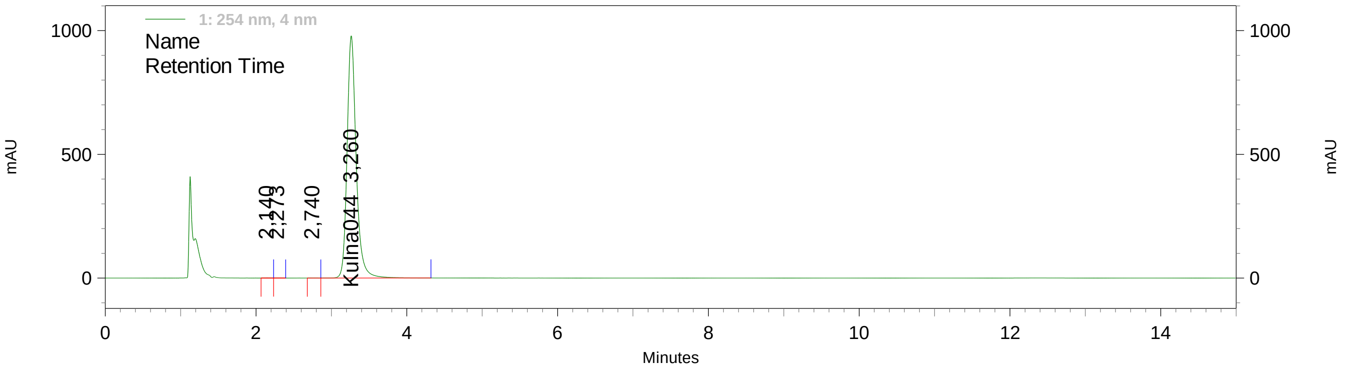

1: 254 nm. 4 nm

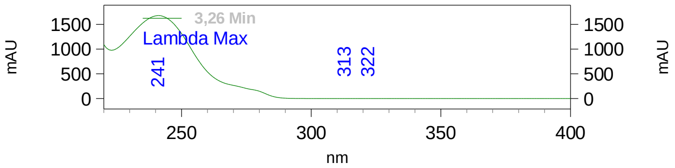

Pk #	*Name*	*Retention Time*	*Area Percent*	*Area*
1		2,140	0,047	15418
2		2,273	0,019	6265
3		2,740	0,007	2422
4	**KuIna044**	3,260	99,927	32915418
Totals			100,000	32939523

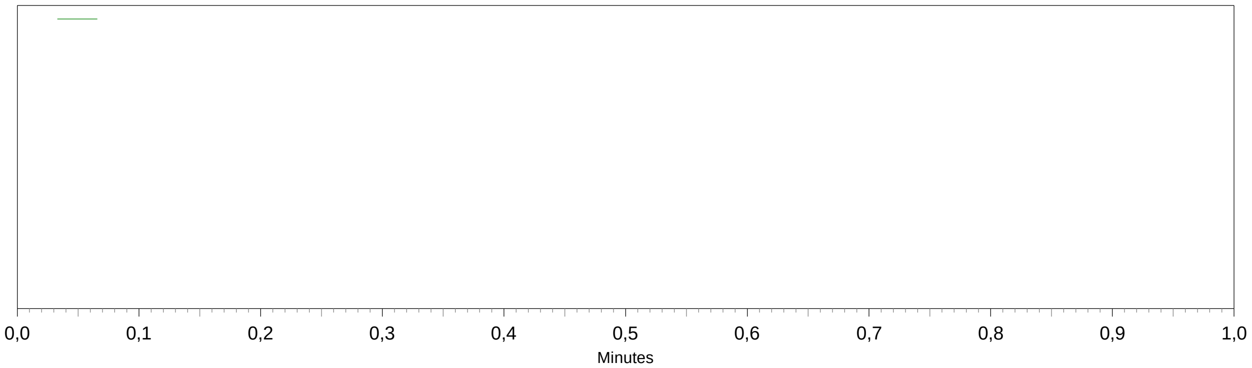

Pk #	*Name*	*Retention Time*	*Area Percent*	*Area*

Spectrum Report

Method Name: **C:\EZChrom Elite\Enterprise\Projects\Reinheit_Irina\Method\ACN-H2O\ACN-H2O_90-10_1min_0,1µL.met**
Data: **C:\EZChrom Elite\Enterprise\Projects\Reinheit_Irina\Data\KuIna044_5µL_04.11.2019 18-26-27_ACN-Puffer_25-75_15min.met**
User: **Irina Ihnatenko**
Acquired: **04.11.2019 18:27:33**
Printed: **04.11.2019 19:11:18**
Sample ID: **KuIna044_5µL**
Injectionvolume: **5**

Spectra of all named detected peaks

(The peak spectrum is defined as the peak apex spectrum)

Multi-Chrom 1 (1: 254 nm, 4 nm) Spectra

Retention time: 3,260 Min
Peak name: KuIna044
Lambda max: 241, 313, 322
Lambda min: 388, 377, 360

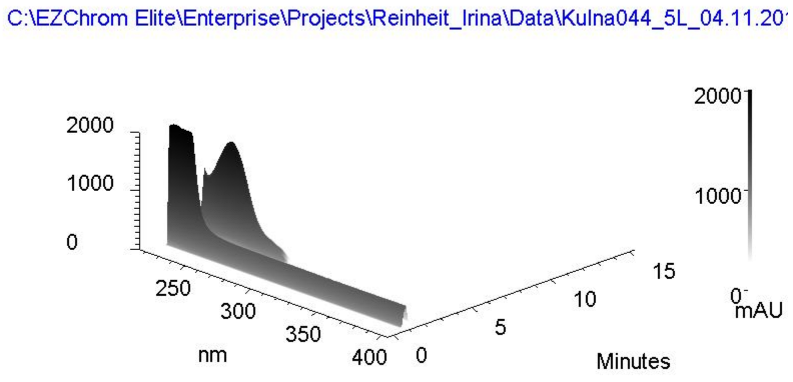

Supplement: S3 File — (ZIP) [file pone.0292946.s003.zip › S4_ZIP-File_HPLC_chromatograms/HPLC-Merck-cmpd-3e-iso-254nm.pdf]
