# Supplementary material for: The indole motif is essential for the antitrypanosomal activity of N5-substituted paullones
Source: PLoS One. 2023 Nov 30;18(11):e0292946. doi: 10.1371/journal.pone.0292946 (PMC10688702; doi:10.1371/journal.pone.0292946)

Method Name: **C:\EZChrom Elite\Enterprise\Projects\Reinheit_Irina\Method\ACN-H2O\ACN-H2O_90-10_1min_0,1µL.met**
Data: **C:\EZChrom Elite\Enterprise\Projects\Reinheit_Irina\Data\KuIna065 isokratisch_5µL_03.02.2020 16-31-05_ACN-Puffer_30-70_15min.met**
User: **Irina Ihnatenko**
Acquired: **03.02.2020 16:32:11**
Printed: **03.02.2020 19:29:14**
Sample ID: **KuIna065 isokratisch_5µL**
Injectionvolume: **5**

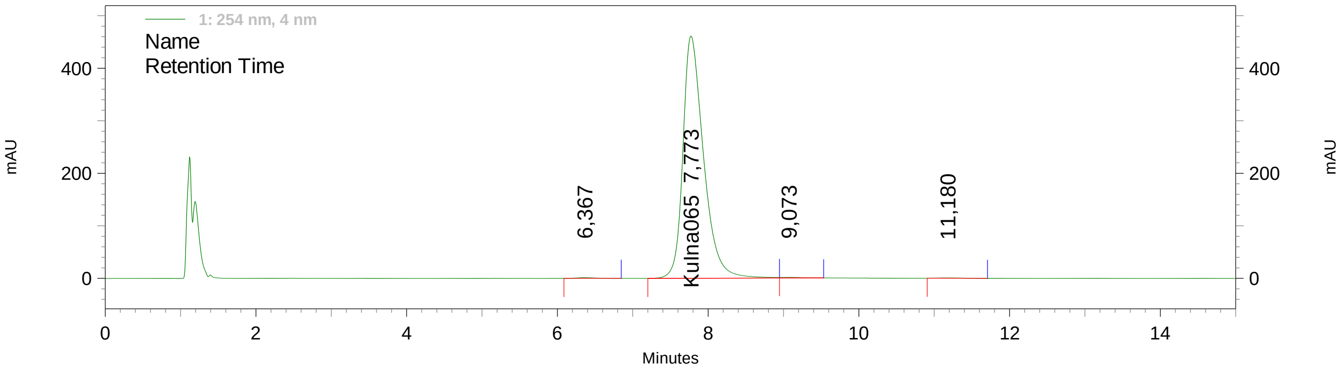

1: 254 nm. 4 nm

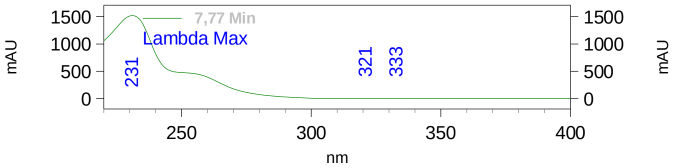

Pk #	*Name*	*Retention Time*	*Area Percent*	*Area*
1		6,367	0,252	89156
2	**KuIna065**	7,773	99,346	35124239
3		9,073	0,238	84220
4		11,180	0,163	57753

Totals				
			100,000	35355368

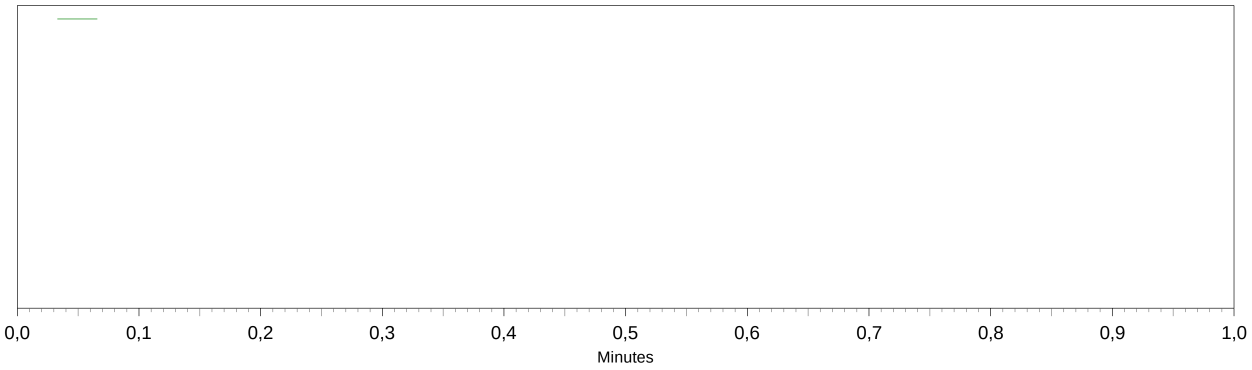

Pk #	*Name*	*Retention Time*	*Area Percent*	*Area*

Spectrum Report

Method Name: **C:\EZChrom Elite\Enterprise\Projects\Reinheit_Irina\Method\ACN-H2O\ACN-H2O_90-10_1min_0,1µL.met**
Data: **C:\EZChrom Elite\Enterprise\Projects\Reinheit_Irina\Data\KuIna065 isokratisch_5µL_03.02.2020 16-31-05_ACN-Puffer_30-70_15min.met**
User: **Irina Ihnatenko**
Acquired: **03.02.2020 16:32:11**
Printed: **03.02.2020 19:29:14**
Sample ID: **KuIna065 isokratisch_5µL**
Injectionvolume: **5**

Spectra of all named detected peaks

(The peak spectrum is defined as the peak apex spectrum)

Multi-Chrom 1 (1: 254 nm, 4 nm) Spectra

Retention time: 7,773 Min
Peak name: KuIna065
Lambda max: 231, 321, 333
Lambda min: 378, 357, 394

C:\EZChrom Elite\Enterprise\Projects\Reinheit_Irina\Data\KuIna065 isokratisch_5l

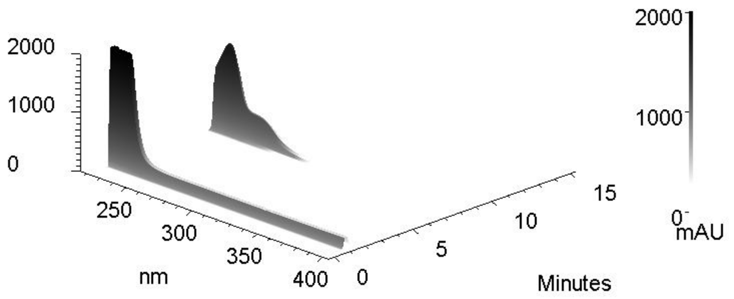

Supplement: S3 File — (ZIP) [file pone.0292946.s003.zip › S4_ZIP-File_HPLC_chromatograms/HPLC-Merck-cmpd-4b-iso-254nm.pdf]
