# Supplementary material for: The indole motif is essential for the antitrypanosomal activity of N5-substituted paullones
Source: PLoS One. 2023 Nov 30;18(11):e0292946. doi: 10.1371/journal.pone.0292946 (PMC10688702; doi:10.1371/journal.pone.0292946)

TU Braunschweig Institut für Pharmazeutische Chemie

Method Name: C:\EZChrom Elite\Enterprise\Projects\Reinheit_Irina\Method\ACN-H2O\ACN-H2O_90-10_1min_0,1µL.met
Data: C:\EZChrom Elite\Enterprise\Projects\Reinheit_Irina\Data\KuIna067 isokratisch_5µL_03.02.2020 18-56-47_ACN-Puffer_20-80_15min.met
User: Irina Ihnatenko
Acquired: 03.02.2020 18:57:52
Printed: 03.02.2020 19:24:08
Sample ID: KuIna067 isokratisch_5µL
Injectionvolume: 5

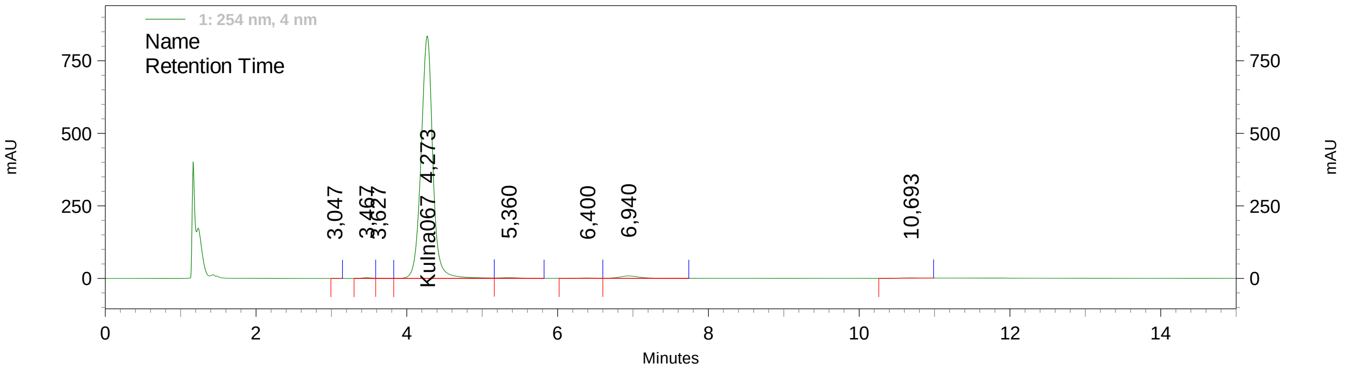

1: 254 nm. 4 nm

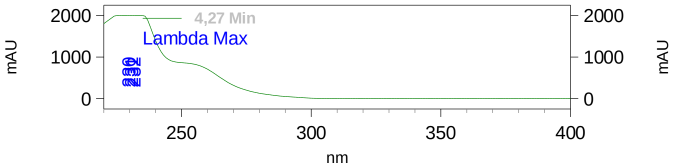

Pk #	*Name*	*Retention Time*	*Area Percent*	*Area*
1		3,047	0,005	1997
2		3,467	0,210	77699
3		3,627	0,066	24412
4	**KuIna067**	4,273	97,233	35913094
5		5,360	0,443	163448
6		6,400	0,162	60003
7		6,940	1,667	615526
8		10,693	0,214	78866
Totals			100,000	36935045

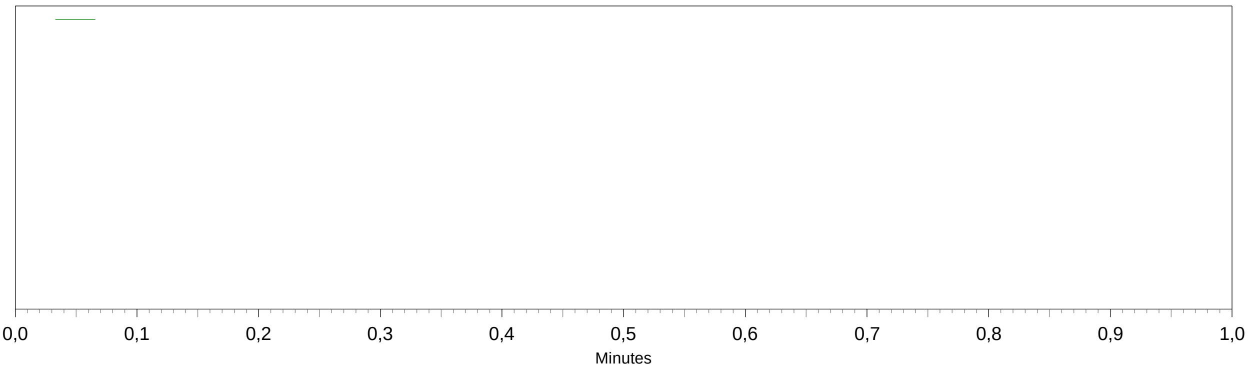

Method Name: **C:\EZChrom Elite\Enterprise\Projects\Reinheit_Irina\Method\ACN-H2O\ACN-H2O_90-10_1min_0,1µL.met**
Data: **C:\EZChrom Elite\Enterprise\Projects\Reinheit_Irina\Data\KuIna067 isokratisch_5µL_03.02.2020 18-56-47_ACN-Puffer_20-80_15min.met**
User: **Irina Ihnatenko**
Acquired: **03.02.2020 18:57:52**
Printed: **03.02.2020 19:24:08**
Sample ID: **KuIna067 isokratisch_5µL**
Injectionvolume: **5**

Pk #	*Name*	*Retention Time*	*Area Percent*	*Area*

Spectrum Report
Spectra of all named detected peaks

(The peak spectrum is defined as the peak apex spectrum)

Multi-Chrom 1 (1: 254 nm, 4 nm) Spectra

Retention time: 4,273 Min
Peak name: KuIna067
Lambda max: 232, 231, 230
Lambda min: 386, 364, 348

C:\EZChrom Elite\Enterprise\Projects\Reinheit_Irina\Data\KuIna067 isokratisch_5l

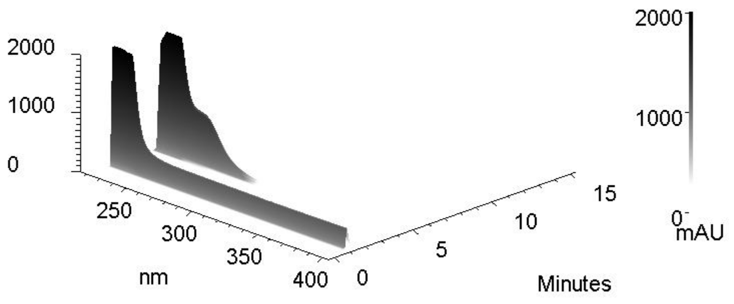

Supplement: S3 File — (ZIP) [file pone.0292946.s003.zip › S4_ZIP-File_HPLC_chromatograms/HPLC-Merck-cmpd-4d-iso-254nm.pdf]
