# Supplementary material for: The indole motif is essential for the antitrypanosomal activity of N5-substituted paullones
Source: PLoS One. 2023 Nov 30;18(11):e0292946. doi: 10.1371/journal.pone.0292946 (PMC10688702; doi:10.1371/journal.pone.0292946)

TU Braunschweig Institut für Pharmazeutische Chemie

Method Name: C:\EZChrom Elite\Enterprise\Projects\Reinheit_Irina\Method\ACN-H2O\ACN-H2O_90-10_1min_0,1µL.met
Data: C:\EZChrom Elite\Enterprise\Projects\Reinheit_Irina\Data\KuIna070 isokratisch_5µL_03.02.2020 17-03-37_ACN-Puffer_30-70_15min.met
User: Irina Ihnatenko
Acquired: 03.02.2020 17:04:39
Printed: 03.02.2020 19:46:46
Sample ID: KuIna070 isokratisch_5µL
Injectionvolume: 5

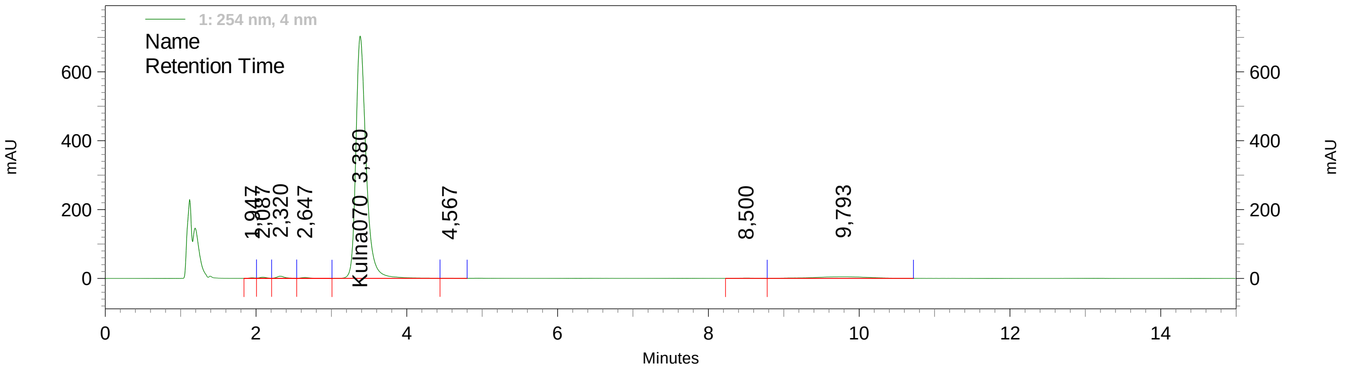

1: 254 nm. 4 nm

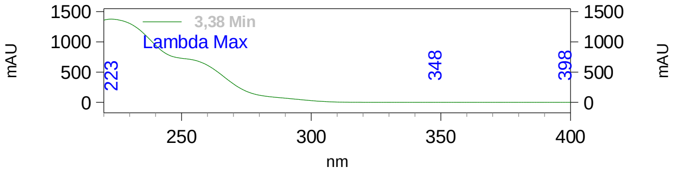

Pk #	*Name*	*Retention Time*	*Area Percent*	*Area*
1		1,947	0,125	35136
2		2,087	0,319	89418
3		2,320	0,705	197348
4		2,647	0,318	89019
5	**KuIna070**	3,380	94,799	26546090
6		4,567	0,082	22888
7		8,500	0,184	51506
8		9,793	3,468	971021

Totals				
			100,000	28002426

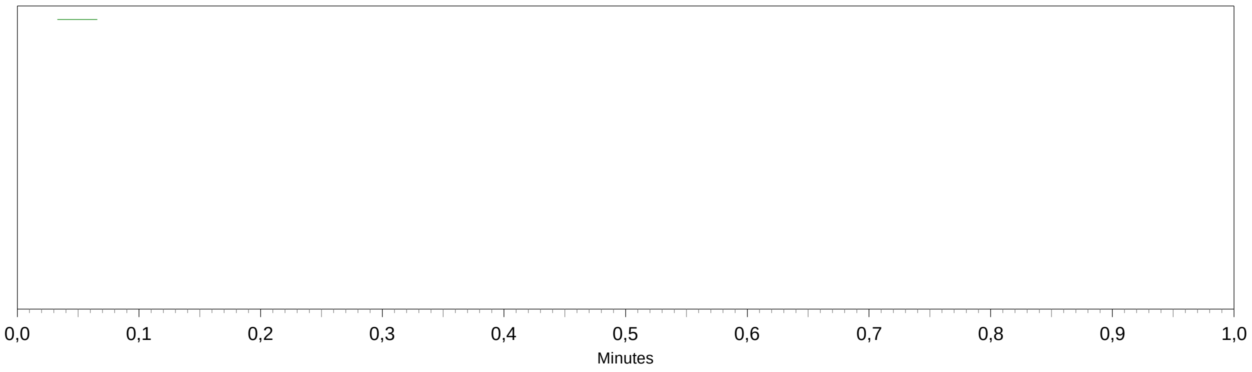

Method Name: **C:\EZChrom Elite\Enterprise\Projects\Reinheit_Irina\Method\ACN-H2O\ACN-H2O_90-10_1min_0,1µL.met**
Data: **C:\EZChrom Elite\Enterprise\Projects\Reinheit_Irina\Data\KuIna070 isokratisch_5µL_03.02.2020 17-03-37_ACN-Puffer_30-70_15min.met**
User: **Irina Ihnatenko**
Acquired: **03.02.2020 17:04:39**
Printed: **03.02.2020 19:46:46**
Sample ID: **KuIna070 isokratisch_5µL**
Injectionvolume: **5**

Pk #	*Name*	*Retention Time*	*Area Percent*	*Area*

Spectrum Report

Spectra of all named detected peaks

(The peak spectrum is defined as the peak apex spectrum)

Multi-Chrom 1 (1: 254 nm, 4 nm) Spectra

Retention time: 3,380 Min
Peak name: KuIna070
Lambda max: 223, 348, 398
Lambda min: 360, 392, 381

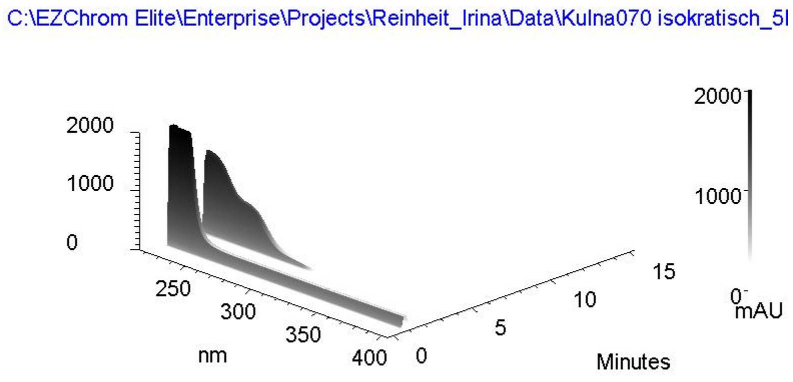

Supplement: S3 File — (ZIP) [file pone.0292946.s003.zip › S4_ZIP-File_HPLC_chromatograms/HPLC-Merck-cmpd-5b-iso-254nm.pdf]
