# Supplementary material for: The indole motif is essential for the antitrypanosomal activity of N5-substituted paullones
Source: PLoS One. 2023 Nov 30;18(11):e0292946. doi: 10.1371/journal.pone.0292946 (PMC10688702; doi:10.1371/journal.pone.0292946)

TU Braunschweig Institut für Pharmazeutische Chemie

Method Name: C:\EZChrom Elite\Enterprise\Projects\Reinheit Sandra\Method\untitled.met
Data: C:\EZChrom Elite\Enterprise\Projects\Reinheit_Irina\Data\KuIna071_15µL_19.02.2020 13-05-45_ACN-Puffer_10-90_15min.met
User: Irina Ihnatenko
Acquired: 19.02.2020 13:06:57
Printed: 19.02.2020 13:50:38
Sample ID: KuIna071_15µL
Injectionvolume: 15

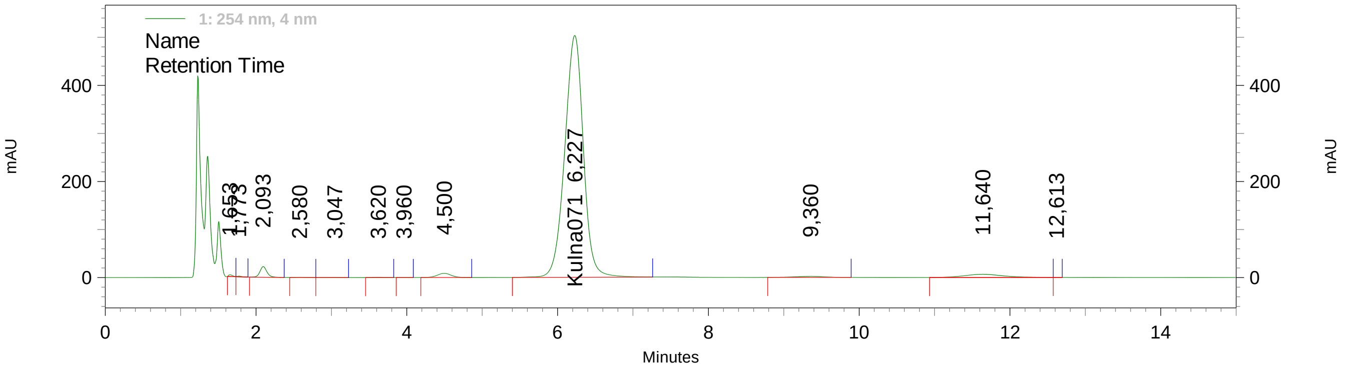

1: 254 nm, 4 nm

Results

Pk #	*Name*	*Retention Time*	*Area Percent*	*Area*
1		1,653	0,150	55468
2		1,773	0,066	24536
3		2,093	1,553	573482
4		2,580	0,037	13818
5		3,047	0,054	19803
6		3,620	0,064	23474
7		3,960	0,016	5968
8		4,500	1,218	449976
9	**KuIna071**	6,227	93,653	34585684
10		9,360	0,618	228248
11		11,640	2,566	947605
12		12,613	0,004	1660
Totals			100,000	36929722

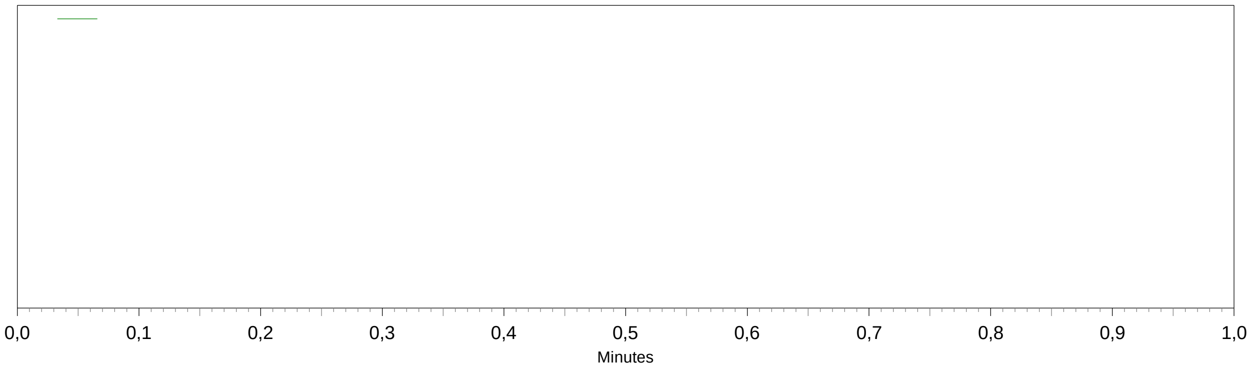

Pk #	*Name*	*Retention Time*	*Area Percent*	*Area*

Method Name: **C:\EZChrom Elite\Enterprise\Projects\Reinheit Sandra\Method\untitled.met**
Data: **C:\EZChrom Elite\Enterprise\Projects\Reinheit_Irina\Data\KuIna071_15µL_19.02.2020 13-05-45_ACN-Puffer_10-90_15min.met**
User: **Irina Ihnatenko**
Acquired: **19.02.2020 13:06:57**
Printed: **19.02.2020 13:50:38**
Sample ID: **KuIna071_15µL**
Injectionvolume: **15**

Spectrum Report

Spectra of all named detected peaks

(The peak spectrum is defined as the peak apex spectrum)

Multi-Chrom 1 (1: 254 nm, 4 nm) Spectra

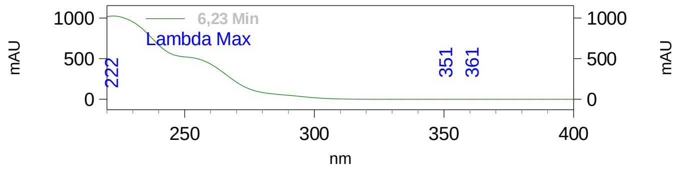

Retention time: 6,227 Min
Peak name: KuIna071
Lambda max: 222, 351, 361
Lambda min: 383, 370, 355

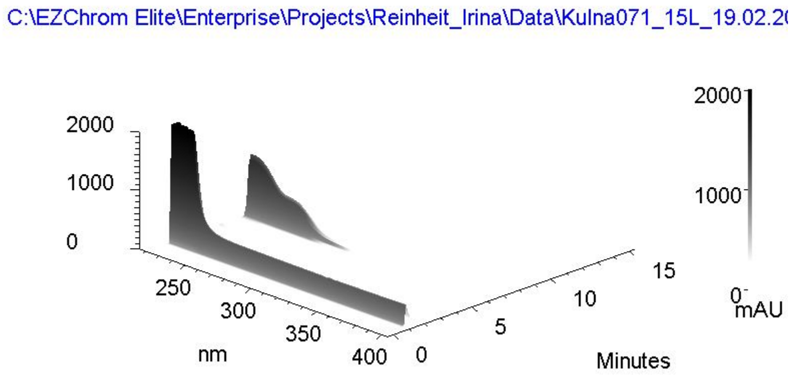

Supplement: S3 File — (ZIP) [file pone.0292946.s003.zip › S4_ZIP-File_HPLC_chromatograms/HPLC-Merck-cmpd-5d-iso-254nm.pdf]
