# Supplementary material for: The indole motif is essential for the antitrypanosomal activity of N5-substituted paullones
Source: PLoS One. 2023 Nov 30;18(11):e0292946. doi: 10.1371/journal.pone.0292946 (PMC10688702; doi:10.1371/journal.pone.0292946)

TU Braunschweig Institut für Pharmazeutische Chemie

Method Name: C:\EZChrom Elite\Enterprise\Projects\Reinheit_Irina\Method\ACN-H2O\ACN-H2O_10-90_10min.met
Data: C:\EZChrom Elite\Enterprise\Projects\Reinheit_Irina\Data\KuIna091_5µL_11.08.2020 11-49-28_ACN-Puffer_50-50_15min.met
User: Irina Ihnatenko
Acquired: 11.08.2020 11:50:31
Printed: 11.08.2020 12:10:40
Sample ID: KuIna091_5µL
Injectionvolume: 5

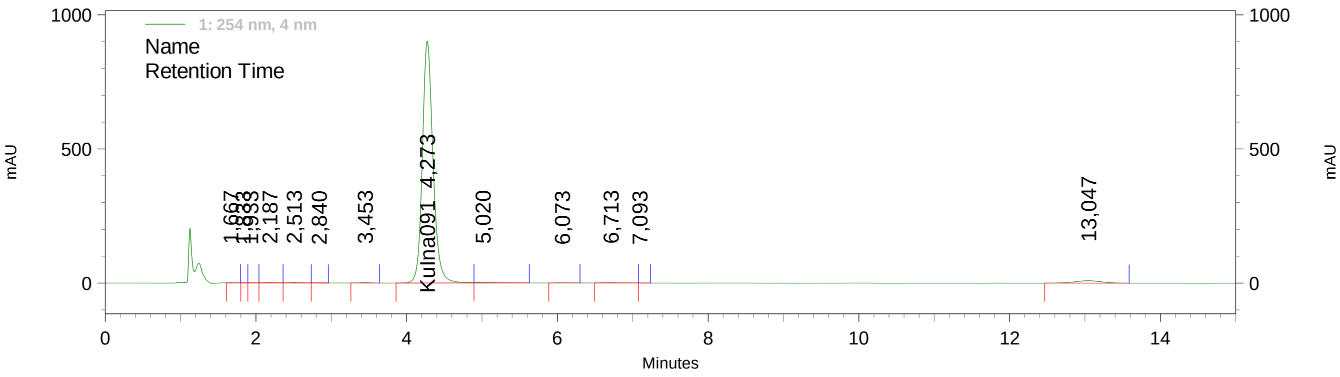

1: 254 nm, 4 nm

Results

Pk #	*Name*	*Retention Time*	*Area Percent*	*Area*
1		1,667	0,039	14518
2		1,833	0,004	1507
3		1,933	0,010	3797
4		2,187	0,104	38444
5		2,513	0,182	67349
6		2,840	0,052	19102
7		3,453	0,115	42386
8	**KuIna091**	4,273	96,410	35578984
9		5,020	0,439	162182
10		6,073	0,090	33248
11		6,713	0,177	65247
12		7,093	0,010	3652
13		13,047	2,367	873435
Totals			100,000	36903851

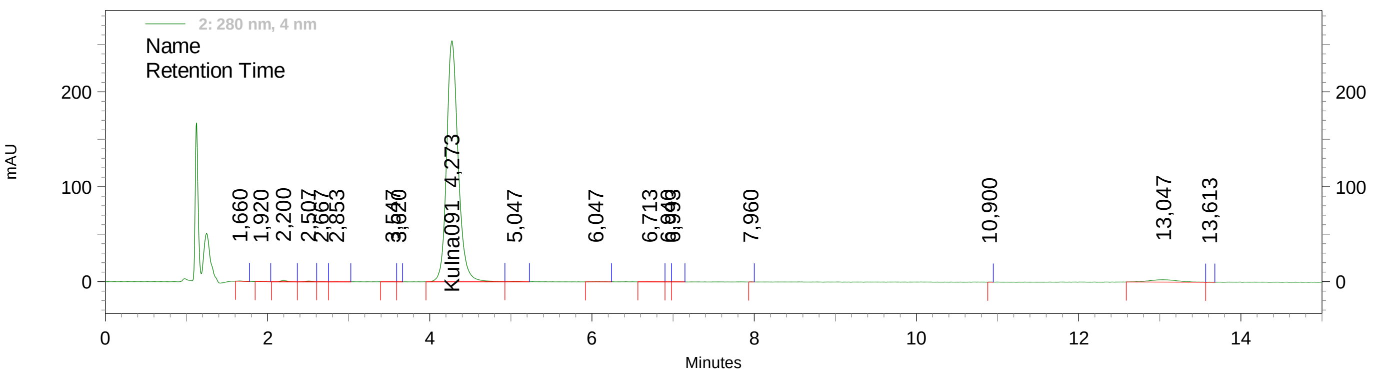

Method Name: C:\EZChrom Elite\Enterprise\Projects\Reinheit_Irina\Method\ACN-H2O\ACN-H2O_10-90_10min.met
Data: C:\EZChrom Elite\Enterprise\Projects\Reinheit_Irina\Data\KuIna091_5µL_11.08.2020 11-49-28_ACN-Puffer_50-50_15min.met
User: Irina Ihnatenko
Acquired: 11.08.2020 11:50:31
Printed: 11.08.2020 12:10:40
Sample ID: KuIna091_5µL
Injectionvolume: 5
2: 280 nm, 4 nm
Results

Pk #	*Name*	*Retention Time*	*Area Percent*	*Area*
1		1,660	0,057	5942
2		1,920	0,052	5458
3		2,200	0,286	29824
4		2,507	0,206	21402
5		2,667	0,065	6739
6		2,853	0,065	6779
7		3,547	0,055	5733
8		3,620	0,008	804
9	**KuIna091**	4,273	96,359	10033908
10		5,047	0,193	20127
11		6,047	0,099	10329
12		6,713	0,116	12117
13		6,940	0,022	2275
14		6,993	0,046	4747
15		7,960	0,013	1347
16		10,900	0,006	603
17		13,047	2,341	243808
18		13,613	0,011	1106
Totals			100,000	10413048

Spectrum Report

Spectra of all named detected peaks

(The peak spectrum is defined as the peak apex spectrum)

Multi-Chrom 1 (1: 254 nm, 4 nm) Spectra

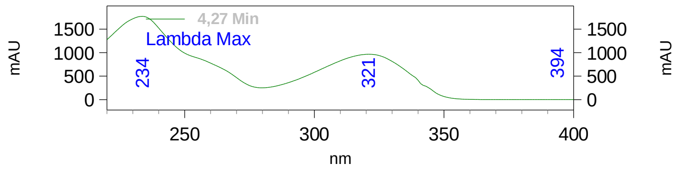

Retention time: 4,273 Min
Peak name: KuIna091
Lambda max: 234, 321, 394
Lambda min: 381, 280

Multi-Chrom 2 (2: 280 nm, 4 nm) Spectra

Method Name: C:\EZChrom Elite\Enterprise\Projects\Reinheit_Irina\Method\ACN-H2O\ACN-H2O_10-90_10min.met
Data: C:\EZChrom Elite\Enterprise\Projects\Reinheit_Irina\Data\KuIna091_5µL_11.08.2020 11-49-28_ACN-Puffer_50-50_15min.met
User: Irina Ihnatenko
Acquired: 11.08.2020 11:50:31
Printed: 11.08.2020 12:10:40
Sample ID: KuIna091_5µL
Injectionvolume: 5

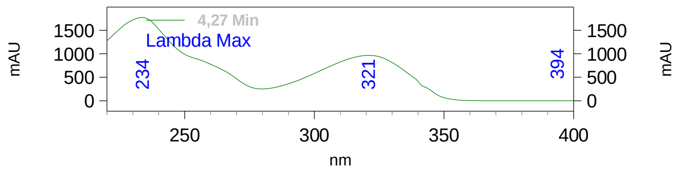

Retention time: 4,273 Min
Peak name: KuIna091
Lambda max: 234, 321, 394
Lambda min: 381, 280

C:\EZChrom Elite\Enterprise\Projects\Reinheit_Irina\Data\KuIna091_5L_11.08.20[illegible]

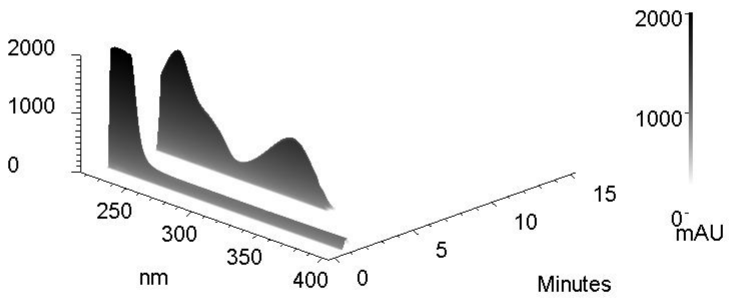

Supplement: S3 File — (ZIP) [file pone.0292946.s003.zip › S4_ZIP-File_HPLC_chromatograms/HPLC-Merck-cmpd-9a-iso-254+280nm.pdf]
