# Supplementary material for: The indole motif is essential for the antitrypanosomal activity of N5-substituted paullones
Source: PLoS One. 2023 Nov 30;18(11):e0292946. doi: 10.1371/journal.pone.0292946 (PMC10688702; doi:10.1371/journal.pone.0292946)

Method Name: C:\EZChrom Elite\Enterprise\Projects\Reinheit_Irina\Method\ACN-H2O\ACN-H2O_10-90_10min.met
Data: C:\EZChrom Elite\Enterprise\Projects\Reinheit_Irina\Data\KuIna092_5µL_11.08.2020 12-05-44_ACN-Puffer_50-50_15min.met
User: Irina Ihnatenko
Acquired: 11.08.2020 12:06:49
Printed: 11.08.2020 13:32:22
Sample ID: KuIna092_5µL
Injectionvolume: 5

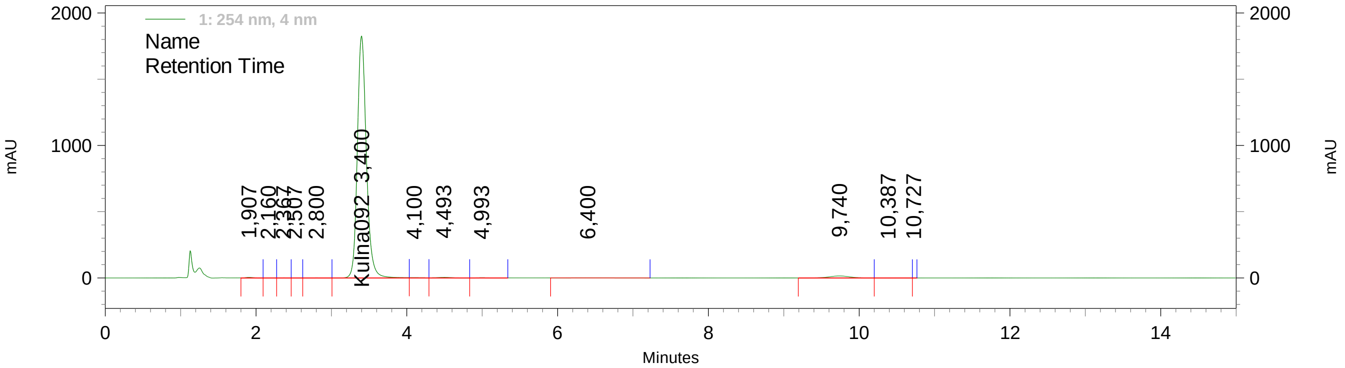

1: 254 nm, 4 nm

Results

Pk #	*Name*	*Retention Time*	*Area Percent*	*Area*
1		1,907	0,153	96068
2		2,160	0,020	12525
3		2,367	0,025	15572
4		2,507	0,012	7240
5		2,800	0,031	19189
6	**KuIna092**	3,400	97,074	60831123
7		4,100	0,155	97436
8		4,493	0,337	211365
9		4,993	0,094	59016
10		6,400	0,253	158394
11		9,740	1,790	1121925
12		10,387	0,054	33838
13		10,727	0,001	797
Totals			100,000	62664488

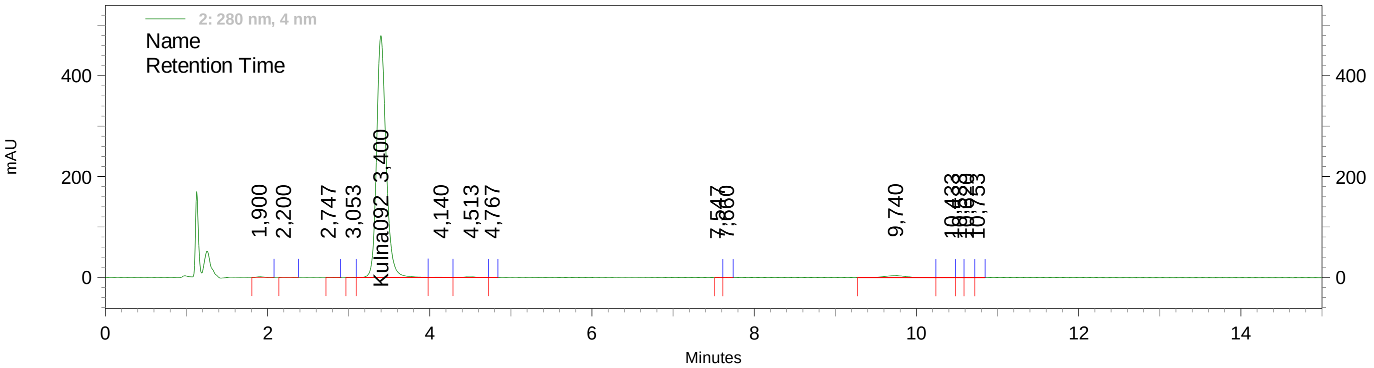

Method Name: **C:\EZChrom Elite\Enterprise\Projects\Reinheit_Irina\Method\ACN-H2O\ACN-H2O_10-90_10min.met**
Data: **C:\EZChrom Elite\Enterprise\Projects\Reinheit_Irina\Data\KuIna092_5µL_11.08.2020 12-05-44_ACN-Puffer_50-50_15min.met**
User: **Irina Ihnatenko**
Acquired: **11.08.2020 12:06:49**
Printed: **11.08.2020 13:32:22**
Sample ID: **KuIna092_5µL**
Injectionvolume: **5**

2: 280 nm, 4 nm

Results

Pk #	***Name***	***Retention Time***	***Area Percent***	***Area***
1		1,900	0,179	28705
2		2,200	0,059	9408
3		2,747	0,016	2600
4		3,053	0,011	1698
5	**KuIna092**	3,400	97,251	15578826
6		4,140	0,214	34234
7		4,513	0,351	56230
8		4,767	0,010	1558
9		7,547	0,005	818
10		7,660	0,009	1389
11		9,740	1,801	288543
12		10,433	0,043	6920
13		10,533	0,019	2992
14		10,620	0,018	2896
15		10,753	0,015	2380
Totals			100,000	16019197

Spectrum Report

Spectra of all named detected peaks

(The peak spectrum is defined as the peak apex spectrum)

Multi-Chrom 1 (1: 254 nm, 4 nm) Spectra

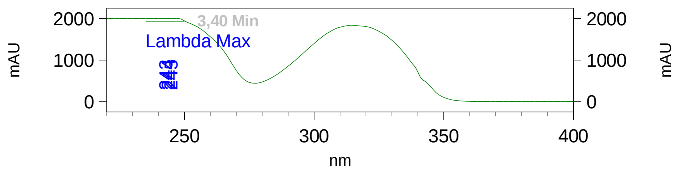

Retention time: 3,400 Min
Peak name: KuIna092
Lambda max: 245, 244, 243
Lambda min: 368, 277, 220

Multi-Chrom 2 (2: 280 nm, 4 nm) Spectra

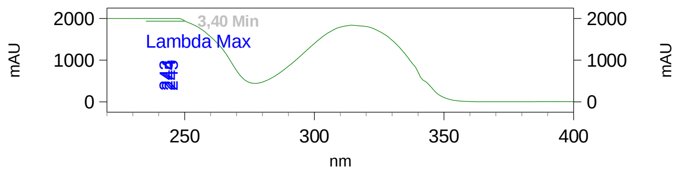

Retention time: 3,400 Min
Peak name: KuIna092
Lambda max: 245, 244, 243
Lambda min: 368, 277, 220

TU Braunschweig Institut für Pharmazeutische Chemie

Method Name: **C:\EZChrom Elite\Enterprise\Projects\Reinheit_Irina\Method\ACN-H2O\ACN-H2O_10-90_10min.met**
Data: **C:\EZChrom Elite\Enterprise\Projects\Reinheit_Irina\Data\KuIna092_5µL_11.08.2020 12-05-44_ACN-Puffer_50-50_15min.met**
User: **Irina Ihnatenko**
Acquired: **11.08.2020 12:06:49**
Printed: **11.08.2020 13:32:22**
Sample ID: **KuIna092_5µL**
Injectionvolume: **5**

C:\EZChrom Elite\Enterprise\Projects\Reinheit_Irina\Data\KuIna092_5L_11.08.20[illegible]

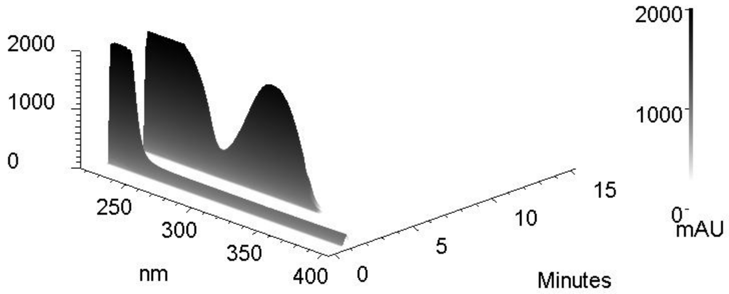

Supplement: S3 File — (ZIP) [file pone.0292946.s003.zip › S4_ZIP-File_HPLC_chromatograms/HPLC-Merck-cmpd-9b-iso-254+280nm.pdf]
