# Supplementary material for: The indole motif is essential for the antitrypanosomal activity of N5-substituted paullones
Source: PLoS One. 2023 Nov 30;18(11):e0292946. doi: 10.1371/journal.pone.0292946 (PMC10688702; doi:10.1371/journal.pone.0292946)

TU Braunschweig Institut für Pharmazeutische Chemie

Method Name: C:\EZChrom Elite\Enterprise\Projects\Reinheit_Irina\Method\ACN-H2O\ACN-H2O_10-90_10min.met
Data: C:\EZChrom Elite\Enterprise\Projects\Reinheit_Irina\Data\KuIna101_5µL_02.09.2020 13-30-00_ACN-Puffer_50-50_15min.met
User: Irina Ihnatenko
Acquired: 02.09.2020 13:31:06
Printed: 02.09.2020 14:51:09
Sample ID: KuIna101_5µL
Injectionvolume: 5

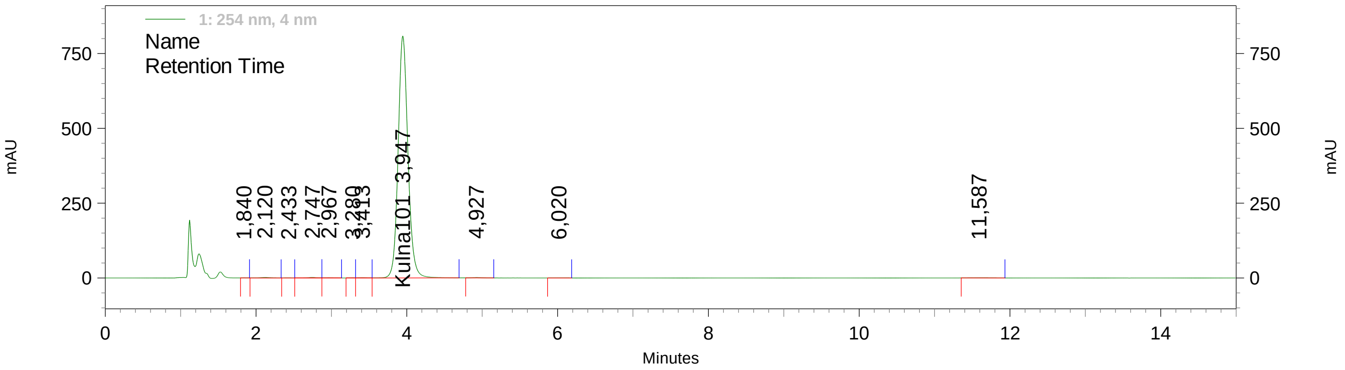

1: 254 nm. 4 nm

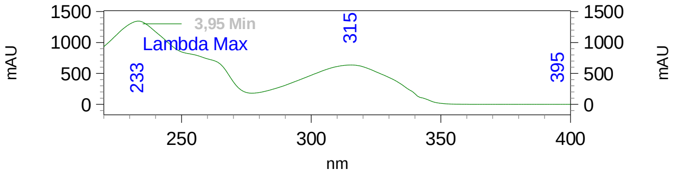

Pk #	*Name*	*Retention Time*	*Area Percent*	*Area*
1		1,840	0,010	2868
2		2,120	0,165	48694
3		2,433	0,007	2134
4		2,747	0,170	50196
5		2,967	0,060	17687
6		3,280	0,019	5548
7		3,413	0,059	17313
8	**KuIna101**	3,947	99,230	29317954
9		4,927	0,128	37770
10		6,020	0,025	7403
11		11,587	0,128	37776

Totals			100,000	29545343

TU Braunschweig Institut für Pharmazeutische Chemie

Method Name: C:\EZChrom Elite\Enterprise\Projects\Reinheit_Irina\Method\ACN-H2O\ACN-H2O_10-90_10min.met
Data: C:\EZChrom Elite\Enterprise\Projects\Reinheit_Irina\Data\KuIna101_5µL_02.09.2020 13-30-00_ACN-Puffer_50-50_15min.met
User: Irina Ihnatenko
Acquired: 02.09.2020 13:31:06
Printed: 02.09.2020 14:51:09
Sample ID: KuIna101_5µL
Injectionvolume: 5

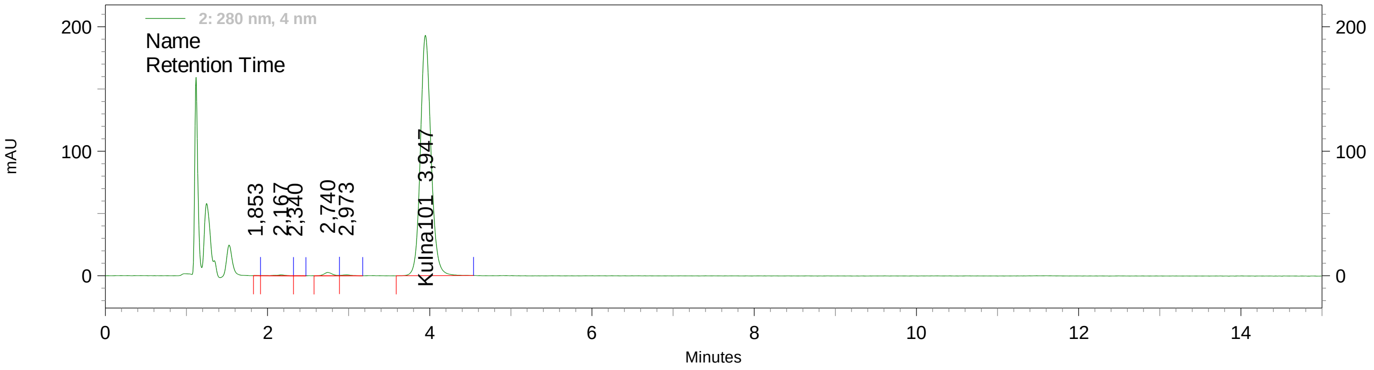

2: 280 nm, 4 nm
Results

Pk #	*Name*	*Retention Time*	*Area Percent*	*Area*
1		1,853	0,012	865
2		2,167	0,312	22268
3		2,340	0,035	2480
4		2,740	1,057	75506
5		2,973	0,326	23327
6	**KuIna101**	3,947	98,258	7020351
Totals			100,000	7144797

Spectrum Report

Spectra of all named detected peaks

(The peak spectrum is defined as the peak apex spectrum)

Multi-Chrom 1 (1: 254 nm, 4 nm) Spectra

Retention time: 3,947 Min
Peak name: KuIna101
Lambda max: 233, 315, 395
Lambda min: 381, 388, 370

Multi-Chrom 2 (2: 280 nm, 4 nm) Spectra

Retention time: 3,947 Min
Peak name: KuIna101
Lambda max: 233, 315, 395
Lambda min: 381, 388, 370

Method Name: **C:\EZChrom Elite\Enterprise\Projects\Reinheit_Irina\Method\ACN-H2O\ACN-H2O_10-90_10min.met**
Data: **C:\EZChrom Elite\Enterprise\Projects\Reinheit_Irina\Data\KuIna101_5µL_02.09.2020 13-30-00_ACN-Puffer_50-50_15min.met**
User: **Irina Ihnatenko**
Acquired: **02.09.2020 13:31:06**
Printed: **02.09.2020 14:51:09**
Sample ID: **KuIna101_5µL**
Injectionvolume: **5**

C:\EZChrom Elite\Enterprise\Projects\Reinheit_Irina\Data\KuIna101_5L_02.09.202

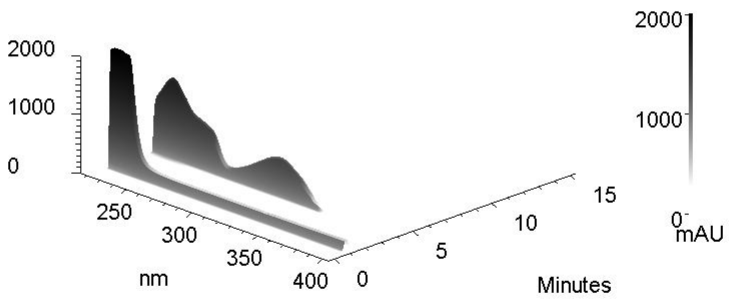

Supplement: S3 File — (ZIP) [file pone.0292946.s003.zip › S4_ZIP-File_HPLC_chromatograms/HPLC-Merck-cmpd-9c-iso-254+280nm.pdf]
