# Supplementary material for: The indole motif is essential for the antitrypanosomal activity of N5-substituted paullones
Source: PLoS One. 2023 Nov 30;18(11):e0292946. doi: 10.1371/journal.pone.0292946 (PMC10688702; doi:10.1371/journal.pone.0292946)
Supplement: S3 File — (ZIP) [file pone.0292946.s003.zip › S4_ZIP-File_HPLC_chromatograms/HPLC-VWR-cmpd-10a-grad-254nm.pdf]

TU Braunschweig Institut für Medizinische und Pharmazeutische Chemie

Analyzed Date and Time: 07.08.2020 10:12
Processed Date and Time: 07.08.2020 11:00
Reported Date and Time: 07.08.2020 11:00:38

Data Path: C:\HPLC-DATEN\Sandra Schweda\DATA\KuIna094 grad v2\
Processing Method: Gradient_ACN-H2O_10->90_25min

System (acquisition): AK Kunick HPLC 3
Series: KuIna094 grad v2
Application(data): Sandra Schweda
Vial Number: 11
Sample Name: KuIna094 gradient
Vial Type: UNK
Injection from this vial: 1 of 1
Volume: 3,0 ul
Sample Description:

Chrom Type: Fixed WL Chromatogram, 254 nm

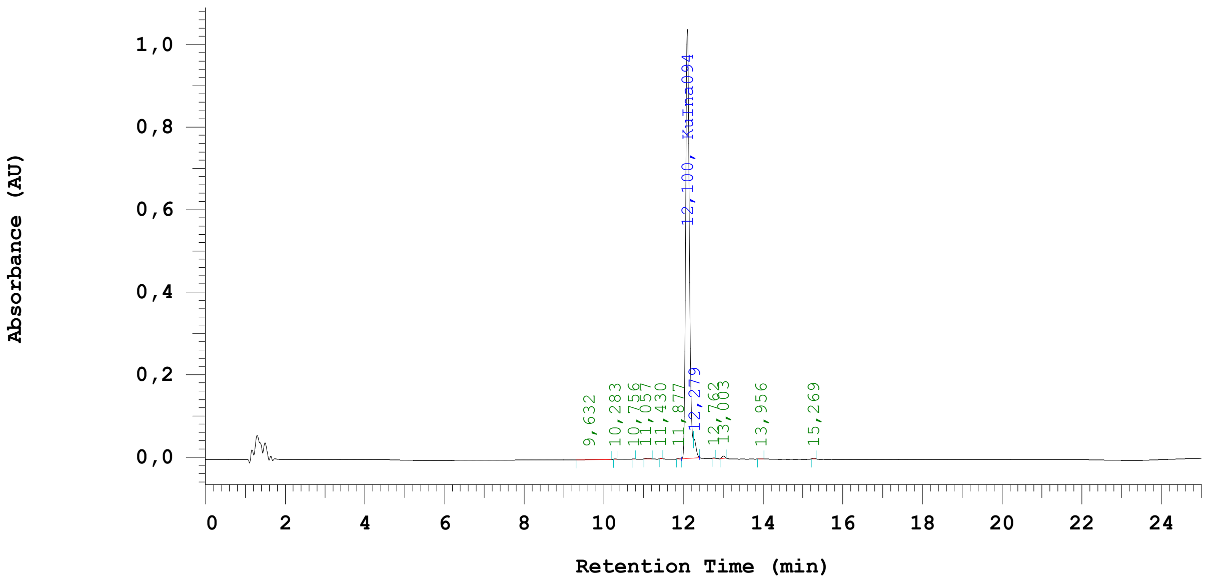

Processing Method: Gradient_ACN-H2O_10->90_25min
Method Developer: Mehmet Karatas
Pump 1: 5110
Pump 1 Solvent A:
Pump 1 Solvent B: ACN
Pump 1 Solvent C: ACN Gradient
Pump 1 Solvent D: H2O
Method Description:

Chrom Type: Fixed WL Chromatogram, 254 nm
Peak Quantitation: AREA
Calculation Method: EXT-STD

No.	Name	RT	Area	Area %	BC
1		9,632	3801	0,118	BB
2		10,283	1685	0,052	BB
3		10,756	1141	0,035	BB
4		11,057	4523	0,141	BB
5		11,430	2109	0,066	BB
6		11,877	1535	0,048	BB
7	KuIna094	12,100	3081201	95,714	MCd
8		12,279	103565	3,217	MCd
9		12,762	1541	0,048	BB
10		13,003	13252	0,412	BB
11		13,956	1165	0,036	BB
12		15,269	3644	0,113	BB

		3219162	100,000

Peak rejection level: 0
Note: (d) Result of Peak Deconvolution.

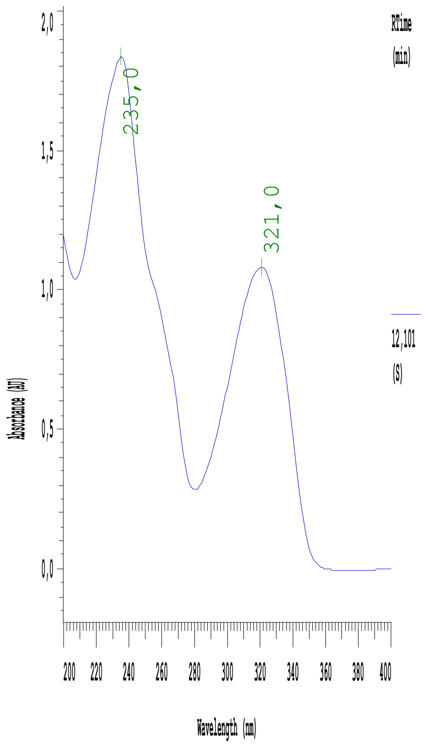

Peak Quantitation: AREA   Calculation Method: EXT-STD

Channel 1 Noise: Not Measured
Channel 1 Drift: Not Measured

Configuration parameters:
Interface: IFC   Gradient Mode: Low
Channel 1 Detector: 5430   Channel 2 Detector: None
Column Oven: 5310   Reaction Unit: None
Autosampler: 5260   Pump 1: 5110
Pump 2: None   Pump 3: None

Method Information:
Method Name: Gradient_ACN-H2O_10->90_25min
Developed by: Mehmet Karatas
Description:

Pump Setup:
Pump 1 Pressure Limit: 0 to 392 bar

Check Degassing Unit Status: YES

Pump 1 (5110):   Low Gradient Mode: LFM
Solvent A: Solvent B: ACN
Solvent C: ACN Gradient   Solvent D: H2O

Pump 1 (5110):
Pump Solvent and Event Table

Time (min)	%SolvA	%SolvB	%SolvC	%SolvD	Flow (mL/min)	Event 1	Event 2	Event 3	Event 4
0,0	0,0	10,0	0,0	90,0	1,000				
2,0	0,0	10,0	0,0	90,0					
12,0	0,0	90,0	0,0	10,0					
20,0	0,0	90,0	0,0	10,0					
23,0	0,0	10,0	0,0	90,0					

Autosampler Setup (5260):
ASP Syringe Speed: 3   DSP Syringe Speed: 3
Needle Down Speed: Fast   Syringe Volume: 175 uL
Air Volume: 2 uL   Rinse Port Wash Time: 1 s
Needle Wash before Injection: YES   Needle Wash Solvent: Solvent1
Needle Wash Time Solvent1: 15 s   Plunger Wash after Series Run: YES
Plunger Wash Time: 15 s   Injection Method: All
Feed Volume: 50 uL   Synchronize with a Pump(PASS): NO
Enable Vial Sensor: YES
Wash Solvent1 Name: H2O-Methanol 50:50
Wash Solvent2 Name: H2O   Check Degassing Unit Status: YES

Column Oven Setup (5310):
Temperature Upper Limit: 70 Centigrade
Tolerance(+/-): 1,0 Centigrade   Wait Time: 1 min

Option Valve: NO
Temperature Time Table

Time (min)	Temp (Centigrade)

0,0	40

Channel 1 Detector Setup (5430):

Slit Width: Coarse
Spectral Bandwidth: 4nm
Sampling Period: 50 ms
Wavelength Range: 200 to 400 nm
Monitoring Wavelength: 254 nm
Auto Zero before Injection: YES
Stop Time: 25,00 min
Response Time: 1,0 s
Lamp Mode: D2&W
Analog Signal Output: NO

Method DP for channel 1

Calculation Method:
Peak Quantitation: Area
Calculation Method: Ext Std
Peak identification Window: Abs Time
STD peaks identification rule: Highest peak
UNK peaks identification rule: Closest peak
Calibration order of curve fit: Linear - f(Response)
Force through zero: YES
Minimum number of calibration levels required: 1
Concentration Weight: 1,0
Update RT in component Table: NO
Do blank subtraction: NO
Do library search: NO

Component Table

RT (min)	Window (min)	Name	Func1	Func2	Func3
12,101	1,000	KuIna094			

RT (min)	Mol. Weight	Multi-plier	E-Conc	Tolerance (%)
12,101	0,000	1,000		

Concentration Table Data:
Dilution factor for STD1: 1,000 *
Concentration units: Other
Concentration Table:

Name	Std1
KuIna094	0,000000

Coefficients table

Name	A0	A1	A2	A3	Units	R-sqr
KuIna094	0,000E+00	0,000E+00	0,000E+00	0,000E+00		

Integration Table

Time (min)	Function	Value/Status

```
0,00    NOISE                   5
0,00    BUNCHING                OFF
0,00    SMOOTHING               OFF
0,00    SENSITIVITY             50
0,00    N-METHOD                0
0,00    INTEGRATION-INHIBIT     ON
2,00    INTEGRATION-INHIBIT     OFF
---------------------------------------------
```

DAD Processing Setup: Peak purity check enabled: YES
  Purity Threshold: 0,950
  Peak Height Percent for Side Spectra: 20 %
  Peak spectrum integration enabled: NO
  Chromatogram to create: Fixed at 254, 280 nm

DAD Display Format: Absorbance Scale: Auto
  Time range: 0,00 to 15,00 min   Wavelength range: 200 to 400 nm
  Offset: 0,0 %   Spectrum Display: Absorbance
  Auto Mark Peak WL: YES   Auto BG Subtraction: NO
  3-D resolution: Medium   3-D tilt: 50
  3-D rotation: 30   3-D mirror: NO
  Display spectra only: NO   Report Spectra: Peak top only.

Perform system suitability test : NO
Perform module performance test : NO
Perform data diagnosis : NO

Chromatogram Display Format: Autoscale: YES
  Autoscale Time Range: 0,00 to 600,00 min
  Use alternate scale: NO   Auto Zero: NO
  Scale to Full Chrom Time Range: YES   Peak rejection level: 0 uV * s
  Baseline overlay: YES   Peak start-end markers: YES
  Marker-In Signals: NO   Peak labels: Time, Name
  Show integration time table: NO   Show gradient curves: NO
  Picture in picture: None
  Report channel 1 labels in the chromatogram overlay graph.
  Multi-injection graph offsets----All: 25, All STDs: 25, All UNKs: 25.

Report Format: Reported peaks: All Peaks
  Name of quantified unknown peaks: Coefficient: Response (A)
  Vial summary average type: Mean
  Report statistics on repetitive injections retention times: NO
  Report statistics on repetitive injections concentrations: NO
  Report statistics on unknown vials retentions times: NO
  Report statistics on unknown vials concentrations: NO
  Use primary layout: YES   Use secondary layout: NO
  Print primary layout report: NO   Print secondary layout report: NO
  Acquisition DDE: NO   Acquisition macro name:
  Reprocess DDE: NO   Reprocess macro name:
  Concentration 1 Unit: Other   Concentration 1 name:
  Concentration 1 Factor: 1,000
  Concentration 1 divide by sample amount: NO
  Concentration 2 Unit: Other   Concentration 2 name:
  Concentration 2 Factor: 1,000
  Concentration 2 use component multiplier: NO
  Injection report column 1 header: PK-NUM
  Injection report column 2 header: NAME
  Injection report column 3 header: RT
  Injection report column 4 header: AREA
  Injection report column 5 header: AREA%
  Injection report column 6 header: BC
