# Supplementary material for: The indole motif is essential for the antitrypanosomal activity of N5-substituted paullones
Source: PLoS One. 2023 Nov 30;18(11):e0292946. doi: 10.1371/journal.pone.0292946 (PMC10688702; doi:10.1371/journal.pone.0292946)
Supplement: S3 File — (ZIP) [file pone.0292946.s003.zip › S4_ZIP-File_HPLC_chromatograms/HPLC-VWR-cmpd-10a-iso-280nm.pdf]

TU Braunschweig Institut für Medizinische und Pharmazeutische Chemie

Analyzed Date and Time: 07.08.2020 12:29
Reported Date and Time: 07.08.2020 13:02:57
Processed Date and Time: 07.08.2020 13:02
Data Path: C:\HPLC-DATEN\Sandra Schweda\DATA\1657\
Processing Method: Gradient_ACN-H2O_10->90_25min

System (acquisition): AK Kunick HPLC 3
Series: 1657
Application(data): Sandra Schweda
Vial Number: 11
Sample Name: KuIna094 isokrat
Vial Type: UNK
Injection from this vial: 1 of 1
Volume: 10,0 ul
Sample Description:

Chrom Type: Fixed WL Chromatogram, 280 nm

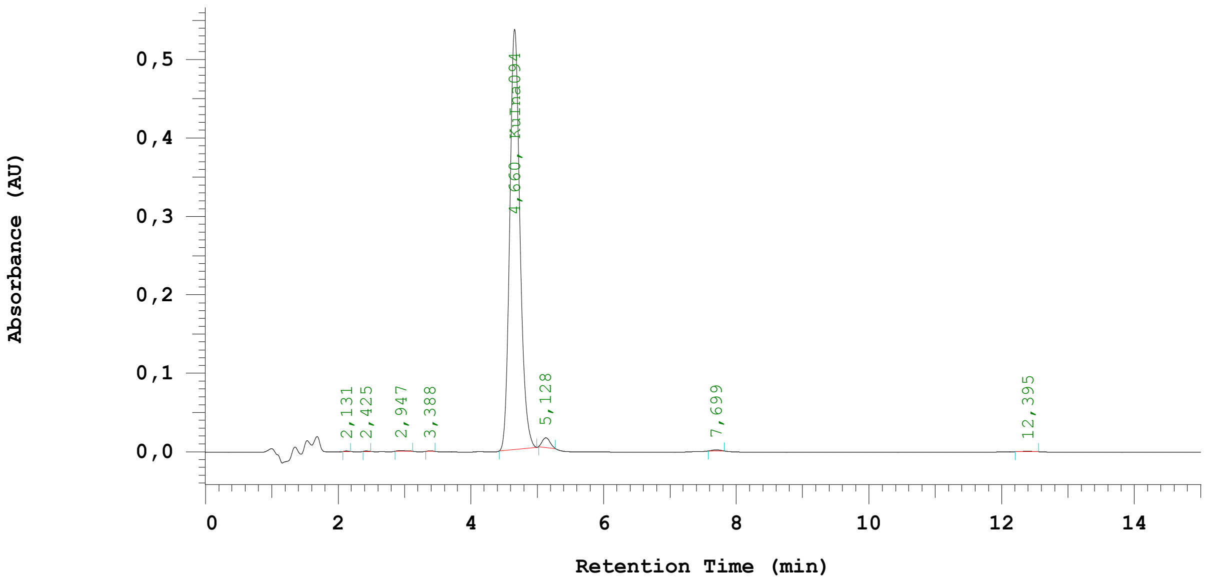

Processing Method: Gradient_ACN-H2O_10->90_25min
Method Developer: Mehmet Karatas
Pump 1: 5110
Pump 1 Solvent A:
Pump 1 Solvent B: ACN
Pump 1 Solvent C:
Pump 1 Solvent D: H2O
Method Description:

Chrom Type: Fixed WL Chromatogram, 280 nm
Peak Quantitation: AREA
Calculation Method: EXT-STD

No.	Name	RT	Area	Area %	BC
1		2,131	1373	0,048	BB
2		2,425	1225	0,043	BB
3		2,947	4268	0,149	BB
4		3,388	1757	0,062	BB
5	KuIna094	4,660	2789758	97,696	BB
6		5,128	47856	1,676	BB
7		7,699	6072	0,213	BB
8		12,395	3236	0,113	BB
			2855545	100,000	

Peak rejection level: 0

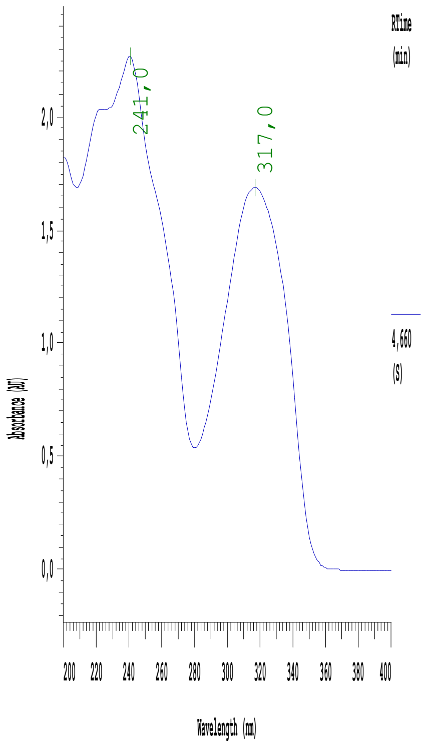

Peak Quantitation: AREA Calculation Method: EXT-STD

Channel 1 Noise: Not Measured
Channel 1 Drift: Not Measured

Configuration parameters:
Interface: IFC   Gradient Mode: Low
Channel 1 Detector: 5430   Channel 2 Detector: None
Column Oven: 5310   Reaction Unit: None
Autosampler: 5260   Pump 1: 5110
Pump 2: None   Pump 3: None

Method Information:
Method Name: ACN-H2O_60-40_15 min   Developed by: Mehmet Karatas
Description:

Pump Setup:
Pump 1 Pressure Limit: 0 to 392 bar

Check Degassing Unit Status: YES

Pump 1 (5110):   Low Gradient Mode: LFM
Solvent A: Solvent B: ACN
Solvent C: Solvent D: H2O

Pump 1 (5110):
Pump Solvent and Event Table

Time (min)	%SolvA	%SolvB	%SolvC	%SolvD	Flow (mL/min)	Event 1	Event 2	Event 3	Event 4
0,0	0,0	60,0	0,0	40,0	1,000				

Autosampler Setup (5260):
ASP Syringe Speed: 3   DSP Syringe Speed: 3
Needle Down Speed: Fast   Syringe Volume: 175 uL
Air Volume: 2 uL   Rinse Port Wash Time: 1 s
Needle Wash before Injection: YES   Needle Wash Solvent: Solvent1
Needle Wash Time Solvent1: 15 s   Plunger Wash after Series Run: YES
Plunger Wash Time: 15 s   Injection Method: All
Feed Volume: 50 uL   Synchronize with a Pump(PASS): NO
Enable Vial Sensor: YES
Wash Solvent1 Name: H2O-Methanol 50:50
Wash Solvent2 Name: H2O   Check Degassing Unit Status: YES

Column Oven Setup (5310):
Temperature Upper Limit: 70 Centigrade
Tolerance(+/-): 1,0 Centigrade   Wait Time: 1 min

Option Valve: NO
Temperature Time Table

Time (min)	Temp (Centigrade)
0,0	40

Channel 1 Detector Setup (5430):
Slit Width: Coarse | Spectral Bandwidth: 4nm
Sampling Period: 50 ms | Wavelength Range: 200 to 400 nm
Monitoring Wavelength: 254 nm | Auto Zero before Injection: YES
Stop Time: 15,00 min | Response Time: 1,0 s
Lamp Mode: D2&W | Analog Signal Output: NO

Method DP for channel 1

Calculation Method: | Peak Quantitation: Area
Calculation Method: Ext Std | Peak identification Window: Abs Time
STD peaks identification rule: Highest peak
UNK peaks identification rule: Closest peak
Calibration order of curve fit: Linear - f(Response)
Force through zero: YES
Minimum number of calibration levels required: 1
Concentration Weight: 1,0 | Update RT in component Table: NO
Do blank subtraction: NO | Do library search: NO

Component Table

RT (min)	Window (min)	Name	Func1	Func2	Func3	Mol. Weight	Multi- plier	E-Conc	Tolerance (%)
4,660	1,000	KuIna094				0,000	1,000		

Concentration Table Data: | Dilution factor for STD1: 1,000 *
Concentration units: Other
Concentration Table:

Name	Std1
KuIna094	0,000000

Coefficients table

Name	A0	A1	A2	A3	Units	R-sqr
KuIna094	0,000E+00	0,000E+00	0,000E+00	0,000E+00		

Integration Table

Time (min)	Function	Value/Status
0,00	NOISE	5
0,00	BUNCHING	OFF
0,00	SMOOTHING	OFF
0,00	SENSITIVITY	50
0,00	N-METHOD	0
0,00	INTEGRATION-INHIBIT	ON
2,00	INTEGRATION-INHIBIT	OFF

DAD Processing Setup: | Peak purity check enabled: YES

Purity Threshold: 0,950
Peak Height Percent for Side Spectra: 20 %
Peak spectrum integration enabled: NO
Chromatogram to create: Fixed at 254, 280 nm

DAD Display Format:
Time range: 0,00 to 15,00 min
Offset: 0,0 %
Auto Mark Peak WL: YES
3-D resolution: Medium
3-D rotation: 30
Display spectra only: NO

Absorbance Scale: Auto
Wavelength range: 200 to 400 nm
Spectrum Display: Absorbance
Auto BG Subtraction: NO
3-D tilt: 50
3-D mirror: NO
Report Spectra: Peak top only.

Perform system suitability test : NO
Perform module performance test : NO
Perform data diagnosis : NO

Chromatogram Display Format:
Autoscale Time Range: 0,00 to 600,00 min
Use alternate scale: NO
Scale to Full Chrom Time Range: YES
Baseline overlay: YES
Marker-In Signals: NO
Show integration time table: NO
Picture in picture: None
Report channel 1 labels in the chromatogram overlay graph.
Multi-injection graph offsets----All: 25, All STDs: 25, All UNKs: 25.

Autoscale: YES
Auto Zero: NO
Peak rejection level: 0 uV * s
Peak start-end markers: YES
Peak labels: Time, Name
Show gradient curves: NO

Report Format:
Name of quantified unknown peaks:
Vial summary average type: Mean
Report statistics on repetitive injections retention times: NO
Report statistics on repetitive injections concentrations: NO
Report statistics on unknown vials retentions times: NO
Report statistics on unknown vials concentrations: NO
Use primary layout: YES
Print primary layout report: NO
Acquisition DDE: NO
Reprocess DDE: NO
Concentration 1 Unit: Other
Concentration 1 Factor: 1,000
Concentration 1 divide by sample amount: NO
Concentration 2 Unit: Other
Concentration 2 Factor: 1,000
Concentration 2 use component multiplier: NO
Injection report column 1 header: PK-NUM
Injection report column 2 header: NAME
Injection report column 3 header: RT
Injection report column 4 header: AREA
Injection report column 5 header: AREA%
Injection report column 6 header: BC

Reported peaks: All Peaks
Coefficient: Response (A)
Use secondary layout: NO
Print secondary layout report: NO
Acquisition macro name:
Reprocess macro name:
Concentration 1 name:
Concentration 2 name:
