# Supplementary material for: The indole motif is essential for the antitrypanosomal activity of N5-substituted paullones
Source: PLoS One. 2023 Nov 30;18(11):e0292946. doi: 10.1371/journal.pone.0292946 (PMC10688702; doi:10.1371/journal.pone.0292946)
Supplement: S3 File — (ZIP) [file pone.0292946.s003.zip › S4_ZIP-File_HPLC_chromatograms/HPLC-VWR-cmpd-2j-iso-254nm.pdf]

TU Braunschweig Institut für Medizinische und Pharmazeutische Chemie

Analyzed Date and Time: 13.08.2019 15:55
Reported Date and Time: 13.08.2019 17:17:17
Processed Date and Time: 13.08.2019 17:17
Data Path: C:\HPLC-DATEN\Irina Ihnatenko\DATA\KuIna046 isokrat\
Processing Method: Gradient_ACN-H2O_10->90_25min

System (acquisition): AK Kunick HPLC 3
Series: KuIna046 isokrat
Application(data): Irina Ihnatenko
Vial Number: 52
Sample Name: KuIna046 isokrat
Vial Type: UNK
Injection from this vial: 1 of 1
Volume: 3,0 ul
Sample Description:

Chrom Type: Fixed WL Chromatogram, 254 nm

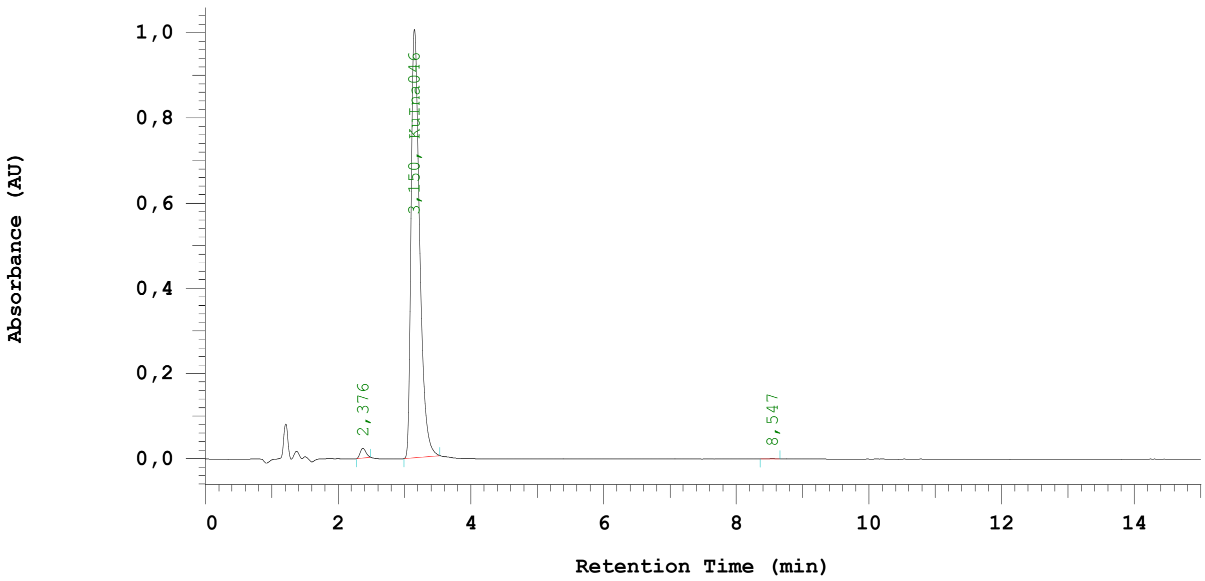

Processing Method: Gradient_ACN-H2O_10->90_25min
Method Developer: Mehmet Karatas
Pump 1: 5110
Pump 1 Solvent A:
Pump 1 Solvent B: ACN
Pump 1 Solvent C:
Pump 1 Solvent D: H2O
Method Description:

Chrom Type: Fixed WL Chromatogram, 254 nm
Peak Quantitation: AREA
Calculation Method: EXT-STD

No.	Name	RT	Area	Area %	BC
1		2,376	67292	1,514	BB
2	KuIna046	3,150	4376916	98,451	BB
3		8,547	1571	0,035	BB
			4445779	100,000	

Peak rejection level: 0

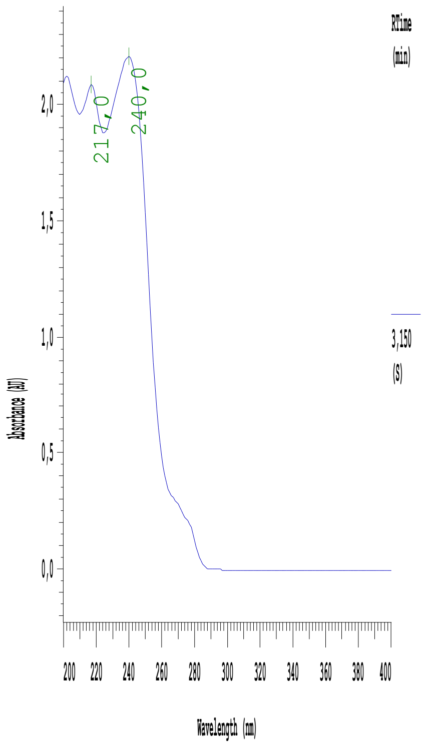

Peak Quantitation: AREA   Calculation Method: EXT-STD

Channel 1 Noise: Not Measured
Channel 1 Drift: Not Measured

Configuration parameters:
Interface: IFC
Gradient Mode: Low
Channel 1 Detector: 5430
Channel 2 Detector: None
Column Oven: 5310
Reaction Unit: None
Autosampler: 5260
Pump 1: 5110
Pump 2: None
Pump 3: None

Method Information:
Method Name: ACN-H2O_40-60_15 min
Developed by: Mehmet Karatas
Description:

Pump Setup:
Pump 1 Pressure Limit: 0 to 392 bar

Check Degassing Unit Status: YES

Pump 1 (5110):
Low Gradient Mode: LFM
Solvent A:
Solvent B: ACN
Solvent C:
Solvent D: H2O

Pump 1 (5110):
Pump Solvent and Event Table

Time (min)	%SolvA	%SolvB	%SolvC	%SolvD	Flow (mL/min)	Event 1	Event 2	Event 3	Event 4
0,0	0,0	40,0	0,0	60,0	1,000				

Autosampler Setup (5260):
ASP Syringe Speed: 3
DSP Syringe Speed: 3
Needle Down Speed: Fast
Syringe Volume: 175 uL
Air Volume: 2 uL
Rinse Port Wash Time: 1 s
Needle Wash before Injection: YES
Needle Wash Solvent: Solvent1
Needle Wash Time Solvent1: 15 s
Plunger Wash after Series Run: YES
Plunger Wash Time: 15 s
Injection Method: All
Feed Volume: 50 uL
Synchronize with a Pump(PASS): NO
Enable Vial Sensor: YES
Wash Solvent1 Name: H2O-Methanol 50:50
Wash Solvent2 Name: H2O
Check Degassing Unit Status: YES

Column Oven Setup (5310):
Temperature Upper Limit: 70 Centigrade
Tolerance(+/-): 1,0 Centigrade
Wait Time: 1 min

Option Valve: NO
Temperature Time Table

Time (min)	Temp (Centigrade)
0,0	40

Channel 1 Detector Setup (5430):
Slit Width: Coarse
Sampling Period: 50 ms
Monitoring Wavelength: 254 nm
Stop Time: 15,00 min
Lamp Mode: D2&W
Spectral Bandwidth: 4nm
Wavelength Range: 200 to 400 nm
Auto Zero before Injection: YES
Response Time: 1,0 s
Analog Signal Output: NO

Method DP for channel 1

Calculation Method:
Calculation Method: Ext Std
STD peaks identification rule: Highest peak
UNK peaks identification rule: Closest peak
Calibration order of curve fit: Linear - f(Response)
Force through zero: YES
Minimum number of calibration levels required: 1
Concentration Weight: 1,0
Do blank subtraction: NO
Peak Quantitation: Area
Peak identification Window: Abs Time
Update RT in component Table: NO
Do library search: NO

Component Table

RT (min)	Window (min)	Name	Func1	Func2	Func3
3,150	1,000	KuIna046			

RT (min)	Mol. Weight	Multi-plier	E-Conc	Tolerance (%)
3,150	272,350	1,000		

Concentration Table Data:
Concentration units: Other
Concentration Table:
Dilution factor for STD1: 1,000 *

Name	Std1
KuIna046	0,000000

Coefficients table

Name	A0	A1	A2	A3	Units	R-sqr
KuIna046	0,000E+00	0,000E+00	0,000E+00	0,000E+00		

Integration Table

Time (min)	Function	Value/Status
0,00	NOISE	5
0,00	BUNCHING	OFF
0,00	SMOOTHING	OFF
0,00	SENSITIVITY	50
0,00	N-METHOD	0

```
0,00    INTEGRATION-INHIBIT   ON
2,00    INTEGRATION-INHIBIT   OFF
---------------------------------------------


DAD Processing Setup:                       Peak purity check enabled: YES
  Purity Threshold: 0,950
  Peak Height Percent for Side Spectra: 20 %
  Peak spectrum integration enabled: NO
  Chromatogram to create: Fixed at 254, 280 nm

DAD Display Format:                         Absorbance Scale: Auto
  Time range: 0,00 to 15,00 min             Wavelength range: 200 to 400 nm
  Offset: 0,0 %                             Spectrum Display: Absorbance
  Auto Mark Peak WL: YES                    Auto BG Subtraction: NO
  3-D resolution: Medium                    3-D tilt: 50
  3-D rotation: 30                          3-D mirror: NO
  Display spectra only: NO                  Report Spectra: Peak top only.

Perform system suitability test     : NO
Perform module performance test     : NO
Perform data diagnosis              : NO

Chromatogram Display Format:                Autoscale: YES
  Autoscale Time Range: 0,00 to 600,00 min
  Use alternate scale: NO                   Auto Zero: NO
  Scale to Full Chrom Time Range: YES       Peak rejection level: 0 uV * s
  Baseline overlay: YES                     Peak start-end markers: YES
  Marker-In Signals: NO                     Peak labels: Time, Name
  Show integration time table: NO           Show gradient curves: NO
  Picture in picture:  None
  Report channel 1 labels in the chromatogram overlay graph.
  Multi-injection graph offsets----All: 25, All STDs: 25, All UNKs: 25.

Report Format:                              Reported peaks: All Peaks
  Name of quantified unknown peaks:         Coefficient: Response (A)
  Vial summary average type: Mean
  Report statistics on repetitive injections retention times: NO
  Report statistics on repetitive injections concentrations: NO
  Report statistics on unknown vials retentions times: NO
  Report statistics on unknown vials concentrations: NO
  Use primary layout: YES                   Use secondary layout: NO
  Print primary layout report: NO           Print secondary layout report: NO
  Acquisition DDE: NO                       Acquisition macro name:
  Reprocess DDE: NO                         Reprocess macro name:
  Concentration 1 Unit: Other               Concentration 1 name:
  Concentration 1 Factor: 1,000
  Concentration 1 divide by sample amount: NO
  Concentration 2 Unit: Other               Concentration 2 name:
  Concentration 2 Factor: 1,000
  Concentration 2 use component multiplier: NO
  Injection report column 1 header: PK-NUM
  Injection report column 2 header: NAME
  Injection report column 3 header: RT
  Injection report column 4 header: AREA
  Injection report column 5 header: AREA%
  Injection report column 6 header: BC
```
