# Supplementary material for: The indole motif is essential for the antitrypanosomal activity of N5-substituted paullones
Source: PLoS One. 2023 Nov 30;18(11):e0292946. doi: 10.1371/journal.pone.0292946 (PMC10688702; doi:10.1371/journal.pone.0292946)
Supplement: S3 File — (ZIP) [file pone.0292946.s003.zip › S4_ZIP-File_HPLC_chromatograms/HPLC-VWR-cmpd-4c-grad-254nm.pdf]

TU Braunschweig Institut für Medizinische und Pharmazeutische Chemie

Analyzed Date and Time: 19.02.2020 11:09   Reported Date and Time: 19.02.2020 12:46:54
Processed Date and Time: 19.02.2020 12:46
Data Path: C:\HPLC-DATEN\Sandra Schweda\DATA\0054\
Processing Method: Gradient_ACN-H2O_10->90_25min
System (acquisition): AK Kunick HPLC 3   Series: 0054
Application(data): Sandra Schweda   Vial Number: 21
Sample Name: KuIna069 gradient   Vial Type: UNK
Injection from this vial: 1 of 1   Volume: 10,0 ul
Sample Description:

Chrom Type: Fixed WL Chromatogram, 254 nm

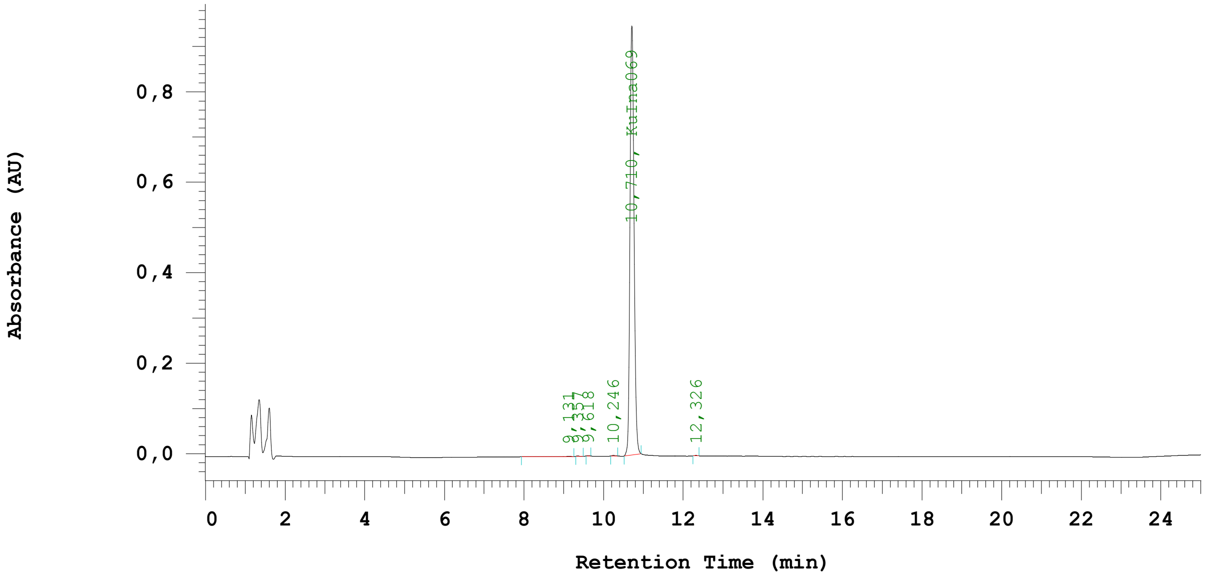

Processing Method: Gradient_ACN-H2O_10->90_25min
Method Developer: Mehmet Karatas
Pump 1: 5110
Pump 1 Solvent A: Pump 1 Solvent B: ACN
Pump 1 Solvent C: ACN Gradient   Pump 1 Solvent D: H2O
Method Description:

Chrom Type: Fixed WL Chromatogram, 254 nm
Peak Quantitation: AREA
Calculation Method: EXT-STD

No.	Name	RT	Area	Area %	BC
1		9,131	6776	0,214	BB
2		9,357	1350	0,043	BB
3		9,618	2031	0,064	BB
4		10,246	3521	0,111	BB
5	KuIna069	10,710	3150178	99,502	BB
6		12,326	2091	0,066	BB
			3165947	100,000	

Peak rejection level: 0

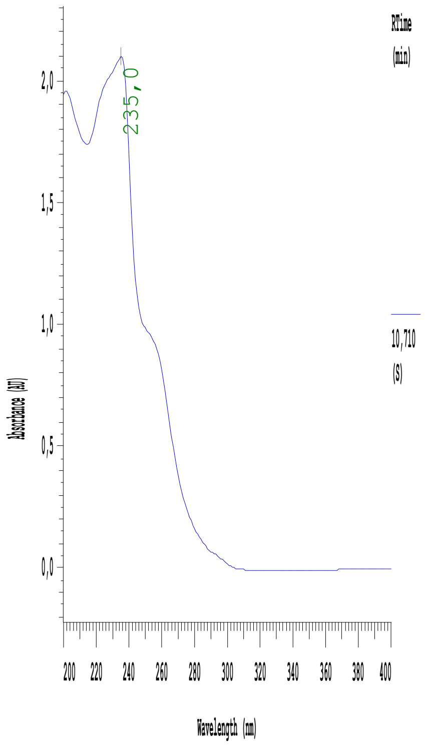

Peak Quantitation: AREA Calculation Method: EXT-STD

Channel 1 Noise: Not Measured
Channel 1 Drift: Not Measured

Configuration parameters:
  Interface: IFC   Gradient Mode: Low
  Channel 1 Detector: 5430   Channel 2 Detector: None
  Column Oven: 5310   Reaction Unit: None
  Autosampler: 5260   Pump 1: 5110
  Pump 2: None   Pump 3: None

Method Information:
  Method Name: Gradient_ACN-H2O_10->90_25min
  Developed by: Mehmet Karatas
  Description:

Pump Setup:
  Pump 1 Pressure Limit: 0 to 392 bar

  Check Degassing Unit Status: YES

Pump 1 (5110):   Low Gradient Mode: LFM
  Solvent A: Solvent B: ACN
  Solvent C: ACN Gradient   Solvent D: H2O

Pump 1 (5110):
  Pump Solvent and Event Table

Time (min)	%SolvA	%SolvB	%SolvC	%SolvD	Flow (mL/min)	Event 1	Event 2	Event 3	Event 4
0,0	0,0	10,0	0,0	90,0	1,000				
2,0	0,0	10,0	0,0	90,0					
12,0	0,0	90,0	0,0	10,0					
20,0	0,0	90,0	0,0	10,0					
23,0	0,0	10,0	0,0	90,0					

Autosampler Setup (5260):
  ASP Syringe Speed: 3   DSP Syringe Speed: 3
  Needle Down Speed: Fast   Syringe Volume: 175 uL
  Air Volume: 2 uL   Rinse Port Wash Time: 1 s
  Needle Wash before Injection: YES   Needle Wash Solvent: Solvent1
  Needle Wash Time Solvent1: 15 s   Plunger Wash after Series Run: YES
  Plunger Wash Time: 15 s   Injection Method: All
  Feed Volume: 50 uL   Synchronize with a Pump(PASS): NO
  Enable Vial Sensor: YES
  Wash Solvent1 Name: H2O-Methanol 50:50
  Wash Solvent2 Name: H2O   Check Degassing Unit Status: YES

Column Oven Setup (5310):
  Temperature Upper Limit: 70 Centigrade
  Tolerance(+/-): 1,0 Centigrade   Wait Time: 1 min

  Option Valve: NO
  Temperature Time Table

Time (min)	Temp (Centigrade)

0,0	40

Channel 1 Detector Setup (5430):
- Slit Width: Coarse
- Sampling Period: 50 ms
- Monitoring Wavelength: 254 nm
- Stop Time: 25,00 min
- Lamp Mode: D2&W
- Spectral Bandwidth: 4nm
- Wavelength Range: 200 to 400 nm
- Auto Zero before Injection: YES
- Response Time: 1,0 s
- Analog Signal Output: NO

Method DP for channel 1

Calculation Method:
- Calculation Method: Ext Std
- STD peaks identification rule: Highest peak
- UNK peaks identification rule: Closest peak
- Calibration order of curve fit: Linear - f(Response)
- Force through zero: YES
- Minimum number of calibration levels required: 1
- Concentration Weight: 1,0
- Do blank subtraction: NO

Peak Quantitation: Area
Peak identification Window: Abs Time
Update RT in component Table: NO
Do library search: NO

Component Table

RT (min)	Window (min)	Name	Func1	Func2	Func3
10,710	1,000	KuIna069			

RT (min)	Mol. Weight	Multi- plier	E-Conc	Tolerance (%)
10,710	423,510	1,000		

Concentration Table Data:
- Concentration units: Other
- Concentration Table:

Dilution factor for STD1: 1,000 *

Name	Std1
KuIna069	0,000000

Coefficients table

Name	A0	A1	A2	A3	Units	R-sqr
KuIna069	0,000E+00	0,000E+00	0,000E+00	0,000E+00		

Integration Table

Time (min)	Function	Value/Status

```
0,00    NOISE                   5
0,00    BUNCHING                OFF
0,00    SMOOTHING               OFF
0,00    SENSITIVITY             50
0,00    N-METHOD                0
0,00    INTEGRATION-INHIBIT     ON
2,00    INTEGRATION-INHIBIT     OFF
---------------------------------------------
```

DAD Processing Setup: Peak purity check enabled: YES
Purity Threshold: 0,950
Peak Height Percent for Side Spectra: 20 %
Peak spectrum integration enabled: NO
Chromatogram to create: Fixed at 254, 280 nm

DAD Display Format: Absorbance Scale: Auto
Time range: 0,00 to 15,00 min Wavelength range: 200 to 400 nm
Offset: 0,0 % Spectrum Display: Absorbance
Auto Mark Peak WL: YES Auto BG Subtraction: NO
3-D resolution: Medium 3-D tilt: 50
3-D rotation: 30 3-D mirror: NO
Display spectra only: NO Report Spectra: Peak top only.

Perform system suitability test : NO
Perform module performance test : NO
Perform data diagnosis : NO

Chromatogram Display Format: Autoscale: YES
Autoscale Time Range: 0,00 to 600,00 min
Use alternate scale: NO Auto Zero: NO
Scale to Full Chrom Time Range: YES Peak rejection level: 0 uV * s
Baseline overlay: YES Peak start-end markers: YES
Marker-In Signals: NO Peak labels: Time, Name
Show integration time table: NO Show gradient curves: NO
Picture in picture: None
Report channel 1 labels in the chromatogram overlay graph.
Multi-injection graph offsets----All: 25, All STDs: 25, All UNKs: 25.

Report Format: Reported peaks: All Peaks
Name of quantified unknown peaks: Coefficient: Response (A)
Vial summary average type: Mean
Report statistics on repetitive injections retention times: NO
Report statistics on repetitive injections concentrations: NO
Report statistics on unknown vials retentions times: NO
Report statistics on unknown vials concentrations: NO
Use primary layout: YES Use secondary layout: NO
Print primary layout report: NO Print secondary layout report: NO
Acquisition DDE: NO Acquisition macro name:
Reprocess DDE: NO Reprocess macro name:
Concentration 1 Unit: Other Concentration 1 name:
Concentration 1 Factor: 1,000
Concentration 1 divide by sample amount: NO
Concentration 2 Unit: Other Concentration 2 name:
Concentration 2 Factor: 1,000
Concentration 2 use component multiplier: NO
Injection report column 1 header: PK-NUM
Injection report column 2 header: NAME
Injection report column 3 header: RT
Injection report column 4 header: AREA
Injection report column 5 header: AREA%
Injection report column 6 header: BC
