# Supplementary material for: The indole motif is essential for the antitrypanosomal activity of N5-substituted paullones
Source: PLoS One. 2023 Nov 30;18(11):e0292946. doi: 10.1371/journal.pone.0292946 (PMC10688702; doi:10.1371/journal.pone.0292946)
Supplement: S1 Table — (PDF) [file pone.0292946.s006.pdf]

# The indole motif is essential for the antitrypanosomal activity of N[5]-substituted paullones

Irina Ihnatenko, Marco J. Müller, Oliver C. F. Orban, Jens C. Lindhof, Diego Benítez, Cecilia Ortíz, Estefanía Dibello, Leonardo L. Seidl, Marcelo A. Comini and Conrad Kunick*

**S2 Table. Physicochemical properties, water solubility and pharmacokinetics predicted data for benzazepinones and paullones tested in biological assays.**

| #cmpd | Molecular weight | Fraction Csp3 | #Rotatable bonds | #H-bond acceptors | #H-bond donors | TPSA | Consensus Log P[2] | ESOL Log S[3] | Ali Log S[3] | Silicos-IT LogSw[3] | GI absorption | BBB permeant | Pgp substrate | Lipinski #violations | Bioavailability Score | PAINS #alerts | Leadlikeness #violations |
|---|---|---|---|---|---|---|---|---|---|---|---|---|---|---|---|---|---|
| **1f** | 522.77 | 0.24 | 6 | 3 | 3 | 77.23 | 3.19 | -4.92 | -4.22 | -8.59 | High | Yes | Yes | 1 | 0.55 | 0 | 1 |
| **1g** | 431.32 | 0.24 | 6 | 3 | 3 | 77.23 | 3.05 | -4.33 | -4.19 | -8.36 | High | Yes | Yes | 0 | 0.55 | 0 | 1 |
| **1h** | 464.87 | 0.27 | 7 | 6 | 3 | 77.23 | 3.55 | -4.60 | -4.46 | -8.59 | High | No | Yes | 0 | 0.55 | 0 | 1 |
| **2a**[1] | 275.35 | 0.47 | 6 | 3 | 2 | 61.44 | 1.20 | -1.70 | -1.38 | -4.38 | High | No | Yes | 0 | 0.55 | 0 | 0 |
| **2b** | 430.54 | 0.61 | 9 | 5 | 1 | 82.19 | 1.95 | -3.10 | -2.99 | -4.52 | High | No | Yes | 0 | 0.55 | 0 | 2 |
| **2c** | 361.44 | 0.53 | 9 | 4 | 2 | 87.74 | 1.88 | -2.70 | -3.11 | -4.85 | High | No | Yes | 0 | 0.55 | 0 | 2 |
| **2d** | 387.47 | 0.57 | 6 | 4 | 0 | 70.16 | 1.98 | -3.13 | -2.88 | -3.70 | High | No | Yes | 0 | 0.55 | 0 | 1 |
| **2e** | 375.46 | 0.55 | 9 | 4 |  | 78.95 | 2.15 | -2.90 | -3.12 | -4.51 | High | No | Yes | 0 | 0.55 | 0 | 2 |
| **2f** | 389.49 | 0.57 | 11 | 4 | 2 | 87.74 | 2.64 | -3.18 | -3.85 | -5.64 | High | No | Yes | 0 | 0.55 | 0 | 2 |
| **2g** | 401.50 | 0.59 | 7 | 4 | 1 | 78.95 | 2.32 | -3.53 | -3.69 | -4.45 | High | No | Yes | 0 | 0.55 | 0 | 1 |
| **2h** | 288.34 | 0.50 | 3 | 3 | 0 | 49.85 | 1.37 | -2.12 | -1.39 | -3.27 | High | No | Yes | 0 | 0.55 | 0 | 0 |
| **2i** | 286.37 | 0.53 | 3 | 2 | 0 | 40.62 | 2.23 | -2.88 | -2.46 | -3.80 | High | Yes | Yes | 0 | 0.55 | 0 | 0 |
| **2j** | 272.34 | 0.50 | 3 | 2 | 0 | 40.62 | 1.95 | -2.57 | -2.09 | -3.53 | High | Yes | Yes | 0 | 0.55 | 0 | 0 |
| **2k** | 274.36 | 0.50 | 5 | 2 | 0 | 40.62 | 2.21 | -2.61 | -2.34 | -4.00 | High | Yes | No | 0 | 0.55 | 0 | 0 |
| **2o**[1] | 330.42 | 0.56 | 6 | 4 | 2 | 64.68 | 0.92 | -1.89 | -1.25 | -4.42 | High | No | Yes | 0 | 0.55 | 0 | 0 |
| **2p**[1] | 261.32 | 0.43 | 5 | 3 | 2 | 75.43 | 0.74 | -1.37 | -1.15 | -3.59 | High | No | Yes | 0 | 0.55 | 0 | 0 |
| **2q**[1] | 287.36 | 0.50 | 3 | 3 | 1 | 52.65 | 1.07 | -1.94 | -1.15 | -3.58 | High | No | Yes | 0 | 0.55 | 0 | 0 |
| **2s**[1] | 289.37 | 0.50 | 7 | 3 | 2 | 75.43 | 1.42 | -1.84 | -1.89 | -4.39 | High | No | Yes | 0 | 0.55 | 0 | 0 |
| **2t**[1] | 331.41 | 0.56 | 6 | 4 | 1 | 61.88 | 1.23 | -2.07 | -1.49 | -4.11 | High | No | Yes | 0 | 0.55 | 0 | 0 |
| **2u**[1] | 344.45 | 0.58 | 6 | 4 | 1 | 55.89 | 1.21 | -2.26 | -1.56 | -4.08 | High | No | Yes | 0 | 0.55 | 0 | 0 |
| **2v**[1] | 301.38 | 0.53 | 3 | 3 | 0 | 43.86 | 1.33 | -2.31 | -1.46 | -3.24 | High | No | Yes | 0 | 0.55 | 0 | 0 |
| **3a**[1] | 303.40 | 0.53 | 6 | 3 | 2 | 61.44 | 1.74 | -2.44 | -2.35 | -5.14 | High | Yes | Yes | 0 | 0.55 | 0 | 0 |
| **3b** | 458.59 | 0.64 | 9 | 5 | 1 | 82.19 | 2.51 | -3.85 | -3.95 | -5.26 | High | No | Yes | 0 | 0.55 | 0 | 2 |
| **3c** | 403.52 | 0.59 | 9 | 4 | 1 | 78.95 | 2.71 | -3.65 | -4.08 | -5.25 | High | No | Yes | 0 | 0.55 | 0 | 2 |
| **3d**[1] | 358.48 | 0.60 | 6 | 4 | 2 | 64.68 | 1.52 | -2.63 | -2.22 | -5.16 | High | No | Yes | 0 | 0.55 | 0 | 1 |
| **4b** | 478.58 | 0.44 | 9 | 5 | 1 | 82.19 | 2.61 | -4.12 | -4.00 | -6.43 | High | No | Yes | 0 | 0.55 | 0 | 2 |

| #cmpd | Molecular weight | Fraction Csp3 | #Rotatable bonds | #H-bond acceptors | #H-bond donors | TPSA | Consensus Log P[2] | ESOL Log S[3] | Ali Log S[3] | Silicos-IT LogSw[3] | GI absorption | BBB permeant | Pgp substrate | Lipinski #violations | Bioavailability Score | PAINS #alerts | Leadlikeness #violations |
|---|---|---|---|---|---|---|---|---|---|---|---|---|---|---|---|---|---|
| **4c** | 423.50 | 0.38 | 9 | 4 | 1 | 78.95 | 2.80 | -3.93 | -4.13 | -6.43 | High | Yes | Yes | 0 | 0.55 | 0 | 2 |
| **4d**[1] | 378.47 | 0.36 | 6 | 4 | 2 | 64.68 | 1.62 | -2.93 | -2.27 | -6.34 | High | No | Yes | 0 | 0.55 | 0 | 1 |
| **5b** | 473.57 | 0.58 | 10 | 7 | 1 | 103.78 | 1.76 | -3.08 | -3.10 | -4.64 | High | No | Yes | 0 | 0.55 | 0 | 2 |
| **5c** | 315.37 | 0.47 | 4 | 4 | 0 | 62.21 | 1.69 | -2.54 | -2.21 | -3.67 | High | Yes | Yes | 0 | 0.55 | 0 | 0 |
| **5d**[1] | 373.45 | 0.53 | 7 | 6 | 2 | 86.27 | 0.69 | -1.86 | -1.37 | -4.55 | High | No | Yes | 0 | 0.55 | 0 | 1 |
| **9a** | 466.66 | 0.11 | 2 | 3 | 2 | 73.40 | 3.44 | -5.33 | -4.85 | -6.78 | High | Yes | No | 0 | 0.85 | 0 | 2 |
| **9b** | 375.21 | 0.11 | 2 | 3 | 2 | 73.40 | 3.32 | -4.74 | -4.82 | -6.53 | High | Yes | No | 0 | 0.85 | 0 | 2 |
| **9c** | 408.76 | 0.16 | 3 | 6 | 2 | 73.40 | 3.81 | -5.00 | -5.09 | -6.77 | High | No | No | 0 | 0.85 | 0 | 2 |
| **10a** | 622.88 | 0.35 | 9 | 4 | 2 | 94.74 | 4.13 | -6.09 | -5.95 | -8.65 | High | No | Yes | 1 | 0.55 | 0 | 3 |
| **10b** | 531.43 | 0.35 | 9 | 4 | 2 | 94.74 | 4.04 | -5.51 | -5.93 | -8.43 | High | No | Yes | 1 | 0.55 | 0 | 3 |
| **10c** | 564.98 | 0.37 | 10 | 7 | 2 | 94.74 | 4.56 | -5.80 | -6.20 | -8.66 | Low | No | Yes | 1 | 0.55 | 0 | 3 |
| **13** | 219.24 | 0.33 | 2 | 3 | 1 | 57.61 | 1.33 | -2.10 | -2.01 | -2.52 | High | Yes | No | 0 | 0.85 | 0 | 1 |
| **16** | 247.29 | 0.43 | 2 | 3 | 1 | 57.61 | 1.94 | -2.83 | -2.97 | -3.28 | High | Yes | No | 0 | 0.85 | 0 | 1 |
| **18** | 323.39 | 0.30 | 4 | 3 | 0 | 46.61 | 3.35 | -4.15 | -4.15 | -5.97 | High | Yes | No | 0 | 0.55 | 0 | 0 |
| **19** | 267.28 | 0.12 | 2 | 3 | 1 | 57.61 | 2.09 | -3.18 | -3.01 | -4.47 | High | Yes | No | 0 | 0.85 | 0 | 0 |
| **20** | 204.23 | 0.27 | 1 | 3 | 1 | 50.69 | 1.49 | -2.03 | -1.76 | -3.61 | High | Yes | No | 0 | 0.55 | 0 | 1 |
| **21** | 318.37 | 0.47 | 5 | 5 | 0 | 68.20 | 2.40 | -3.06 | -3.26 | -4.18 | High | Yes | No | 0 | 0.55 | 0 | 0 |
| **22** | 262.26 | 0.31 | 3 | 5 | 1 | 79.20 | 1.13 | -2.06 | -2.13 | -2.67 | High | No | No | 0 | 0.56 | 0 | 0 |

All calculations performed with SwissADME from SMILES of neutral and uncharged compounds as recommended by the software [1]. [1]The neutral compound was used for calculations instead of the biologically tested hydrochloride form. **2a** is the free base to the hydrochloride **2r**, and **3a** is the free base to the hydrochloride **3e**; [2]Consensus clogP from ilogP, XlogP3, WlogP, MlogP and SILICOS-IT; [3]Theoretical aqueous solubility was calculated by two topological methods: ESOL [2] and one adopted from Ali *et al.* [3]; or the fragmental method from SILICOS-IT in SwissADME [1].

# References


**1**. Daina A, Michielin O, Zoete V. SwissADME: a free web tool to evaluate pharmacokinetics, drug-likeness and medicinal chemistry friendliness of small molecules. Sci Rep. 2017; 7:1–13. doi: 10.1038/srep42717.

**2**. Delaney JS. ESOL: Estimating aqueous solubility directly from molecular structure. J Chem Inf Comput Sci. 2004; 44:1000–5. doi: 10.1021/ci034243x PMID: 15154768.

**3**. Ali J, Camilleri P, Brown MB, Hutt AJ, Kirton SB. Revisiting the general solubility equation: in silico prediction of aqueous solubility incorporating the effect of topographical polar surface area. J Chem Inf Model. 2012; 52:420–8. Epub 2012/01/13. doi: 10.1021/ci200387c PMID: 22196228.
