# Supplementary figures and images for: The indole motif is essential for the antitrypanosomal activity of N5-substituted paullones
Source: PLoS One. 2023 Nov 30;18(11):e0292946. doi: 10.1371/journal.pone.0292946 (PMC10688702; doi:10.1371/journal.pone.0292946)

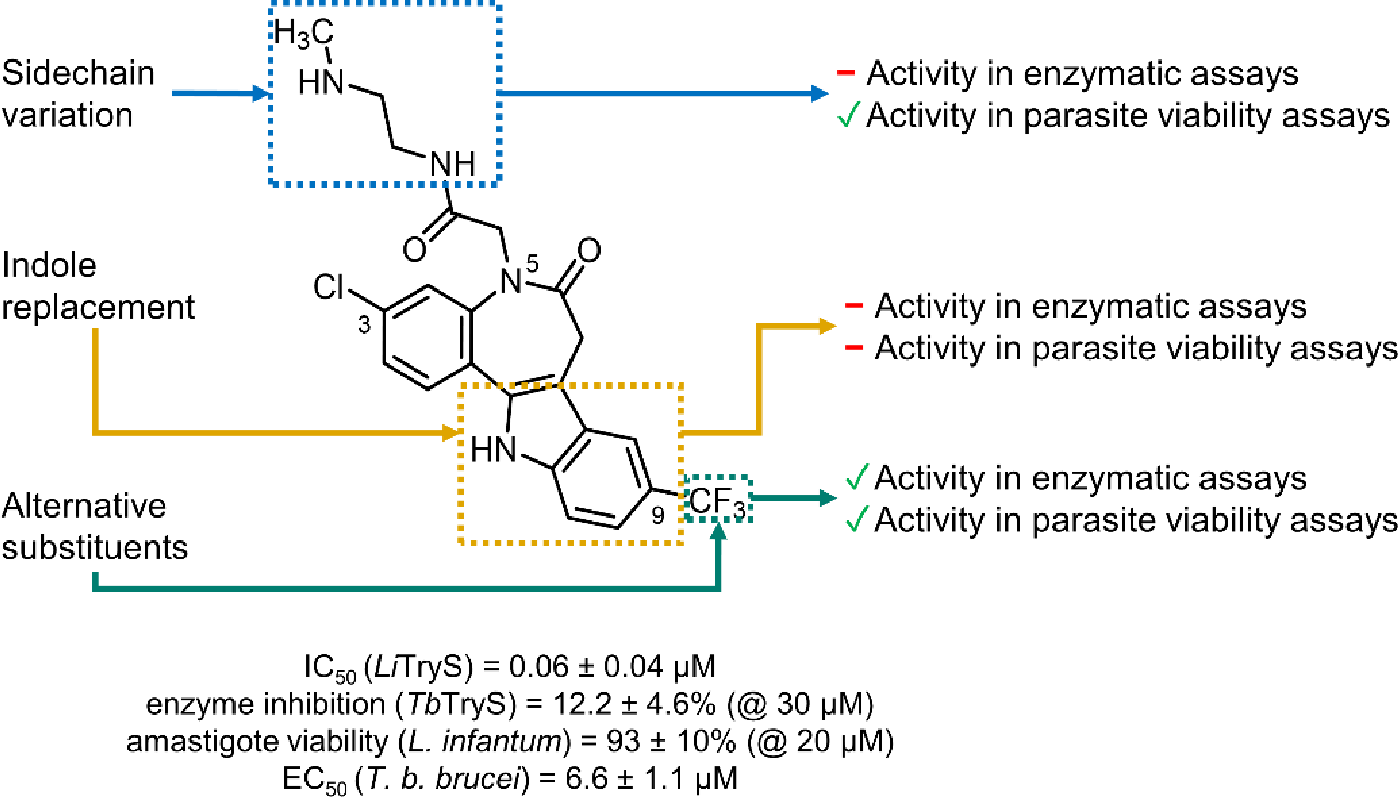

Supplement: S1 Graphical abstract — (TIF) [file pone.0292946.s007.tif]
